# S(vi) in three-component sulfonamide synthesis: use of sulfuric chloride as a linchpin in palladium-catalyzed Suzuki–Miyaura coupling†

**DOI:** 10.1039/d1sc01351c

**Published:** 2021-04-06

**Authors:** Xuefeng Wang, Min Yang, Shengqing Ye, Yunyan Kuang, Jie Wu

**Affiliations:** School of Pharmaceutical and Materials Engineering, Institute for Advanced Studies, Taizhou University 1139 Shifu Avenue Taizhou 318000 China jie_wu@fudan.edu.cn; Department of Chemistry, Fudan University 2005 Songhu Road Shanghai 200438 China; Department of Forensic Science, Gannan Medical University 1 Yixueyuan Road Ganzhou 341000 China; State Key Laboratory of Organometallic Chemistry, Shanghai Institute of Organic Chemistry, Chinese Academy of Sciences 345 Lingling Road Shanghai 200032 China; School of Chemistry and Chemical Engineering, Henan Normal University 46 East Jianshe Road Xinxiang 453007 China

## Abstract

Sulfuric chloride is used as the source of the –SO_2_– group in a palladium-catalyzed three-component synthesis of sulfonamides. Suzuki–Miyaura coupling between the *in situ* generated sulfamoyl chlorides and boronic acids gives rise to diverse sulfonamides in moderate to high yields with excellent reaction selectivity. Although this transformation is not workable for primary amines or anilines, the results show high functional group tolerance. With the solving of the desulfonylation problem and utilization of cheap and easily accessible sulfuric chloride as the source of sulfur dioxide, redox-neutral three-component synthesis of sulfonamides is first achieved.

## Abstract 2

Sulfuric chloride is used as the source of the –SO_2_– group in a palladium-catalyzed three-component synthesis of sulfonamides.

## Graphical abstract

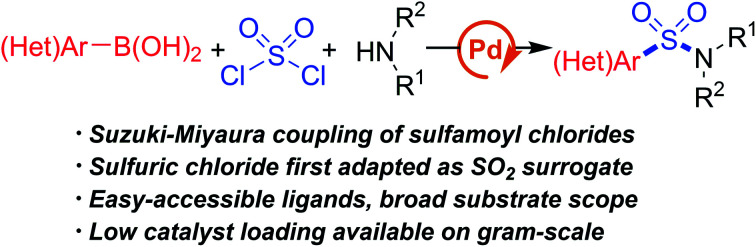

Since its development in the 1970s,^1^ Suzuki–Miyaura coupling has become a widely used synthetic step in diverse areas. With two of the most widely sourced materials, organoborons and alkyl/aryl halides, a number of C–C coupling reactions are established and the Suzuki–Miyaura reaction has successfully acted as the key step in the synthesis of medicines and agrochemicals.^2^

In addition to the well-known aryl halides and esters, various other substrates such as acid chlorides,^3^ anhydrides,^4^ diazonium salts^5^ and sulfonyl chlorides^6^ were also reported for the coupling in the past decades. As far as acid chlorides are concerned, carbamoyl chlorides were successfully transformed to the corresponding benzamides in the early years of the 21st century.^7^ However, the use of sulfamoyl chlorides as coupling partners is challenging due to the strong electron-withdrawing properties of the sulfonyl group, which cause the tendency of desulfonylation to form tertiary amines.

Synthesis of sulfonyl-containing compounds, especially sulfones and sulfonamides, *via* the insertion of sulfur dioxide has been extensively studied during the last decade.^8^ A series of sulfur-containing surrogates have been developed as the source of the –SO_2_– group. Willis and co-workers first reported the use of DABCO·(SO_2_)_2_, a bench-stable solid adduct of DABCO and gaseous SO_2_ discovered by Santos and Mello,^9*a*^ as the source of sulfur dioxide in the synthesis of sulfonylhydrazines.^9*b*^ Soon after, alkali metal metabisulfites were found to provide sulfur dioxide for the formation of sulfonyl compounds.^10^ In the recent developments in this field, DABCO·(SO_2_)_2_ and metabisulfites have become the most popular SO_2_ surrogates for the insertion of sulfur dioxide.^8^ However, the practical applications of sulfur dioxide insertion reactions are limited by atom-efficiency problems and the unique properties of reactants. For instance, the three-component synthesis of aryl sulfonylhydrazines using aryl halides, SO_2_ surrogates and hydrazines by a SO_2_-doped Buchward–Hartwig reaction was realized in the earliest developments in this field.^10^ However, similar transformations from aryl halides and amines to the corresponding sulfonamides still remain unresolved (Scheme 1a).^11,12^

**Scheme 1:**
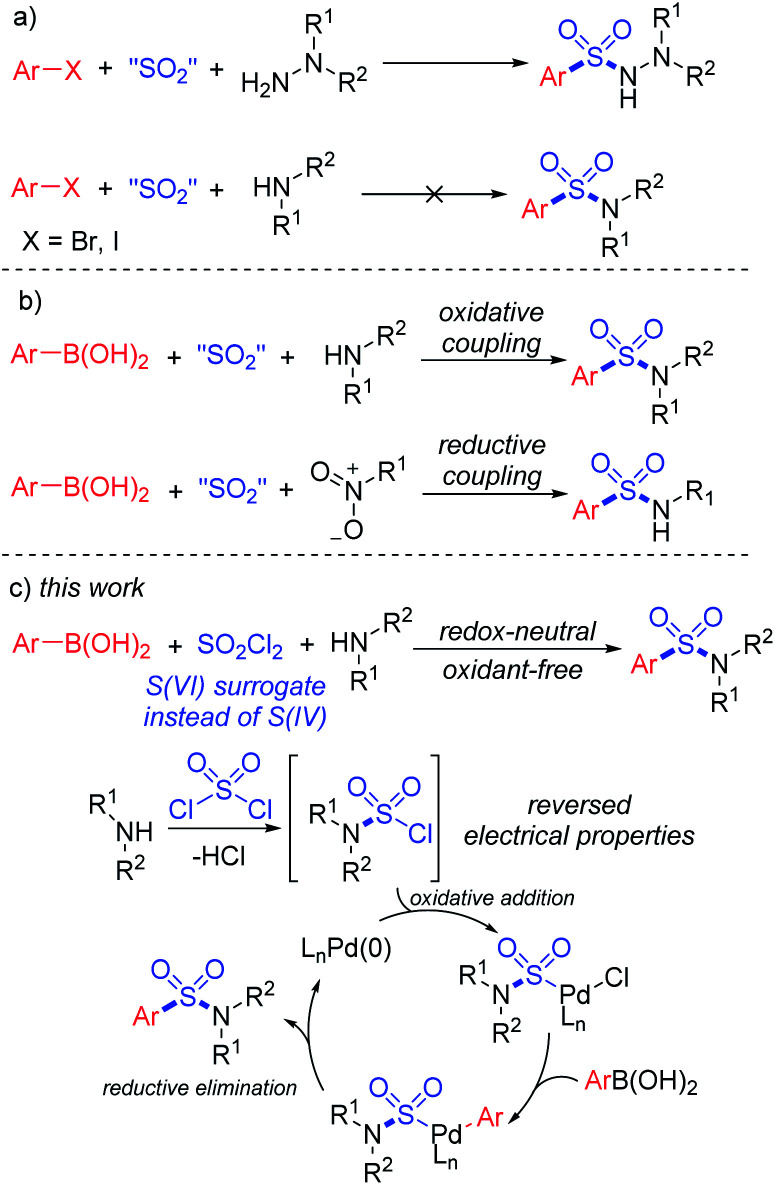
Synthetic approaches to sulfonamides.

In order to provide a simple and efficient method for the three-component synthesis of aryl sulfonamides without the pre-synthesis of sulfonyl chlorides, many scientists have made various attempts. Interestingly, the use of arylboronic acids instead of aryl halides provided an alternative route. An oxidative reaction between boronic acids, DABCO·(SO_2_)_2_ and amines for the preparation of aryl sulfonamides at high temperature was realized,^12^ while reductive couplings of boronic acids, SO_2_ surrogates and nitroarenes were also reported (Scheme 1b).^13^ However, due to the reversed electronic properties of boronic acids from halides, additional additives and restrictions had to be considered. Extra oxidants and harsh conditions were usually used, and some of the transformations required “oxidative” substrates, such as nitroarenes and chloroamines.^14^

Early in 2020, a reductive hydrosulfonamination of alkenes by sulfamoyl chlorides was reported,^15^ which gave us the inspiration to use *in situ* generated sulfamoyl chlorides as the electrophile for the synthesis of aryl sulfonamides by Suzuki–Miyaura coupling. In this way, sulfamoyl chlorides could be formed by nucleophilic substitution of an amine to sulfuric chloride, and the S(vi) central atom introduced into the reaction could reverse the electronic properties of the amine, which would eliminate the addition of oxidants (Scheme 1c). With the utilization of boronic acids as the coupling partner, a palladium-catalyzed Suzuki–Miyaura coupling could provide the sulfonamide products. Compared with traditional attempts, reversing the electronic properties of an amine from nucleophilic to electrophilic could reverse the whole reaction process, and two-step synthesis starting from the amine side could bypass the existing difficulty of S–N bond forming reductive elimination.^12^ Instead, a C–S bond formation could be the key for success (Scheme 2). In this proposed route, the presence of a base would be essential to remove the acid generated *in situ* during the reaction process. Additionally, we expected that the addition of a ligand would improve the oxidative addition of Pd(0) to sulfamoyl chloride, thus leading to the desired sulfonamide product.

**Scheme 2:**
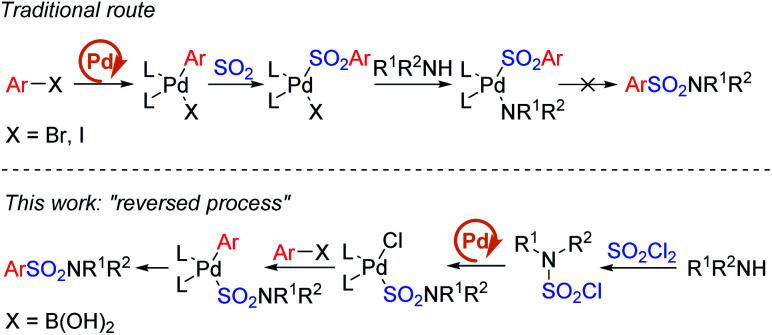
Comparison between the traditional route and designed work.

As designed based on our assumption, we used a commercialized sulfamoyl chloride intermediate **A**, which would be generated from morpholine **1a** and SO_2_Cl_2_, to start our early investigations. The results showed that the direct Suzuki–Miyaura coupling of sulfamoyl chloride intermediate **A** and 2-naphthaleneboronic acid **2a** mostly led to the generation of byproduct **3a′** with traditional phosphine ligands added to the reaction, and the desired product **3a** was obtained in poor yields (Table 1, entries 1 and 2). It is known that an electron-rich ligand would enhance the oxidative addition of Pd(0) to the electrophile, and the bulky factor would facilitate the reductive elimination process. As expected, the yield of product **3a** was increased significantly when electron-rich and bulky tris-(2,6-dimethoxyphenyl)phosphine was used as the ligand (Table 1, entry 3). Moreover, the reaction could proceed more efficiently by using a mixture of THF and MeCN as the co-solvent (Table 1, entry 4).

**Table 1:** Early investigations using morpholine-4-sulfonyl chloride **A** as the starting material

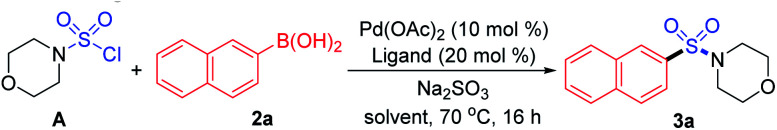			
Entry	Solvent	Ligand	Yield^a^ (%)
1	1,4-Dioxane	P^*t*^Bu_3_·HBF_4_	14
2	THF	P^*t*^Bu_3_·HBF_4_	23
3	THF	PAr_3_·Ar = 2,6-di-OMe–C_6_H_3_	57
4	THF/MeCN	PAr_3_·Ar = 2,6-di-OMe–C_6_H_3_	72

a ^1^H NMR yield obtained using 1,3,5-trimethoxybenzene as the internal standard.

With that brief conclusion in hand, we then shifted our focus to the *in situ* generation of sulfamoyl chloride intermediate **A** in the reaction process, and a number of attempts were made with morpholine **1a** and SO_2_Cl_2_ (for details, see the ESI†). After careful measurement of product **3a** and desulfonylated byproduct **3a′** generated during the transformation, the selective formation of compound **3a** was realized and “standard conditions” were identified. By using PdCl_2_(PhCN)_2_ as the catalyst and Na_2_HPO_4_ as the base, the desired product **3a** was isolated in 71% yield, giving the least amount of desulfonylated product **3a′** (Table 2, entry 1). The control experiment showed that **3a** or **3a′** was not detected in the absence of the palladium catalyst (Table 2, entry 2). It was also observed that compound **3a′** could not be generated when SO_2_Cl_2_ was omitted (Table 2, entry 3), indicating that the byproduct wasn't produced by the direct coupling of boronic acid and amine. Other changes to the catalyst, ligand, base or solvent all resulted in lower yields of compound **3a** or higher yields of desulfonylated product **3a′** (Table 2, entries 4–7).

**Table 2:** Effects of variation of reaction parameters^a^

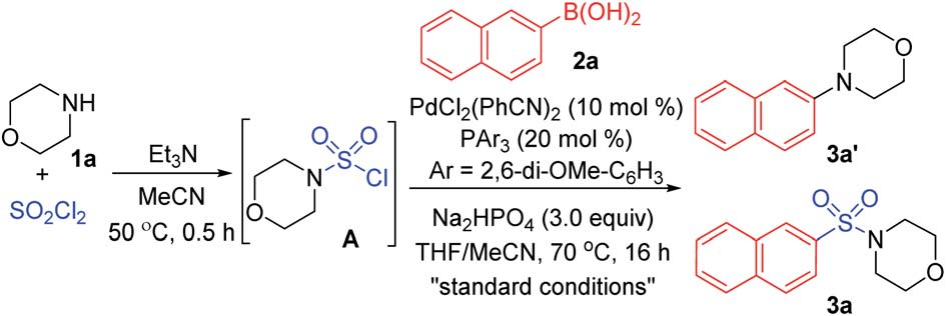			
Entry	Variation from “standard conditions”	Yield of **3a′**^b^ (%)	Yield of **3a**^b^ (%)
1	None	5	80 (69)
2	No PdCl_2_(PhCN)_2_	n.d.	n.d.
3	No SO_2_Cl_2_	n.d.	n.d.
4	Pd(OAc)_2_ instead of PdCl_2_(PhCN)_2_	13	80
5	PPh_3_ instead of PAr_3_	15	68
6	K_2_CO_3_ instead of Na_2_HPO_4_	43	23
7	MeCN instead of THF/MeCN	16	63

a Standard conditions: morpholine **1a** (0.2 mmol, 1.0 equiv.), SO_2_Cl_2_ (0.5 mmol, 2.5 equiv.), Et_3_N (0.53 mmol, 2.65 equiv.), 2-naphthaleneboronic acid **2a** (0.4 mmol, 2.0 equiv.), Na_2_HPO_4_ (0.6 mmol, 3.0 equiv.), PdCl_2_(PhCN)_2_ (10 mol%), tris-(2,6-dimethoxyphenyl)phosphine (20 mol%), THF (1.0 mL)/MeCN (1.5 mL), 70 °C, 16 h. See the ESI for the detailed procedure.

b ^1^H NMR yield obtained using 1,3,5-trimethoxybenzene as the internal standard. The isolated yield of entry 1 is shown in parentheses.

With the “standard conditions” in hand, various secondary amines **1** and arylboronic acids **2** were subjected to the reaction for the exploration of substrate adaptability (Scheme 3). To our delight, most of the reactions proceeded smoothly, giving rise to the desired product **3** in moderate to high yields. Considering the scope of boronic acids, a number of *para*-, *meta*- and *ortho*-(**3t**) substituted boronic acids showed good reactivities. However, lower yields were observed for some substrates with electron-withdrawing substituents, providing more desulfonylated byproducts due to the electron-deficiency of the palladium intermediate. Aryl boronic acids with acid-sensitive Boc-substituted amine, oxidation-sensitive phenol, sulfide and vinyl substitution were all tolerated. It is noteworthy that bromo- and acetoxy-substrates could also be efficiently converted to the corresponding products **3f** and **3r**, showing quite high selectivity during the reaction process. A series of heteroaromatic products were afforded successfully as well, and compounds with indole, indazole, dibenzothiophene and pyridine were all compatible (**3aa–3af**).

**Scheme 3:**
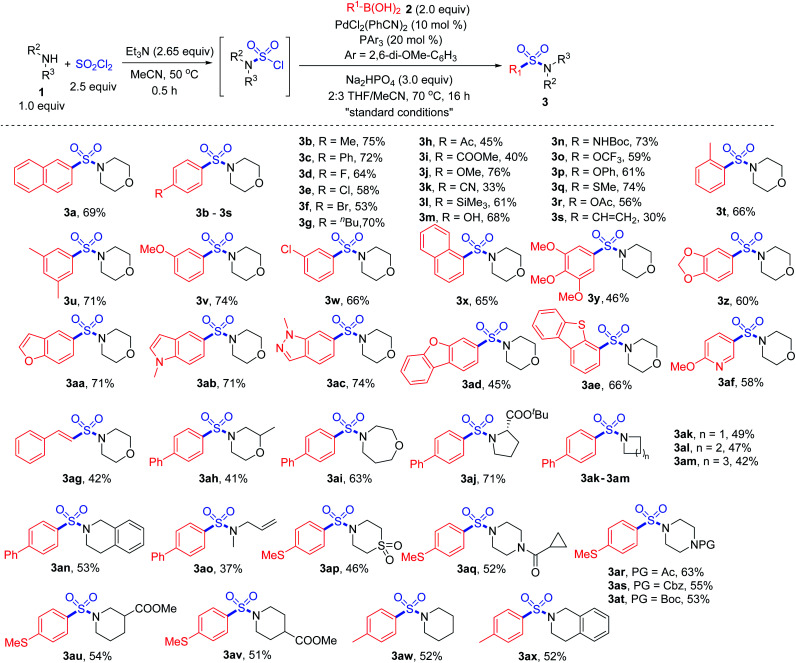
Synthesis of sulfonamides *via* a palladium-catalyzed Suzuki–Miyaura coupling. Isolated yields.

Subsequently, with respect to amines, 4-phenylboronic acid and 4-(methylthio)phenylboronic acid were selected as coupling partners based on their electronic properties and cost. Saturated cyclic products **3ah–3an** were obtained in moderate yields, among which an α-amino acid derivative showed high reactivity, giving rise to product **3aj** in 71% yield. Methylallylamine was transformed to the corresponding product **3ao** smoothly, and thiomorpholine 1,1-dioxide was also tolerated under the conditions (**3ap**). Various sensitive groups including acetyl, Boc, Cbz and cyclopropylcarbonyl (**3aq–3at**) on amines remained intact during the transformation. However, the amine scope was limited, since the transformation failed to provide the corresponding products when primary amines or anilines were used as the substrates. We assumed that during the reaction process for the oxidative addition of the sulfamoyl chloride intermediate to the palladium catalyst, Pd–SO_2_–NHR would be formed when a primary amine was used. Thus, β-hydride elimination would occur instead of the desired process.

Furthermore, the practicality of this method was also verified by gram-scale synthesis and late-stage functionalization (Scheme 4). The reaction worked smoothly on the 4.0 mmol scale, and reducing the loading amount of the palladium catalyst to 1 mol% showed no obvious impact on the transformation. With a boronic acid synthesized from estrone and desloratadine, an antihistamine drug used as the substrate, the target products **4a** and **4b** were achieved in moderate to good yields, showing potential possibilities for synthetic applications.

**Scheme 4:**
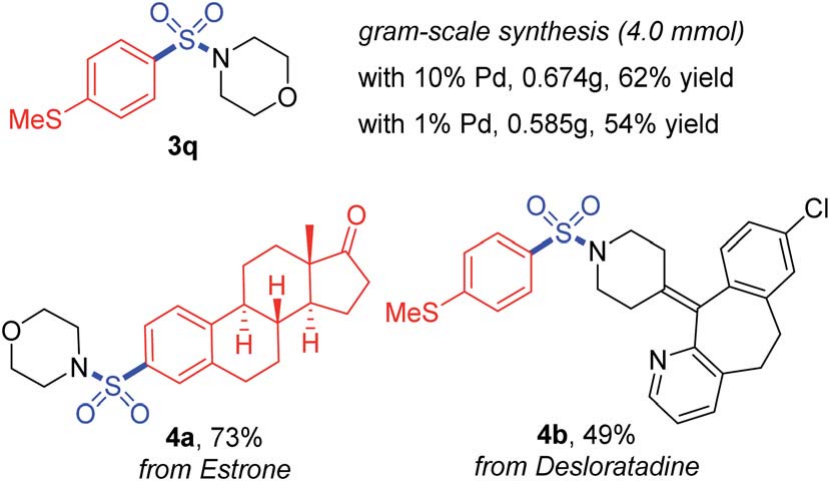
Gram-scale synthesis and late-stage functionalization.

In conclusion, a redox-neutral three-component synthesis of sulfonamides is established through a palladium-catalyzed Suzuki–Miyaura coupling of sulfuric chloride, secondary amines and arylboronic acids. Sulfuric chloride is used as the source of sulfur dioxide, and the S(vi) linchpin makes the transformation possible without the assistance of oxidants. Although this transformation is not workable for primary amines or anilines, the results show high functional group tolerance and good selectivity. A clear reaction process is described, in which the *in situ* generated sulfamoyl chloride undergoes a palladium-catalyzed Suzuki–Miyaura reaction with boronic acids, giving rise to the corresponding sulfonamide products. Additionally, the desulfonylation problem is surmounted during the reaction process. With a boronic acid synthesized from estrone and an antihistamine drug, desloratadine, used as the substrate, the target products are achieved in moderate to good yields, showing potential possibilities for synthetic applications in organic chemistry and medicinal chemistry.

## Author contributions

X. Wang and J. Wu conceived the study. X. Wang, M. Yang and S. Ye conducted the experiments and analyzed the data. J. Wu and Y. Kuang directed the project. X. Wang and J. Wu prepared the manuscript and supplemental information with input from all authors. All authors discussed the results and commented on the manuscript.

## Conflicts of interest

There are no conflicts to declare.

## Supplementary Material

SC-012-D1SC01351C-s001
