# Supplementary material for: S(vi) in three-component sulfonamide synthesis: use of sulfuric chloride as a linchpin in palladium-catalyzed Suzuki–Miyaura coupling
Source: Chem Sci. 2021 Apr 6;12(18):6437–41. doi: 10.1039/d1sc01351c (PMC8115270; doi:10.1039/d1sc01351c)
Supplement: SC-012-D1SC01351C-s001 [file SC-012-D1SC01351C-s001.pdf]

Electronic Supplementary Material (ESI) for Chemical Science.
This journal is © The Royal Society of Chemistry 2021

S(VI) in Three-Component Sulfonamide Synthesis: Use of Sulfuric Chloride as Linchpin in Palladium-Catalyzed Suzuki-Miyaura Coupling

Xuefeng Wang, Min Yang, Shengqing Ye, Yunyan Kuang and Jie Wu*

Supporting Information

1. General experimental methods (S2).
2. Early investigations using morpholine-4-sulfonyl chloride (S3).
3. Optimization of "standard conditions" (S4–S6).
4. General experimental procedure (S7–S8).
5. Characterization data (S9 – S24).
6. ^{1}H, ^{13}C and ^{19}F NMR spectra of compounds **3** and **4** (S25 – S79).

1. General experimental methods:

Unless otherwise stated, all commercial reagents were used as received. $PdCl_2(PhCN)_2$, phosphine ligands, boronic acids and amines (except morpholine) are purchased from *Bidepharm* and used as received. Morpholine(99.9%, GC) and tetrahydrofuran (THF, 99.5%, *Energyseal*, Extra Dry, with molecular sieves, Water≤50 ppm) were purchased from *Energy Chemical* and used as received. Acetonitrile (MeCN, 99.9%, SafeDry, with molecular sieves, Water≤50 ppm) was purchased from *Adamas-Beta* and used as received. Flash column chromatography was performed using silica gel (300-400 mesh, standard grade). Analytical thin–layer chromatography was performed using glass plates pre-coated with 0.25 mm 230–400 mesh silica gel impregnated with a fluorescent indicator (254 nm). Thin layer chromatography plates were visualized by exposure to 254 nm ultraviolet light or iodine stain. Organic solutions were concentrated on rotary evaporators at ~20 Torr at 30–50°C. Nuclear magnetic resonance (NMR) spectra are recorded in parts per million(ppm) from solvent residual peak on the δ scale. ^{1}H, ^{13}C and ^{19}F NMR spectra were recorded in chloroform-*d* or acetone-d_6 on a Bruker DRX-400 spectrometer operating at 400 MHz, 101 MHz and 376 MHz, respectively. All chemical shift values are quoted in ppm and coupling constants quoted in Hz. Melting points are tested automatically on a Melting Point Apparatus produced by *Shanghai JINGMI Scientific Instruments Co., Ltd.* High resolution mass spectrometry (HRMS) spectra were obtained on a Bruker McriOTOF11 Instrument.

2. Early investigations using Morpholine-4-sulfonyl chloride

Yields were determined by ^{1}H NMR using 1,3,5-trimethoxybenzene as internal standard.

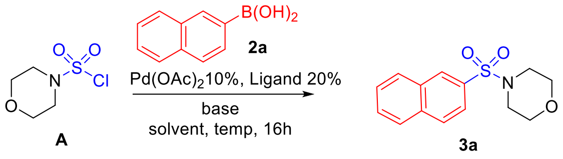

Entry	Solvent	Ligand	Base	Temp.	Yield **3a** (%)
1	dioxane	P^tBu_3-HBF_4	Na_2CO_3	70	n.d.
2	dioxane	P^tBu_3-HBF_4	K_2CO_3	70	n.d.
3	dioxane	P^tBu_3-HBF_4	KOAc	70	n.d.
4	dioxane	P^tBu_3-HBF_4	Na_2SO_3	70	14
5	dioxane	P^tBu_3-HBF_4	Na_2HPO_4	70	trace
6	dioxane	P^tBu_3-HBF_4	K_2HPO_4	70	trace
7	DMF	P^tBu_3-HBF_4	Na_2SO_3	70	trace
8	MeCN	P^tBu_3-HBF_4	Na_2SO_3	70	15
9	*p*-Xylene	P^tBu_3-HBF_4	Na_2SO_3	70	n.d.
10	DME	P^tBu_3-HBF_4	Na_2SO_3	70	n.d.
11	THF	P^tBu_3-HBF_4	Na_2SO_3	70	23
12	THF	dppe, dcpp-HBF_4, XPhos, ^{t}BuXPhos, P(*o*-tol)$_3$, JohnPhos, SPhos, BINAP, dppf	Na_2SO_3	70	n.d.
13	THF	PPh_3	Na_2SO_3	70	trace
14	THF	PAr_3 Ar=2,6-di-OMe-C_6H_3	Na_2SO_3	70	57
15	THF	DavePhos	Na_2SO_3	70	30
16	THF	BrettPhos	Na_2SO_3	70	45
17	THF/MeCN	PAr_3 Ar=2,6-di-OMe-C_6H_3	Na_2SO_3	70	67
18	THF/MeCN	PAr_3 Ar=2,5-di-Me-C_6H_3	Na_2SO_3	70	30
19	THF/MeCN	PAr_3 Ar=*o*-OMe-C_6H_4	Na_2SO_3	70	50
20	THF/MeCN	PAr_3 Ar=*m*-OMe-C_6H_4	Na_2SO_3	70	62
21	THF/MeCN	PAr_3 Ar=*p*-OMe-C_6H_4	Na_2SO_3	70	66
**22**	**THF/MeCN**	**PAr_3 Ar=2,6-di-OMe-C_6H_3**	**Na_2SO_3**	**70**	**72**

3. Optimization of "standard conditions"

Yields were determined by ^{1}H NMR using 1,3,5-trimethoxybenzene as internal standard.

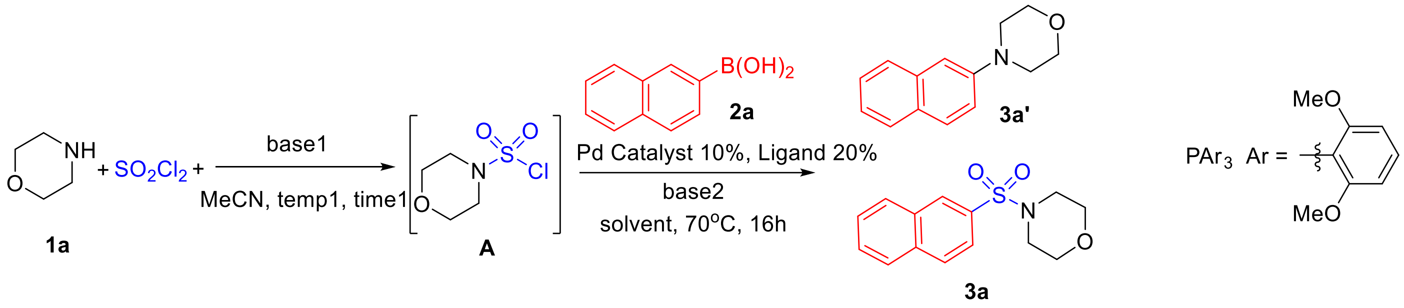

Entry	SO_2Cl_2(eq)	Base1 (eq)	Temp1(°C)	Time(h)	Pd catalyst	Ligand	Base2 (3.0 eq)	Solvent (ratio)	**3a'** yield(%)	**3a** yield (%)
1	1.5	Et_3N(1.2)	r.t.	0.1667	$Pd(OAc)_2$	PAr_3	Na_2SO_3	THF/MeCN 1:1	15	39
2	1.5	Et_3N(1.2)	r.t.	0.5	$Pd(OAc)_2$	PAr_3	Na_2SO_3	THF/MeCN 1:1	40	36
3	1.5	Et_3N(1.5)	r.t.	0.5	$Pd(OAc)_2$	PAr_3	Na_2SO_3	THF/MeCN 1:1	30	51
4	2.0	Et_3N(1.2)	r.t.	0.5	$Pd(OAc)_2$	PAr_3	Na_2SO_3	THF/MeCN 1:1	60	26
5	1.5	Et_3N(1.2)	r.t.	0.5	$Pd(OAc)_2$	PAr_3	Na_2SO_3	THF/MeCN 1:1	63	20(**2a** 1.0 eq)
6	1.5	Et_3N(2.1)	r.t.	0.5	$Pd(OAc)_2$	PAr_3	Na_2SO_3	THF/MeCN 1:1	23	50
7	1.5	DIPEA(1.5)	r.t.	0.5	$Pd(OAc)_2$	PAr_3	Na_2SO_3	THF/MeCN 1:1	27	36
8	1.5	Et_3N(1.5)	Reflux	0.5	$Pd(OAc)_2$	PAr_3	Na_2SO_3	THF/MeCN 1:1	24	39
9	1.5	Et_3N(2.0)	Reflux	0.5	$Pd(OAc)_2$	PAr_3	Na_2SO_3	THF/MeCN 1:1	15	34
10	1.8	Et_3N(1.8)	Reflux	0.5	$Pd(OAc)_2$	PAr_3	Na_2SO_3	THF/MeCN 1:1	24	42
11	1.8	Et_3N(2.4)	reflux	0.5	$Pd(OAc)_2$	PAr_3	Na_2SO_3	THF/MeCN 1:1	16	42

12	1.8	Et_3N(3.0)	reflux	0.5	$Pd(OAc)_2$	PAr_3	Na_2SO_3	THF/MeCN 1:1	20	37
Entry	SO_2Cl_2(eq)	Base1 (eq)	Temp1(°C)	Time(h)	Pd catalyst	Ligand	Base2 (3.0 eq)	Solvent (ratio)	**3a’** yield(%)	**3a** yield (%)
13	1.8	Et_3N(2.0)	r.t.	0.5	$Pd(OAc)_2$	PAr_3	Na_2SO_3	THF/MeCN 1:1	23	50
14	1.8	Et_3N(2.0)	50	0.5	$Pd(OAc)_2$	PAr_3	Na_2SO_3	THF/MeCN 1:1	26	55
15	1.8	Et_3N(2.0)	70	0.5	$Pd(OAc)_2$	PAr_3	Na_2SO_3	THF/MeCN 1:1	25	44
16	1.8	Et_3N(2.0)	90	0.5	$Pd(OAc)_2$	PAr_3	Na_2SO_3	THF/MeCN 1:1	32	30
17	1.8	Et_3N(2.0)	50	1.0	$Pd(OAc)_2$	PAr_3	Na_2SO_3	THF/MeCN 1:1	24	53
18	1.8	Et_3N(2.0)	50	1.5	$Pd(OAc)_2$	PAr_3	Na_2SO_3	THF/MeCN 1:1	26	48
19	1.8	Et_3N(2.0)	50	2.0	$Pd(OAc)_2$	PAr_3	Na_2SO_3	THF/MeCN 1:1	20	47
20	1.8	Et_3N(2.0)	50	0.5	$Pd(OAc)_2$	PAr_3	Na_2SO_3	THF/MeCN 1:1	38	50
21	2.5	Et_3N(2.65)	50	0.5	$Pd(OAc)_2$	PAr_3	Na_2SO_3	THF/MeCN 1:1	31	64
22	3.0	Et_3N(3.1)	50	0.5	$Pd(OAc)_2$	PAr_3	Na_2SO_3	THF/MeCN 1:1	52	33
23	2.5	Et_3N(1.0)	50	0.5	$Pd(OAc)_2$	PAr_3	Na_2SO_3	THF/MeCN 1:1	40	32
24	2.5	Et_3N(1.2)	50	0.5	$Pd(OAc)_2$	PAr_3	Na_2SO_3	THF/MeCN 1:1	29	37
25	2.5	Et_3N(1.25)	50	0.5	$Pd(OAc)_2$	PAr_3	Na_2SO_3	THF/MeCN 1:1	39	33
26	2.5	Et_3N(1.325)	50	0.5	$Pd(OAc)_2$	PAr_3	Na_2SO_3	THF/MeCN 1:1	32	31
27	2.5	Et_3N(1.5)	50	0.5	$Pd(OAc)_2$	PAr_3	Na_2SO_3	THF/MeCN 1:1	23	36
28	2.5	Et_3N(2.0)	50	0.5	$Pd(OAc)_2$	PAr_3	Na_2SO_3	THF/MeCN 1:1	17	41
29	2.5	Et_3N(2.4)	50	0.5	$Pd(OAc)_2$	PAr_3	Na_2SO_3	THF/MeCN 1:1	22	26
30	2.5	Et_3N(2.65)	50	0.5	$Pd(OAc)_2$	PAr_3	Na_2SO_3	THF/MeCN 1:1	41	54

31	2.5	Et_3N(2.65)	50	0.5	$PdCl_2$	PAr_3	Na_2SO_3	THF/MeCN 1:1	23	47
32	2.5	Et_3N(2.65)	50	0.5	$PdBr_2$	PAr_3	Na_2SO_3	THF/MeCN 1:1	13	66
33	2.5	Et_3N(2.65)	50	0.5	$Pd(PPh_3)_2Cl_2$	PAr_3	Na_2SO_3	THF/MeCN 1:1	22	51
Entry	SO_2Cl_2(eq)	Base1 (eq)	Temp1(°C)	Time(h)	Pd catalyst	Ligand	Base2 (3.0 eq)	Solvent (ratio)	**3a'** yield(%)	**3a** yield (%)
34	2.5	Et_3N(2.65)	50	0.5	$Pd(PhCN)_2Cl_2$	PAr_3	Na_2SO_3	THF/MeCN 1:1	16	68
35	2.5	Et_3N(2.65)	50	0.5	$Pd(PhCN)_2Cl_2$	PAr_3	Na_2SO_3	THF/MeCN 1:1	18	73
36	2.5	Et_3N(2.65)	50	0.5	$Pd(PhCN)_2Cl_2$	PAr_3	Na_2SO_3	THF/MeCN 3:2	18	68
37	2.5	Et_3N(2.65)	50	0.5	$Pd(PhCN)_2Cl_2$	PAr_3	Na_2SO_3	THF/MeCN 2:1	19	63
38	2.5	Et_3N(2.65)	50	0.5	$Pd(PhCN)_2Cl_2$	PAr_3	Na_2SO_3	THF/MeCN 2:3	9	68
39	2.5	Et_3N(2.65)	50	0.5	$Pd(PhCN)_2Cl_2$	PAr_3	Na_2SO_3	dioxane/MeCN 1:1	24	66
40	2.5	Et_3N(2.65)	50	0.5	$Pd(PhCN)_2Cl_2$	PAr_3	Na_2SO_3	dioxane/MeCN 3:2	26	57
41	2.5	Et_3N(2.65)	50	0.5	$Pd(PhCN)_2Cl_2$	PAr_3	K_2HPO_4	THF/MeCN 2:3	33	50
**42**	**2.5**	**Et_3N(2.65)**	**50**	**0.5**	**$Pd(PhCN)_2Cl_2$**	**PAr_3**	**Na_2HPO_4**	**THF/MeCN 2:3**	**5**	**80**
43	2.5	Et_3N(2.65)	50	0.5	$Pd(PhCN)_2Cl_2$	PAr_3	$K_2S_2O_5$	THF/MeCN 2:3	11	78
44	2.5	Et_3N(2.65)	50	0.5	$Pd(PhCN)_2Cl_2$	PAr_3	$Na_2S_2O_5$	THF/MeCN 2:3	18	68

Entries 23-29: Et_3N added to SO_2Cl_2 solution in MeCN, then morpholine added.

4. General experimental procedure:

4.1 Procedure of Palladium-catalyzed coupling reaction

*General experimental procedure for the reaction of secondary amine **1**, SO_2Cl_2 and boronic acid **2**. Procedure to product **3aa** is shown below as an example.*

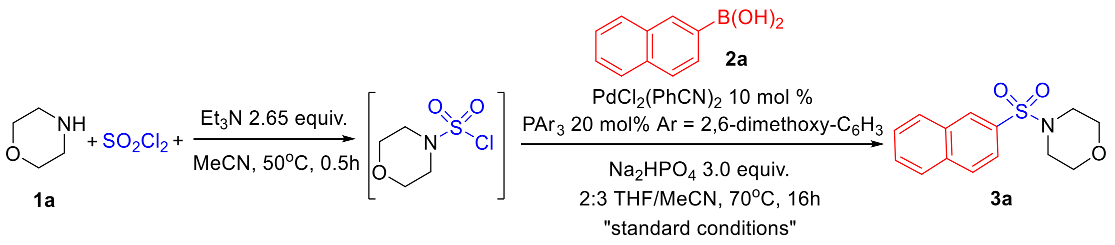

A solution of SO_2Cl_2 (0.5 mmol, 2.5 equiv) in MeCN (1.0 mL) was cooled to 0 °C before morpholine **1a*** (0.2 mmol, 1.0 equiv) was added dropwisely. Then Et_3N (0.53 mmol, 2.65 equiv) was added dropwisely and the mixture was heated to 50 °C for 0.5 h. After the scheduled time, the mixture was cooled to -15 °C for Et_3N-HCl to precipitate. The solution was filtered and transferred to a rubber-septa-sealed tube containing a mixture of 2-naphthaleneboronic acid **2a** (0.4 mmol, 2.0 equiv), $PdCl_2(PhCN)_2$ (0.02 mmol, 10 mol %), tris(2,6-dimethoxyphenyl)phosphine (0.04 mmol, 20 mol %), and Na_2HPO_4 (0.6 mmol, 3.0 equiv) in THF (1.0 mL) under argon. Followed by addition of MeCN (0.5 mL). The mixture was then stirred at 70 °C for 16 h. After the scheduled time, the solvent was evaporated under reduced pressure and the residue was purified directly by flash column chromatography (eluent: 20% EtOAc/*n*-hexane) to afford the corresponding product **3aa** as a white solid (38.1 mg, 69% yield).

* In cases that amine hydrochloride was used, the amine hydrochloride was treated by 1.0 equiv Et_3N in minimum amount of MeCN before addition to the SO_2Cl_2 solution.

4.2 Procedure of the Gram-scale synthesis of product 3q

4.2.1 Procedure using 10% Pd catalyst

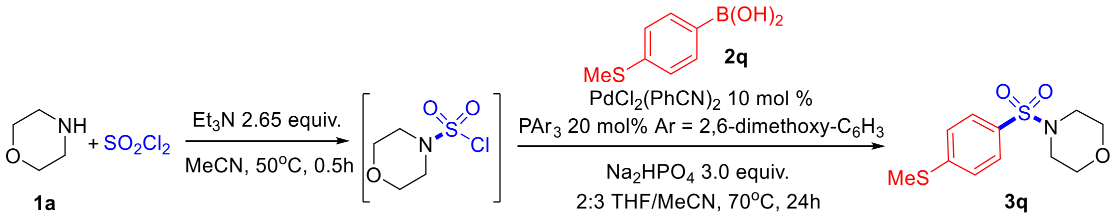

A solution of SO_2Cl_2 (10 mmol, 2.5 equiv) in MeCN (20 mL) was cooled to 0 °C

before morpholine **1a** (4 mmol, 1.0 equiv) was added dropwisely. Then Et_3N (10.6 mmol, 2.65 equiv) was added dropwisely and the mixture was heated to 50 °C for 0.5 h. After the scheduled time, the mixture was cooled to -15 °C for Et_3N-HCl to precipitate. The solution was filtered and transferred to a rubber-septa-sealed flask containing a mixture of (4-(methylthio)phenyl)boronic acid **2q** (8.0 mmol, 2.0 equiv), $PdCl_2(PhCN)_2$ (0.4 mmol, 10 mol %), tris(2,6-dimethoxyphenyl)phosphine (0.8 mmol, 20 mol %), and Na_2HPO_4 (12.0 mmol, 3.0 equiv) in THF (20 mL) under argon. Followed by addition of MeCN (10 mL). The mixture was then stirred at 70 °C for 24 h. After the scheduled time, the solvent was evaporated under reduced pressure and the residue was purified directly by flash column chromatography (eluent: 20% EtOAc/*n*-hexane) to afford the corresponding product **3q** as a white solid (0.674 g, 62% yield).

4.2.2 Procedure using 1% Pd catalyst

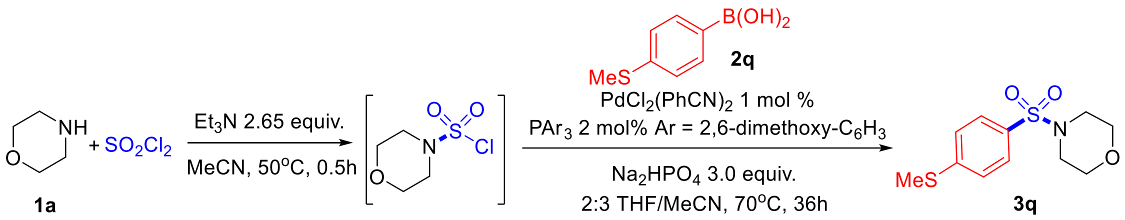

A solution of SO_2Cl_2 (10 mmol, 2.5 equiv) in MeCN (20 mL) was cooled to 0 °C before morpholine **1a** (4 mmol, 1.0 equiv) was added dropwisely. Then Et_3N (10.6 mmol, 2.65 equiv) was added dropwisely and the mixture was heated to 50 °C for 0.5 h. After the scheduled time, the mixture was cooled to -15 °C for Et_3N-HCl to precipitate. The solution was filtered and transferred to a rubber-septa-sealed flask containing a mixture of (4-(methylthio)phenyl)boronic acid **2q** (8.0 mmol, 2.0 equiv), $PdCl_2(PhCN)_2$ (0.04 mmol, 1 mol %), tris(2,6-dimethoxyphenyl)phosphine (0.08 mmol, 2 mol %), and Na_2HPO_4 (12.0 mmol, 3.0 equiv) in THF (20 mL) under argon. Followed by addition of MeCN (10 mL). The mixture was then stirred at 70 °C for 36 h. After the scheduled time, the solvent was evaporated under reduced pressure and the residue was purified directly by flash column chromatography (eluent: 20% EtOAc/*n*-hexane) to afford the corresponding product **3q** as a white solid (0.585 g, 54% yield).

5. Characterization data

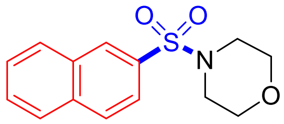

4-(Naphthalen-2-ylsulfonyl)morpholine (**3a**)[1]

38.1 mg, 69% yield. White solid. M.p. 153.9 – 154.9 °C.

^{1}H NMR (400 MHz, $CDCl_3$) δ 8.34 (s, 1H), 8.00 (d, *J* = 8.9 Hz, 2H), 7.94 (d, *J* = 7.9 Hz, 1H), 7.75 (dd, *J* = 8.6, 1.8 Hz, 1H), 7.70 – 7.61 (m, 2H), 3.75 (t, *J* = 4.8 Hz, 4H), 3.10 – 3.05 (t, *J* = 4.8 Hz, 4H). ^{13}C NMR (101 MHz, $CDCl_3$) δ 135.14, 132.35, 129.46, 129.39, 129.14, 128.11, 127.83, 123.11, 66.29, 46.23.

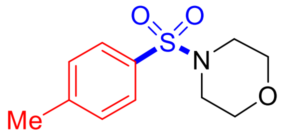

4-Tosylmorpholine (**3b**) [2]

36.1 mg, 75% yield. White solid. M.p. 139.7 – 140.5 °C.

^{1}H NMR (400 MHz, $CDCl_3$) δ 7.63 (d, *J* = 8.0 Hz, 2H), 7.34 (d, *J* = 8.0 Hz, 2H), 3.76 – 3.70 (t, *J* = 4.4 Hz, 4H), 3.01 – 2.95 (t, *J* = 4.8 Hz, 4H), 2.44 (s, 3H). ^{13}C NMR (101 MHz, $CDCl_3$) δ 144.07, 132.25, 129.87, 128.03, 66.23, 46.12, 21.66.

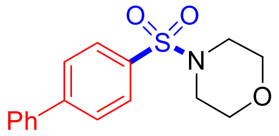

4-([1,1'-Biphenyl]-4-ylsulfonyl)morpholine (**3c**) [1]

43.7 mg, 72% yield. Off-white solid. M.p. 206.5 – 208.8 °C.

^{1}H NMR (400 MHz, $CDCl_3$) δ 7.82 (d, *J* = 8.3 Hz, 2H), 7.75 (d, *J* = 8.4 Hz, 2H), 7.61 (d, *J* = 7.4 Hz, 2H), 7.51 – 7.41 (m, 3H), 3.82 – 3.71 (t, *J* = 4.8 Hz, 4H), 3.11 – 3.01 (t, *J* = 4.8 Hz, 4H). ^{13}C NMR (101 MHz, $CDCl_3$) δ 146.19, 139.31, 133.85, 129.24, 128.74, 128.52, 127.89, 127.48, 66.27, 46.16.

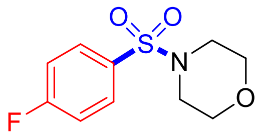

4-((4-Fluorophenyl)sulfonyl)morpholine (**3d**) [1]

31.2 mg, 64% yield. White solid. M.p. 92.5 – 92.7 °C.

^{1}H NMR (400 MHz, $CDCl_3$) δ 7.81 – 7.74 (m, 2H), 7.28 – 7.20 (m, 2H), 3.76 – 3.73 (t, *J* = 4.8 Hz, 4H), 3.01 – 2.98 (t, *J* = 4.8 Hz, 4H). ^{13}C NMR (101 MHz, $CDCl_3$) δ 165.53 (d, J_F = 256.6 Hz), 131.47, 130.66 (d, J_F = 9.4 Hz), 116.58 (d, J_F = 22.6 Hz), 66.19, 46.10. ^{19}F NMR (376 MHz, $CDCl_3$) δ -104.50.

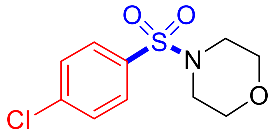

4-((4-Chlorophenyl)sulfonyl)morpholine (**3e**)[1]

30.2 mg, 58% yield. White solid. M.p. 145.2 – 146.6 °C.

^{1}H NMR (400 MHz, $CDCl_3$) δ 7.69 (d, *J* = 8.6 Hz, 2H), 7.53 (d, *J* = 8.6 Hz, 2H), 3.76 – 3.72 (t, *J* = 4.8 Hz, 4H), 3.02 – 2.98 (t, *J* = 4.8 Hz, 4H). ^{13}C NMR (101 MHz, $CDCl_3$) δ 139.89, 133.93, 129.63, 129.37, 66.19, 46.08.

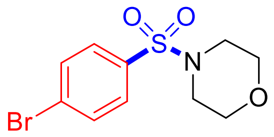

4-((4-Bromophenyl)sulfonyl)morpholine (**3f**)[3]

32.4 mg, 53% yield. White solid. M.p. 148.8 – 150.0 °C.

^{1}H NMR (400 MHz, $CDCl_3$) δ 7.72 – 7.68 (m, 2H), 7.64 – 7.59 (m, 2H), 3.74 (t, *J* = 4.8 Hz, 4H), 3.00 (t, *J* = 4.8 Hz, 4H). ^{13}C NMR (101 MHz, $CDCl_3$) δ 134.47, 132.62, 129.44, 128.37, 66.19, 46.07.

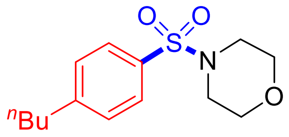

4-((4-Butylphenyl)sulfonyl)morpholine (**3g**)

39.7 mg, 70% yield. White solid. M.p. 86.2 – 87.9 °C.

^{1}H NMR (400 MHz, $CDCl_3$) δ 7.65 (d, *J* = 8.3 Hz, 2H), 7.34 (d, *J* = 8.2 Hz, 2H), 3.73 (t, *J* = 4.8 Hz, 4H), 2.98 (t, *J* = 4.8 Hz, 4H), 2.68 (t, *J* = 8.0 Hz, 2H), 1.62 (dt, *J* = 15.4, 7.6 Hz, 2H), 1.40 – 1.31 (m, 2H), 0.93 (t, *J* = 7.3 Hz, 3H). HRMS(ESI) Calc. for $C_{14}H_{22}NO_3S^+$(M + H^+): 284.1315, found: 284.1318.

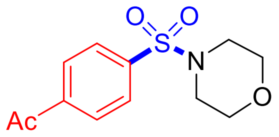

1-(4-(Morpholinosulfonyl)phenyl)ethan-1-one (**3h**)[4]

24.2 mg, 45% yield. White solid. M.p. 131.8 – 132.1 °C.

^{1}H NMR (400 MHz, $CDCl_3$) δ 8.10 (d, *J* = 8.6 Hz, 2H), 7.84 (d, *J* = 8.6 Hz, 2H), 3.76 – 3.72 (m, 4H), 3.04 – 2.99 (t, *J* = 4.8 Hz, 4H), 2.66 (s, 3H). ^{13}C NMR (101 MHz, $CDCl_3$) δ 196.80, 140.46, 139.34, 129.06, 128.22, 66.18, 46.06, 27.00.

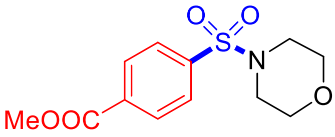

Methyl 4-(morpholinosulfonyl)benzoate (**3i**)[4]

22.8 mg, 40% yield. White solid. M.p. 140.2 – 142.3 °C.

^{1}H NMR (400 MHz, $CDCl_3$) δ 8.21 (d, *J* = 8.4 Hz, 2H), 7.82 (d, *J* = 8.4 Hz, 2H), 3.97 (s, 3H), 3.76 – 3.72 (m, 4H), 3.05 – 3.00 (m, 4H). ^{13}C NMR (101 MHz, $CDCl_3$) δ 165.68, 139.45, 134.40, 130.47, 127.94, 66.21, 52.85, 46.09.

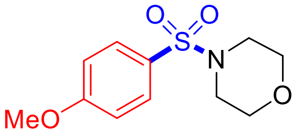

4-((4-Methoxyphenyl)sulfonyl)morpholine (**3j**) [1]

38.9 mg, 76% yield. White solid. M.p. 106.5 – 108.7 °C.

^{1}H NMR (400 MHz, $CDCl_3$) δ 7.71 – 7.66 (m, 2H), 7.03 – 6.98 (m, 2H), 3.87 (s, 3H), 3.73 (t, *J* = 4.8 Hz, 4H), 2.97 (t, *J* = 4.8 Hz, 4H). ^{13}C NMR (101 MHz, $CDCl_3$) δ 163.37, 130.11, 126.83, 114.43, 66.22, 55.77, 46.13.

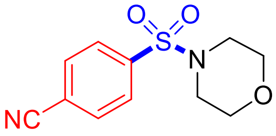

4-(Morpholinosulfonyl)benzonitrile (**3k**)[4]

16.7 mg, 33% yield. White solid. M.p. 155.7 - 158.1 °C.

^{1}H NMR (400 MHz, $CDCl_3$) δ 7.87 (s, 4H), 3.77 – 3.74 (m, 4H), 3.04 (t, *J* = 4.8 Hz, 4H). ^{13}C NMR (101 MHz, $CDCl_3$) δ 140.04, 133.11, 128.51, 117.26, 117.05, 66.18, 46.04.

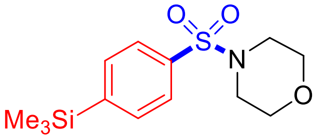

4-((4-(Trimethylsilyl)phenyl)sulfonyl)morpholine (**3l**) [1]

36.1 mg, 61% yield. White powder. M.p. 144.9 – 145.8 °C.

^{1}H NMR (400 MHz, $CDCl_3$) δ 7.71 – 7.66 (m, 4H), 3.75 – 3.71 (m, 4H), 3.02 – 2.98 (m, 4H), 0.30 (s, 9H). ^{13}C NMR (101 MHz, $CDCl_3$) δ 147.61, 135.26, 134.03, 126.83, 66.22, 46.08, -1.27.

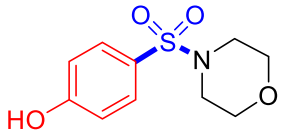

4-(Morpholinosulfonyl)phenol (**3m**)[4]

32.9 mg, 68% yield. White solid. M.p. 144.1 – 147.3 °C.

^{1}H NMR (400 MHz, CD_3CN) δ 7.86 (broad, 1H), 7.62 – 7.57 (m, 2H), 7.00 – 6.97 (m, 2H), 3.65 (t, J = 4.8 Hz, 4H), 2.87 (t, J = 4.8 Hz, 4H). ^{13}C NMR (101 MHz, CD_3CN) δ 162.18, 131.21, 118.31, 116.67, 66.70, 47.13.

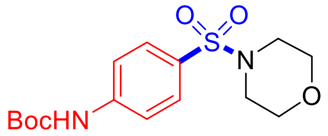

tert-Butyl (4-(morpholinosulfonyl)phenyl)carbamate (**3n**)[5]

47.3 mg,73% yield. White solid. M.p. 162.7 – 163.9 °C.

^{1}H NMR (400 MHz, $CDCl_3$) δ 7.66 (d, J = 8.7 Hz, 2H), 7.54 (d, J = 8.8 Hz, 2H), 6.88 (d, J = 13.6 Hz, 1H), 3.74 – 3.70 (m, 4H), 2.99 – 2.94 (m, 4H), 1.52 (s, 9H). ^{13}C NMR (101 MHz, $CDCl_3$) δ 152.25, 143.16, 129.33, 128.58, 118.03, 81.78, 66.21, 46.12, 28.36.

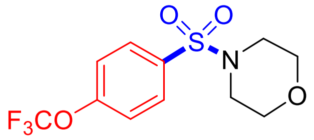

4-((4-(Trifluoromethoxy)phenyl)sulfonyl)morpholine (**3o**) [1]

36.2 mg, 59% yield. Colorless oil.

^{1}H NMR (400 MHz, $CDCl_3$) δ 7.83 – 7.79 (m, 2H), 7.38 (d, J = 8.1 Hz, 2H), 3.77 – 3.73 (t, J = 4.8 Hz, 4H), 3.03 – 2.99 (t, J = 4.8 Hz, 4H). ^{13}C NMR (101 MHz, $CDCl_3$) δ 152.62, 133.83, 130.09, 121.10, 120.36(q, J_F = 260.7 Hz), 66.18, 46.07. ^{19}F NMR (376 MHz, $CDCl_3$) δ -57.72.

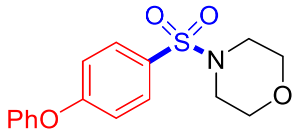

4-((4-Phenoxyphenyl)sulfonyl)morpholine (**3p**)

38.4 mg, 61% yield. White solid. M.p. 149.4 – 150.6 °C.

^{1}H NMR (400 MHz, $CDCl_3$) δ 7.72 – 7.67 (m, 2H), 7.42 (t, *J* = 7.9 Hz, 2H), 7.23 (t, *J* = 7.5 Hz, 1H), 7.10 – 7.04 (m, 4H), 3.77 – 3.73 (m, 4H), 3.02 – 2.98 (m, 4H). ^{13}C NMR (101 MHz, $CDCl_3$) δ 162.14, 155.12, 130.37, 130.20, 128.67, 125.23, 120.53, 117.66, 66.23, 46.13. HRMS(ESI) Calc. for $C_{16}H_{18}NO_4S^+$(M + H^+): 320.0951, found: 320.0952.

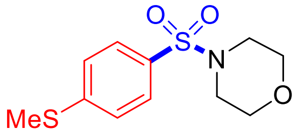

4-((4-(Methylthio)phenyl)sulfonyl)morpholine (**3q**) [1]

40.4 mg, 74% yield. White solid. M.p. 133.4 – 134.3 °C.

^{1}H NMR (400 MHz, $CDCl_3$) δ 7.63 (d, *J* = 8.5 Hz, 2H), 7.33 (d, *J* = 8.5 Hz, 2H), 3.73 (t, *J* = 4.8 Hz, 4H), 3.00 – 2.95 (m, 4H), 2.52 (s, 3H). ^{13}C NMR (101 MHz, $CDCl_3$) δ 146.44, 130.92, 128.25, 125.52, 66.20, 46.10, 14.90.

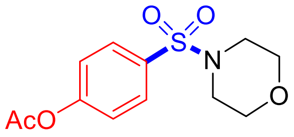

4-(Morpholinosulfonyl)phenyl acetate (**3r**)

31.9 mg, 56% yield. White solid. M.p. 107.0 – 109.1 °C.

^{1}H NMR (400 MHz, $CDCl_3$) δ 7.78 (d, *J* = 8.7 Hz, 2H), 7.30 (d, *J* = 8.7 Hz, 2H), 3.74 (t, *J* = 4.8 Hz, 4H), 3.06 – 2.99 (m, 4H), 2.34 (s, 3H). ^{13}C NMR (101 MHz, $CDCl_3$) δ 168.67, 154.34, 132.63, 129.57, 122.53, 66.21, 46.08, 21.27. HRMS(ESI) Calc. for $C_{12}H_{16}NO_5S^+$ (M + H^+): 286.0744, found: 286.0742.

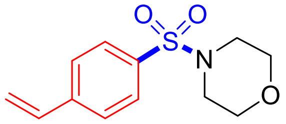

4-((4-Vinylphenyl)sulfonyl)morpholine (**3s**)

15.3 mg, 30% yield. Off-white solid. M.p. 125.5 – 128.5 °C.

^{1}H NMR (400 MHz, $CDCl_3$) δ 7.71 (d, *J* = 8.4 Hz, 2H), 7.56 (d, *J* = 8.4 Hz, 2H), 6.76 (dd, *J* = 17.6, 10.9 Hz, 1H), 5.90 (d, *J* = 17.6 Hz, 1H), 5.46 (d, *J* = 10.9 Hz, 1H), 3.74 (t, *J* = 4.8 Hz, 4H), 3.00 (t, *J* = 4.8 Hz, 4H). ^{13}C NMR (101 MHz, $CDCl_3$) δ 142.39, 135.38, 128.34, 126.88, 117.85, 66.24, 46.12. HRMS(ESI) Calc. for $C_{12}H_{15}NNaO_3S^+$ (M + Na^+): 276.0665, found: 276.0668.

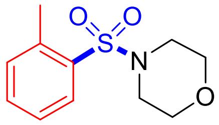

4-(*o*-Tolylsulfonyl)morpholine (**3t**) [1]

31.5 mg, 66% yield. White solid. M.p. 127.0 – 127.4 °C.

^{1}H NMR (400 MHz, $CDCl_3$) δ 7.89 (dd, *J* = 8.2, 1.1 Hz, 1H), 7.48 (td, *J* = 7.5, 1.2 Hz, 1H), 7.35 – 7.31 (m, 2H), 3.72 (t, *J* = 4.8 Hz, 4H), 3.15 (t, *J* = 4.8 Hz, 4H), 2.64 (s, 3H). ^{13}C NMR (101 MHz, $CDCl_3$) δ 138.31, 135.17, 133.22, 133.06, 130.58, 126.31, 66.47, 45.46, 20.97.

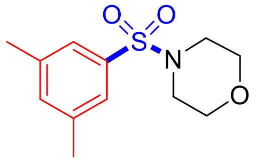

4-((3,5-Dimethylphenyl)sulfonyl)morpholine (**3u**) [6]

36.0 mg, 71% yield. White solid. M.p. 149.2 – 151.4 °C.

^{1}H NMR (400 MHz, $CDCl_3$) δ 7.35 (s, 2H), 7.23 (s, 1H), 3.75 – 3.72 (t, *J* = 4.8 Hz, 4H), 3.01 – 2.97 (t, *J* = 4.8 Hz, 4H), 2.39 (s, 6H). ^{13}C NMR (101 MHz, $CDCl_3$) δ 139.30, 134.92, 125.51, 66.27, 46.17, 21.40.

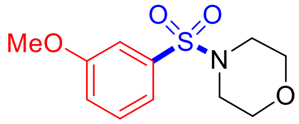

4-((3-Methoxyphenyl)sulfonyl)morpholine (**3v**) [1]

37.7 mg, 74% yield. White solid. M.p. 125.9 – 127.3 °C.

^{1}H NMR (400 MHz, $CDCl_3$) δ 7.45 (t, *J* = 8.0 Hz, 1H), 7.32 (d, *J* = 7.8 Hz, 1H), 7.26 – 7.23 (m, 1H), 7.14 (dd, *J* = 8.2, 2.3 Hz, 1H), 3.86 (s, 3H), 3.74 (t, *J* = 4.8 Hz, 4H), 3.01 (t, *J* = 4.8 Hz, 4H). ^{13}C NMR (101 MHz, $CDCl_3$) δ 160.12, 136.46, 130.33, 120.08, 119.24, 112.93, 66.25, 55.82, 46.15.

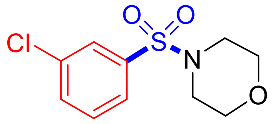

4-((3-Chlorophenyl)sulfonyl)morpholine (**3w**) [1]

34.3 mg, 66% yield. White solid. M.p. 144.2 – 145.2 °C.

^{1}H NMR (400 MHz, $CDCl_3$) δ 7.75 (t, *J* = 1.8 Hz, 1H), 7.65 – 7.59 (m, 2H), 7.50 (t, *J* = 7.9 Hz, 1H), 3.75 (t, *J* = 4.8 Hz, 4H), 3.02 (t, *J* = 4.8 Hz, 4H). ^{13}C NMR (101 MHz, $CDCl_3$) δ 137.23, 135.68, 133.36, 130.59, 127.93, 126.03, 66.20, 46.11.

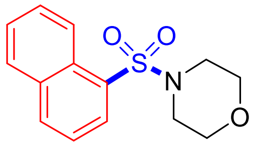

4-(Naphthalen-1-ylsulfonyl)morpholine (**3x**) [1]

36.1 mg, 65% yield. White solid. M.p. 105.3 – 105.9 °C.

^{1}H NMR (400 MHz, $CDCl_3$) δ 8.77 (d, *J* = 8.7 Hz, 1H), 8.21 (dd, *J* = 7.4, 1.1 Hz, 1H), 8.09 (d, *J* = 8.2 Hz, 1H), 7.94 (d, *J* = 7.6 Hz, 1H), 7.68 – 7.54 (m, 3H), 3.68 (t, *J* = 4.8 Hz, 4H), 3.16 (t, *J* = 4.8 Hz, 4H). ^{13}C NMR (101 MHz, $CDCl_3$) δ 134.86, 134.52, 132.10, 130.96, 129.21, 129.10, 128.30, 127.08, 125.25, 124.25, 66.39, 45.75.

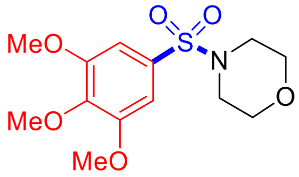

4-((3,4,5-Trimethoxyphenyl)sulfonyl)morpholine (**3y**) [1]

29.2 mg, 46% yield. White solid. M.p. 109.2 – 110.0 °C.

^{1}H NMR (400 MHz, $CDCl_3$) δ 6.94 (s, 2H), 3.90 (s, 9H), 3.74 (t, *J* = 4.8 Hz, 4H), 3.04 – 2.98 (m, 4H). ^{13}C NMR (101 MHz, $CDCl_3$) δ 153.52, 142.06, 129.97, 105.26, 66.22, 61.07, 56.62, 46.10.

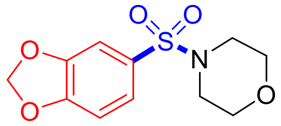

4-(Benzo[*d*][1,3]dioxol-5-ylsulfonyl)morpholine (**3z**) [1]

32.6 mg, 60% yield. White solid. M.p. 144.5 – 146.5 °C.

^{1}H NMR (400 MHz, $CDCl_3$) δ 7.30 (dd, *J* = 8.2, 1.8 Hz, 1H), 7.15 (d, *J* = 1.8 Hz, 1H), 6.91 (d, *J* = 8.2 Hz, 1H), 6.09 (s, 2H), 3.73 (t, *J* = 4.8 Hz, 4H), 3.00 – 2.97 (m, 4H). ^{13}C NMR (101 MHz, $CDCl_3$) δ 151.81, 148.41, 128.50, 123.67, 108.49, 108.10, 102.51, 66.20, 46.14.

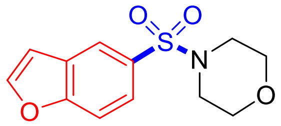

4-(Benzofuran-5-ylsulfonyl)morpholine (**3aa**) [1]

37.6 mg, 71% yield. White solid. M.p. 125.5 – 128.0 °C.

^{1}H NMR (400 MHz, $CDCl_3$) δ 8.06 (d, *J* = 1.2 Hz, 1H), 7.77 (d, *J* = 2.1 Hz, 1H), 7.70 (dd, *J* = 8.7, 1.5 Hz, 1H), 7.64 (d, *J* = 8.7 Hz, 1H), 6.89 (d, *J* = 1.6 Hz, 1H), 3.73 (t, *J* = 4.8 Hz, 4H), 3.03 – 2.98 (m, 4H). ^{13}C NMR (101 MHz, $CDCl_3$) δ 157.04, 147.41, 129.95, 128.09, 124.09, 122.22, 112.21, 107.21, 66.21, 46.20.

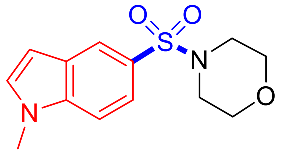

4-((1-Methyl-1*H*-indol-5-yl)sulfonyl)morpholine (**3ab**) [1]

39.8 mg, 71% yield. White solid. M.p. 171.4 – 175.5 °C.

^{1}H NMR (400 MHz, $CDCl_3$) δ 8.08 (d, *J* = 1.3 Hz, 1H), 7.58 (dd, *J* = 8.7, 1.6 Hz, 1H), 7.42 (d, *J* = 8.7 Hz, 1H), 7.20 (d, *J* = 3.1 Hz, 1H), 6.63 (d, *J* = 2.8 Hz, 1H), 3.86 (s, 3H), 3.73 (t, *J* = 4.8 Hz, 4H), 3.02 – 2.97 (m, 4H). ^{13}C NMR (101 MHz, $CDCl_3$) δ 138.68, 131.38, 128.10, 125.60, 122.34, 120.87, 109.66, 102.87, 66.29, 46.30, 33.30.

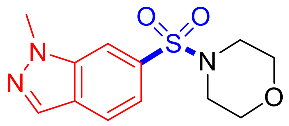

4-((1-Methyl-1*H*-indazol-6-yl)sulfonyl)morpholine (**3ac**)

41.6 mg, 74% yield. White solid. M.p. 156.7 – 160.0 °C.

^{1}H NMR (400 MHz, $CDCl_3$) δ 8.09 (s, 1H), 7.90 – 7.86 (m, 2H), 7.47 (d, *J* = 8.3 Hz, 1H), 4.16 (s, 3H), 3.78 – 3.70 (m, 4H), 3.05 (broad, 4H). ^{13}C NMR (101 MHz, $CDCl_3$) δ 138.96, 133.36, 133.21, 126.35, 122.26, 118.85, 110.09, 66.25, 46.20, 36.15. HRMS(ESI) Calc. for $C_{12}H_{16}N_3O_3S^+$ (M + H$^+$): 282.0907, found: 282.0911.

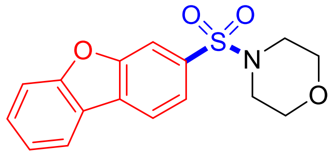

4-(Dibenzo[*b*,*d*]furan-3-ylsulfonyl)morpholine (**3ad**) [7]

28.6 mg, 45% yield. White solid. M.p. 208.7 – 211.3 °C.

^{1}H NMR (400 MHz, $CDCl_3$) δ 8.11 (d, *J* = 8.1 Hz, 1H), 8.03 (d, *J* = 7.7 Hz, 1H), 7.99 (d, *J* = 1.3 Hz, 1H), 7.75 (dd, *J* = 8.1, 1.4 Hz, 1H), 7.65 (d, *J* = 8.3 Hz, 1H), 7.61 – 7.56 (m, 1H), 7.43 (t, *J* = 7.5 Hz, 1H), 3.76 (t, *J* = 4.8 Hz, 4H), 3.06 (t, *J* = 4.8 Hz, 4H). ^{13}C NMR (101

MHz, $CDCl_3$) δ 158.51, 152.41, 133.73, 129.32, 123.76, 122.37, 121.67, 121.24, 112.35, 111.92, 66.28, 46.27.

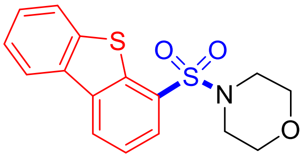

4-(Dibenzo[*b*,*d*]thiophen-4-ylsulfonyl)morpholine (**3ae**)

44.0 mg, 66% yield. White solid. M.p. 149.2 – 150.4 °C.

^{1}H NMR (400 MHz, $CDCl_3$) δ 8.38 (dd, *J* = 7.9, 0.9 Hz, 1H), 8.21 – 8.16 (m, 1H), 7.88 (dd, *J* = 6.4, 1.3 Hz, 2H), 7.61 (t, *J* = 7.7 Hz, 1H), 7.56 – 7.49 (m, 2H), 3.71 (t, *J* = 4.8 Hz, 4H), 3.16 (t, *J* = 4.8 Hz, 4H). ^{13}C NMR (101 MHz, $CDCl_3$) δ 140.32, 138.32, 138.15, 134.05, 130.36, 128.02, 127.65, 125.98, 125.08, 124.65, 122.61, 121.92, 66.21, 46.23. HRMS(ESI) Calc. for $C_{16}H_{16}NO_3S_2^+$ (M + H$^+$): 334.0566, found: 334.0566.

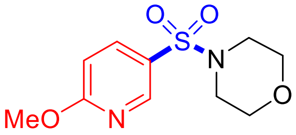

4-((6-Methoxypyridin-3-yl)sulfonyl)morpholine (**3af**) [1]

30.2 mg, 58% yield. White solid. M.p. 156.0 – 157.5 °C.

^{1}H NMR (400 MHz, $CDCl_3$) δ 8.56 (d, *J* = 2.2 Hz, 1H), 7.86 (dd, *J* = 8.8, 2.5 Hz, 1H), 6.86 (d, *J* = 8.7 Hz, 1H), 4.01 (s, 3H), 3.78 – 3.73 (m, 4H), 3.01 (t, *J* = 4.8 Hz, 4H). ^{13}C NMR (101 MHz, $CDCl_3$) δ 166.84, 148.13, 137.93, 124.67, 111.60, 66.13, 54.47, 45.99.

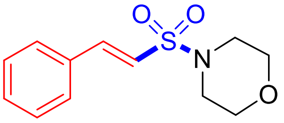

(*E*)-4-(Styrylsulfonyl)morpholine (**3ag**) [8]

21.3 mg, 42% yield. Yellow solid. M.p. 158.8 – 160.8 °C.

^{1}H NMR (400 MHz, $CDCl_3$) δ 7.51 (dd, *J* = 4.9, 1.8 Hz, 2H), 7.48 (d, *J* = 11.0 Hz, 1H), 7.45 – 7.40 (m, 3H), 6.67 (d, *J* = 15.5 Hz, 1H), 3.80 – 3.76 (m, 4H), 3.19 (t, *J* = 4.8 Hz, 4H). ^{13}C NMR (101 MHz, $CDCl_3$) δ 144.40, 132.54, 131.28, 129.29, 128.45, 120.69, 66.42, 45.85.

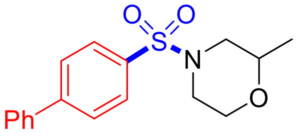

4-([1,1'-Biphenyl]-4-ylsulfonyl)-2-methylmorpholine (**3ah**)

25.6 mg, 41% yield. White crystalline solid. M.p. 179.6 – 182.6 °C.

^{1}H NMR (400 MHz, $CDCl_3$) δ 7.82 (d, *J* = 8.5 Hz, 2H), 7.75 (d, *J* = 8.5 Hz, 2H), 7.61 (d, *J* = 7.1 Hz, 2H), 7.49 (t, *J* = 7.3 Hz, 2H), 7.43 (t, *J* = 7.3 Hz, 1H), 3.91 (dd, *J* = 11.7, 2.1 Hz, 1H), 3.74 – 3.65 (m, 2H), 3.59 (t, *J* = 12.1 Hz, 2H), 2.45 (td, *J* = 11.5, 3.3 Hz, 1H), 2.10 (t, *J* = 10.6 Hz, 1H), 1.15 (d, *J* = 6.3 Hz, 3H). HRMS(ESI) Calc. for $C_{17}H_{20}NO_3S^+$ (M + H^+): 318.1158, found: 318.1162.

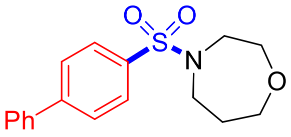

4-([1,1'-Biphenyl]-4-ylsulfonyl)-1,4-oxazepane (**3ai**)

39.9 mg, 63% yield. White crystalline solid. M.p. 127.8 – 130.1 °C.

^{1}H NMR (400 MHz, $CDCl_3$) δ 7.85 (d, *J* = 8.3 Hz, 2H), 7.72 (d, *J* = 8.3 Hz, 2H), 7.60 (d, *J* = 7.2 Hz, 2H), 7.48 (t, *J* = 7.4 Hz, 2H), 7.42 (t, *J* = 7.2 Hz, 1H), 3.85 – 3.74 (m, 4H), 3.48 – 3.37 (m, 4H), 2.02 – 1.93 (m, 2H). ^{13}C NMR (101 MHz, $CDCl_3$) δ 145.64, 139.41, 137.69, 129.20, 128.62, 127.90, 127.63, 127.44, 71.30, 69.63, 51.57, 47.04, 30.49. HRMS(ESI) Calc. for $C_{17}H_{20}NO_3S^+$ (M + H^+): 318.1158, found: 318.1158.

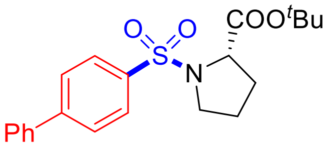

tert-Butyl ([1,1'-biphenyl]-4-ylsulfonyl)-*L*-prolinate (**3aj**)

54.8 mg, 71% yield. Off-white solid. M.p. 83.0 – 85.0 °C.

^{1}H NMR (400 MHz, $CDCl_3$) δ 7.94 (d, *J* = 8.4 Hz, 2H), 7.71 (d, *J* = 8.4 Hz, 2H), 7.60 (d, *J* = 7.2 Hz, 2H), 7.47 (t, *J* = 7.4 Hz, 2H), 7.41 (t, *J* = 7.3 Hz, 1H), 4.25 (dd, *J* = 8.3, 3.4 Hz, 1H), 3.53 – 3.48 (m, 1H), 3.42 – 3.34 (m, 1H), 2.08 – 1.95 (m, 3H), 1.84 – 1.77 (m, 1H), 1.45 (s, 9H). ^{13}C NMR (101 MHz, $CDCl_3$) δ 171.40, 145.65, 139.54, 137.56, 129.15, 128.53, 128.11, 127.71, 127.44, 81.81, 61.33, 48.48, 31.15, 28.04, 24.72. HRMS(ESI) Calc. for $C_{21}H_{26}NO_4S^+$ (M + H^+): 388.1577, found: 388.1572.

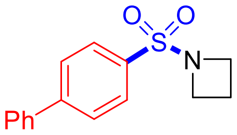

1-([1,1'-Biphenyl]-4-ylsulfonyl)azetidine (**3ak**)

26.8 mg, 49% yield. White solid. M.p. 115.8 – 118.0 °C.

^{1}H NMR (400 MHz, $CDCl_3$) δ 7.91 (d, *J* = 8.5 Hz, 2H), 7.78 (d, *J* = 8.5 Hz, 2H), 7.66 – 7.61 (m, 2H), 7.50 (t, *J* = 7.3 Hz, 2H), 7.43 (t, *J* = 7.3 Hz, 1H), 3.83 (t, *J* = 7.6 Hz, 4H), 2.12 (dd, *J* = 15.3, 7.6 Hz, 2H). ^{13}C NMR (101 MHz, $CDCl_3$) δ 146.14, 139.43, 133.55, 129.24, 129.01, 128.72, 127.84, 127.50, 51.10, 15.48. HRMS(ESI) Calc. for $C_{15}H_{16}NO_2S^+$ (M + H^+): 274.0896, found: 274.0898.

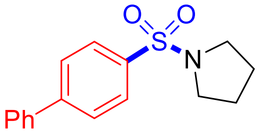

1-([1,1'-Biphenyl]-4-ylsulfonyl)pyrrolidine (**3al**)

26.9 mg, 47% yield. White solid. M.p. 130.5 – 133.0 °C.

^{1}H NMR (400 MHz, $CDCl_3$) δ 7.92 (d, *J* = 8.4 Hz, 2H), 7.76 (d, *J* = 8.4 Hz, 2H), 7.64 (d, *J* = 7.2 Hz, 2H), 7.51 (t, *J* = 7.4 Hz, 2H), 7.44 (t, *J* = 7.3 Hz, 1H), 3.32 (t, *J* = 6.7 Hz, 4H), 1.85 – 1.78 (m, 4H). ^{13}C NMR (101 MHz, $CDCl_3$) δ 145.59, 139.52, 135.85, 129.20, 128.59, 128.18, 127.75, 127.45, 48.10, 25.41. HRMSESI) Calc. for $C_{16}H_{18}NO_2S^+$ (M + H^+): 288.1053, found: 288.1062.

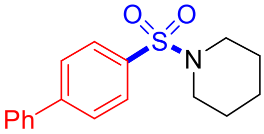

1-([1,1'-Biphenyl]-4-ylsulfonyl)piperidine (**3am**)

25.4 mg, 42% yield. White solid. M.p. 176.9 – 178.0 °C.

^{1}H NMR (400 MHz, $CDCl_3$) δ 7.82 (d, *J* = 8.4 Hz, 2H), 7.73 (d, *J* = 8.4 Hz, 2H), 7.63 – 7.59 (m, 2H), 7.48 (t, *J* = 7.4 Hz, 2H), 7.42 (t, *J* = 7.3 Hz, 1H), 3.07 – 3.01 (m, 4H), 1.67 (dt, *J* = 11.3, 5.8 Hz, 4H), 1.44 (t, *J* = 5.9 Hz, 2H). ^{13}C NMR (101 MHz, $CDCl_3$) δ 145.59, 139.51, 135.21, 129.20, 128.60, 128.34, 127.68, 127.46, 47.11, 25.36, 23.68. HRMS(ESI) Calc. for $C_{17}H_{20}NO_2S^+$ (M + H^+): 302.1209, found: 302.1208.

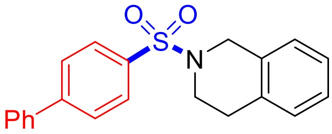

2-([1,1'-Biphenyl]-4-ylsulfonyl)-1,2,3,4-tetrahydroisoquinoline (**3an**)

36.9 mg, 53% yield. Off-white solid. M.p. 133.9 – 135.6 °C.

^{1}H NMR (400 MHz, $CDCl_3$) δ 7.91 (d, *J* = 8.5 Hz, 2H), 7.73 (d, *J* = 8.5 Hz, 2H), 7.59 (d, *J* = 7.1 Hz, 2H), 7.48 (t, *J* = 7.3 Hz, 2H), 7.42 (t, *J* = 7.3 Hz, 1H), 7.17 – 7.12 (m, 2H), 7.11 –

7.03 (m, 2H), 4.33 (s, 2H), 3.43 (t, *J* = 5.9 Hz, 2H), 2.95 (t, *J* = 5.9 Hz, 2H). ^{13}C NMR (101 MHz, $CDCl_3$) δ 145.92, 139.40, 135.20, 133.21, 131.73, 129.20, 128.97, 128.65, 128.34, 127.83, 127.45, 126.92, 126.51, 47.69, 43.89, 28.99. HRMS(ESI) Calc. for $C_{21}H_{20}NO_2S^+$ (M + H^+): 350.1209, found: 350.1209.

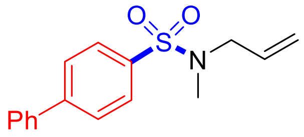

N-Allyl-*N*-methyl-[1,1'-biphenyl]-4-sulfonamide (**3ao**)

21.2 mg, 37% yield. White solid. M.p. 86.8 – 88.4 °C.

^{1}H NMR (400 MHz, $CDCl_3$) δ 7.88 (d, *J* = 8.5 Hz, 2H), 7.77 (d, *J* = 8.5 Hz, 2H), 7.62 (d, *J* = 7.0 Hz, 2H), 7.50 (t, *J* = 7.3 Hz, 2H), 7.47 – 7.41 (m, 1H), 4.42 (tt, *J* = 6.6, 4.8 Hz, 1H), 3.93 (qd, *J* = 12.1, 4.8 Hz, 2H), 3.52 (dd, *J* = 14.6, 7.2 Hz, 1H), 3.28 (dd, *J* = 14.6, 6.4 Hz, 1H), 2.94 (s, 3H). ^{13}C NMR (101 MHz, $CDCl_3$) δ 146.21, 139.25, 135.40, 129.25, 128.77, 128.13, 128.06, 127.47, 58.71, 54.22, 46.63, 37.80. HRMS(EI) Calc. for $C_{16}H_{17}NO_2S^+$ (M+): 287.0975, found: 287.0986.

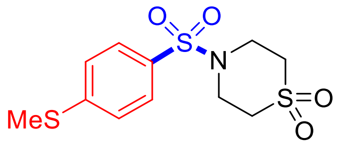

4-((4-(Methylthio)phenyl)sulfonyl)thiomorpholine 1,1-dioxide (**3ap**)

29.5 mg, 46% yield. White solid.

^{1}H NMR (400 MHz, $CDCl_3$) δ 7.65 (d, *J* = 8.6 Hz, 2H), 7.34 (d, *J* = 8.6 Hz, 2H), 3.65 (broad, 4H), 3.17 – 3.11 (m, 4H), 2.54 (s, 3H). ^{13}C NMR (101 MHz, $CDCl_3$) δ 147.51, 132.31, 127.67, 125.84, 51.50, 44.96, 14.88. HRMS(ESI) Calc. for $C_{11}H_{15}NNaO_4S_3^+$ (M + Na^+): 344.0055, found: 344.0060.

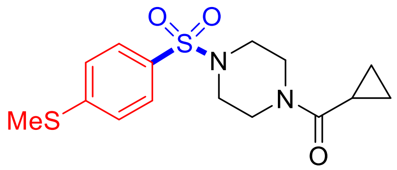

Cyclopropyl(4-((4-(methylthio)phenyl)sulfonyl)piperazin-1-yl)methanone (**3aq**)

35.3 mg, 52% yield. White solid. M.p. 78 – 80 °C. (Measured manually)

^{1}H NMR (400 MHz, $CDCl_3$) δ 7.62 (d, *J* = 8.6 Hz, 2H), 7.32 (d, *J* = 8.6 Hz, 2H), 3.74 (broad, 4H), 3.01 (broad, 4H), 2.53 (s, 3H), 1.66 – 1.59 (m, 1H), 0.96 – 0.90 (m, 2H), 0.77 – 0.70 (m, 2H). ^{13}C NMR (101 MHz, $CDCl_3$) δ 172.13, 146.66, 131.08, 128.14, 125.59, 46.36,

46.08, 45.05, 41.52, 14.90, 11.02, 7.82. HRMS(ESI) Calc. for $C_{15}H_{21}N_2O_3S_2^+$ (M + H$^+$): 341.0988, found: 341.0966.

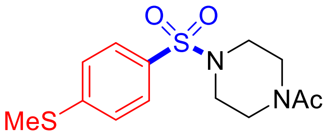

1-(4-((4-(Methylthio)phenyl)sulfonyl)piperazin-1-yl)ethan-1-one (**3ar**)

39.6 mg, 63% yield. Yellow solid.

^{1}H NMR (400 MHz, $CDCl_3$) δ 7.59 (d, *J* = 8.4 Hz, 2H), 7.30 (d, *J* = 8.4 Hz, 2H), 3.66 (t, *J* = 4.8 Hz, 2H), 3.52 (t, *J* = 4.8 Hz, 2H), 3.01 – 2.92 (m, 4H), 2.50 (s, 3H), 2.01 (s, 3H). ^{13}C NMR (101 MHz, $CDCl_3$) δ 168.91, 146.69, 130.78, 128.05, 125.50, 46.16, 45.87, 45.71, 40.73, 21.30, 14.81. HRMS(ESI) Calc. for $C_{13}H_{18}N_2O_3S_2^+$ (M + H$^+$): 315.0832, found: 315.0832.

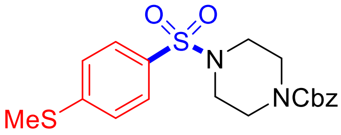

Benzyl 4-((4-(methylthio)phenyl)sulfonyl)piperazine-1-carboxylate (**3as**)

44.7 mg, 55% yield. White solid. M.p. 126.3 – 126.9 ^{o}C.

^{1}H NMR (400 MHz, $CDCl_3$) δ 7.61 (d, *J* = 8.6 Hz, 2H), 7.37 – 7.27 (m, 7H), 5.07 (s, 2H), 3.58 (t, *J* = 5.2 Hz, 1H), 2.97 (broad, 4H), 2.52 (s, 3H). ^{13}C NMR (101 MHz, $CDCl_3$) δ 154.89, 146.56, 136.29, 131.08, 128.66, 128.35, 128.17, 128.12, 125.54, 67.64, 45.91, 43.31, 14.87. HRMS(ESI) Calc. for $C_{19}H_{22}N_2NaO_4S_2^+$ (M + Na$^+$): 429.0913, found: 429.0921.

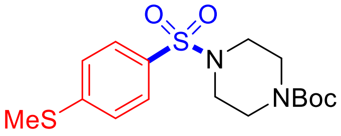

tert-Butyl 4-((4-(methylthio)phenyl)sulfonyl)piperazine-1-carboxylate (**3at**)

39.4 mg, 53% yield. White crystalline solid. M.p. 158.9 – 159.6 ^{o}C.

^{1}H NMR (400 MHz, $CDCl_3$) δ 7.62 (d, *J* = 8.5 Hz, 2H), 7.31 (d, *J* = 8.5 Hz, 2H), 3.51 – 3.46 (m, 4H), 3.01 – 2.91 (m, 4H), 2.52 (s, 3H), 1.40 (s, 9H). ^{13}C NMR (101 MHz, $CDCl_3$) δ 154.27, 146.43, 131.35, 128.16, 125.56, 80.52, 46.00, 43.25, 28.44, 14.90. HRMS(ESI) Calc. for $C_{16}H_{24}N_2NaO_4S_2^+$ (M + Na$^+$): 395.1070, found: 395.1084.

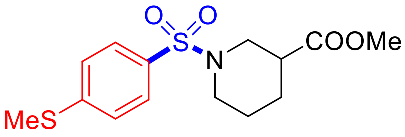

Methyl 1-((4-(methylthio)phenyl)sulfonyl)piperidine-3-carboxylate (**3au**)

35.5 mg, 54% yield. Light yellow oil.

^{1}H NMR (400 MHz, CDCl$_3$) δ 7.64 (d, *J* = 8.5 Hz, 2H), 7.31 (d, *J* = 8.5 Hz, 2H), 3.82 (d, *J* = 11.5 Hz, 1H), 3.67 (s, 3H), 3.60 (d, *J* = 11.5 Hz, 1H), 2.62 (tt, *J* = 10.8, 3.7 Hz, 1H), 2.52 (s, 3H), 2.47 (d, *J* = 10.7 Hz, 1H), 2.33 (td, *J* = 11.4, 2.7 Hz, 1H), 1.98 (dd, *J* = 13.2, 3.4 Hz, 1H), 1.81 – 1.75 (m, 1H), 1.70 – 1.55 (m, 2H), 1.42 – 1.32 (m, 2H). ^{13}C NMR (101 MHz, CDCl$_3$) δ 173.24, 146.01, 131.85, 128.03, 125.46, 52.06, 47.80, 46.37, 41.09, 29.81, 26.57, 24.08, 14.87. HRMS(ESI) Calc. for $C_{14}H_{20}NO_4S_2^+$ (M + H$^+$): 330.0828, found: 330.0828.

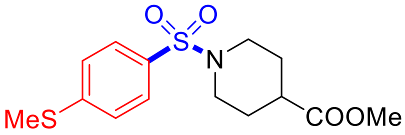

Methyl 1-((4-(methylthio)phenyl)sulfonyl)piperidine-4-carboxylate (**3av**)

33.4 mg, 51% yield. Off-white solid. M.p. 102.6 – 105.5 °C.

^{1}H NMR (400 MHz, CDCl$_3$) δ 7.62 (d, *J* = 8.5 Hz, 2H), 7.30 (d, *J* = 8.5 Hz, 2H), 3.65 (s, 3H), 3.63 – 3.57 (m, 2H), 2.52 (s, 3H), 2.46 (td, *J* = 11.7, 2.5 Hz, 2H), 2.29 – 2.22 (m, 1H), 1.96 (dd, *J* = 13.6, 3.3 Hz, 2H), 1.85 – 1.76 (m, 2H). ^{13}C NMR (101 MHz, CDCl$_3$) δ 174.36, 145.96, 131.86, 128.04, 125.43, 52.01, 45.50, 39.97, 29.81, 27.52, 14.87. HRMS(ESI) Calc. for $C_{14}H_{20}NO_4S_2^+$ (M + H$^+$): 330.0828, found: 330.0838.

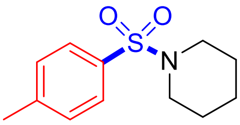

1-Tosylpiperidine (**3aw**)

24.7 mg, 52% yield. White solid.

^{1}H NMR (400 MHz, CDCl$_3$) δ 7.63 (d, *J* = 8.0 Hz, 2H), 7.31 (d, *J* = 7.9 Hz, 2H), 2.98 – 2.93 (m, 4H), 2.43 (s, 3H), 1.65 – 1.60 (m, 4H), 1.43 – 1.37 (m, 2H). ^{13}C NMR (101 MHz, CDCl$_3$) δ 143.42, 133.35, 129.66, 127.84, 47.06, 25.28, 23.64, 21.65.

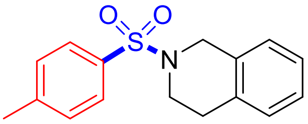

2-Tosyl-1,2,3,4-tetrahydroisoquinoline (**3ax**)

29.5 mg, 52% yield. Off-white solid.

^{1}H NMR (400 MHz, $CDCl_3$) δ 7.73 (d, *J* = 8.0 Hz, 2H), 7.32 (d, *J* = 7.9 Hz, 2H), 7.16 – 7.11 (m, 2H), 7.09 – 7.06 (m, 1H), 7.05 – 7.00 (m, 1H), 4.24 (s, 2H), 3.35 (t, *J* = 5.9 Hz, 2H), 2.93 (t, *J* = 5.8 Hz, 2H), 2.42 (s, 3H). ^{13}C NMR (101 MHz, $CDCl_3$) δ 143.81, 133.30, 133.17, 131.73, 129.82, 128.92, 127.86, 126.84, 126.47, 126.44, 47.65, 43.84, 28.98, 21.64.

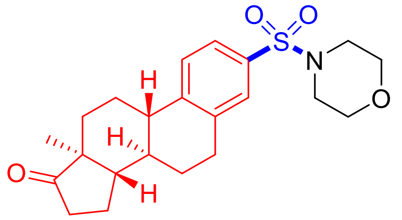

(8*R*,9*S*,13*S*,14*S*)-13-Methyl-3-(morpholinosulfonyl)-6,7,8,9,11,12,13,14,15,16-decahydro-17*H*-cyclopenta[*a*]phenanthren-17-one (**4a**)

61.7 mg, 73% yield. White sticky solid.

^{1}H NMR (400 MHz, $CDCl_3$) δ 7.51 – 7.43 (m, 3H), 3.73 (t, *J* = 4.8 Hz, 4H), 3.00 – 2.95 (m, 6H), 2.52 (dd, *J* = 18.7, 8.6 Hz, 1H), 2.47 – 2.41 (m, 1H), 2.35 (td, *J* = 10.9, 3.9 Hz, 1H), 2.20 – 1.98 (m, 4H), 1.67 – 1.46 (m, 6H), 0.92 (s, 3H). ^{13}C NMR (101 MHz, $CDCl_3$) δ 220.47, 145.64, 138.03, 132.22, 128.32, 126.30, 125.32, 66.23, 50.55, 47.95, 46.12, 44.63, 37.73, 35.89, 31.58, 29.48, 26.18, 25.64, 21.68, 13.91. HRMS(ESI) Calc. for $C_{22}H_{30}NO_4S^+$ (M + H^+): 404.1890, found: 404.1894.

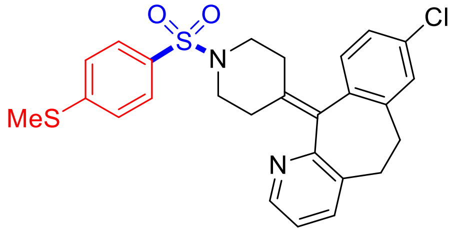

8-Chloro-11-(1-((4-(methylthio)phenyl)sulfonyl)piperidin-4-ylidene)-6,11-dihydro-5*H*-benzo[5,6]cyclohepta[1,2-*b*]pyridine (**4b**)

48.7 mg, 49% yield. Light yellow oil.

^{1}H NMR (400 MHz, $CDCl_3$) δ 8.36 (dd, *J* = 4.7, 1.5 Hz, 1H), 7.65 – 7.60 (m, 2H), 7.41 (dd, *J* = 7.7, 1.3 Hz, 1H), 7.32 – 7.29 (m, 2H), 7.14 – 7.06 (m, 3H), 7.01 (d, *J* = 8.1 Hz, 1H), 3.33 – 3.19 (m, 4H), 2.95 – 2.88 (m, 2H), 2.82 – 2.71 (m, 2H), 2.64 – 2.58 (m, 1H), 2.54 (s, 3H), 2.52 – 2.45 (m, 1H), 2.39 – 2.31 (m, 2H). ^{13}C NMR (101 MHz, $CDCl_3$) δ 156.72, 146.78, 145.93, 139.63, 137.78, 137.44, 135.90, 134.93, 133.49, 133.23, 132.23,

130.50, 129.14, 128.05, 126.33, 125.56, 122.53, 47.47, 31.71, 31.56, 30.30, 30.03, 14.96. HRMS(ESI) Calc. for $C_{26}H_{26}ClN_2O_2S_2^+$ (M + H^+): 497.1119, found: 497.1101.

References:

[1] This compound is known, and NMR data is in agreement with reported in *J. Am. Chem. Soc.* **2018**, *140*, 8781.

[2] This compound is known, and NMR data is in agreement with reported in *Green Chem.* **2015**, *17*, 1395.

[3] This compound is known, and NMR data is in agreement with reported in *Org. Lett.* **2018**, *20*, 1167.

[4] This compound is known, and NMR data is in agreement with reported in *Synlett* **2016**, *27*, 101.

[5] This compound is known, and NMR data is in agreement with reported in *Tetrahedron Lett.* **2011**, *52*, 820.

[6] This compound is known, and NMR data is in agreement with reported in *Angew. Chem. Int. Ed.* **2014**, *53*, 10204.

[7] This compound is known, and NMR data is in agreement with reported in *J. Am. Chem. Soc.* **2011**, *133*, 9250.

[8] This compound is known, and NMR data is in agreement with reported in *J. Am. Chem. Soc.* **2012**, 134, 9086.

5. ^{1}H, ^{13}C and ^{19}F NMR spectra of compounds 3 and 4.

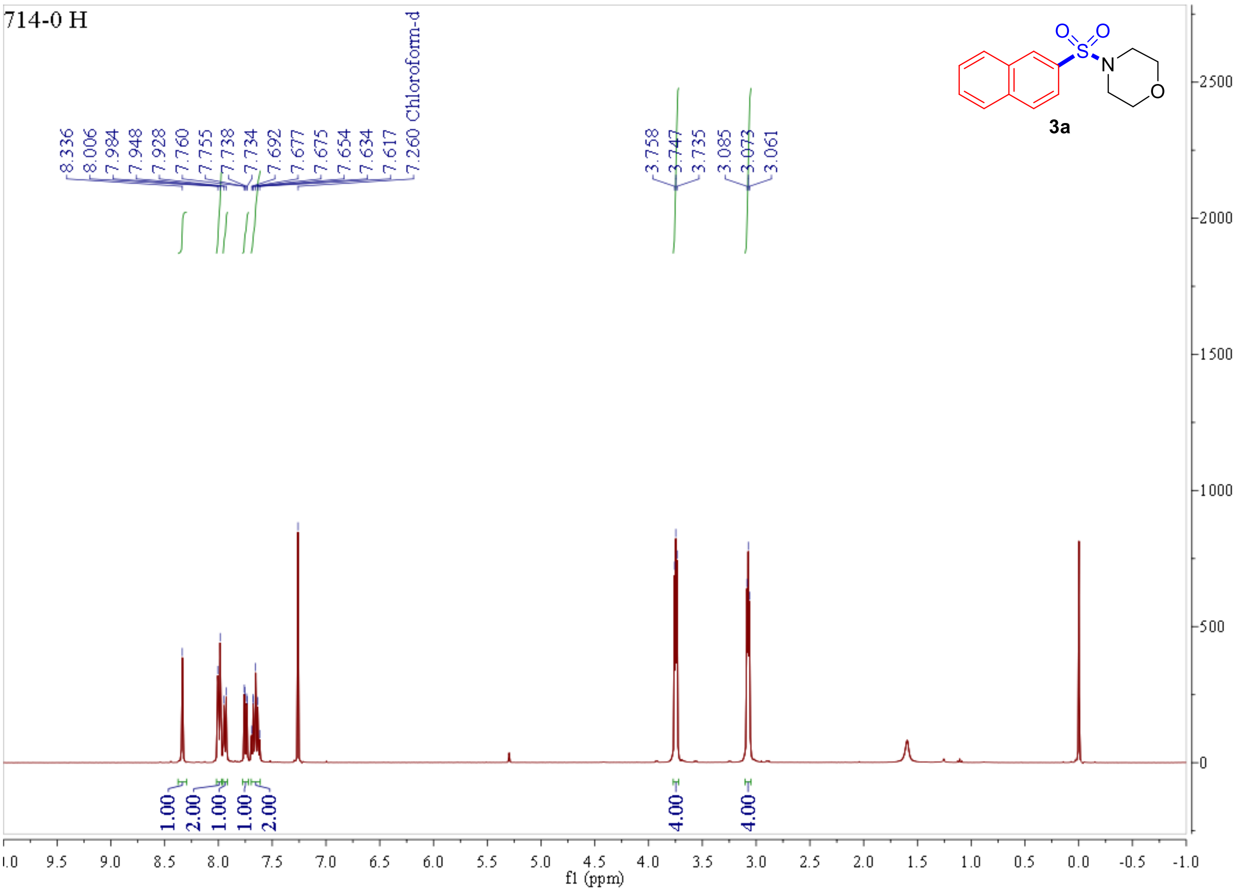

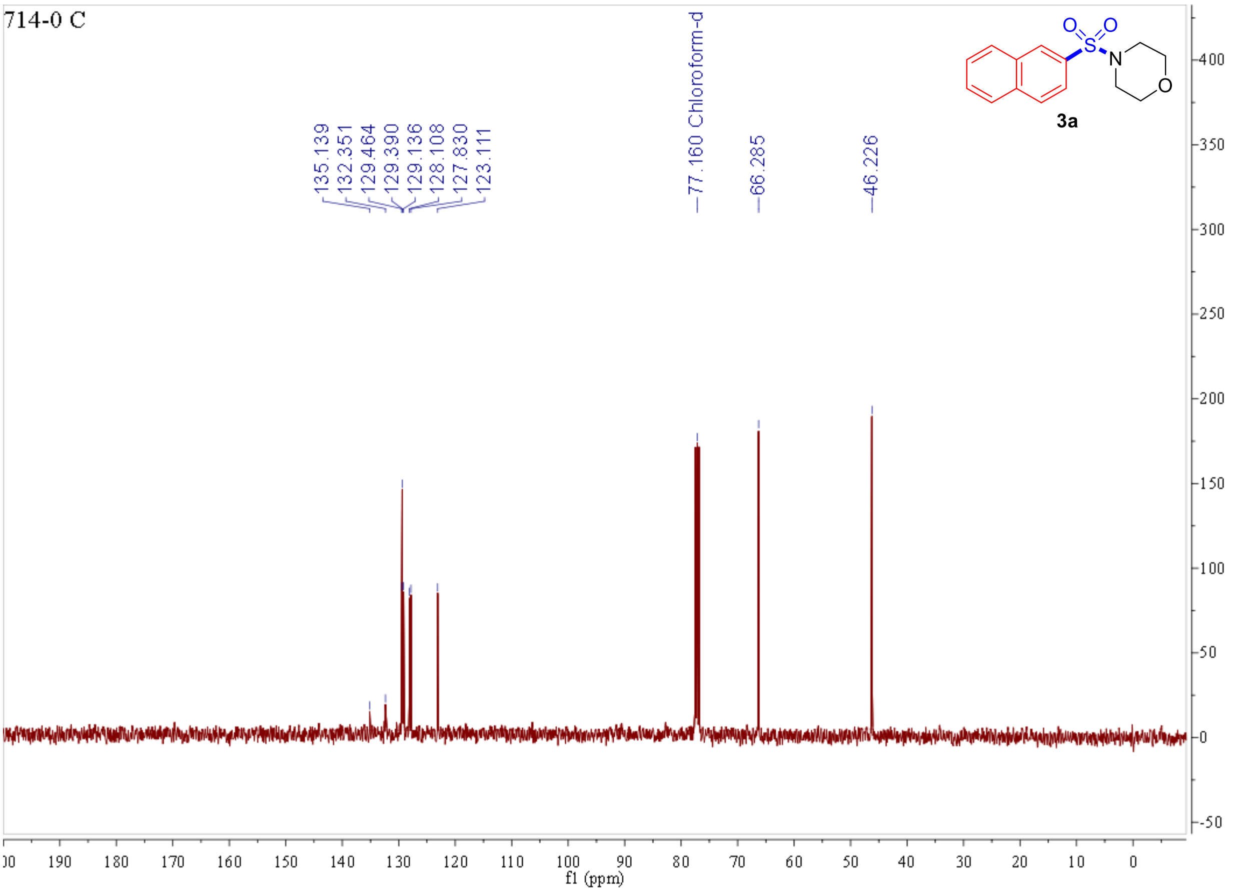

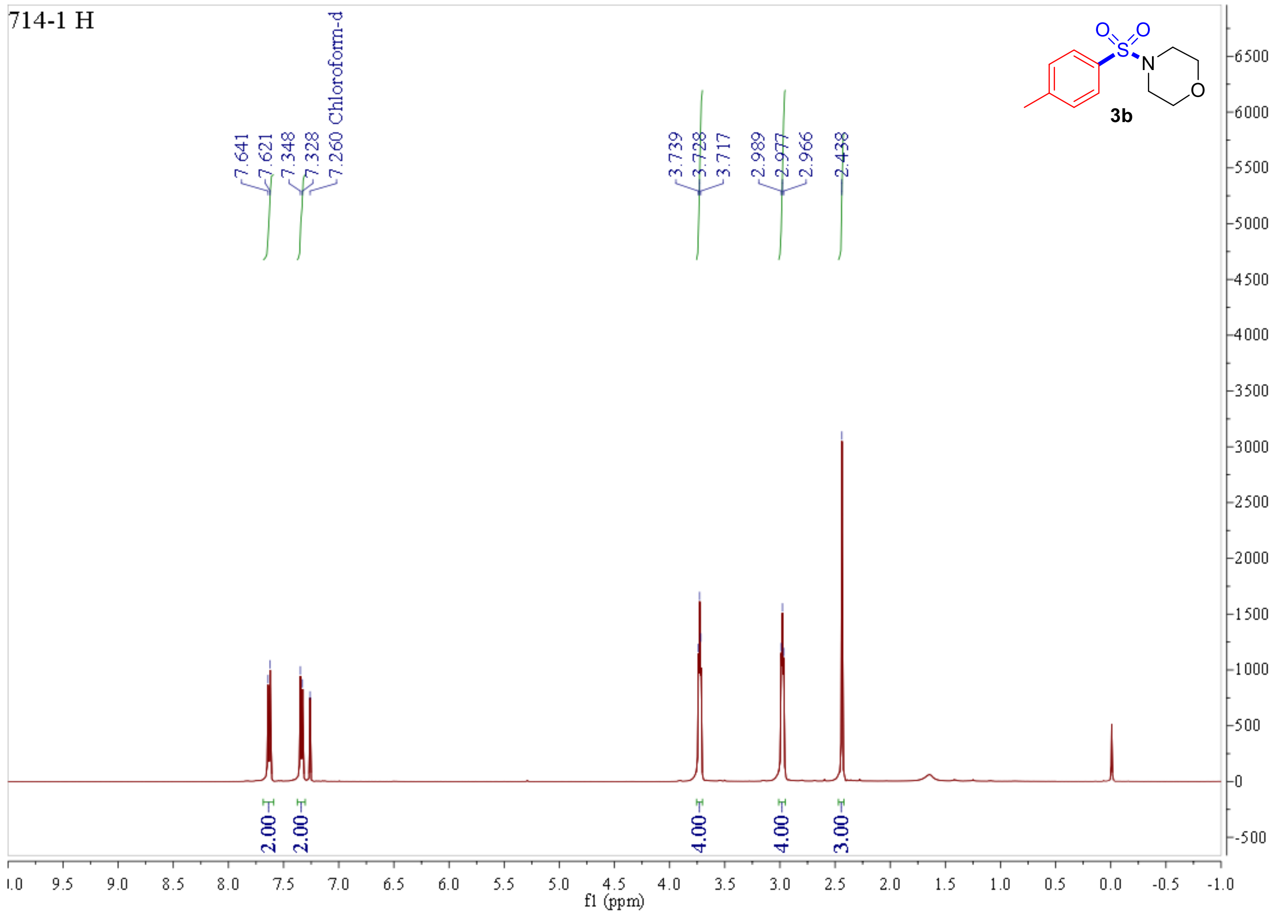
714-1 H
7.641
7.621
7.348
7.328
7.260 Chloroform-d
3.739
3.728
3.717
2.989
2.977
2.966
2.438
3b
2.00
2.00
4.00
4.00
3.00
f1 (ppm)

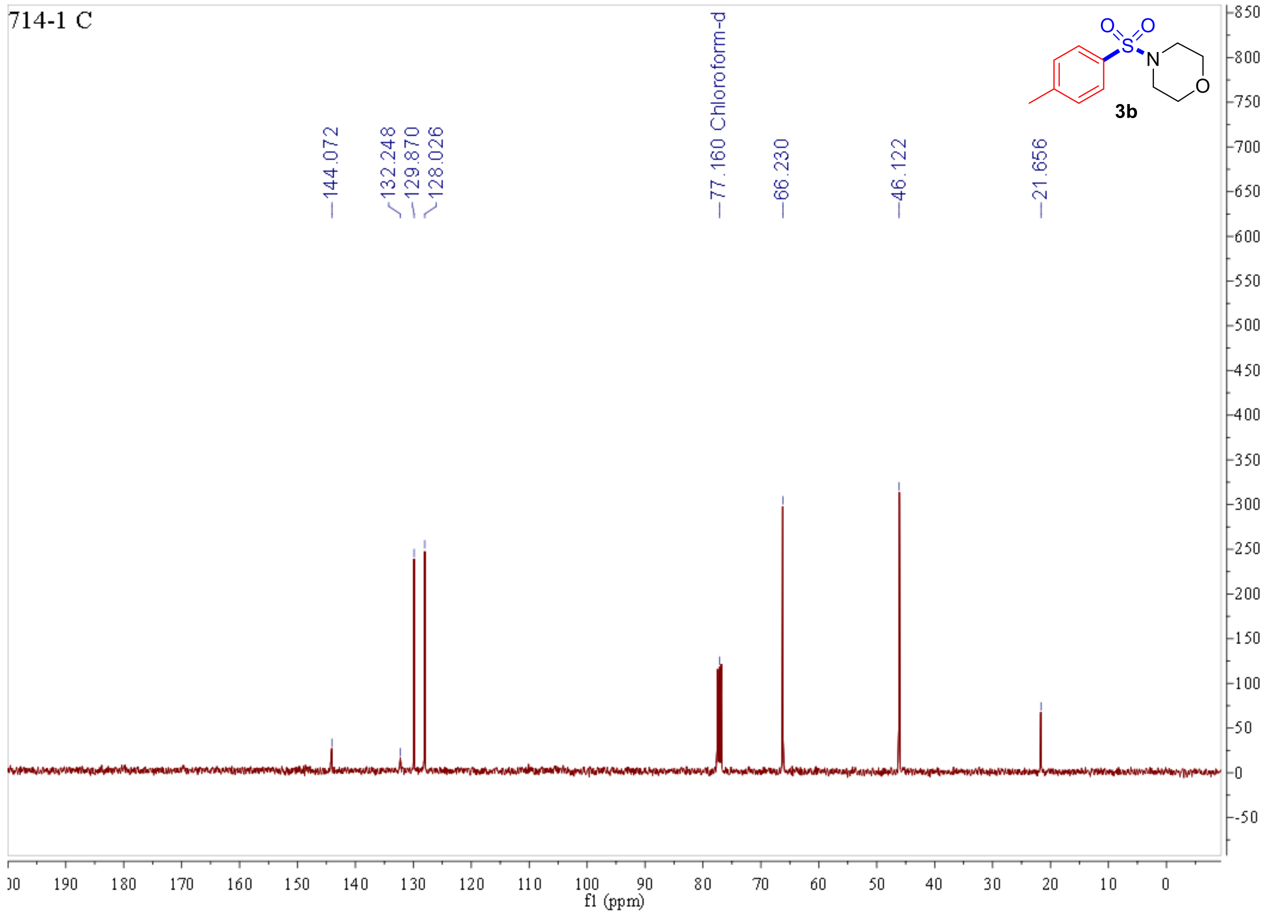
714-1 C
144.072
132.248
129.870
128.026
77.160 Chloroform-d
66.230
46.122
21.656
3b
f1 (ppm)

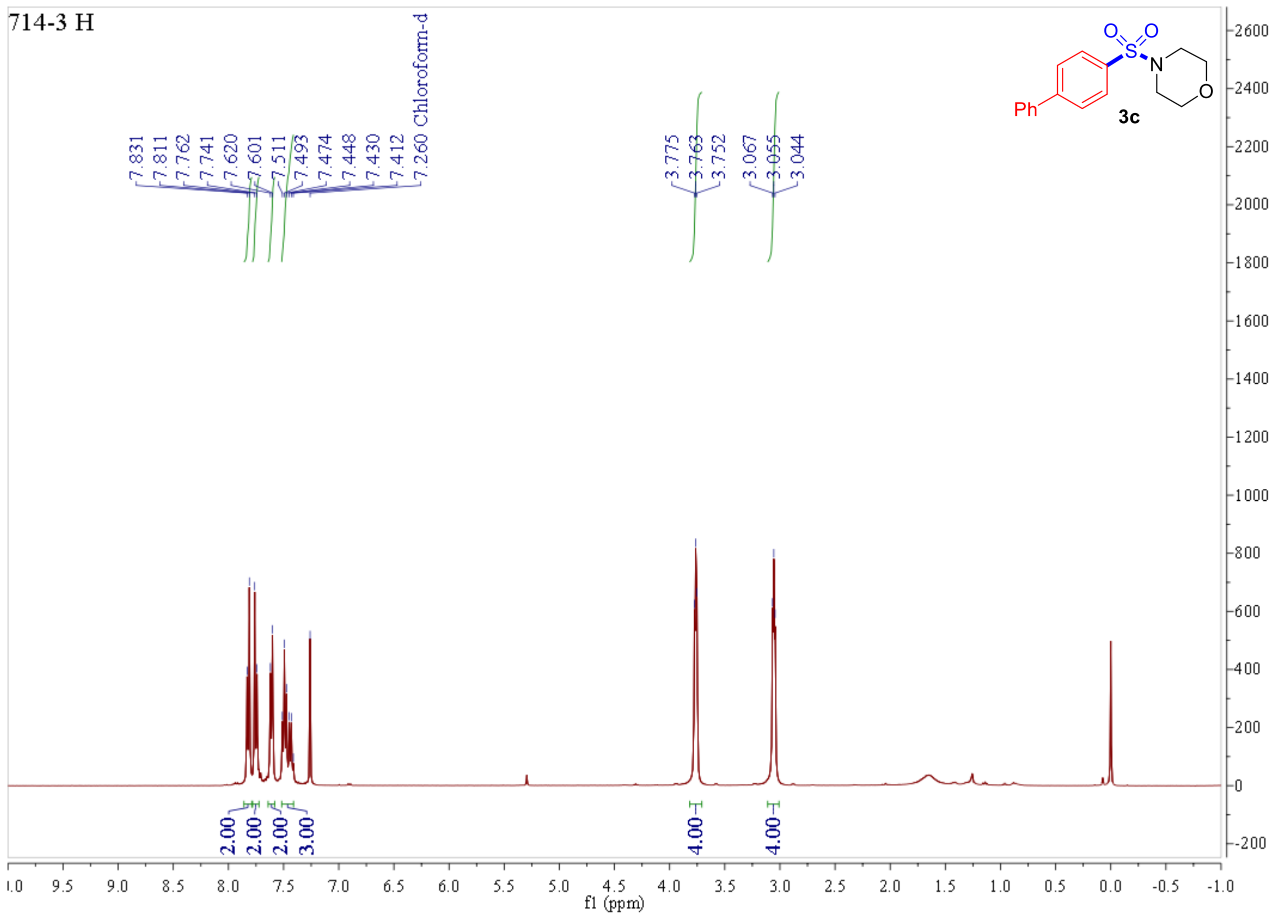
714-3 H
7.831
7.811
7.762
7.741
7.620
7.601
7.511
7.493
7.474
7.448
7.430
7.412
7.260 Chloroform-d
3.775
3.763
3.752
3.067
3.055
3.044
Ph
3c
2.00
2.00
2.00
3.00
4.00
4.00
f1 (ppm)

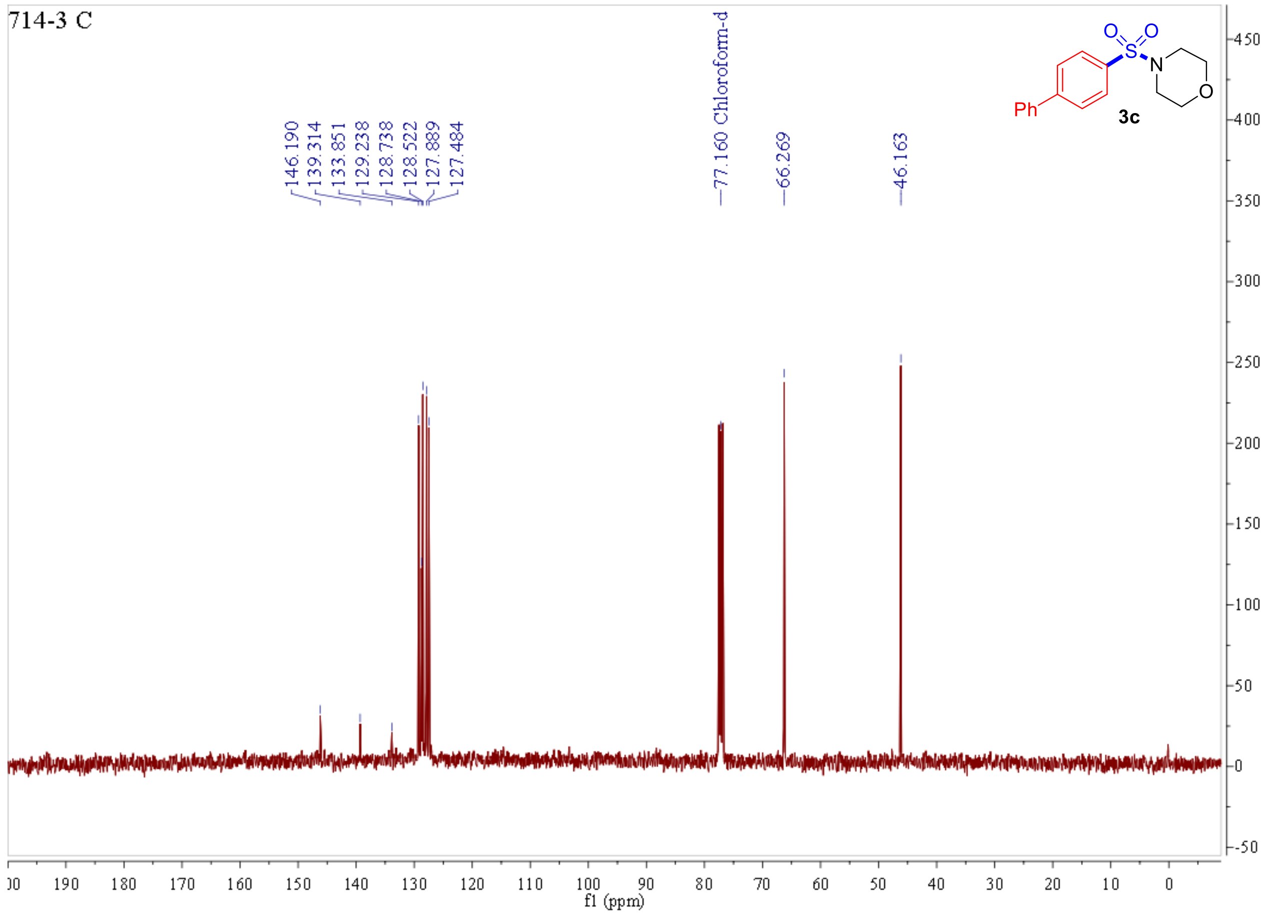
714-3 C
146.190
139.314
133.851
129.238
128.738
128.522
127.889
127.484
77.160 Chloroform-d
66.269
46.163
Ph
3c
f1 (ppm)

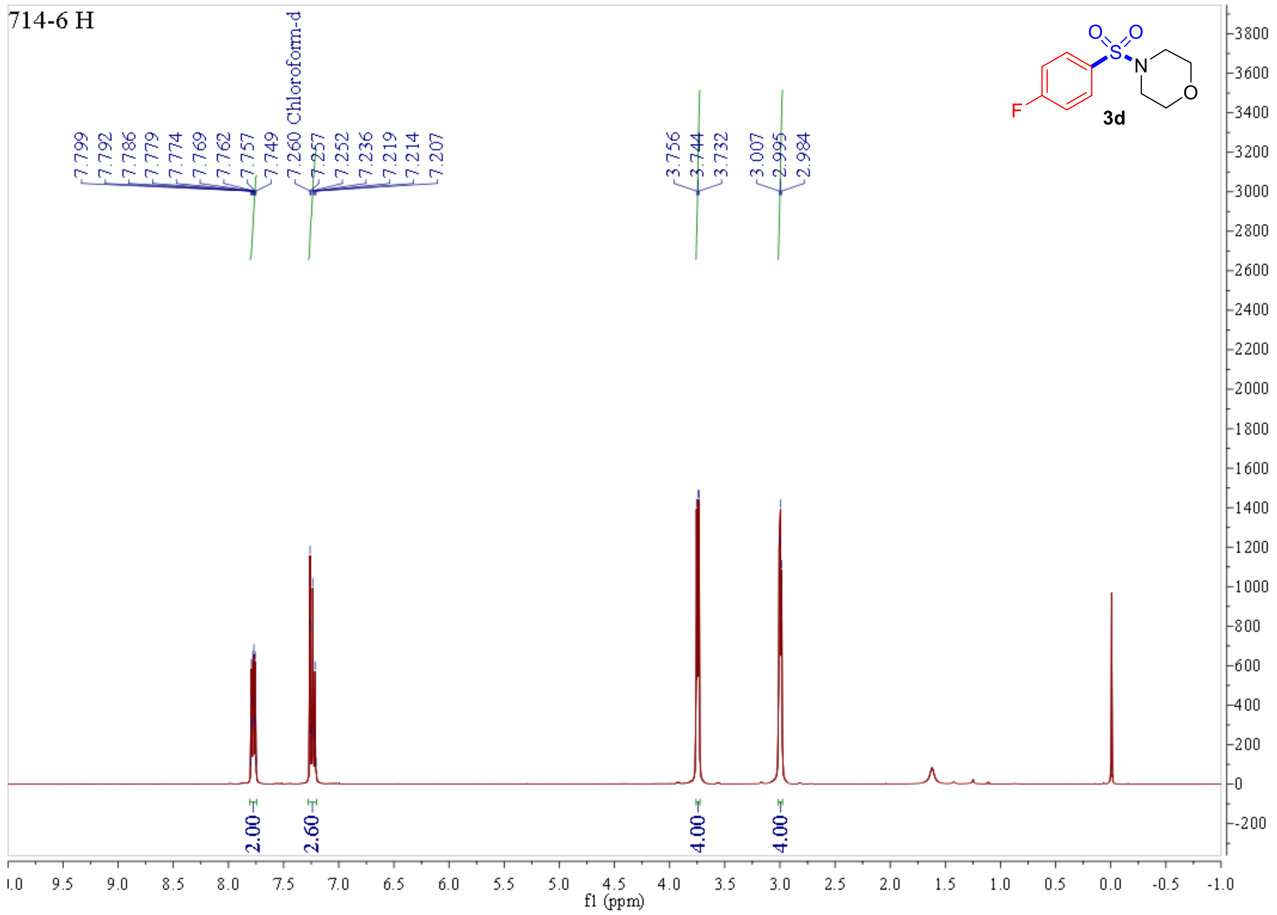
714-6 H
7.799
7.792
7.786
7.779
7.774
7.769
7.762
7.757
7.749
7.260 Chloroform-d
7.257
7.252
7.236
7.219
7.214
7.207
3.756
3.744
3.732
3.007
2.995
2.984
F
O
O
S
N
O
3d
2.00
2.60
4.00
4.00
f1 (ppm)

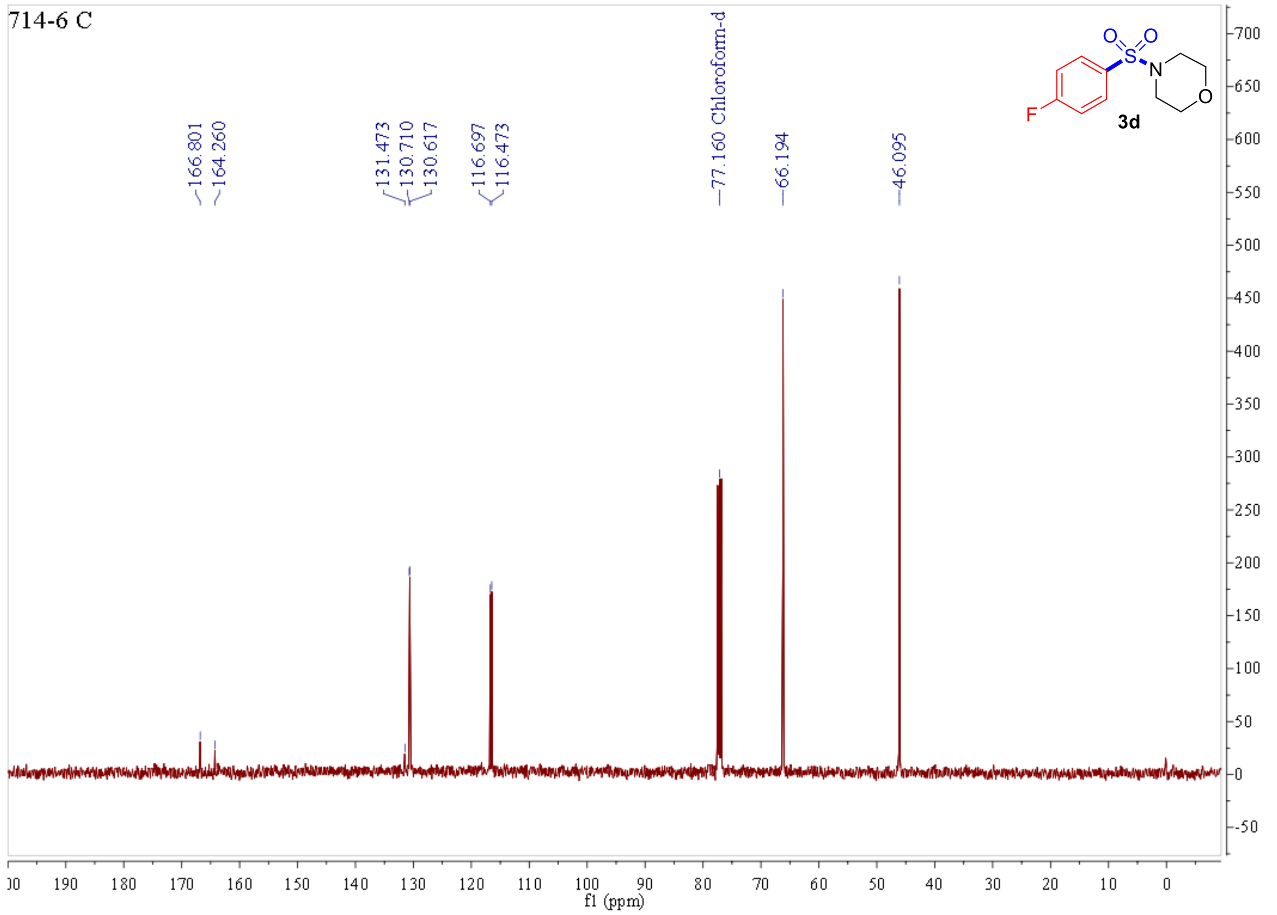
714-6 C
166.801
164.260
131.473
130.710
130.617
116.697
116.473
77.160 Chloroform-d
66.194
46.095
F
O
O
S
N
O
3d
f1 (ppm)

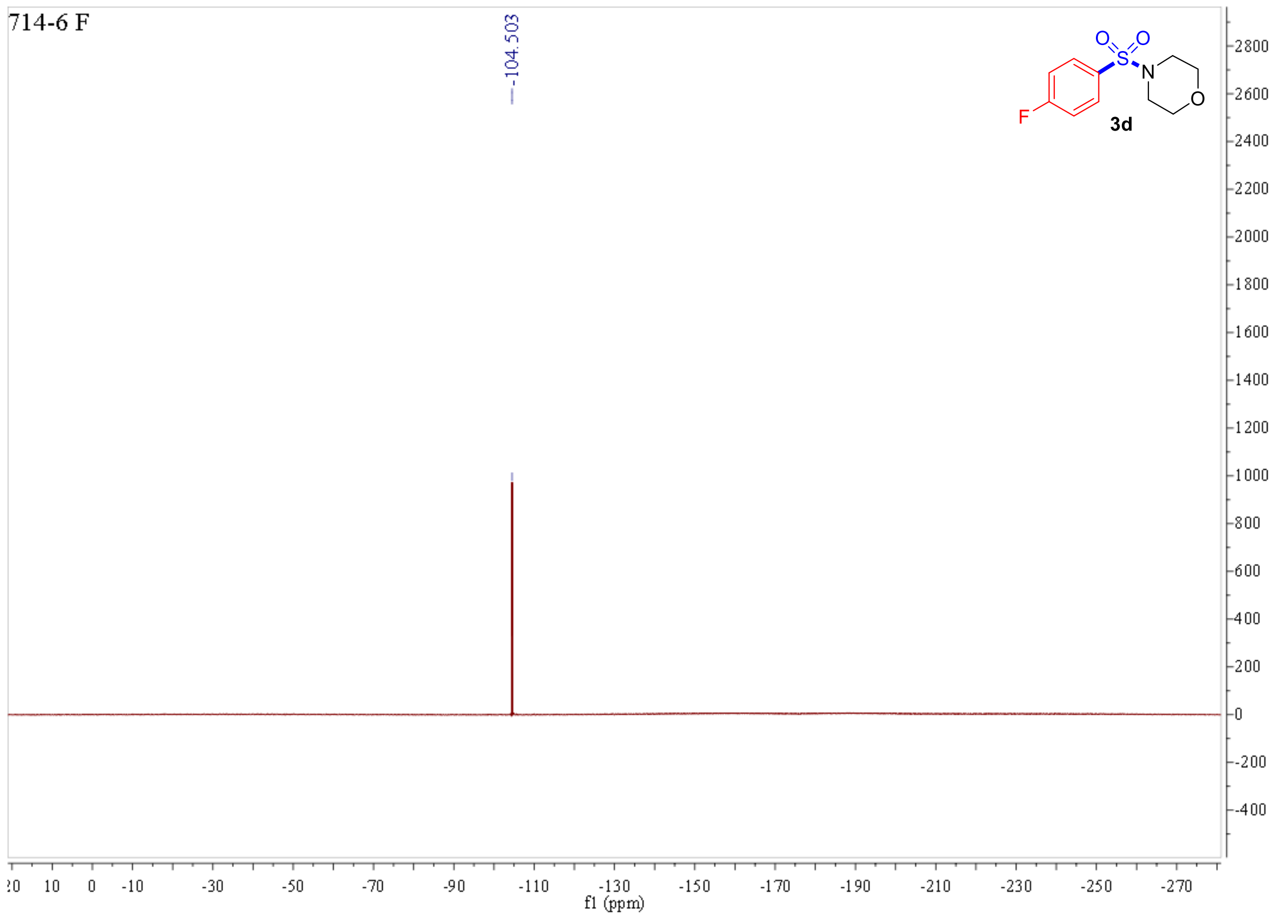
714-6 F
-104.503
3d
F
O
S
N
f1 (ppm)

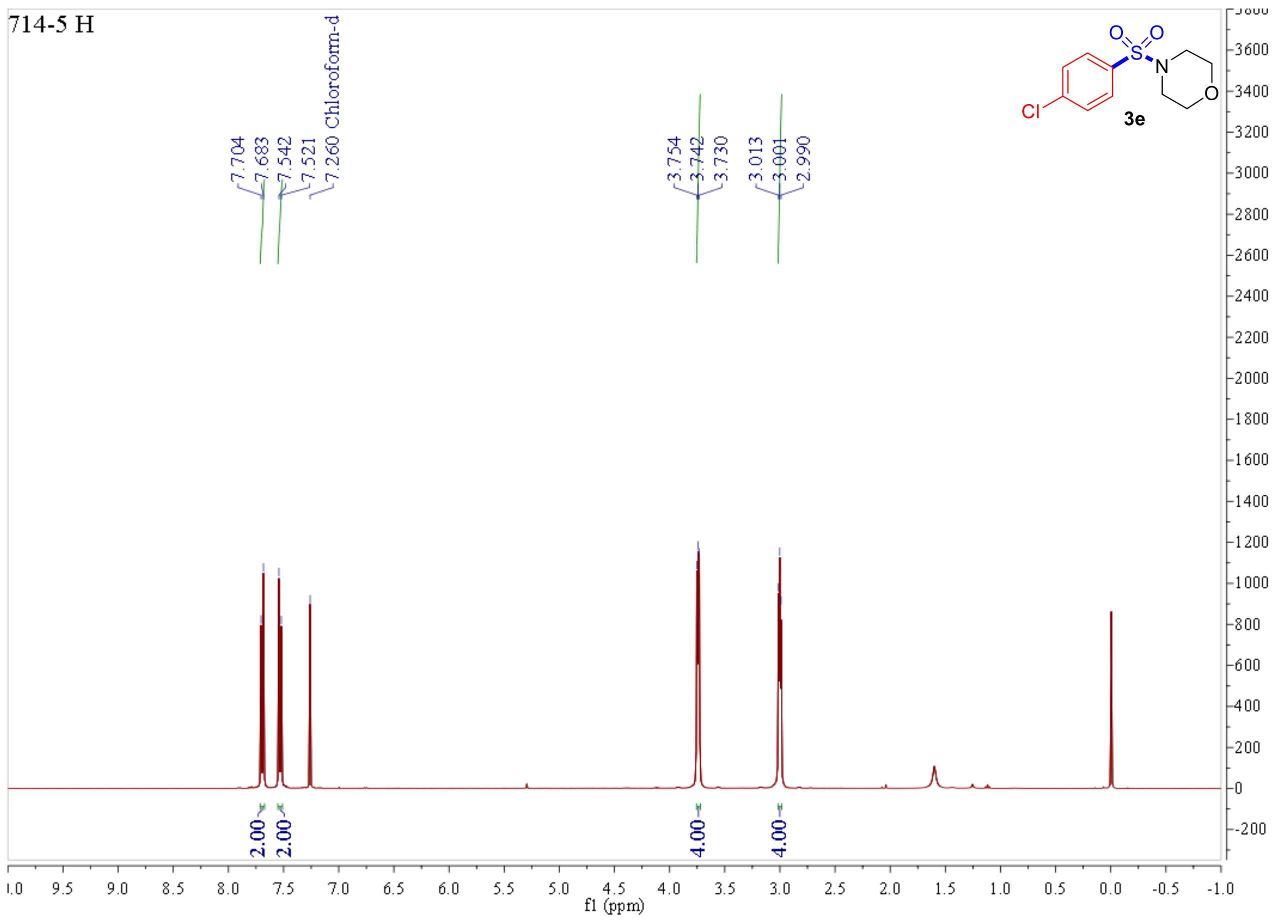
714-5 H
7.704
7.683
7.542
7.521
7.260 Chloroform-d
3.754
3.742
3.730
3.013
3.001
2.990
2.00
2.00
4.00
4.00
f1 (ppm)
3e

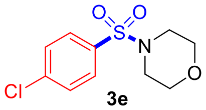

3e

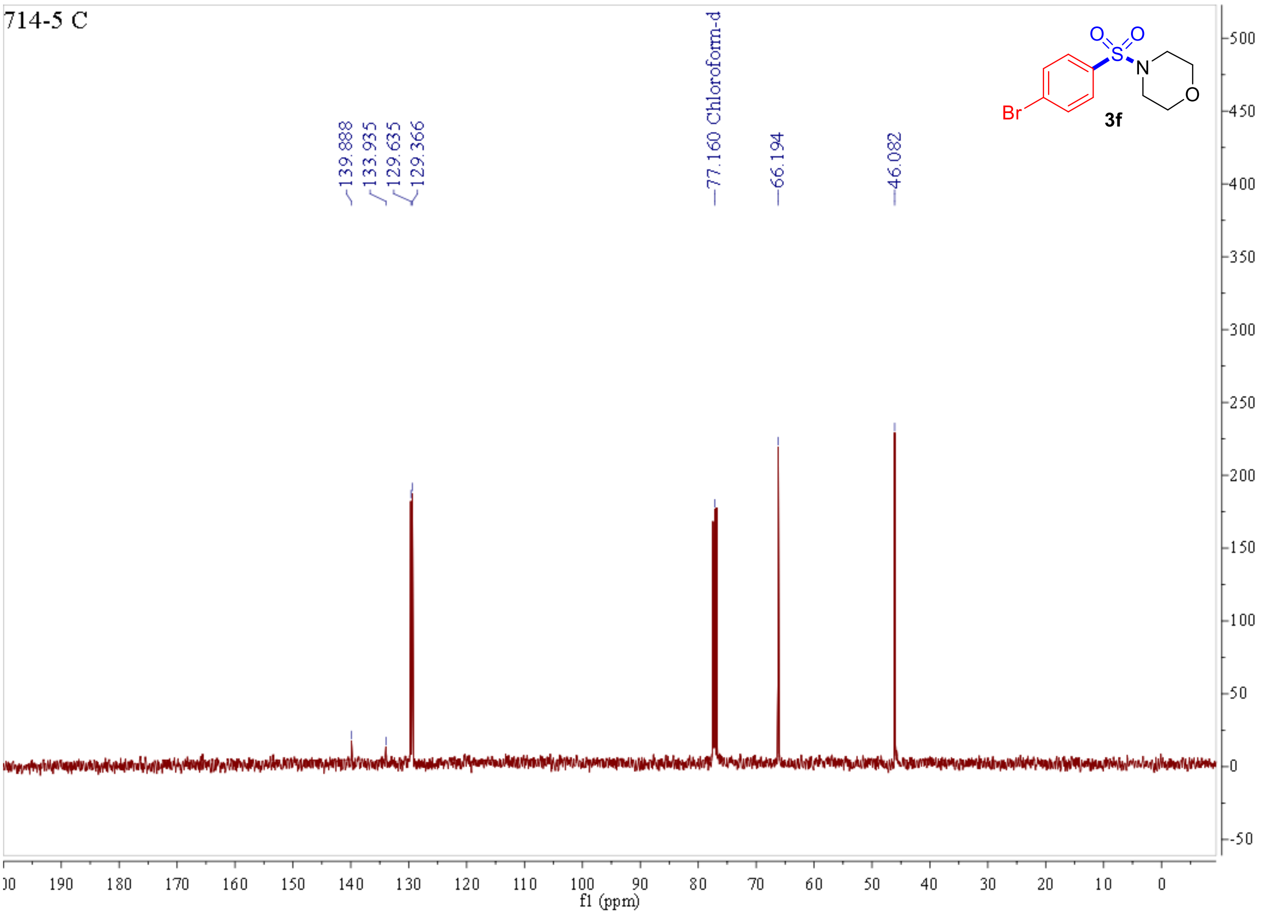
714-5 C
139.888
133.935
129.635
129.366
77.160 Chloroform-d
66.194
46.082
Br
O
S
O
N
O
3f
f1 (ppm)

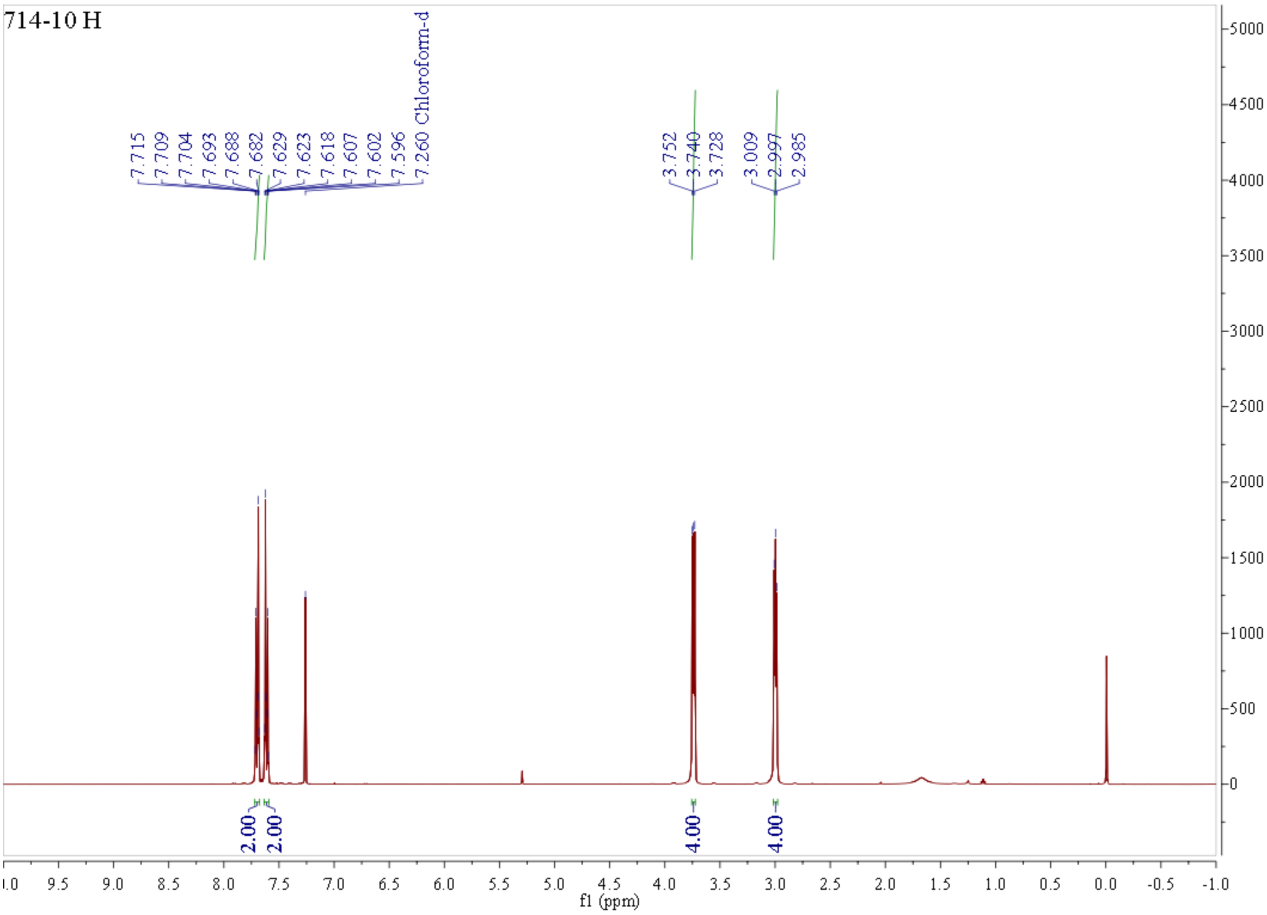
714-10 H
7.715
7.709
7.704
7.693
7.688
7.682
7.629
7.623
7.618
7.607
7.602
7.596
7.260 Chloroform-d
3.752
3.740
3.728
3.009
2.997
2.985
2.00
2.00
4.00
4.00
f1 (ppm)

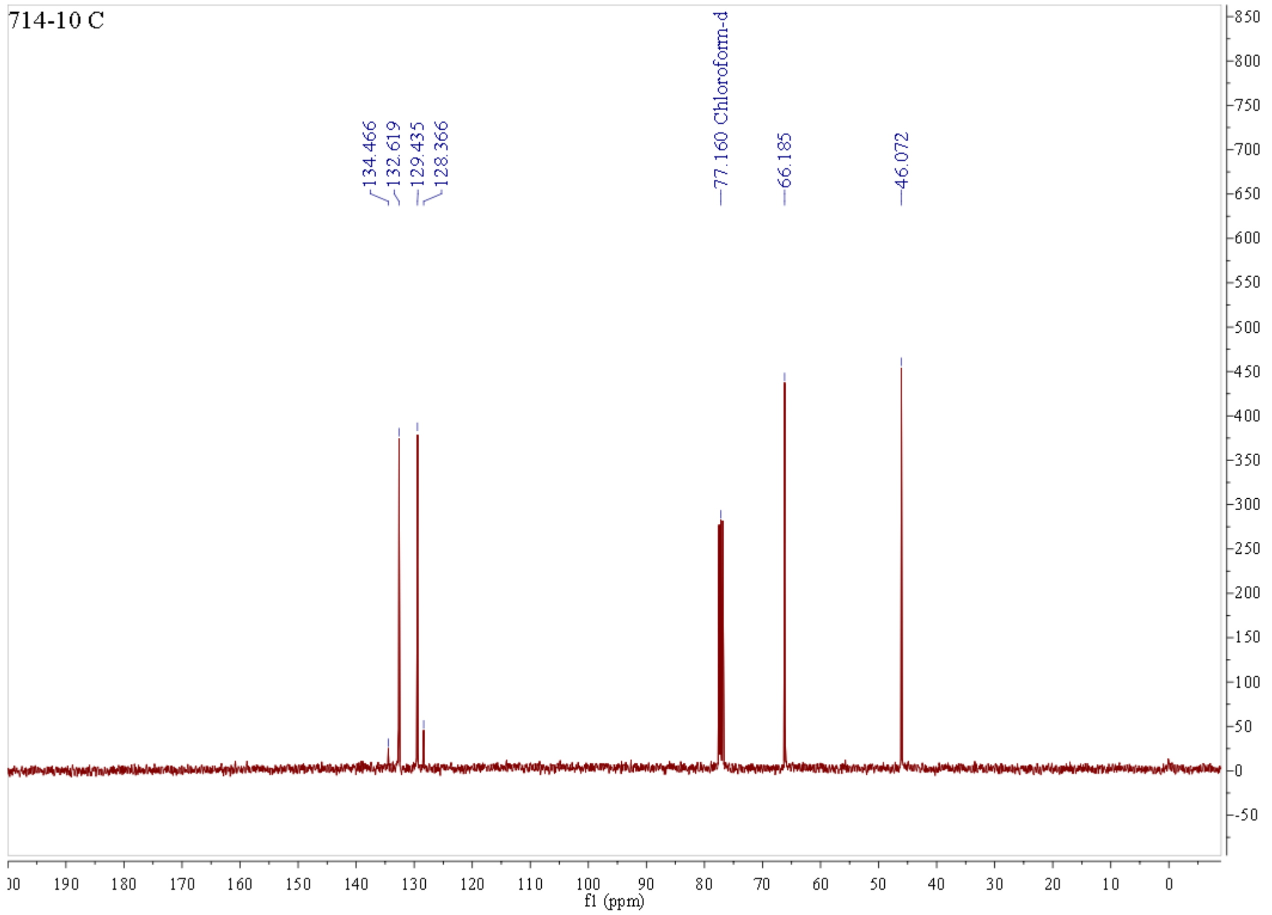
714-10 C
134.466
132.619
129.435
128.366
77.160 Chloroform-d
66.185
46.072
f1 (ppm)

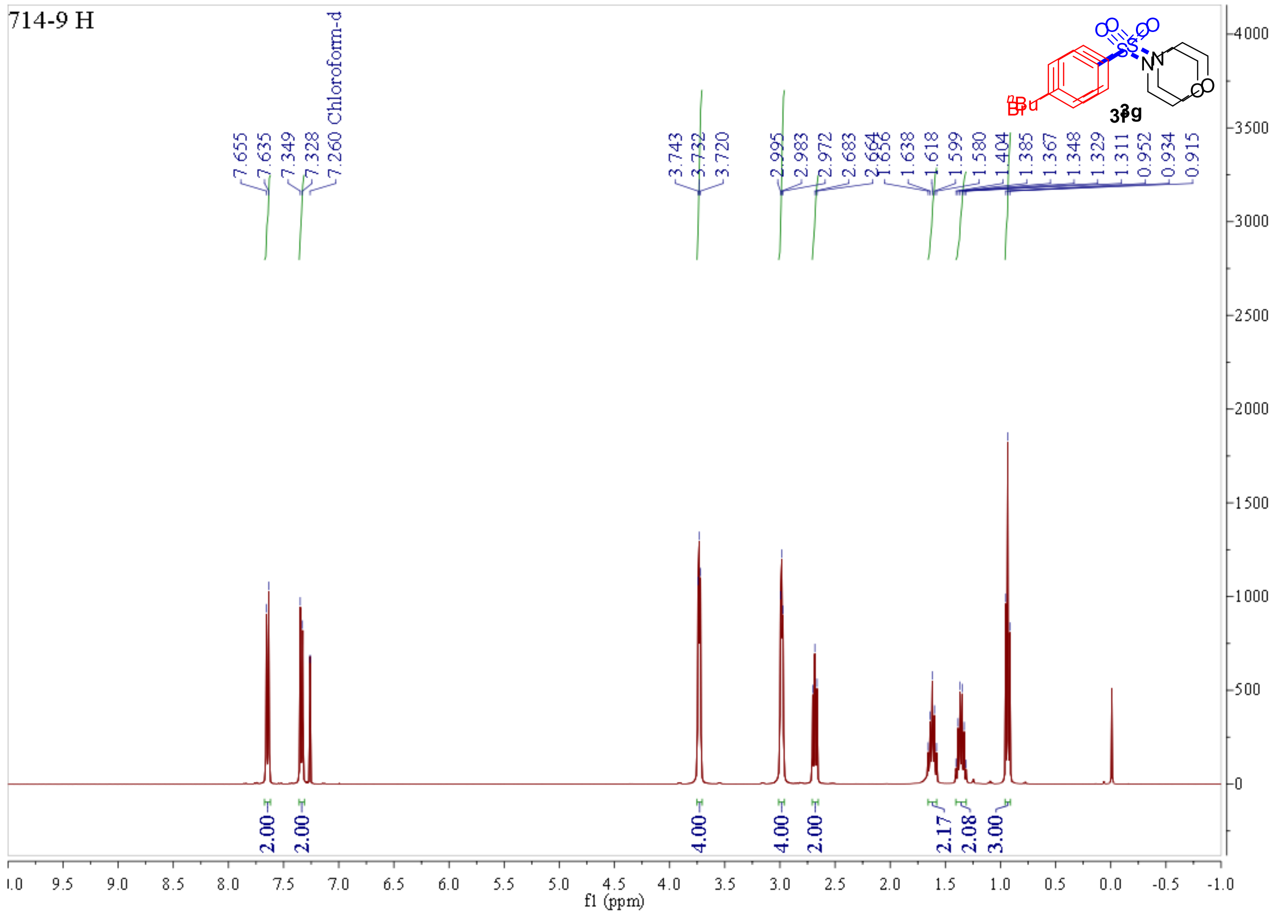
714-9 H
7.655
7.635
7.349
7.328
7.260 Chloroform-d
3.743
3.732
3.720
2.995
2.983
2.972
2.683
2.664
1.656
1.638
1.618
1.599
1.580
1.404
1.385
1.367
1.348
1.329
1.311
0.952
0.934
0.915
2.00
2.00
4.00
4.00
2.00
2.17
2.08
3.00
f1 (ppm)

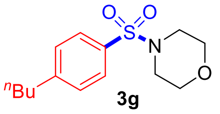
^{n}Bu

3g

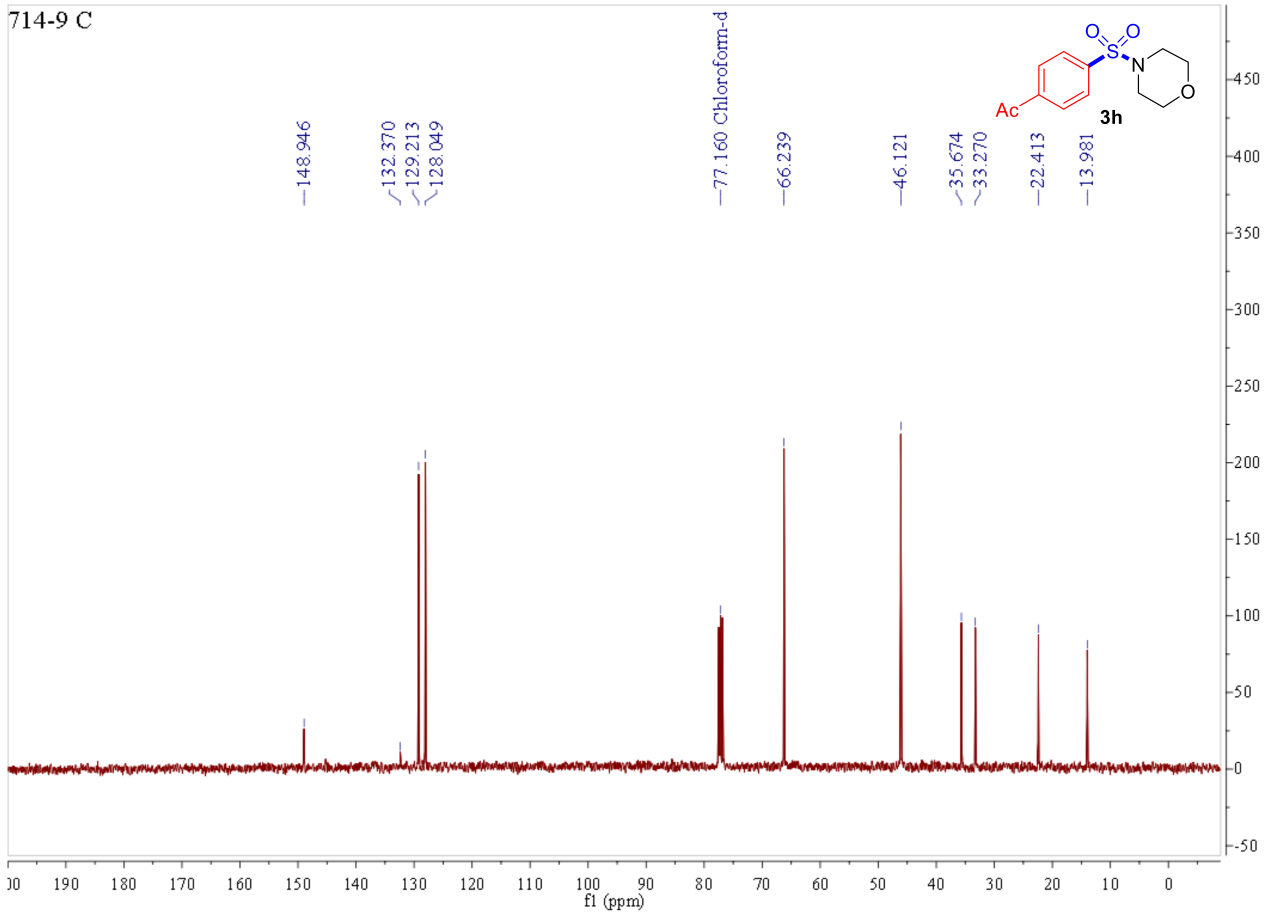
714-9 C
148.946
132.370
129.213
128.049
77.160 Chloroform-d
66.239
46.121
35.674
33.270
22.413
13.981
Ac
O
S
N
3h
f1 (ppm)

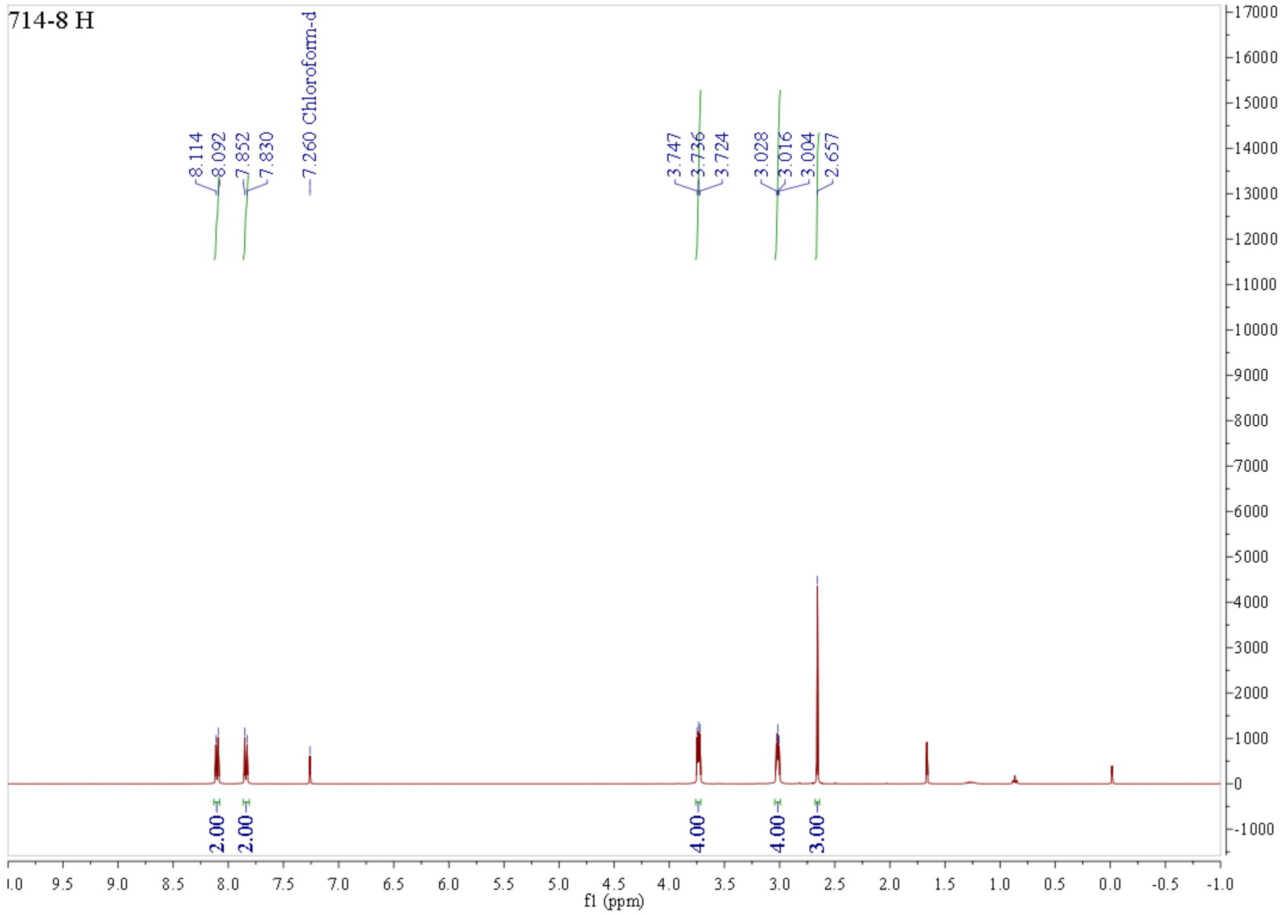
714-8 H
8.114
8.092
7.852
7.830
7.260 Chloroform-d
3.747
3.736
3.724
3.028
3.016
3.004
2.657
2.00
2.00
4.00
4.00
3.00
f1 (ppm)

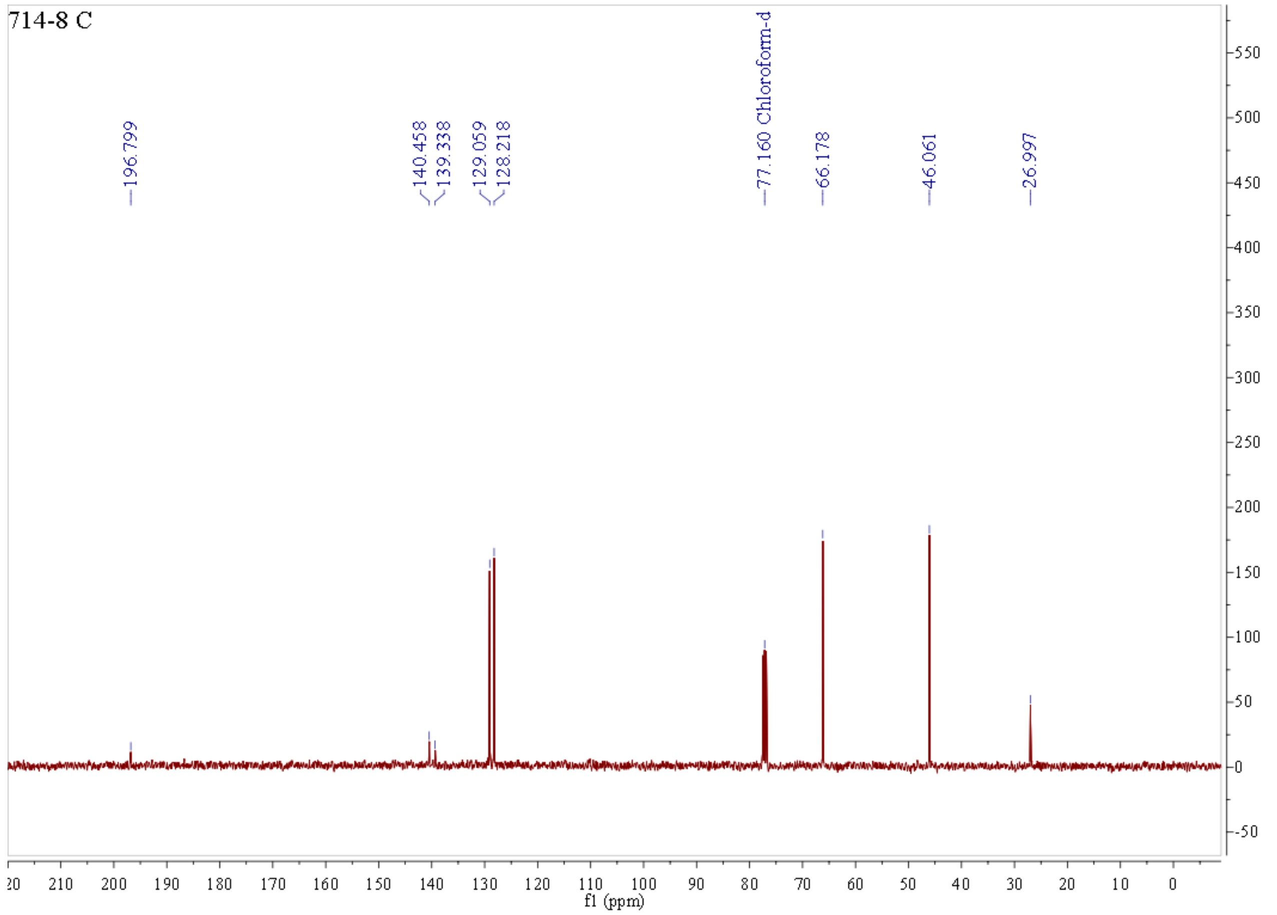
714-8 C
196.799
140.458
139.338
129.059
128.218
77.160 Chloroform-d
66.178
46.061
26.997
f1 (ppm)

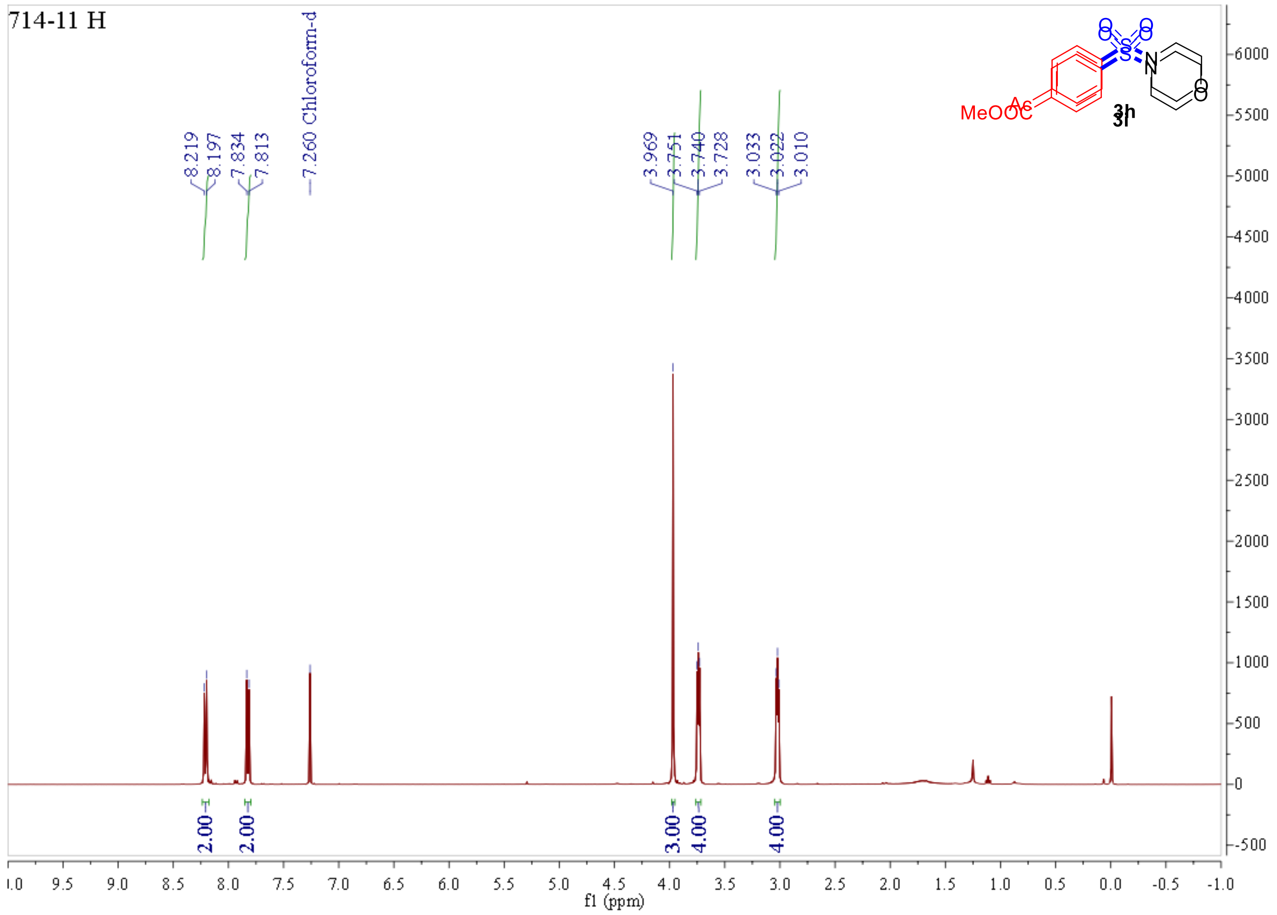
714-11 H
8.219
8.197
7.834
7.813
7.260 Chloroform-d
3.969
3.751
3.740
3.728
3.033
3.022
3.010
2.00
2.00
3.00
4.00
4.00
f1 (ppm)

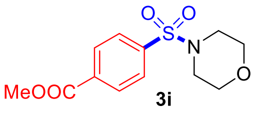
MeOOC
3i

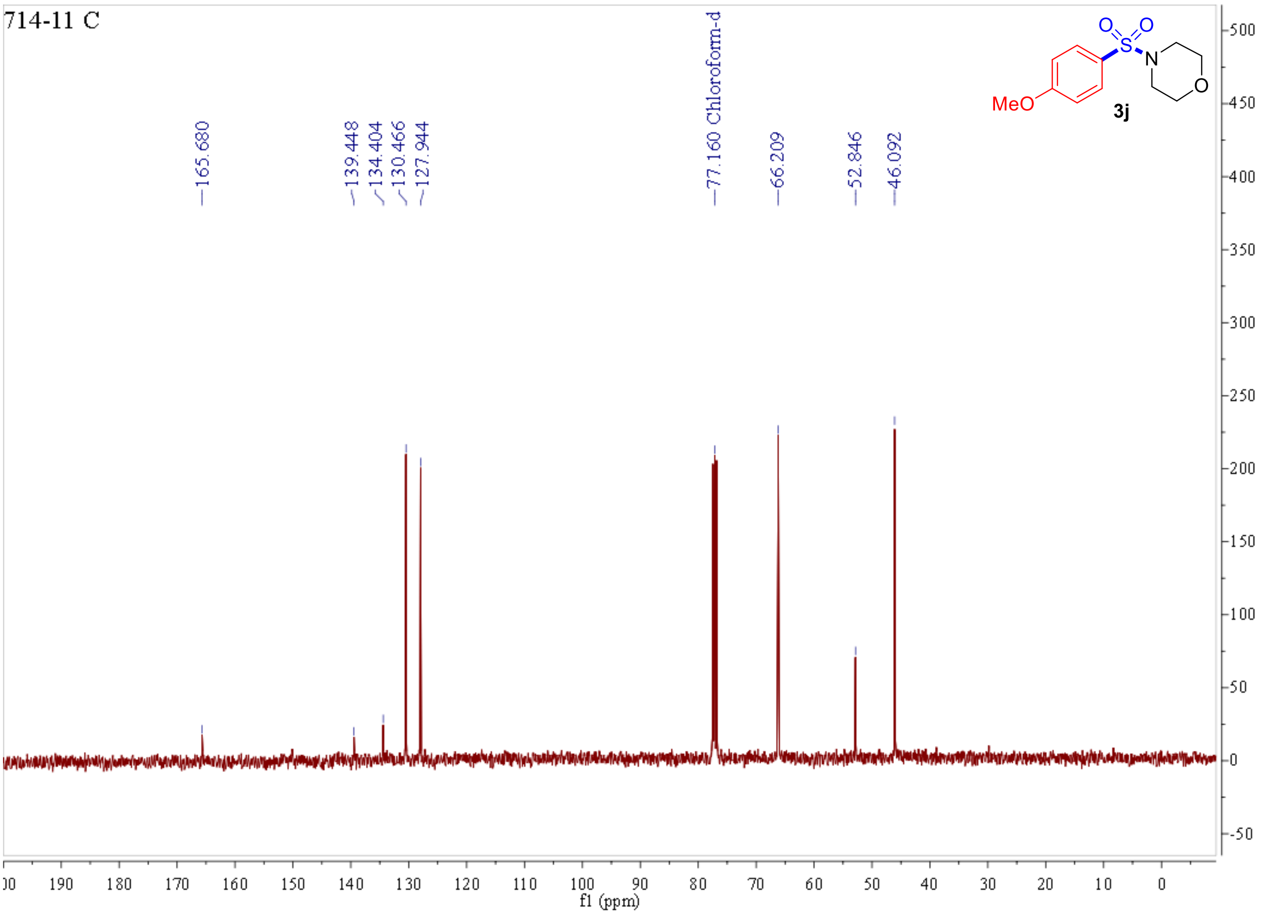
714-11 C
165.680
139.448
134.404
130.466
127.944
77.160 Chloroform-d
66.209
52.846
46.092
MeO
O
S
O
N
O
3j
f1 (ppm)

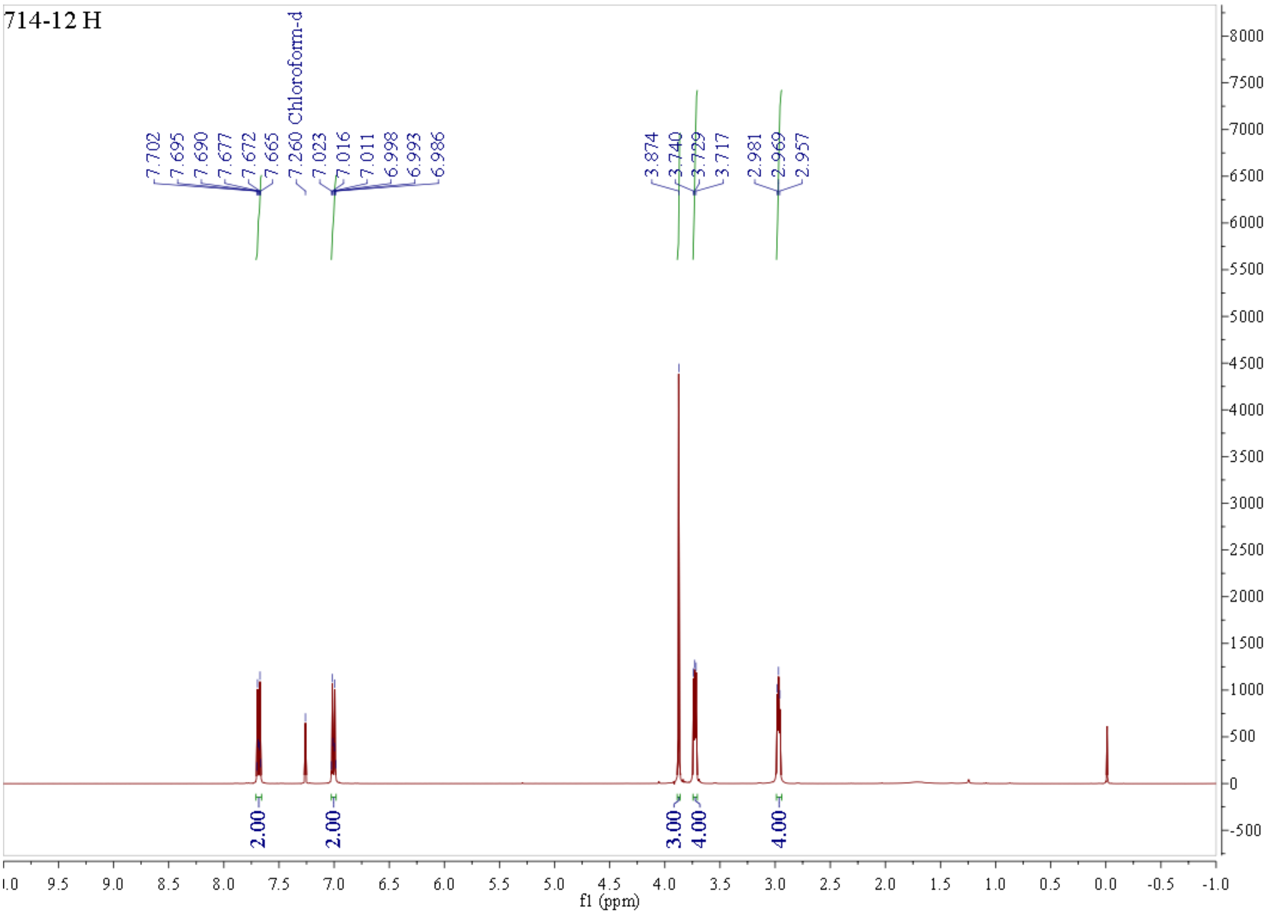
714-12 H
7.702
7.695
7.690
7.677
7.672
7.665
7.260 Chloroform-d
7.023
7.016
7.011
6.998
6.993
6.986
3.874
3.740
3.729
3.717
2.981
2.969
2.957
2.00
2.00
3.00
4.00
4.00
f1 (ppm)

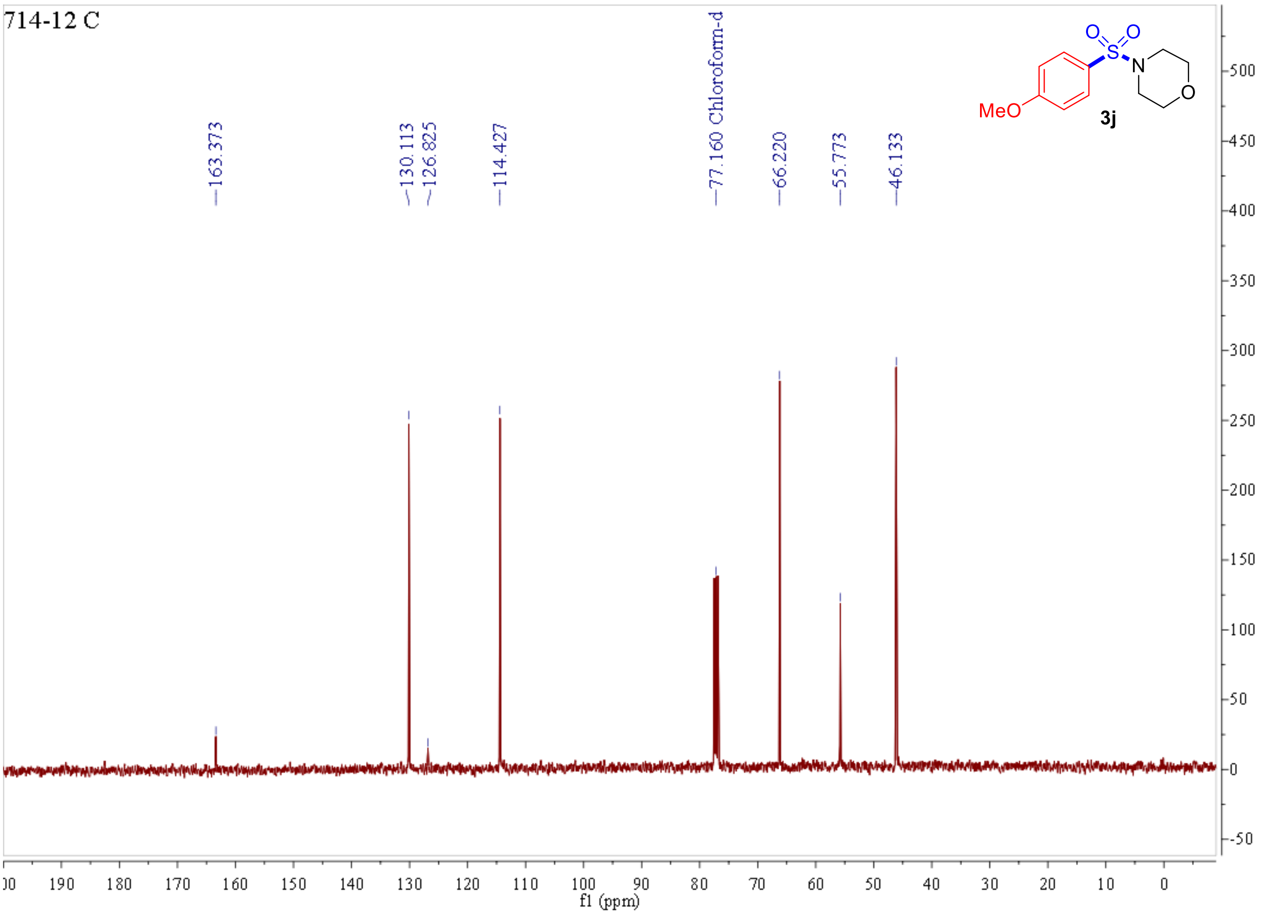
714-12 C
163.373
130.113
126.825
114.427
77.160 Chloroform-d
66.220
55.773
46.133
MeO
O
S
O
N
O
3j
f1 (ppm)

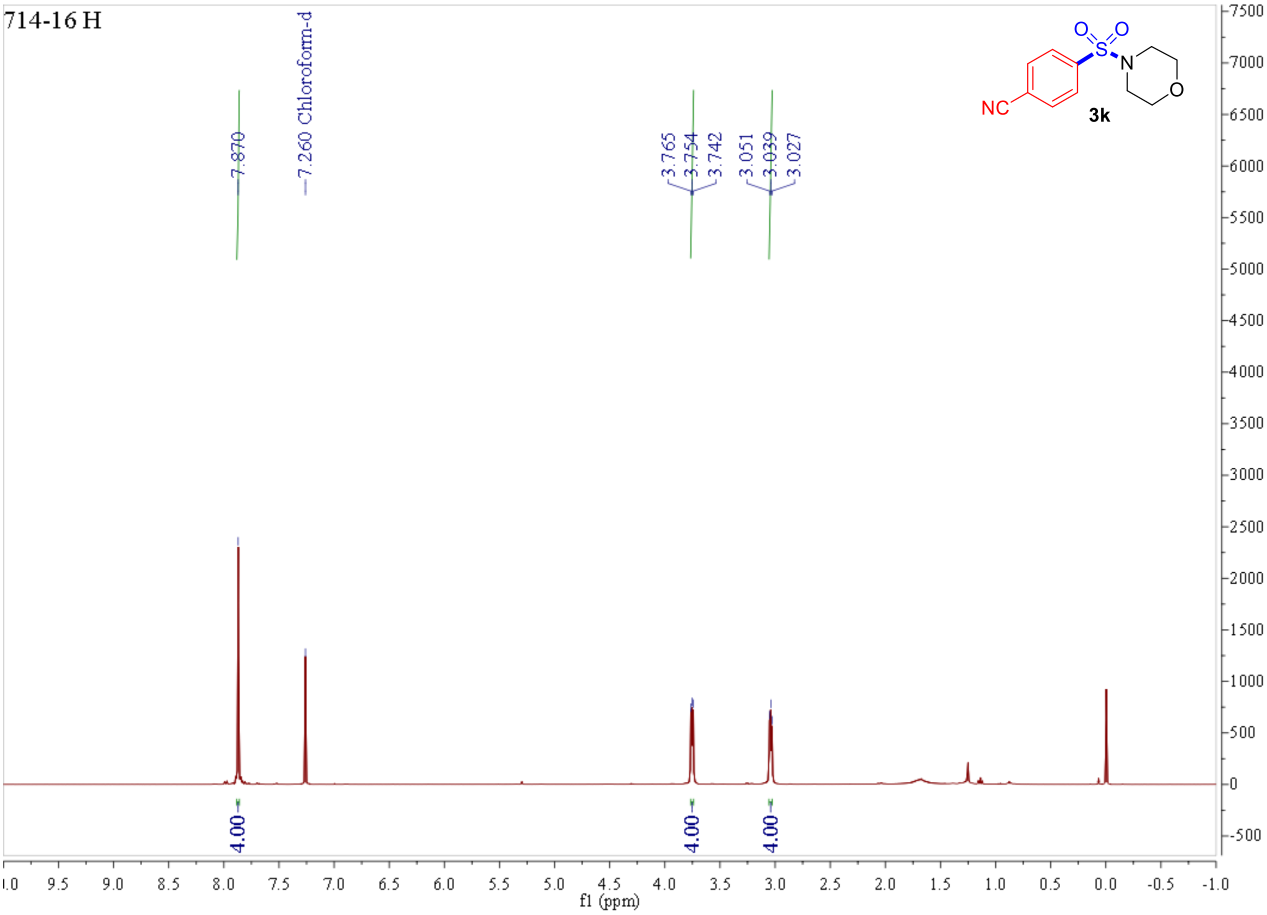
714-16 H
7.870
7.260 Chloroform-d
3.765
3.754
3.742
3.051
3.039
3.027
NC
O
S
O
N
O
3k
4.00
4.00
4.00
f1 (ppm)

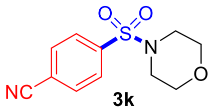
NC
O
S
O
N
O

3k

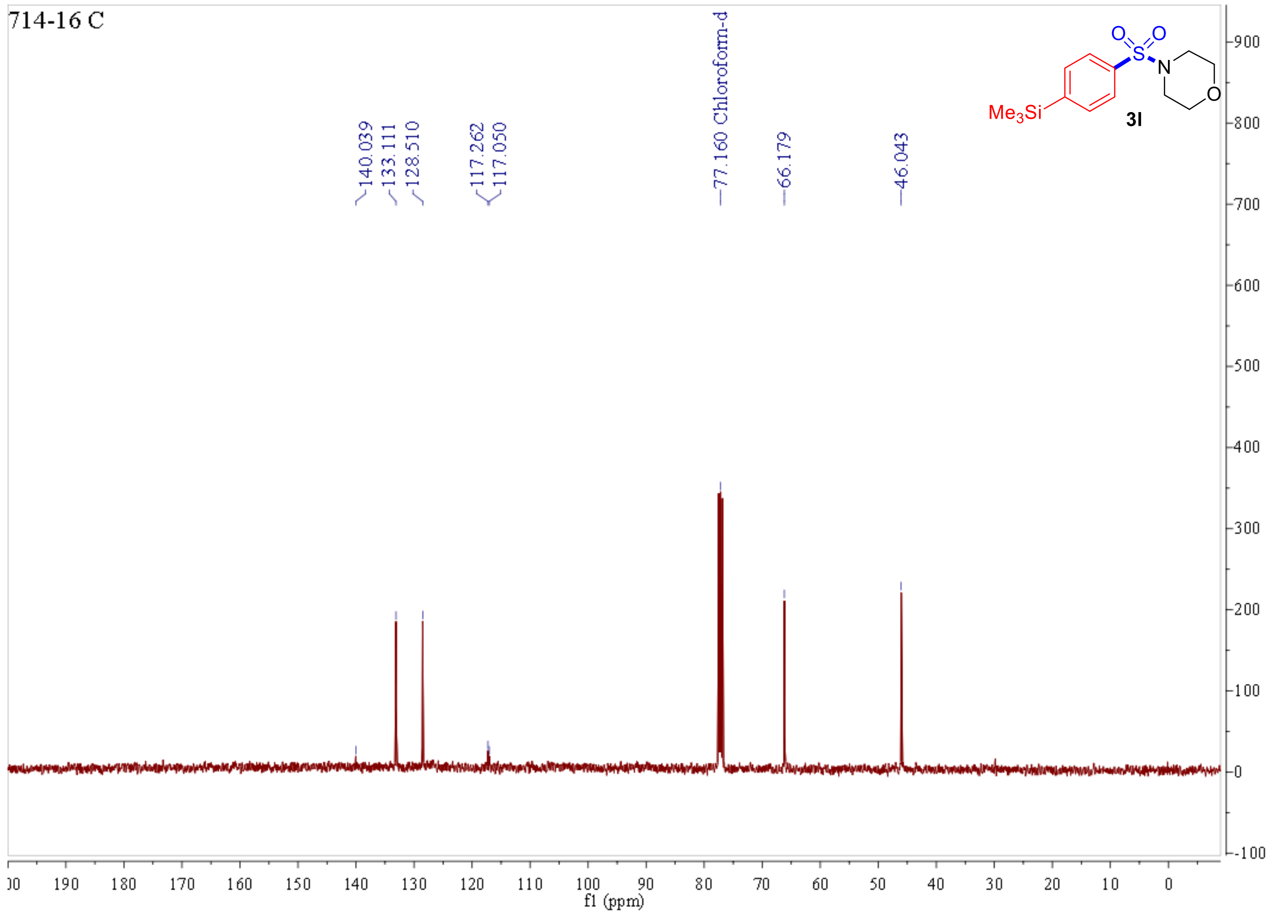
714-16 C
140.039
133.111
128.510
117.262
117.050
77.160 Chloroform-d
66.179
46.043
Me3Si
3l
f1 (ppm)

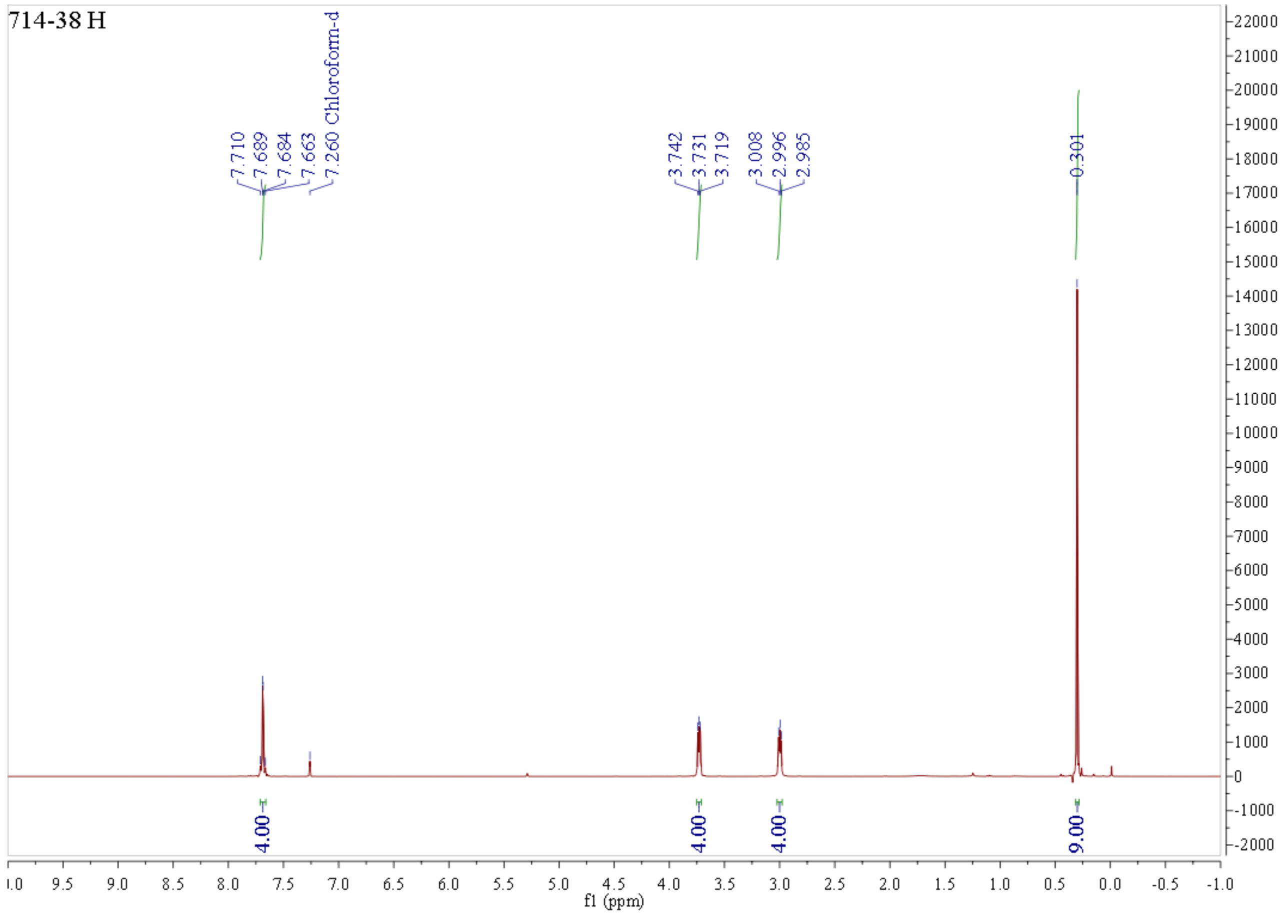
714-38 H
7.710
7.689
7.684
7.663
7.260 Chloroform-d
3.742
3.731
3.719
3.008
2.996
2.985
0.301
4.00
4.00
4.00
9.00
f1 (ppm)

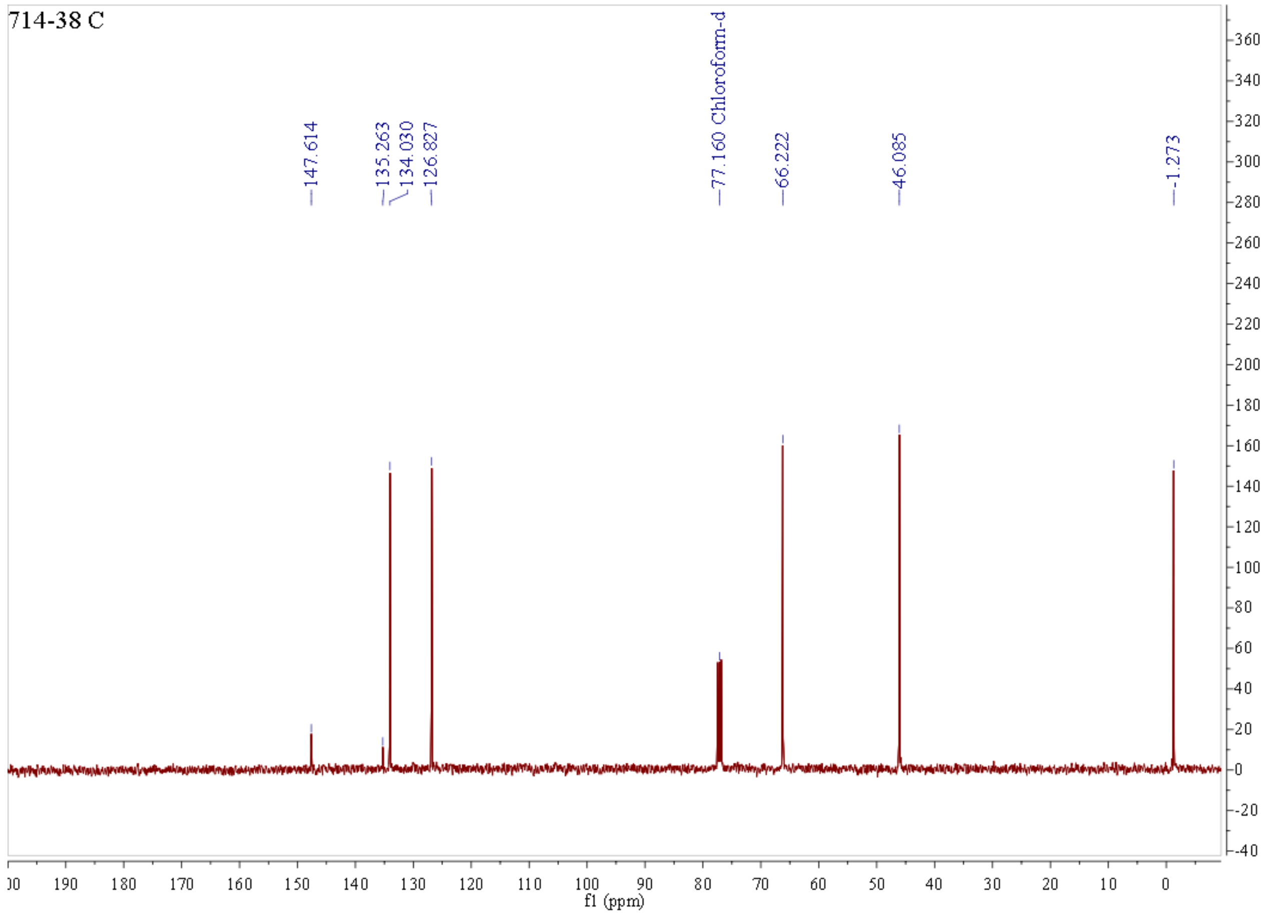
714-38 C
147.614
135.263
134.030
126.827
77.160 Chloroform-d
66.222
46.085
-1.273
f1 (ppm)

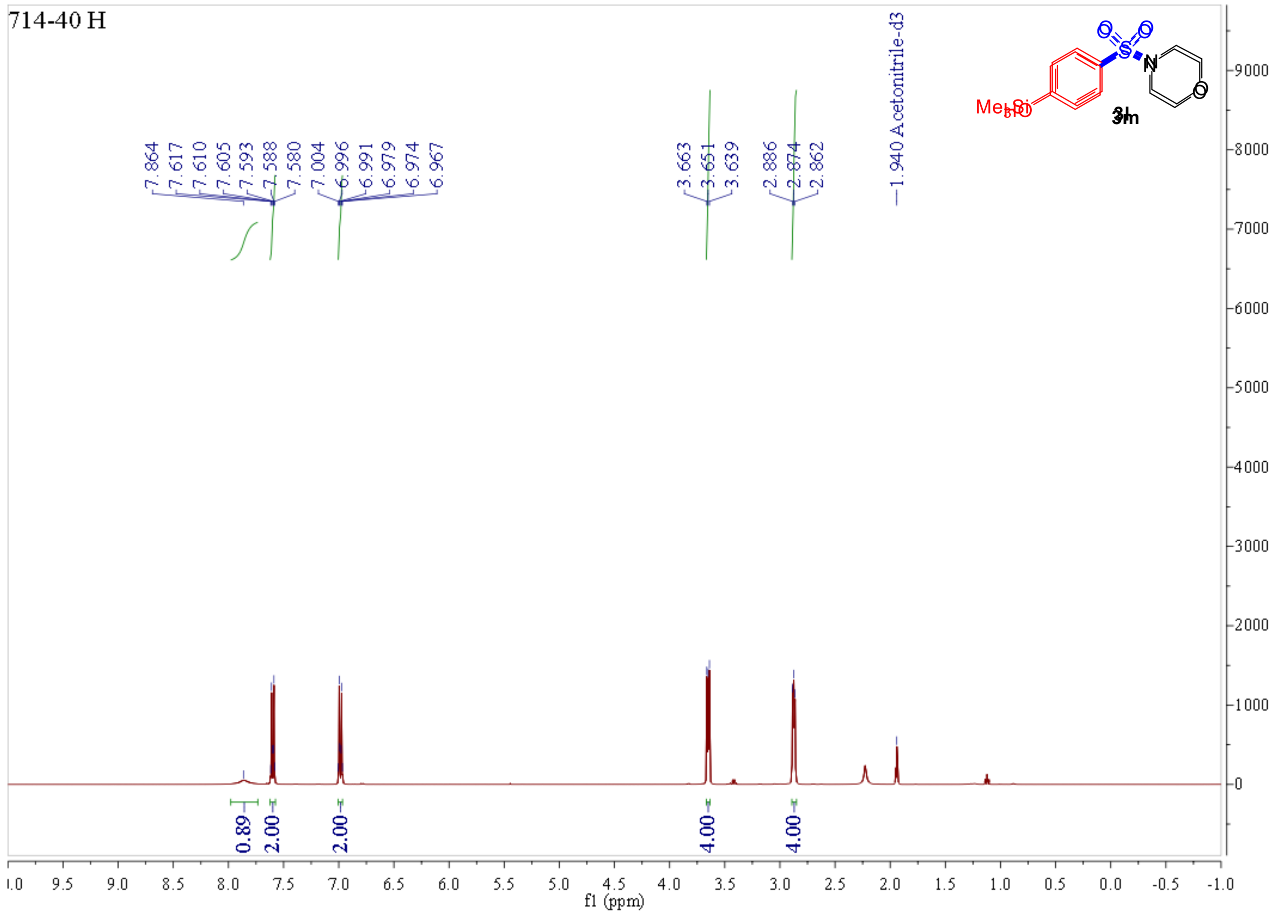
714-40 H
1.940 Acetonitrile-d3
0.89
2.00
2.00
4.00
4.00
f1 (ppm)

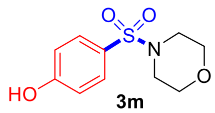

3m

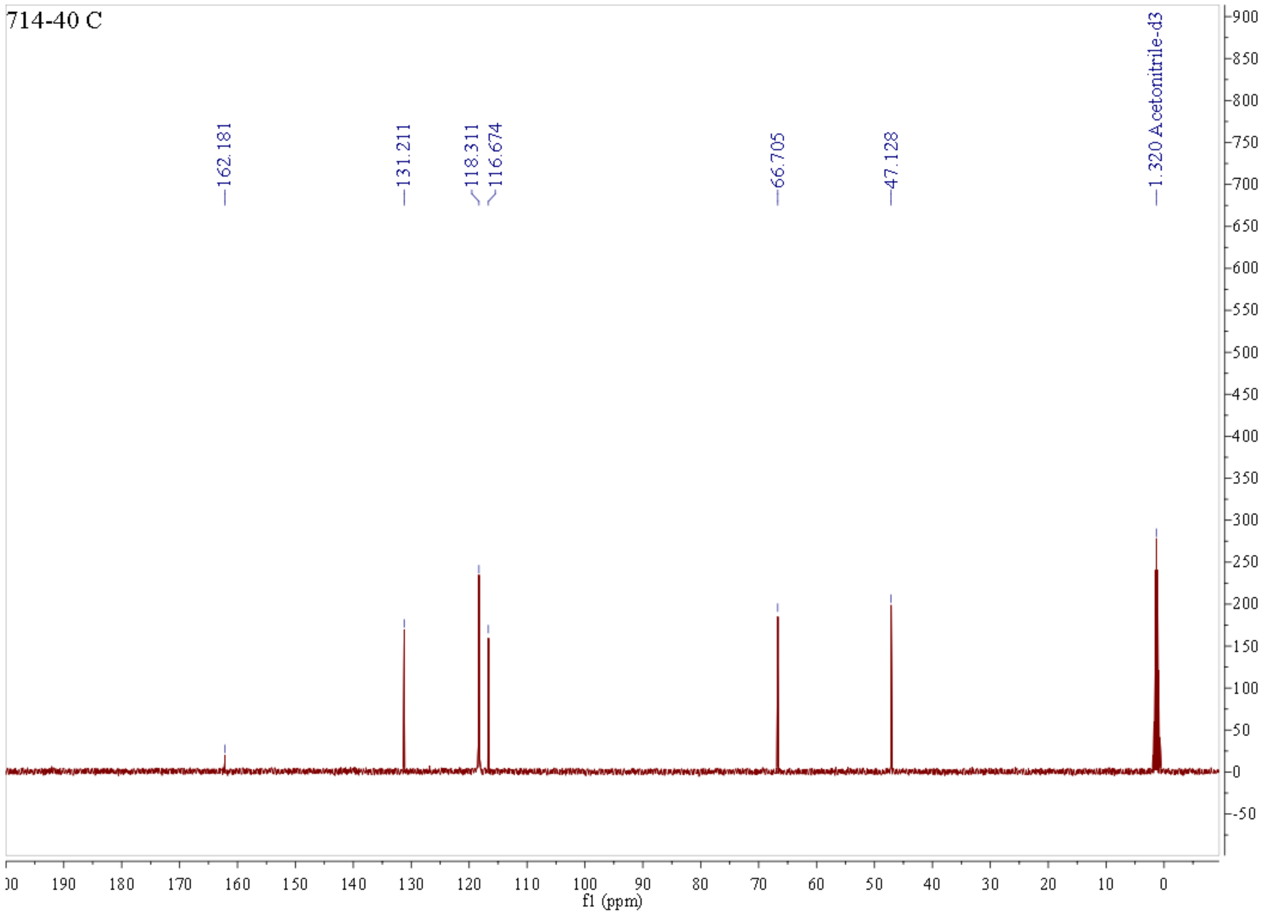
714-40 C
162.181
131.211
118.311
116.674
66.705
47.128
1.320 Acetonitrile-d3
f1 (ppm)

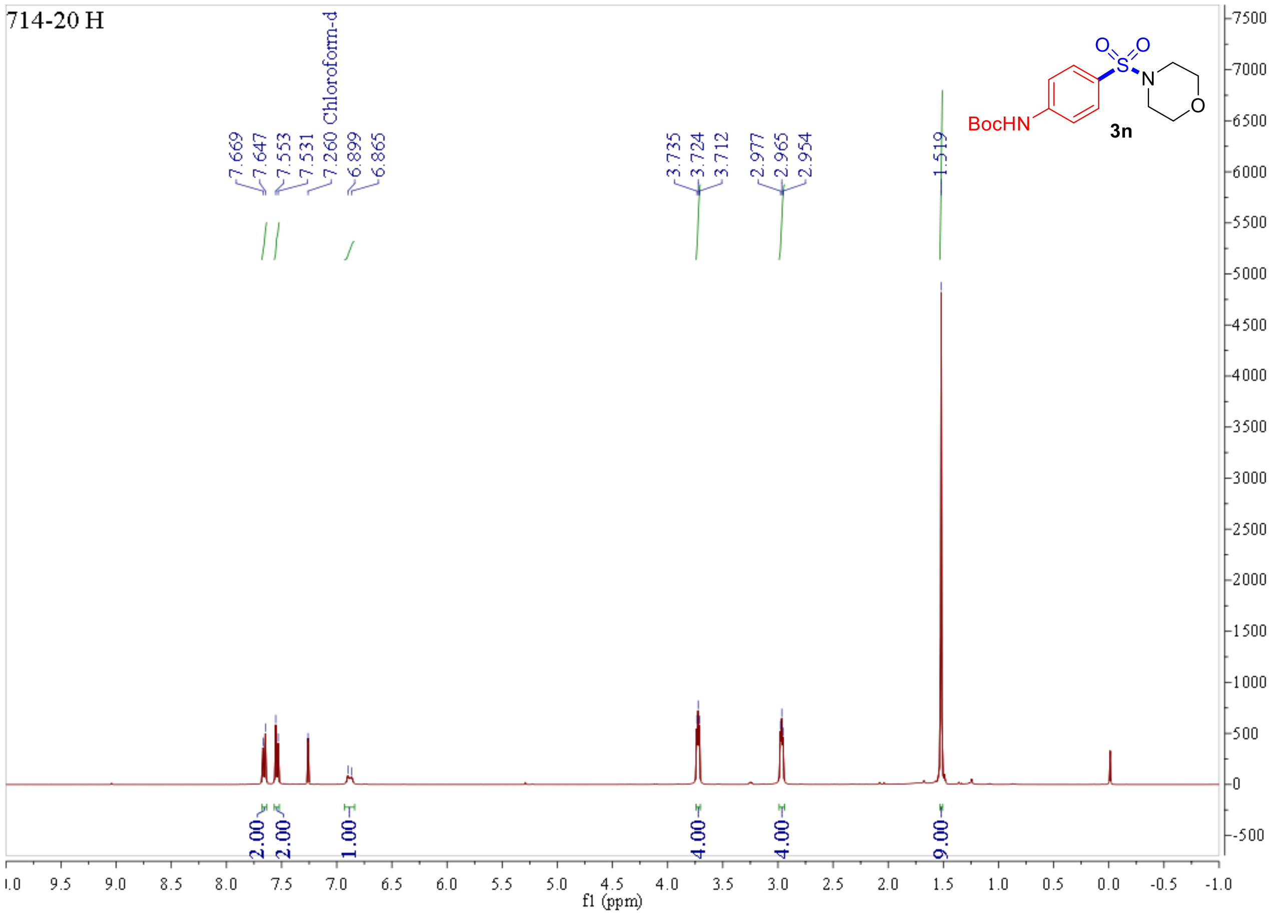
714-20 H
7.669
7.647
7.553
7.531
7.260 Chloroform-d
6.899
6.865
3.735
3.724
3.712
2.977
2.965
2.954
1.519
2.00
2.00
1.00
4.00
4.00
9.00
BocHN
3n
f1 (ppm)

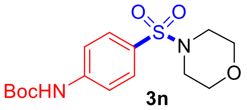
BocHN
3n

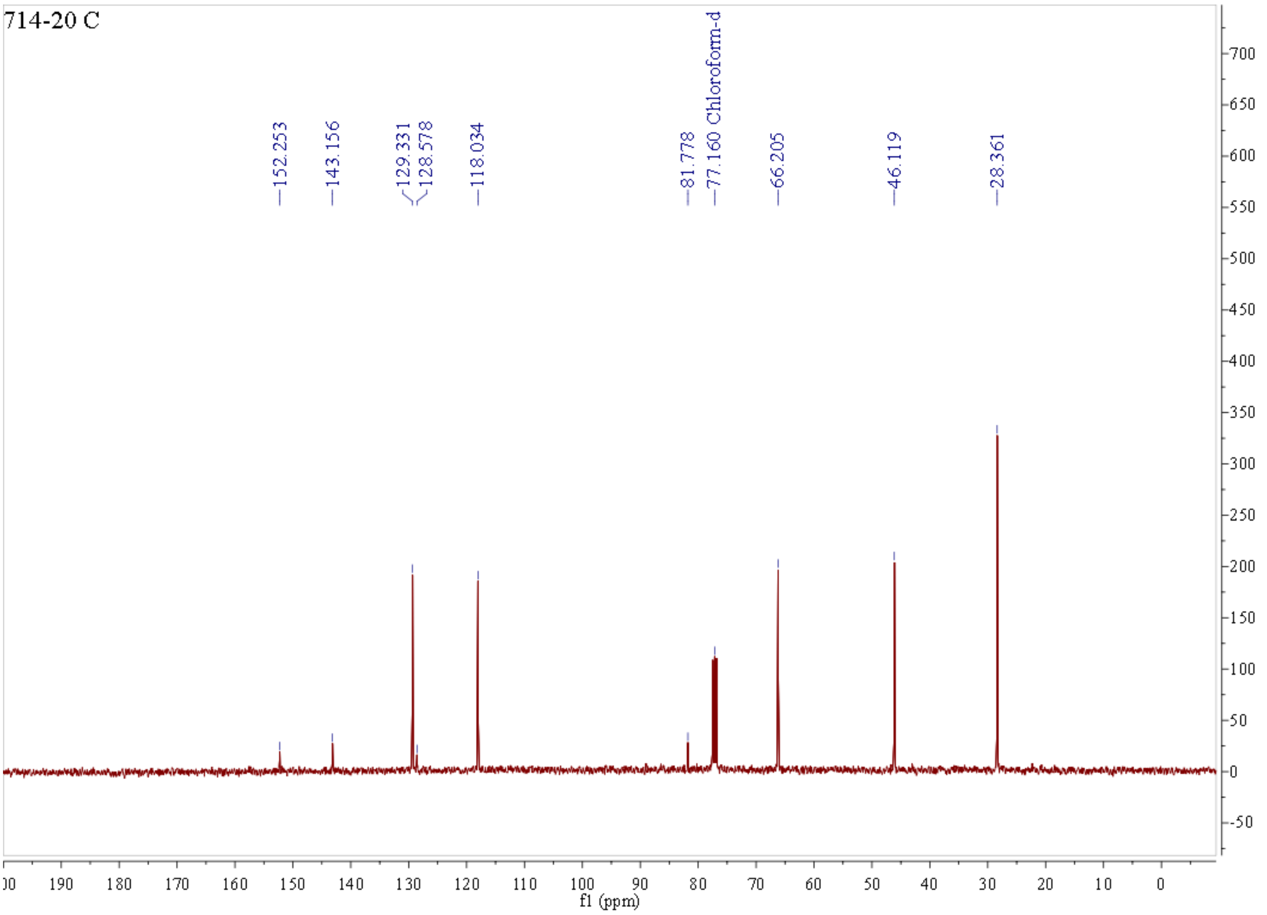
714-20 C
152.253
143.156
129.331
128.578
118.034
81.778
77.160 Chloroform-d
66.205
46.119
28.361
f1 (ppm)

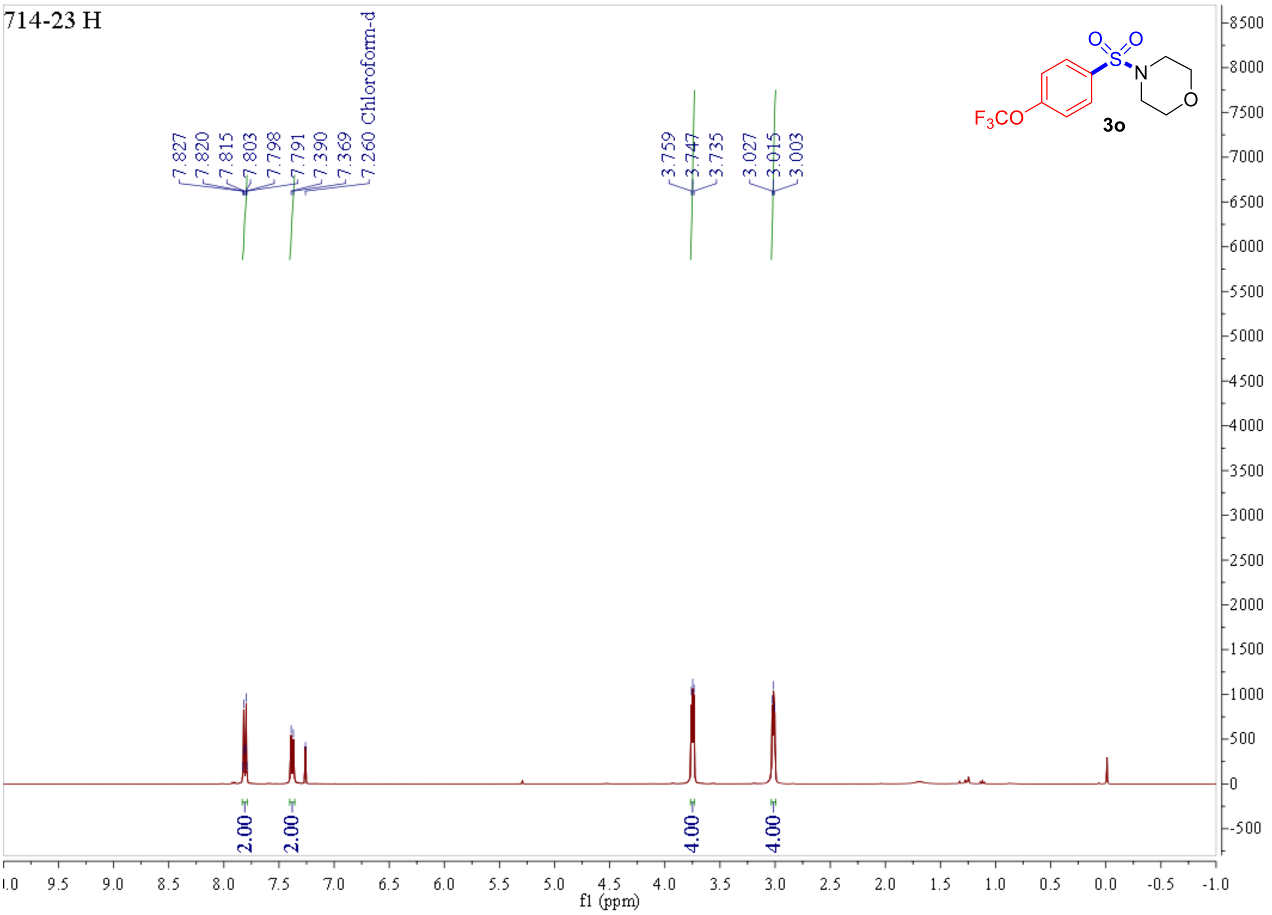
714-23 H
7.827
7.820
7.815
7.803
7.798
7.791
7.390
7.369
7.260 Chloroform-d
3.759
3.747
3.735
3.027
3.015
3.003
2.00
2.00
4.00
4.00
f1 (ppm)
3o

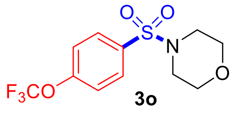
3o

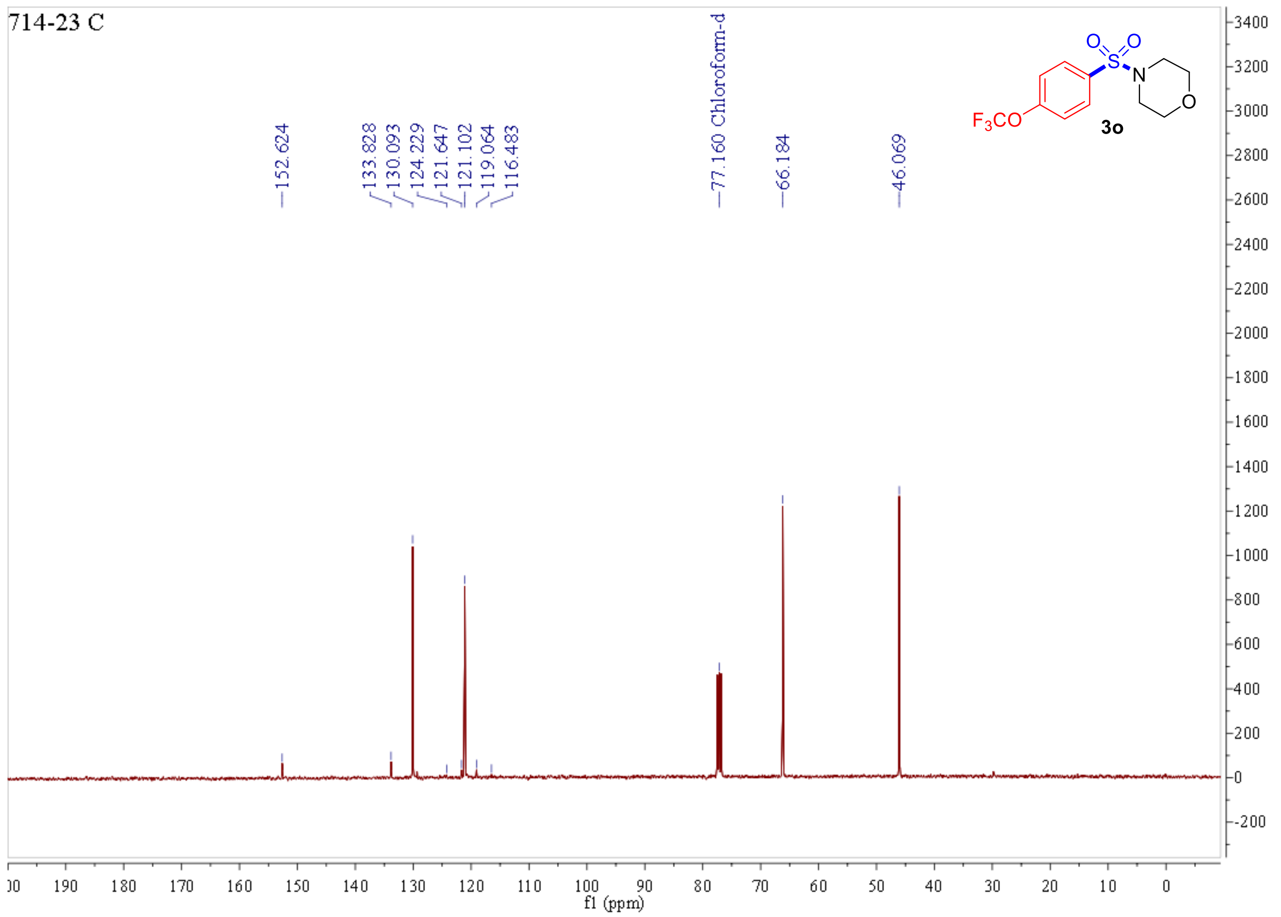
714-23 C
152.624
133.828
130.093
124.229
121.647
121.102
119.064
116.483
77.160 Chloroform-d
66.184
46.069
F3CO
3o
f1 (ppm)

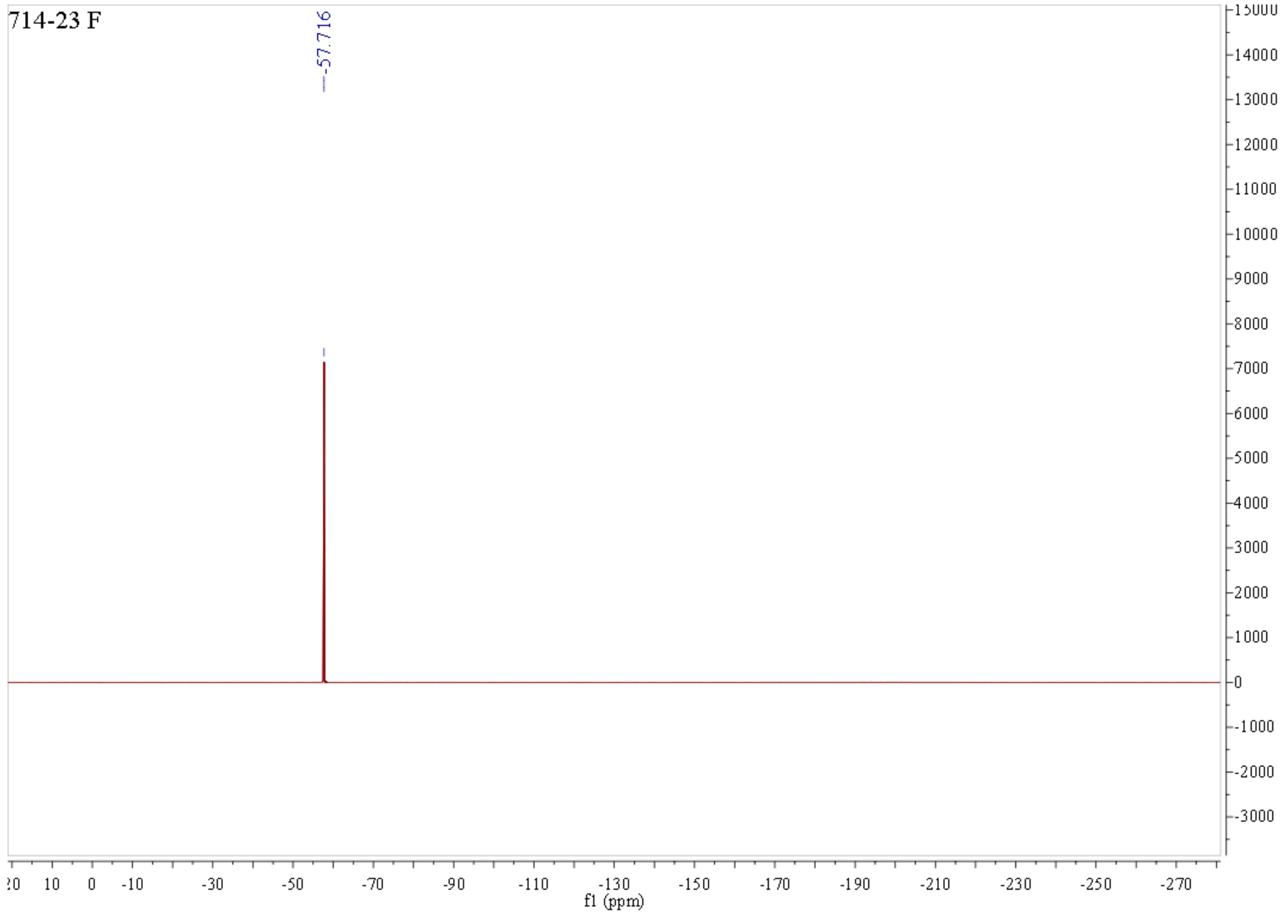
714-23 F
-57.716
f1 (ppm)

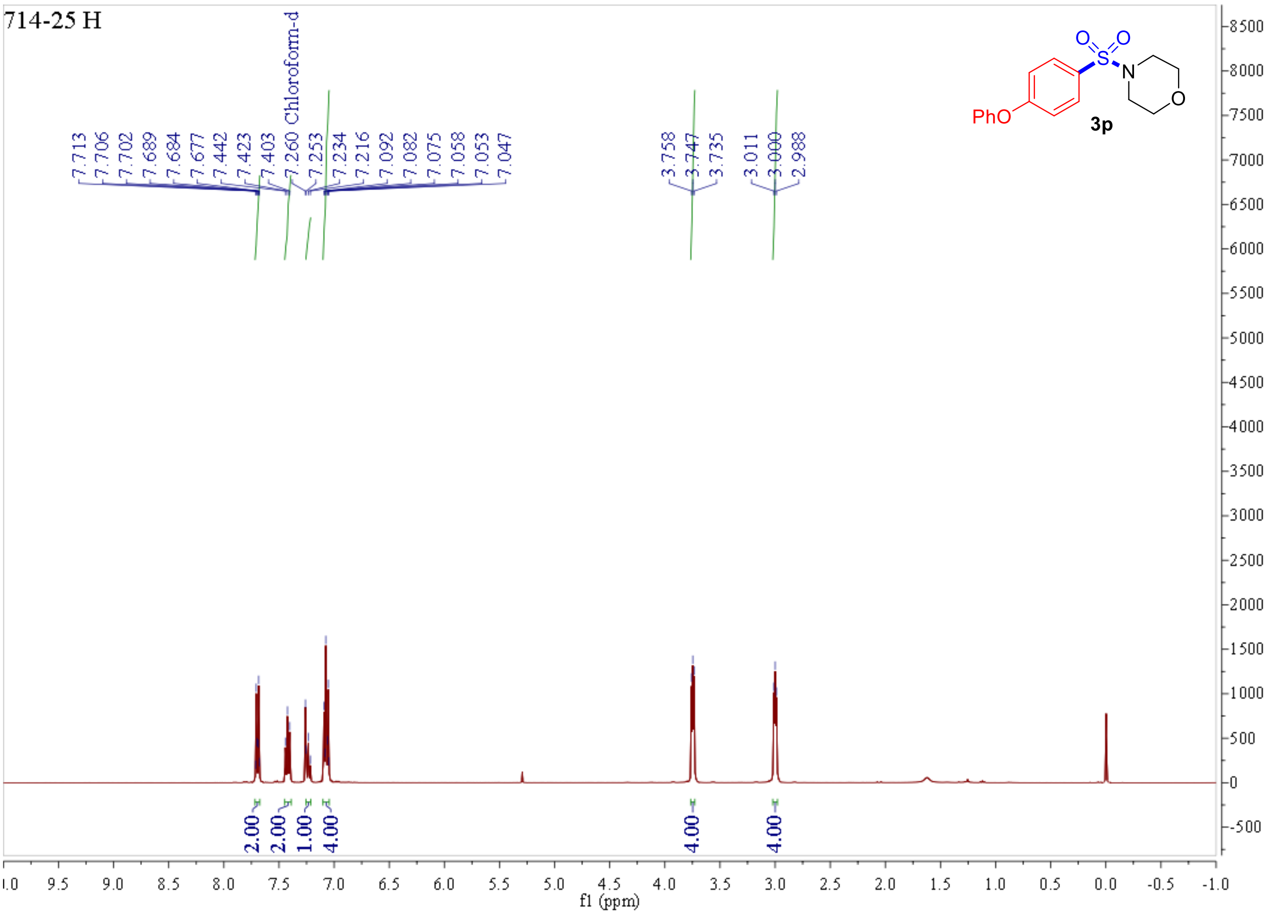
714-25 H
7.713
7.706
7.702
7.689
7.684
7.677
7.442
7.423
7.403
7.260 Chloroform-d
7.253
7.234
7.216
7.092
7.082
7.075
7.058
7.053
7.047
3.758
3.747
3.735
3.011
3.000
2.988
PhO
O
S
O
N
O
3p
2.00
2.00
1.00
4.00
4.00
4.00
f1 (ppm)

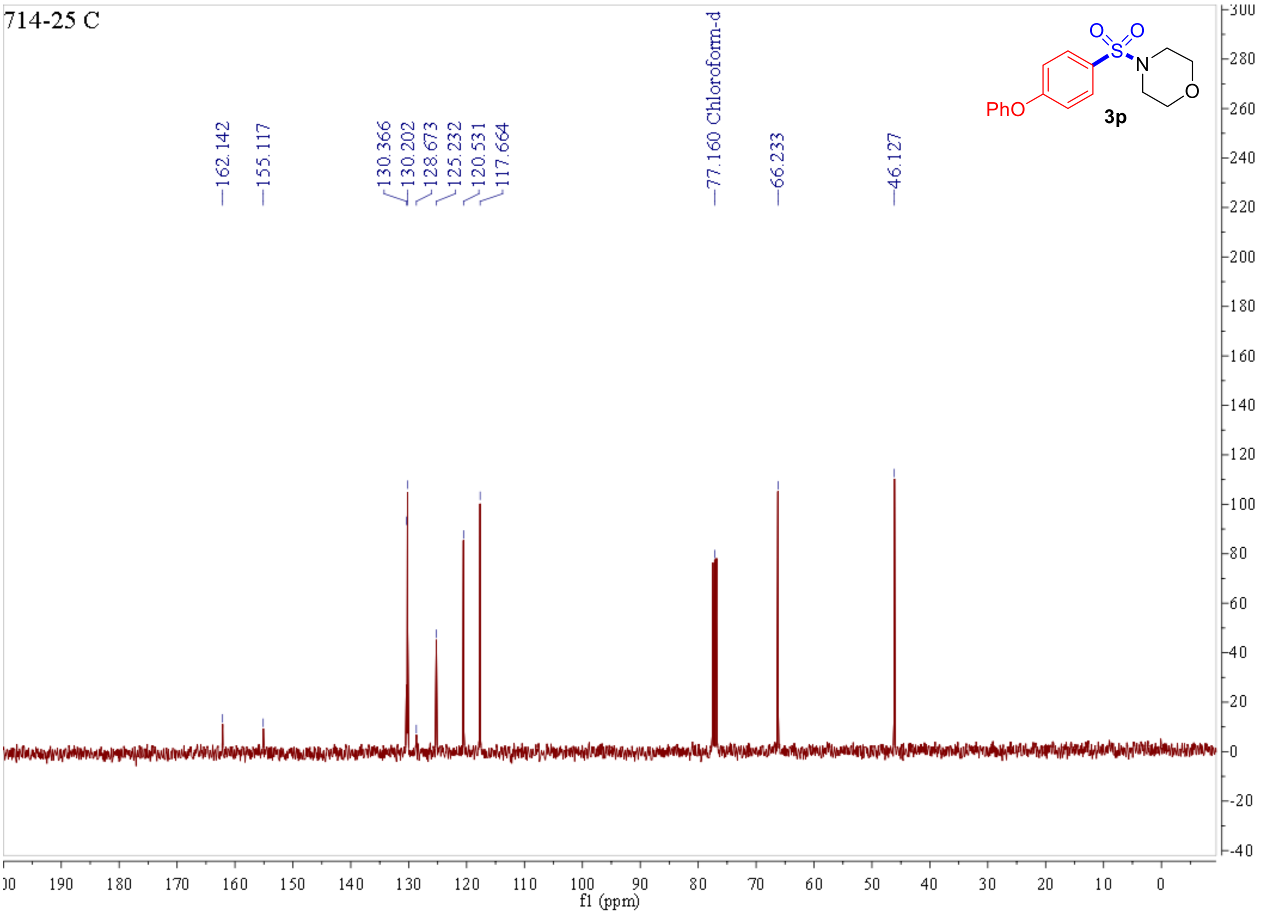
714-25 C
162.142
155.117
130.366
130.202
128.673
125.232
120.531
117.664
77.160 Chloroform-d
66.233
46.127
PhO
O
S
O
N
O
3p
f1 (ppm)

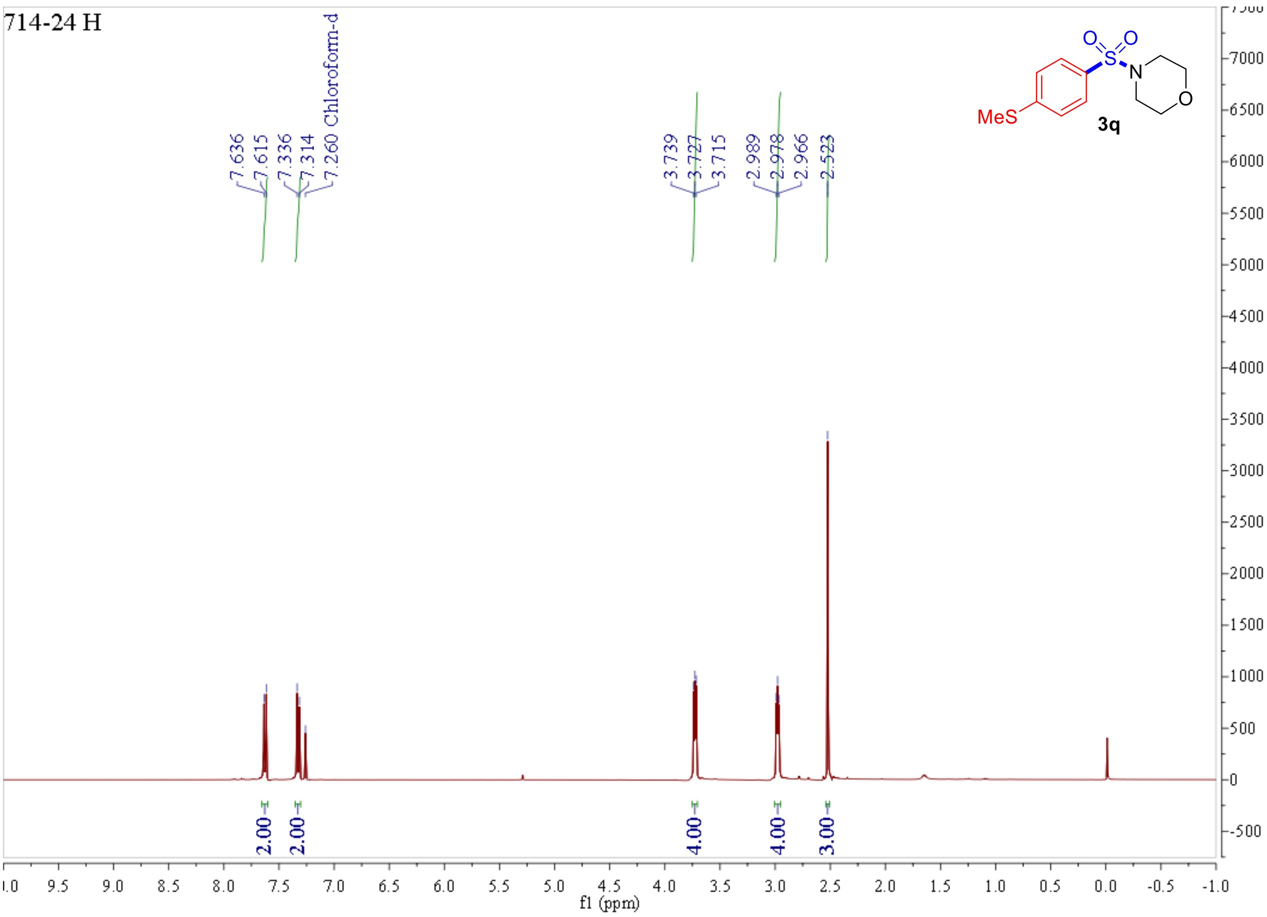
714-24 H
7.636
7.615
7.336
7.314
7.260 Chloroform-d
3.739
3.727
3.715
2.989
2.978
2.966
2.523
MeS
O
S
O
N
O
3q
2.00
2.00
4.00
4.00
3.00
f1 (ppm)

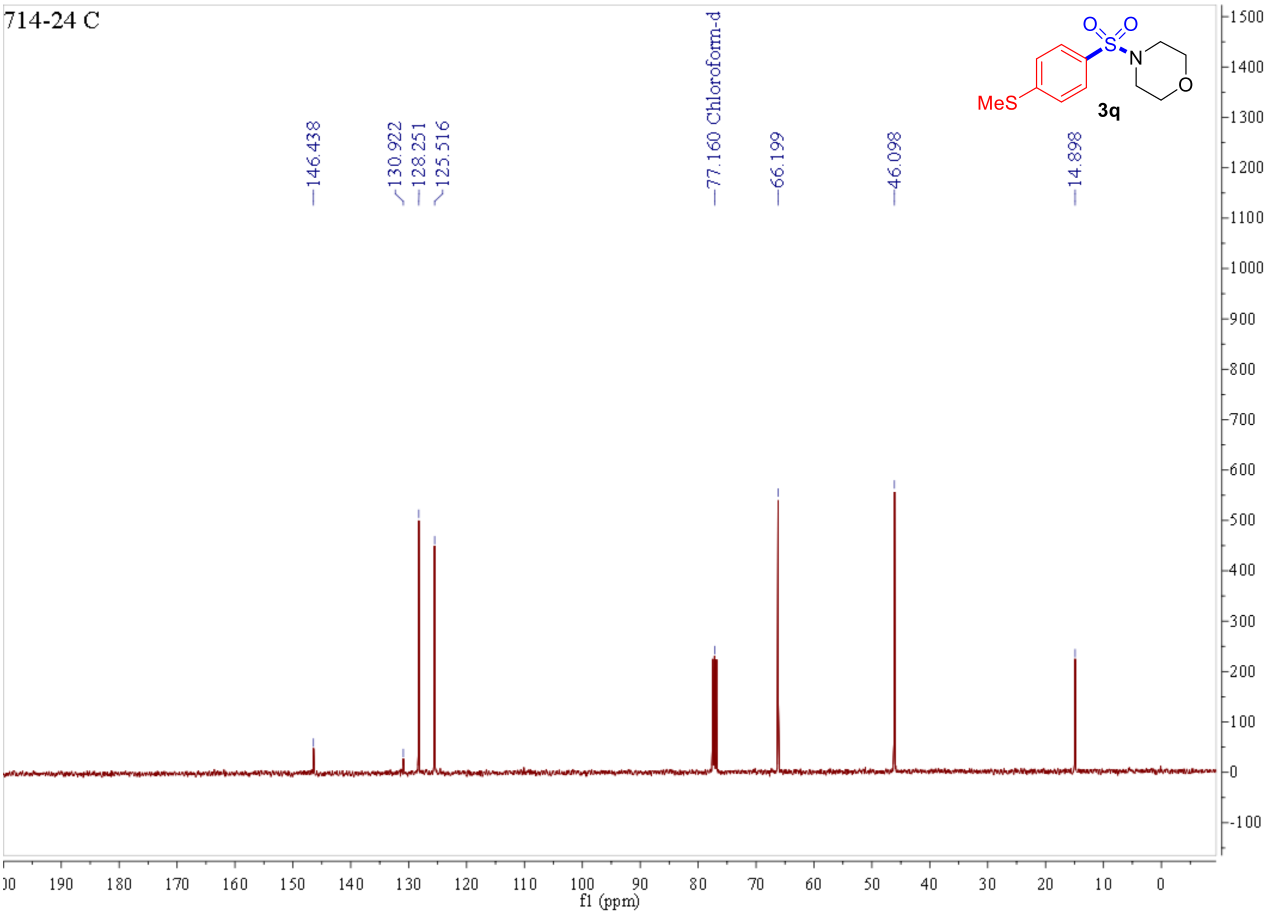
714-24 C
146.438
130.922
128.251
125.516
77.160 Chloroform-d
66.199
46.098
14.898
MeS
O
S
O
N
O
3q
f1 (ppm)

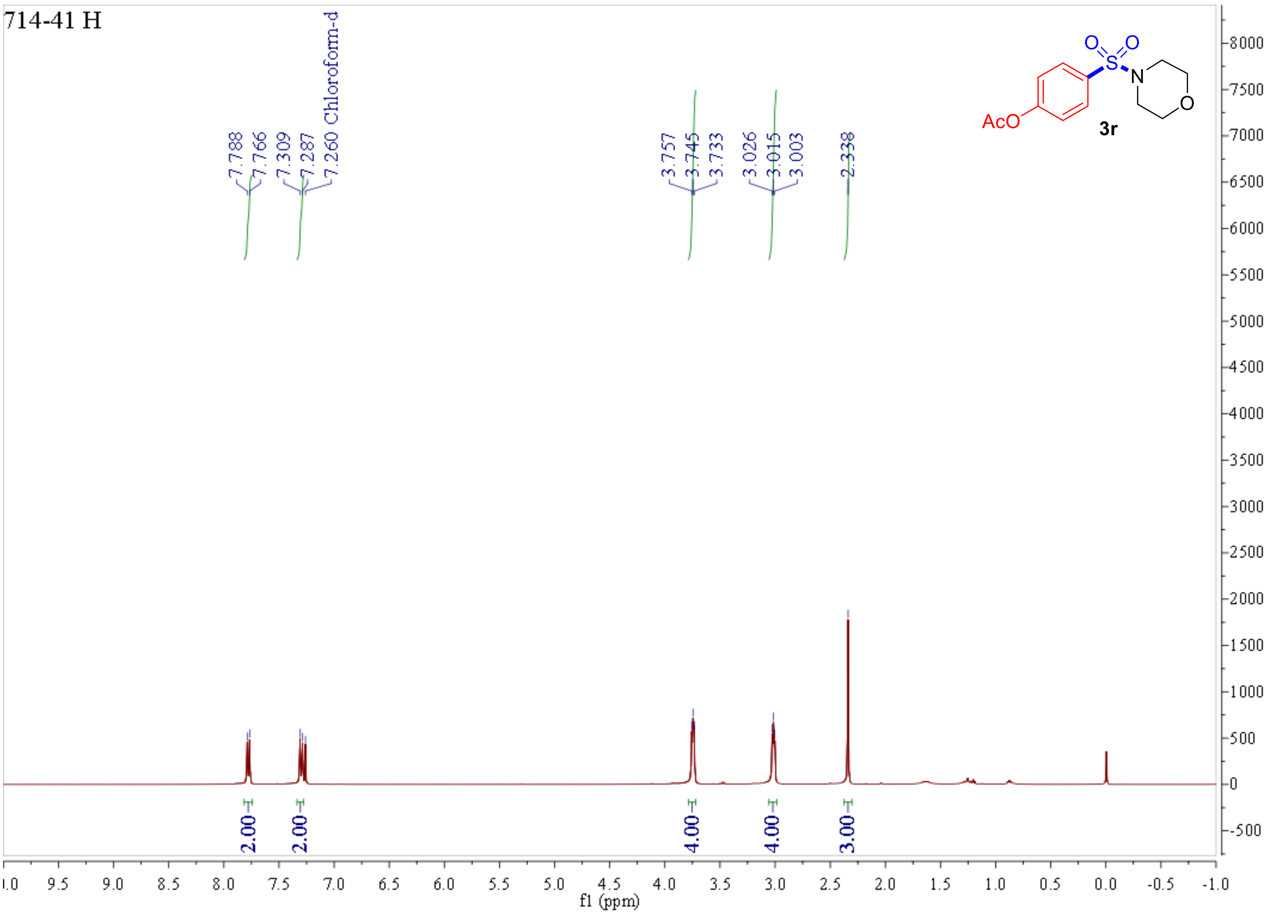
714-41 H
7.788
7.766
7.309
7.287
7.260 Chloroform-d
3.757
3.745
3.733
3.026
3.015
3.003
2.338
AcO
O
O
S
N
O
3r
2.00
2.00
4.00
4.00
3.00
f1 (ppm)

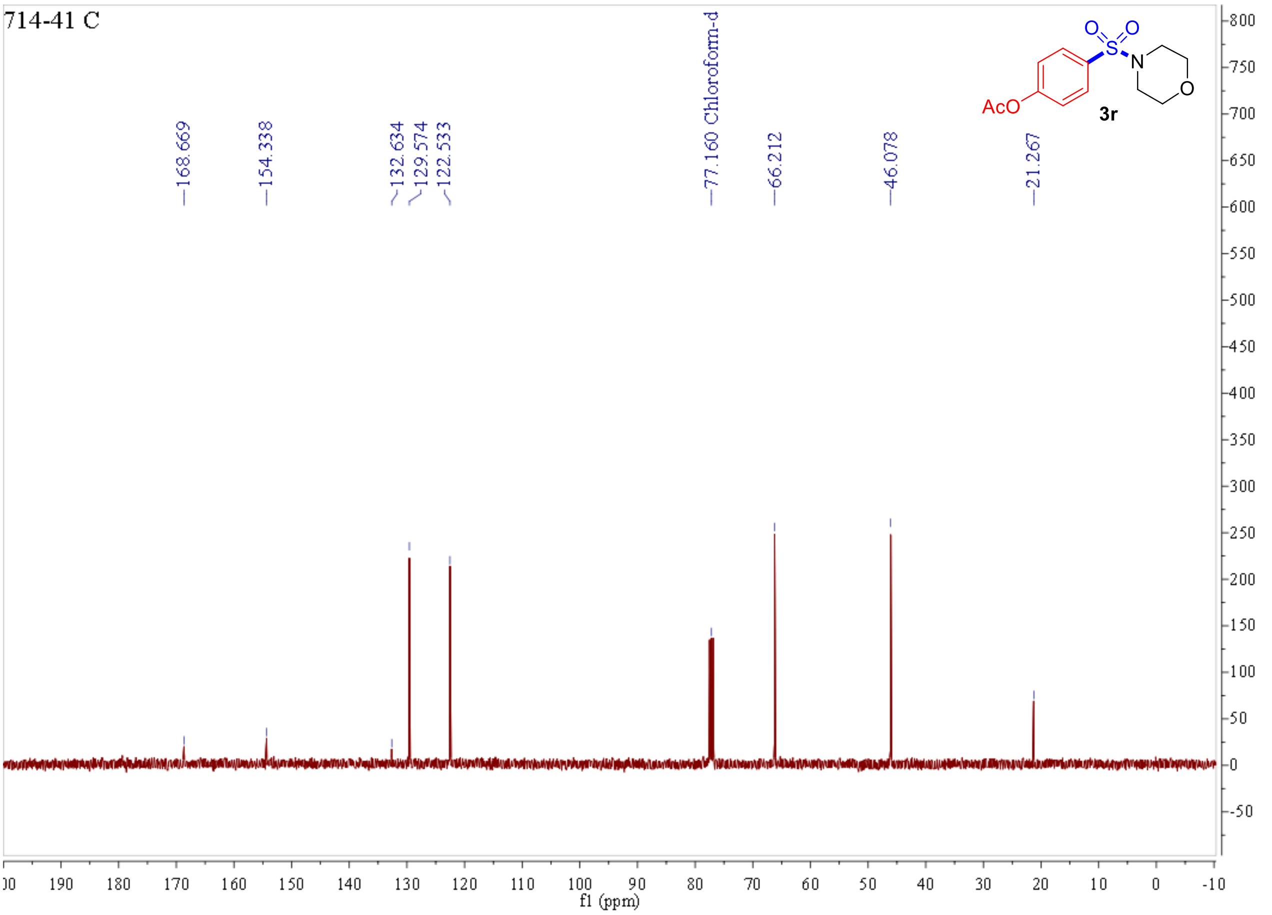
714-41 C
168.669
154.338
132.634
129.574
122.533
77.160 Chloroform-d
66.212
46.078
21.267
AcO
O
O
S
N
O
3r
f1 (ppm)

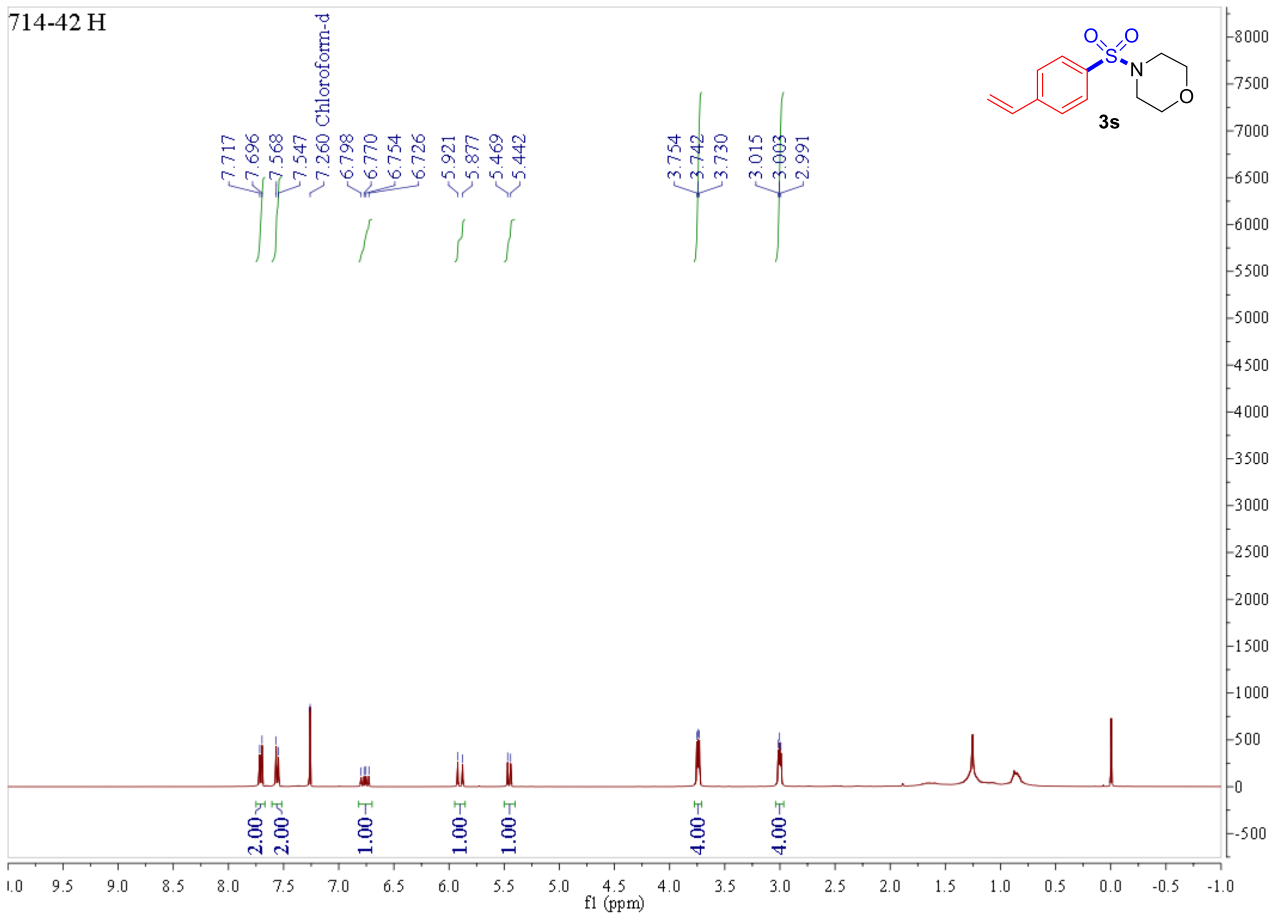
714-42 H
7.717
7.696
7.568
7.547
7.260 Chloroform-d
6.798
6.770
6.754
6.726
5.921
5.877
5.469
5.442
3.754
3.742
3.730
3.015
3.003
2.991
3s
2.00
2.00
1.00
1.00
1.00
4.00
4.00
f1 (ppm)

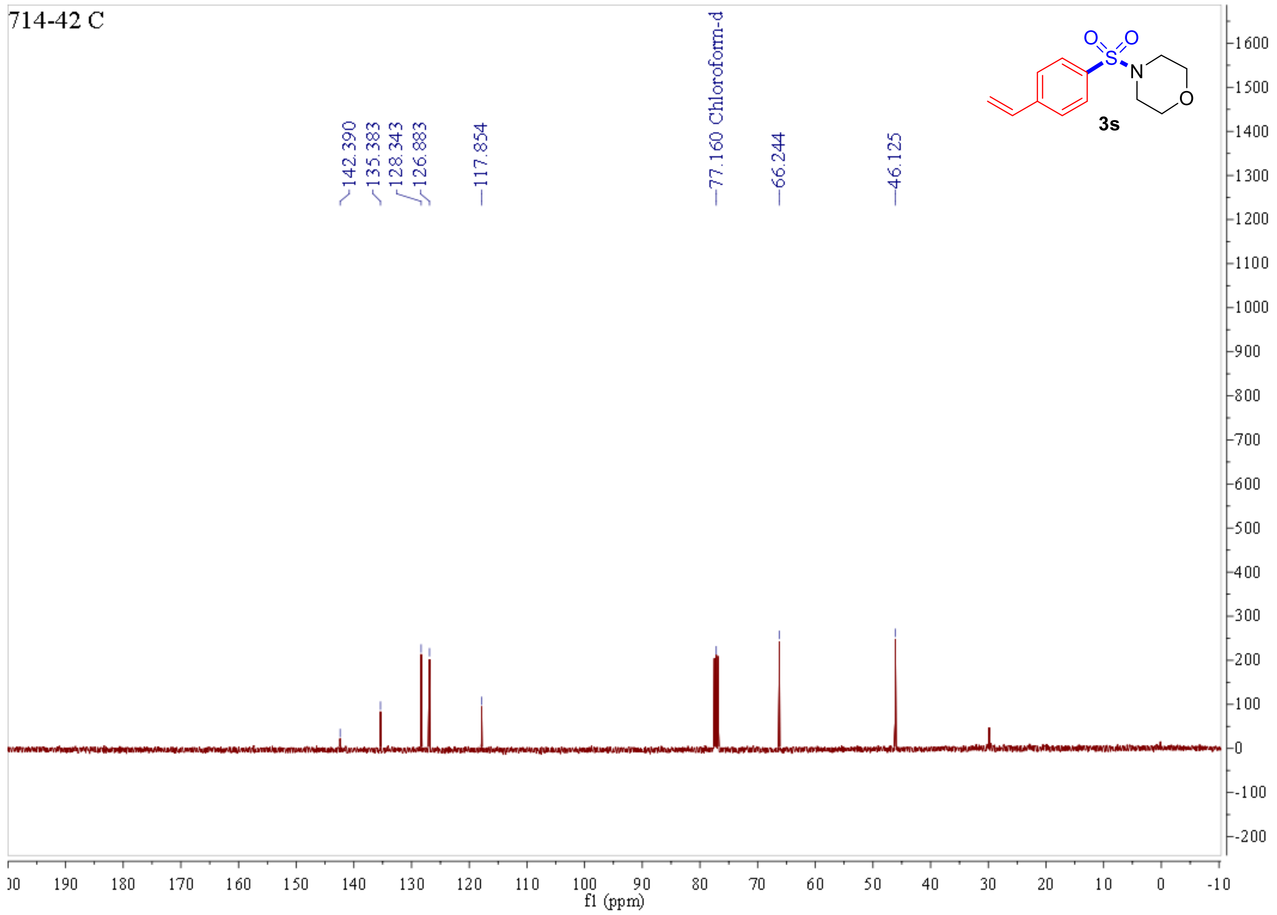
714-42 C
142.390
135.383
128.343
126.883
117.854
77.160 Chloroform-d
66.244
46.125
3s
f1 (ppm)

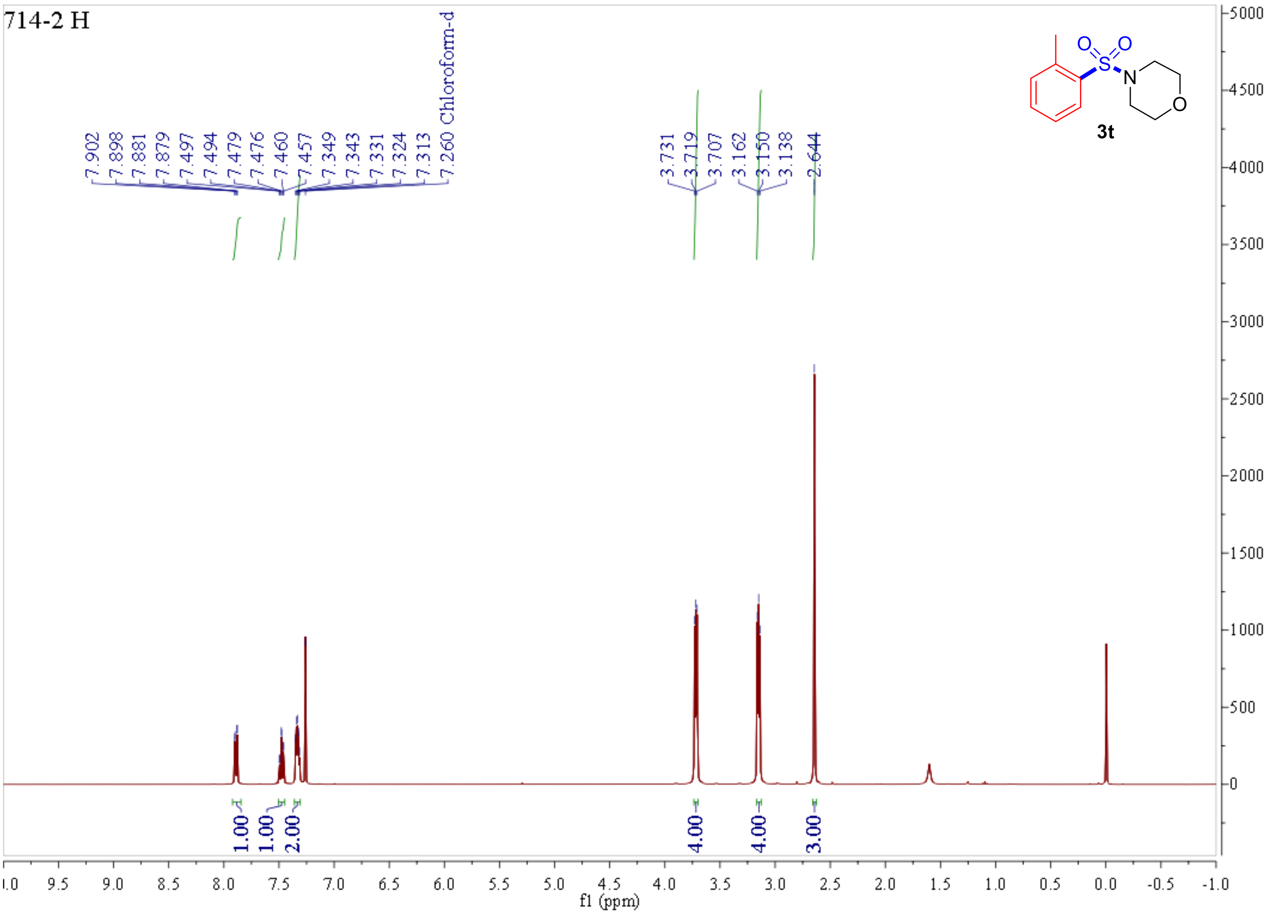
714-2 H
7.902
7.898
7.881
7.879
7.497
7.494
7.479
7.476
7.460
7.457
7.349
7.343
7.331
7.324
7.313
7.260 Chloroform-d
3.731
3.719
3.707
3.162
3.150
3.138
2.644
1.00
1.00
2.00
4.00
4.00
3.00
3t
f1 (ppm)

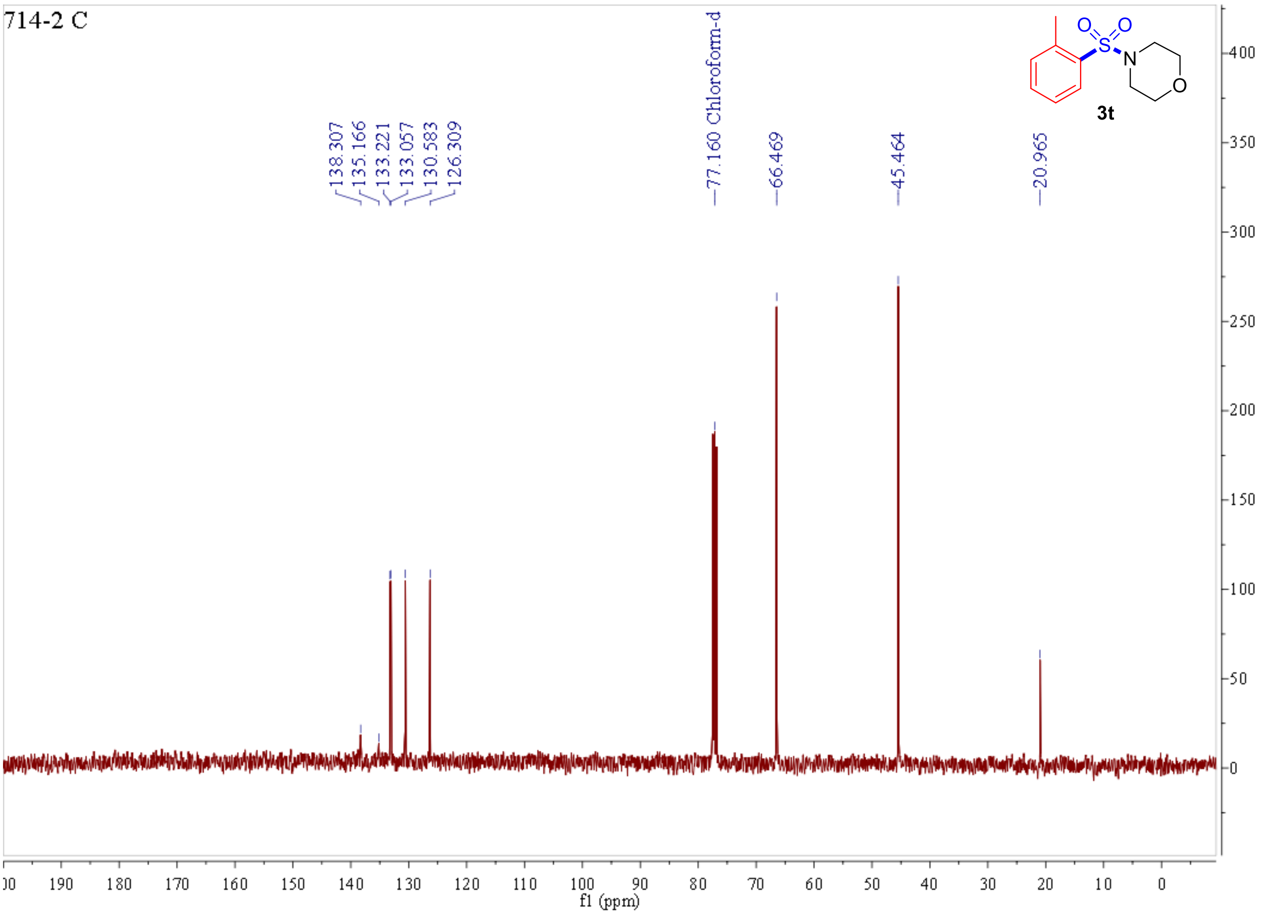
714-2 C
138.307
135.166
133.221
133.057
130.583
126.309
77.160 Chloroform-d
66.469
45.464
20.965
3t
f1 (ppm)

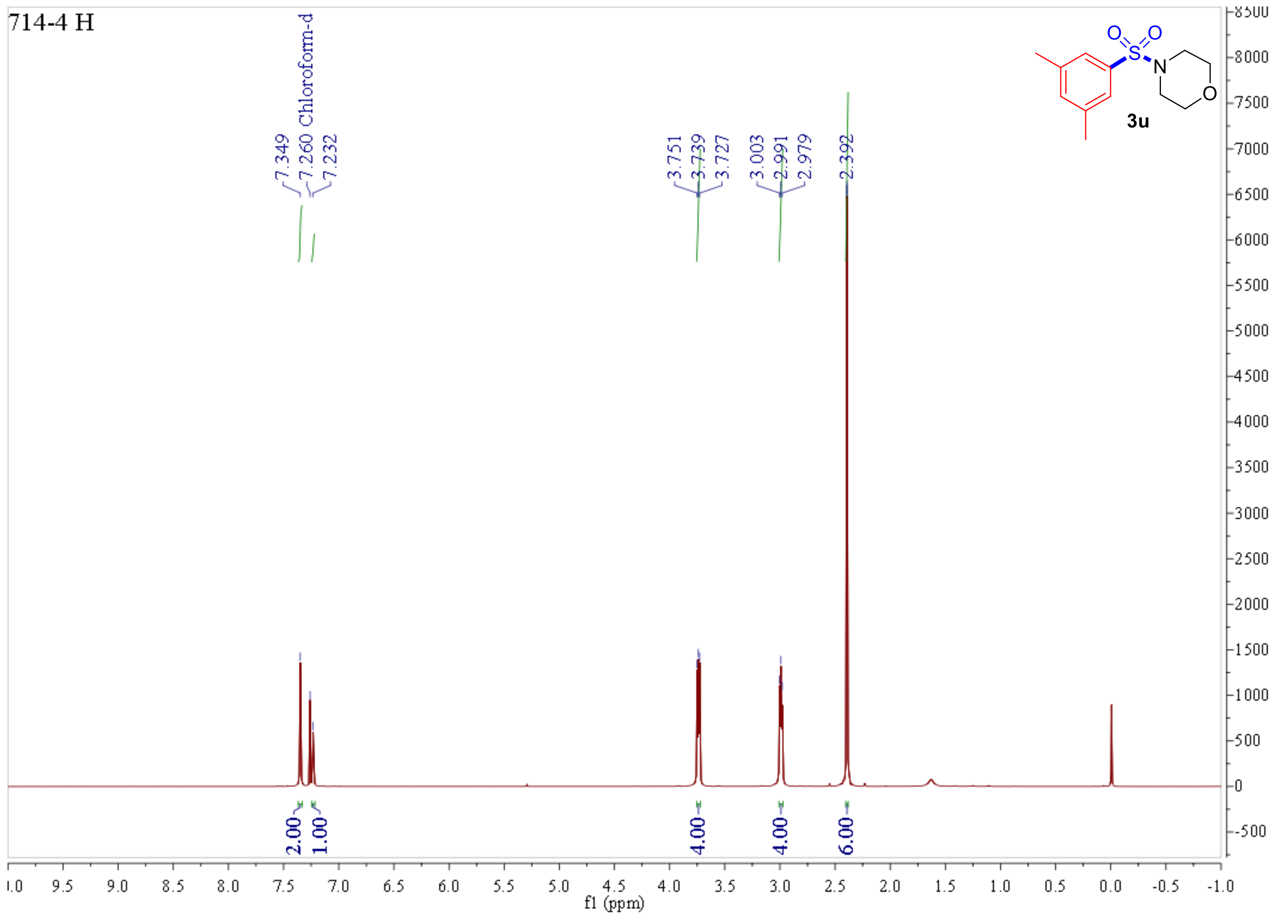
714-4 H
7.349
7.260 Chloroform-d
7.232
3.751
3.739
3.727
3.003
2.991
2.979
2.392
2.00
1.00
4.00
4.00
6.00
f1 (ppm)
3u

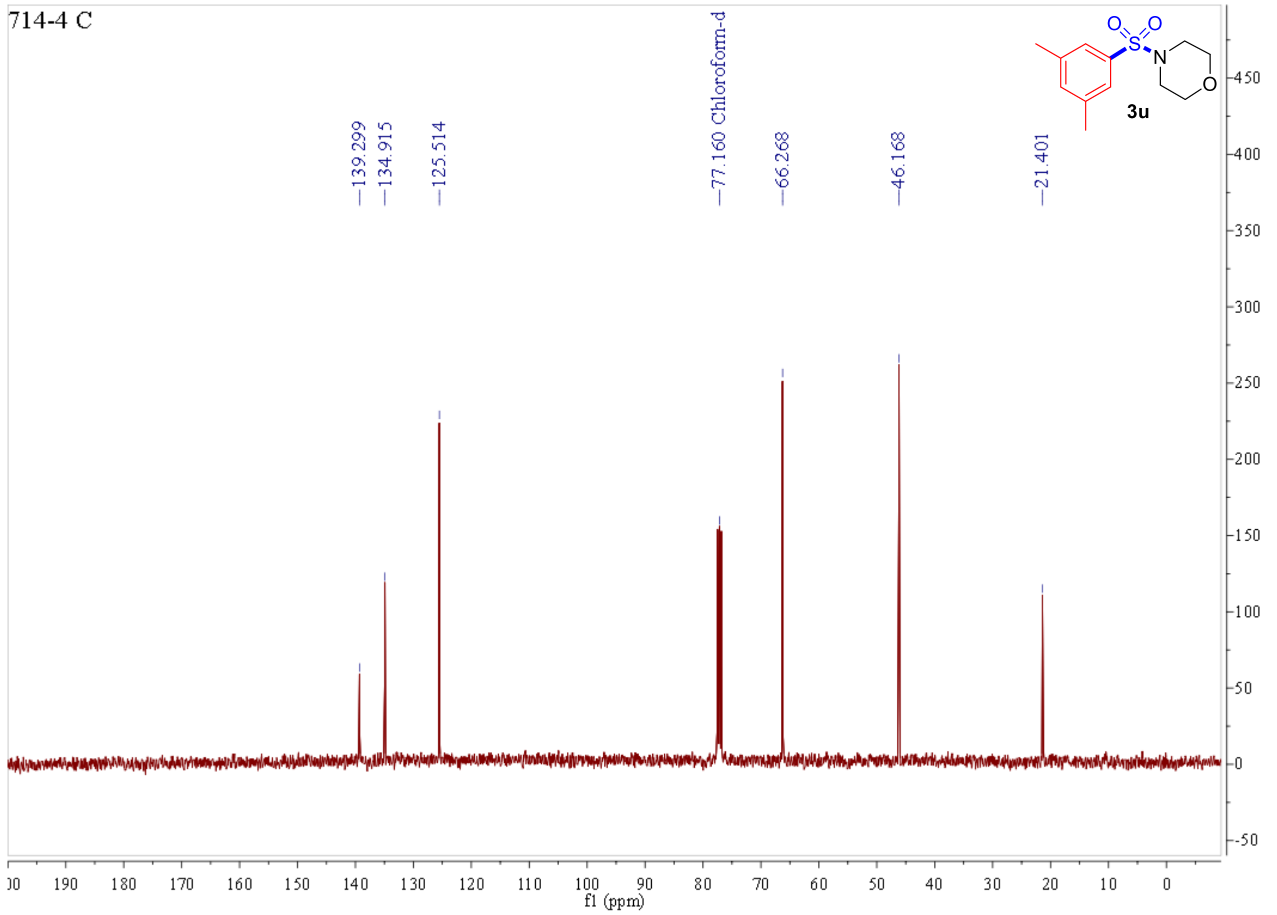
714-4 C
139.299
134.915
125.514
77.160 Chloroform-d
66.268
46.168
21.401
f1 (ppm)
3u

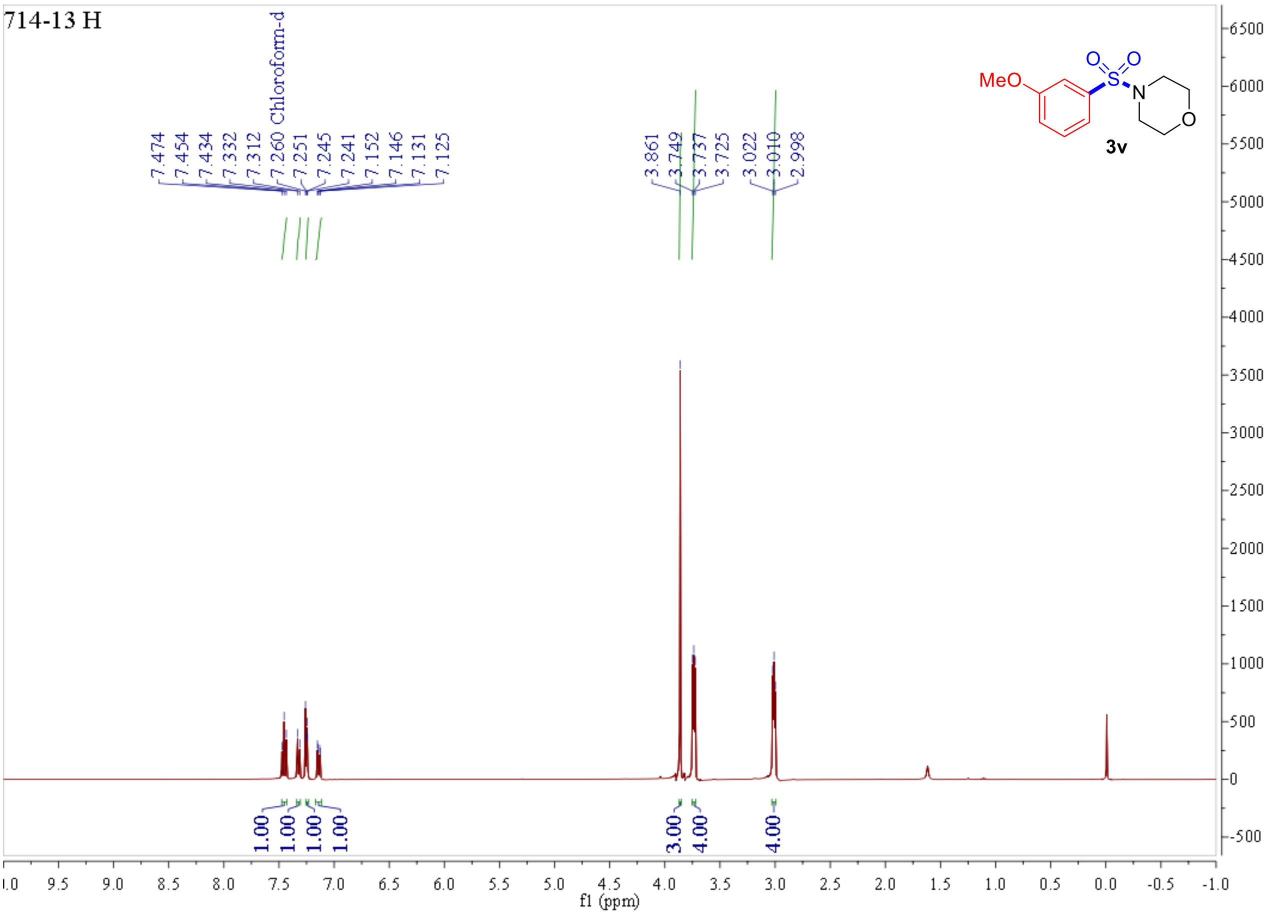
714-13 H
7.474
7.454
7.434
7.332
7.312
7.260 Chloroform-d
7.251
7.245
7.241
7.152
7.146
7.131
7.125
3.861
3.749
3.737
3.725
3.022
3.010
2.998
MeO
O
S
O
N
O
3v
1.00
1.00
1.00
1.00
3.00
4.00
4.00
f1 (ppm)

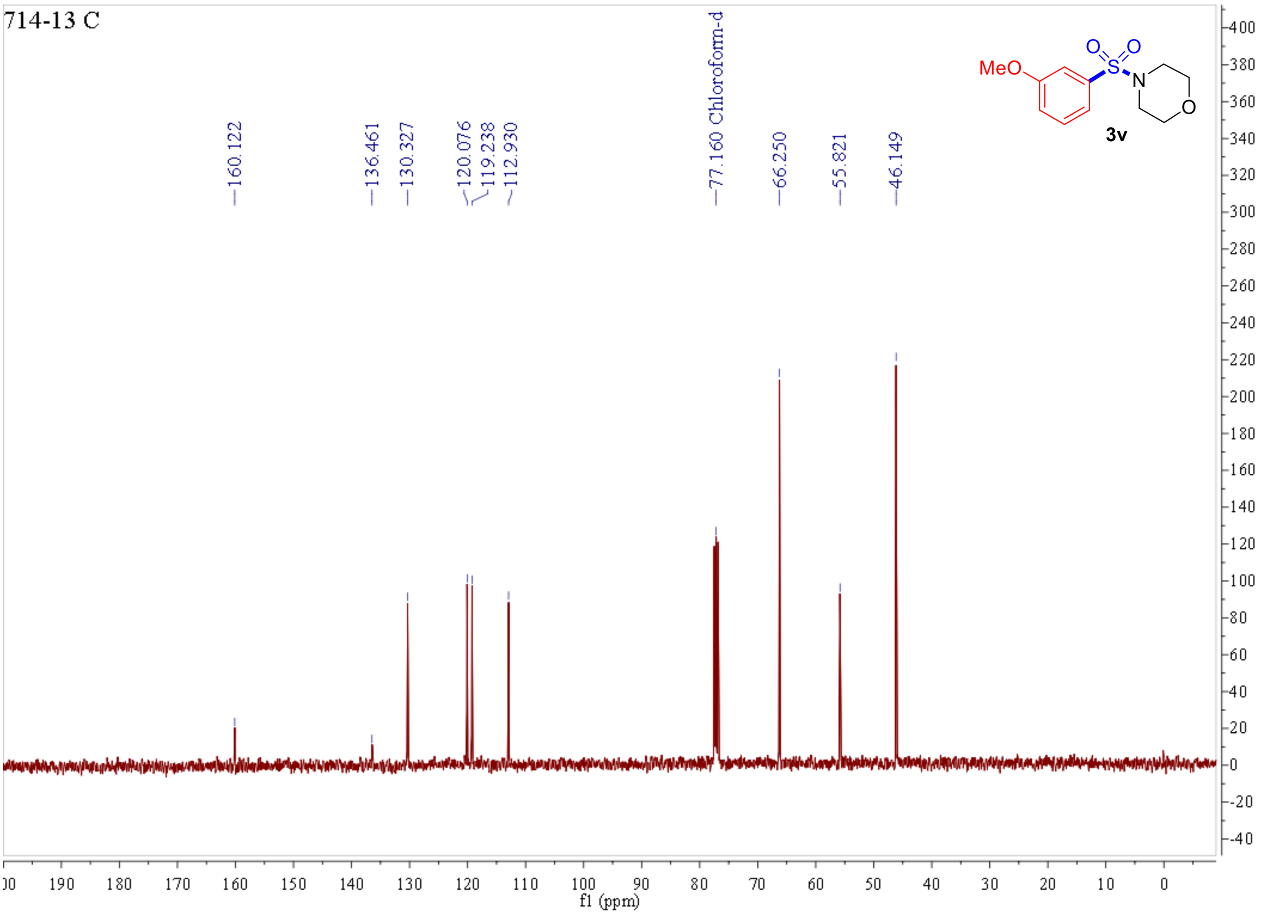
714-13 C
160.122
136.461
130.327
120.076
119.238
112.930
77.160 Chloroform-d
66.250
55.821
46.149
MeO
O
S
O
N
O
3v
f1 (ppm)

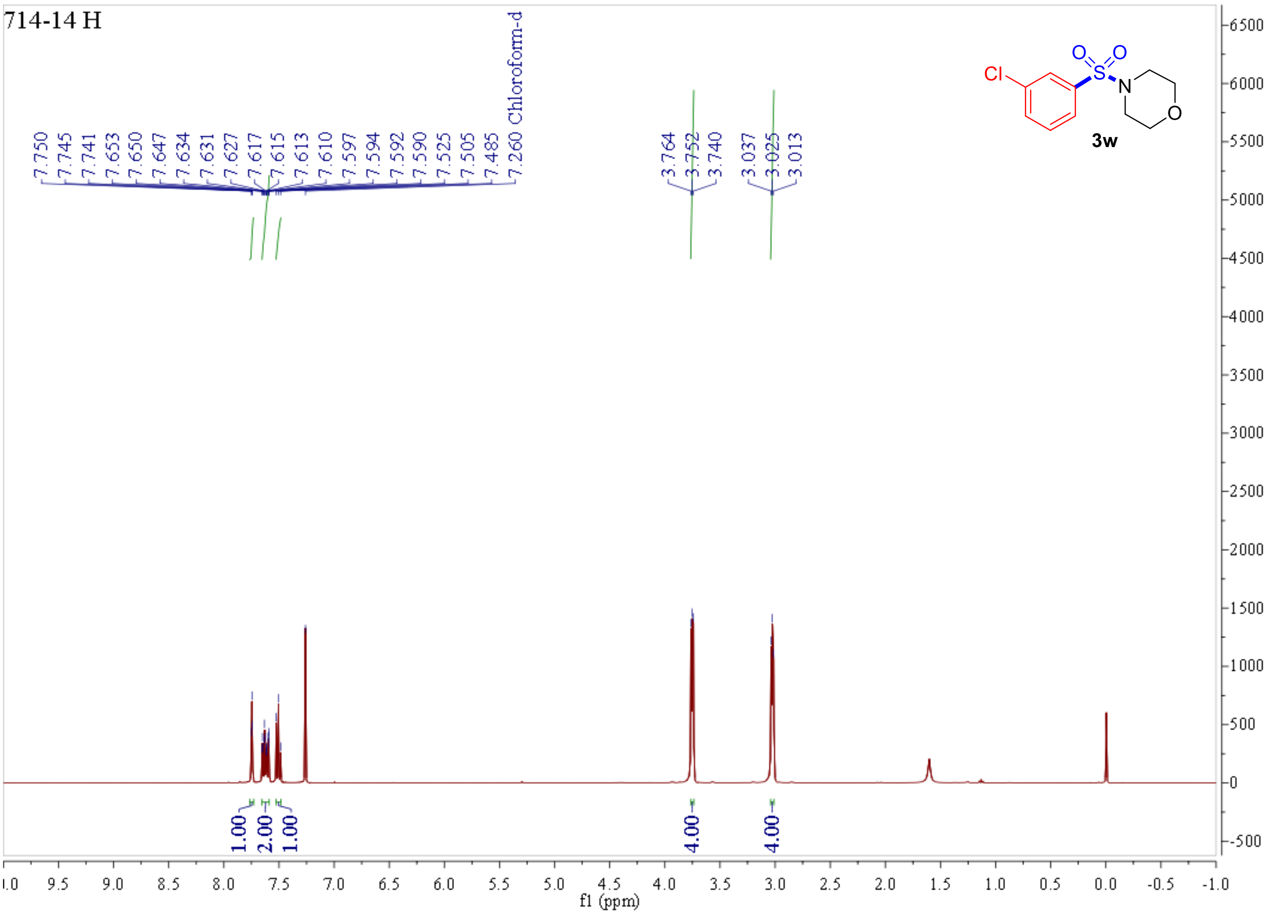
714-14 H
7.750
7.745
7.741
7.653
7.650
7.647
7.634
7.631
7.627
7.617
7.615
7.613
7.610
7.597
7.594
7.592
7.590
7.525
7.505
7.485
7.260 Chloroform-d
3.764
3.752
3.740
3.037
3.025
3.013
3w
1.00
2.00
1.00
4.00
4.00
f1 (ppm)

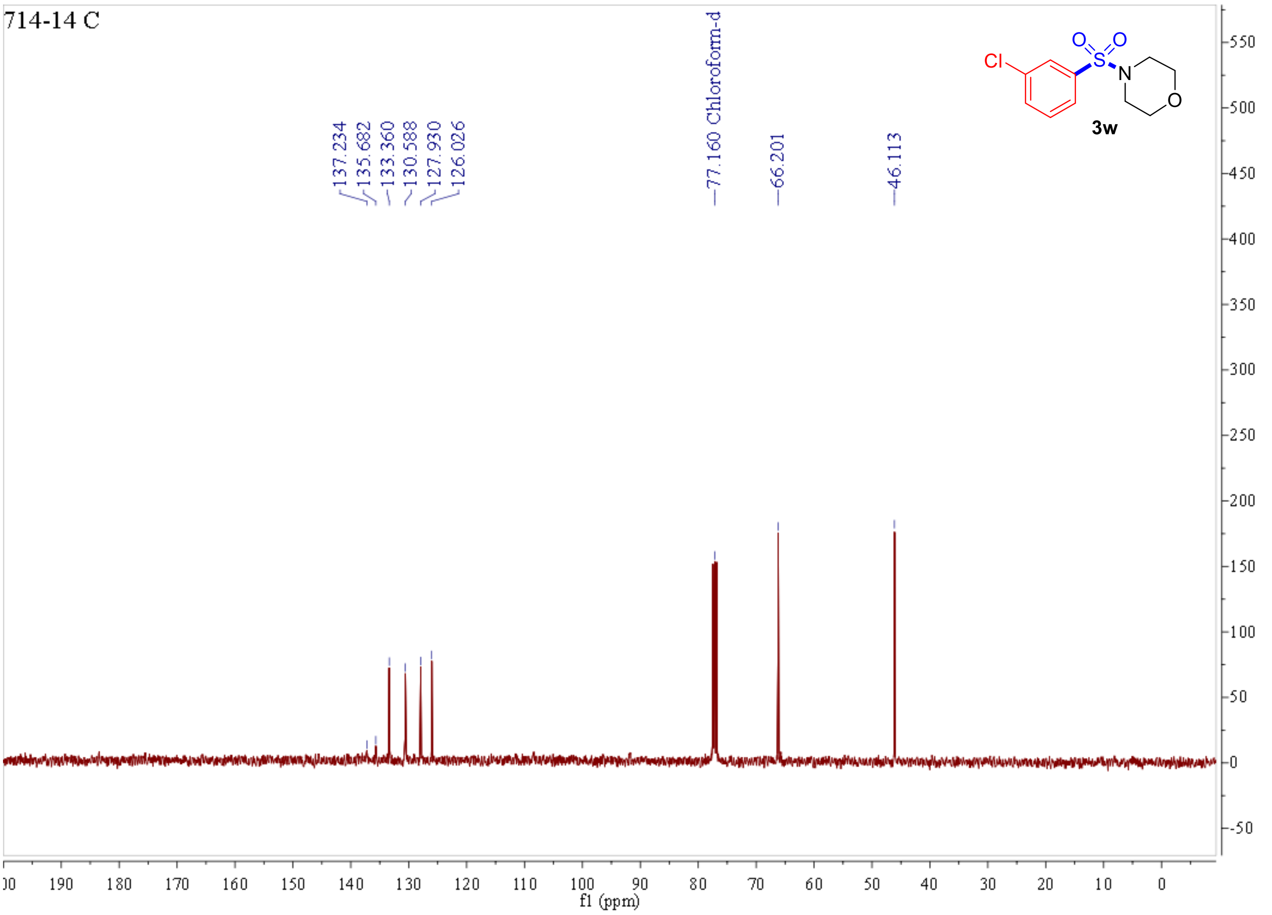
714-14 C
137.234
135.682
133.360
130.588
127.930
126.026
77.160 Chloroform-d
66.201
46.113
3w
f1 (ppm)

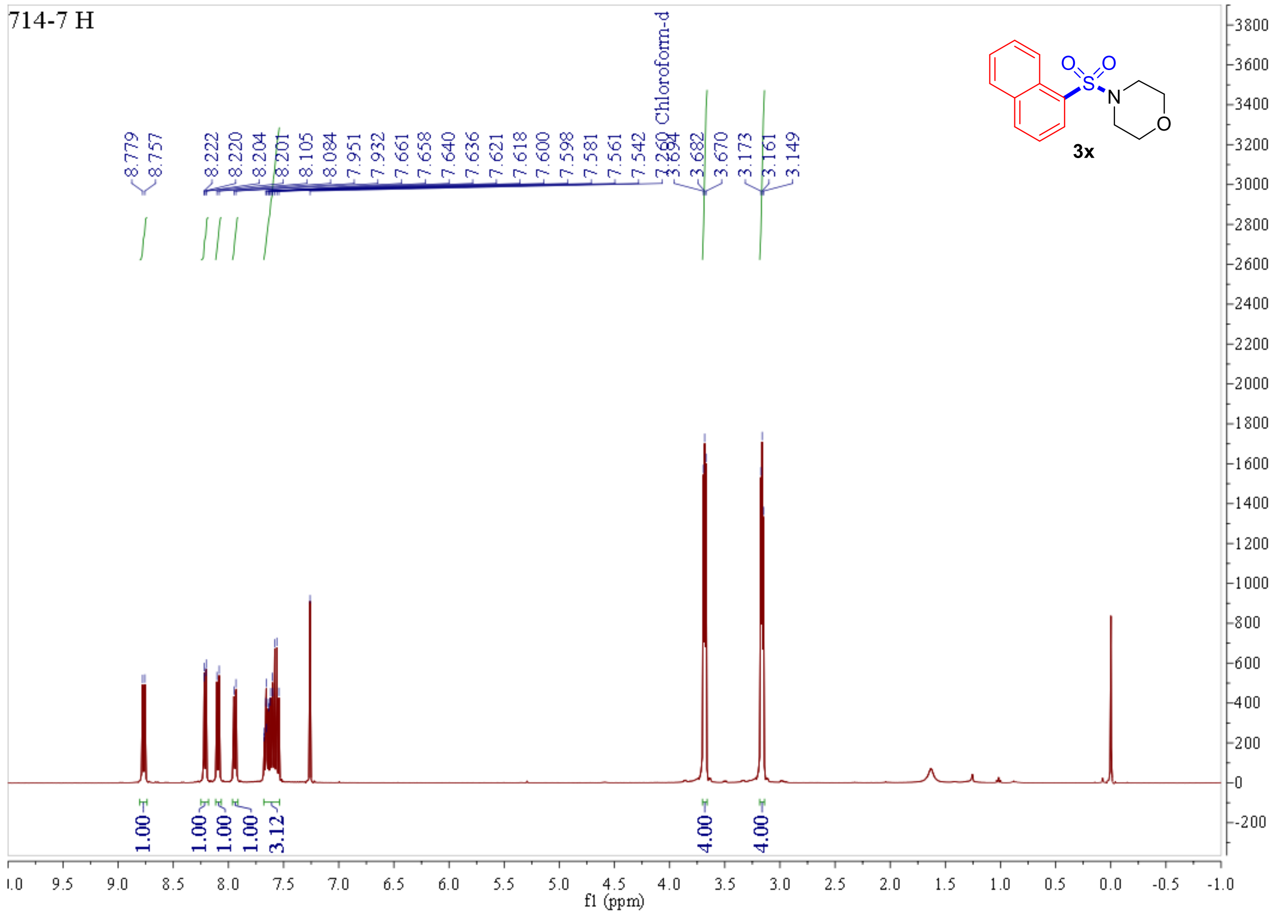
714-7 H
3x
8.779
8.757
8.222
8.220
8.204
8.201
8.105
8.084
7.951
7.932
7.661
7.658
7.640
7.636
7.621
7.618
7.600
7.598
7.581
7.561
7.542
7.260 Chloroform-d
3.694
3.682
3.670
3.173
3.161
3.149
1.00
1.00
1.00
1.00
3.12
4.00
4.00
f1 (ppm)

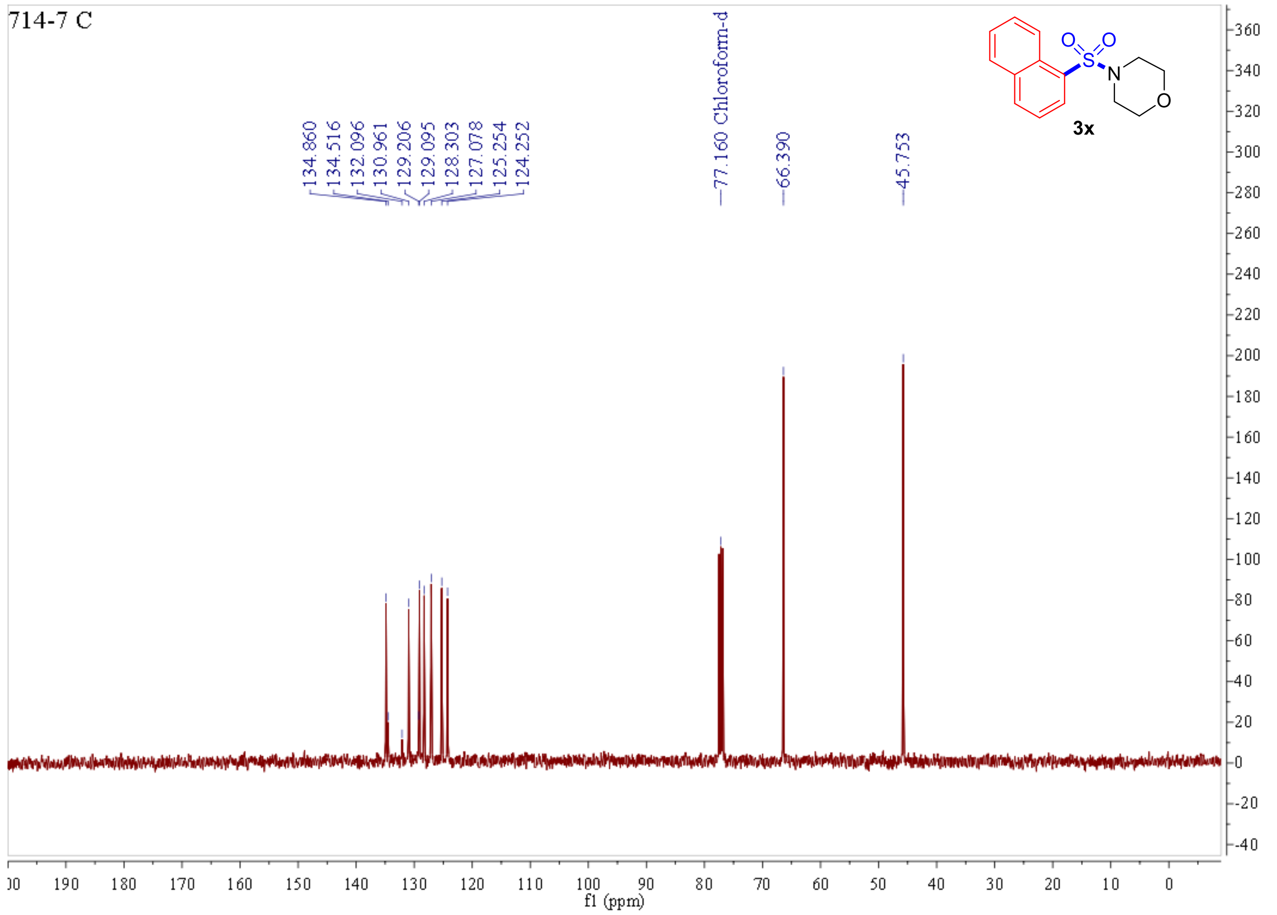
714-7 C
3x
134.860
134.516
132.096
130.961
129.206
129.095
128.303
127.078
125.254
124.252
77.160 Chloroform-d
66.390
45.753
f1 (ppm)

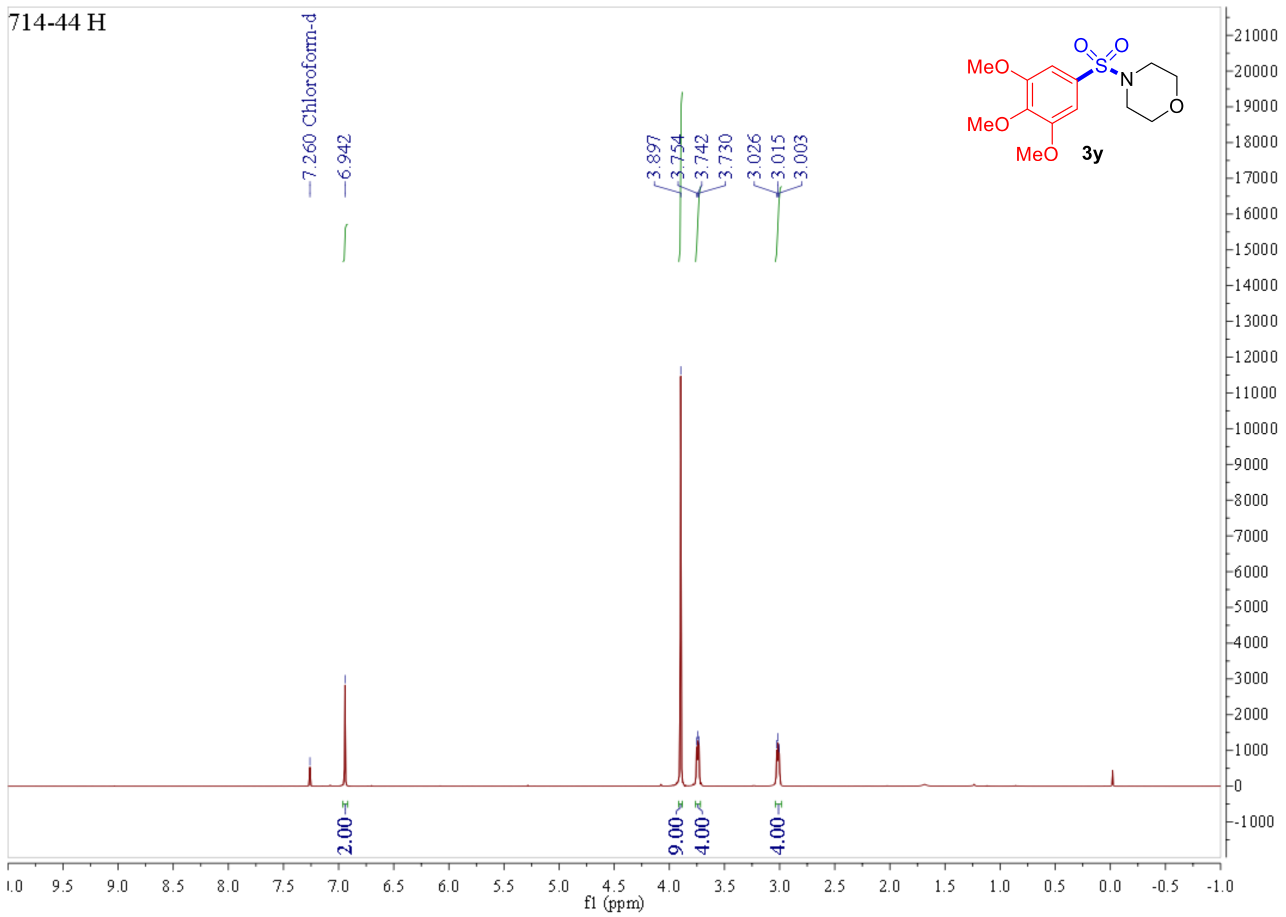
714-44 H
7.260 Chloroform-d
6.942
3.897
3.754
3.742
3.730
3.026
3.015
3.003
MeO
MeO
MeO
3y
2.00
9.00
4.00
4.00
f1 (ppm)

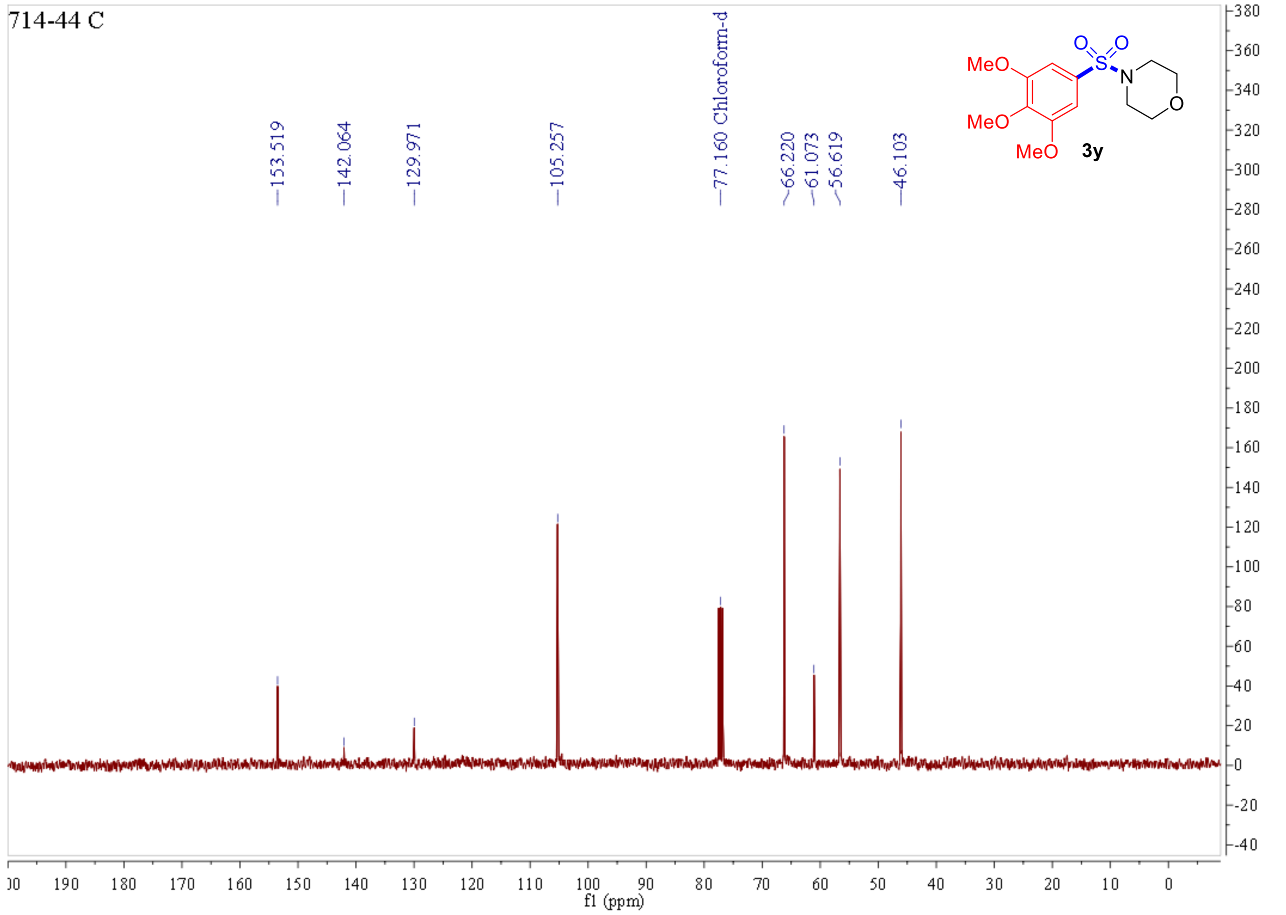
714-44 C
153.519
142.064
129.971
105.257
77.160 Chloroform-d
66.220
61.073
56.619
46.103
MeO
MeO
MeO
3y
f1 (ppm)

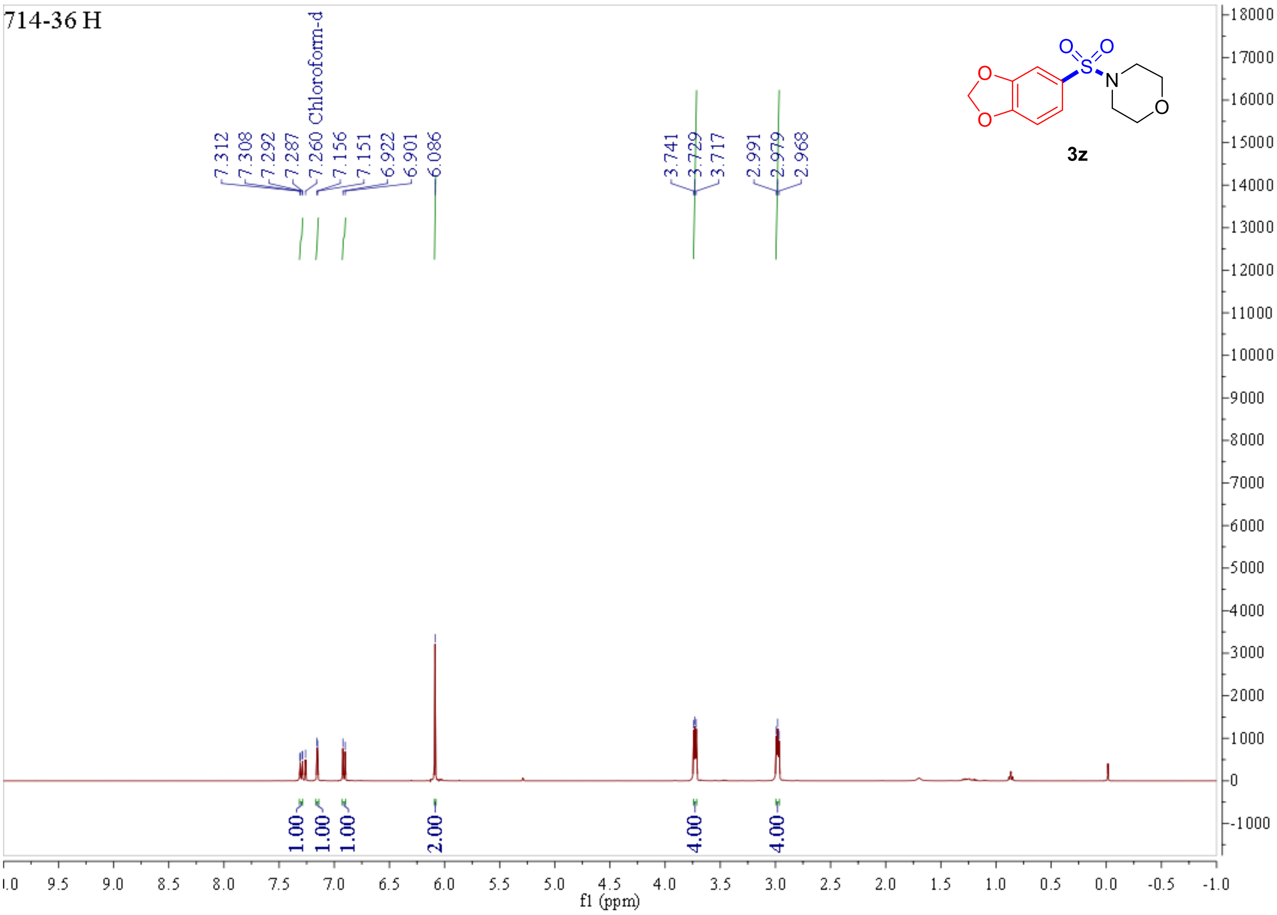

714-36 H
7.312
7.308
7.292
7.287
7.260 Chloroform-d
7.156
7.151
6.922
6.901
6.086
3.741
3.729
3.717
2.991
2.979
2.968
3z
1.00
1.00
1.00
2.00
4.00
4.00
f1 (ppm)

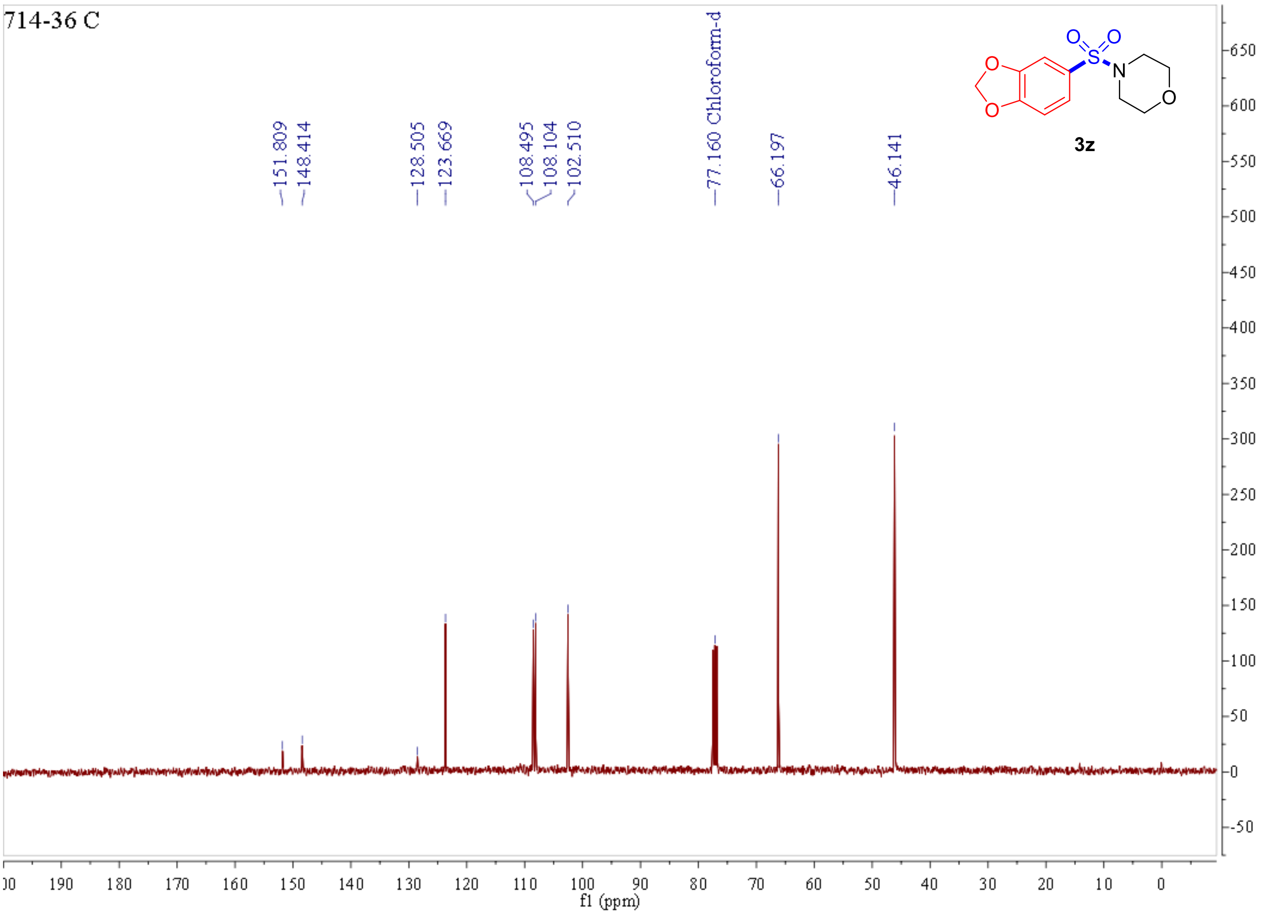

714-36 C
151.809
148.414
128.505
123.669
108.495
108.104
102.510
77.160 Chloroform-d
66.197
46.141
3z
f1 (ppm)

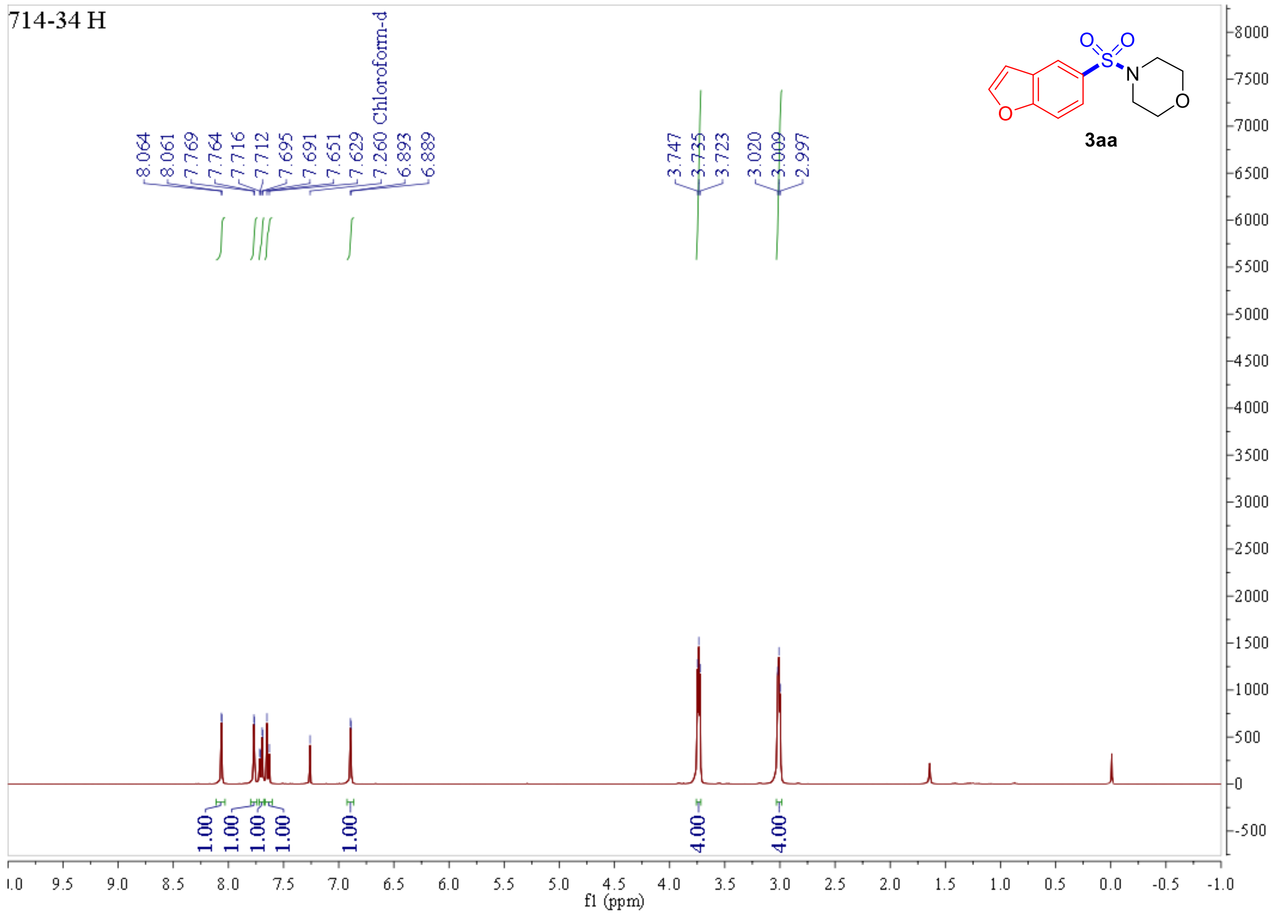
714-34 H
8.064
8.061
7.769
7.764
7.716
7.712
7.695
7.691
7.651
7.629
7.260 Chloroform-d
6.893
6.889
3.747
3.735
3.723
3.020
3.009
2.997
1.00
1.00
1.00
1.00
1.00
4.00
4.00
3aa
f1 (ppm)

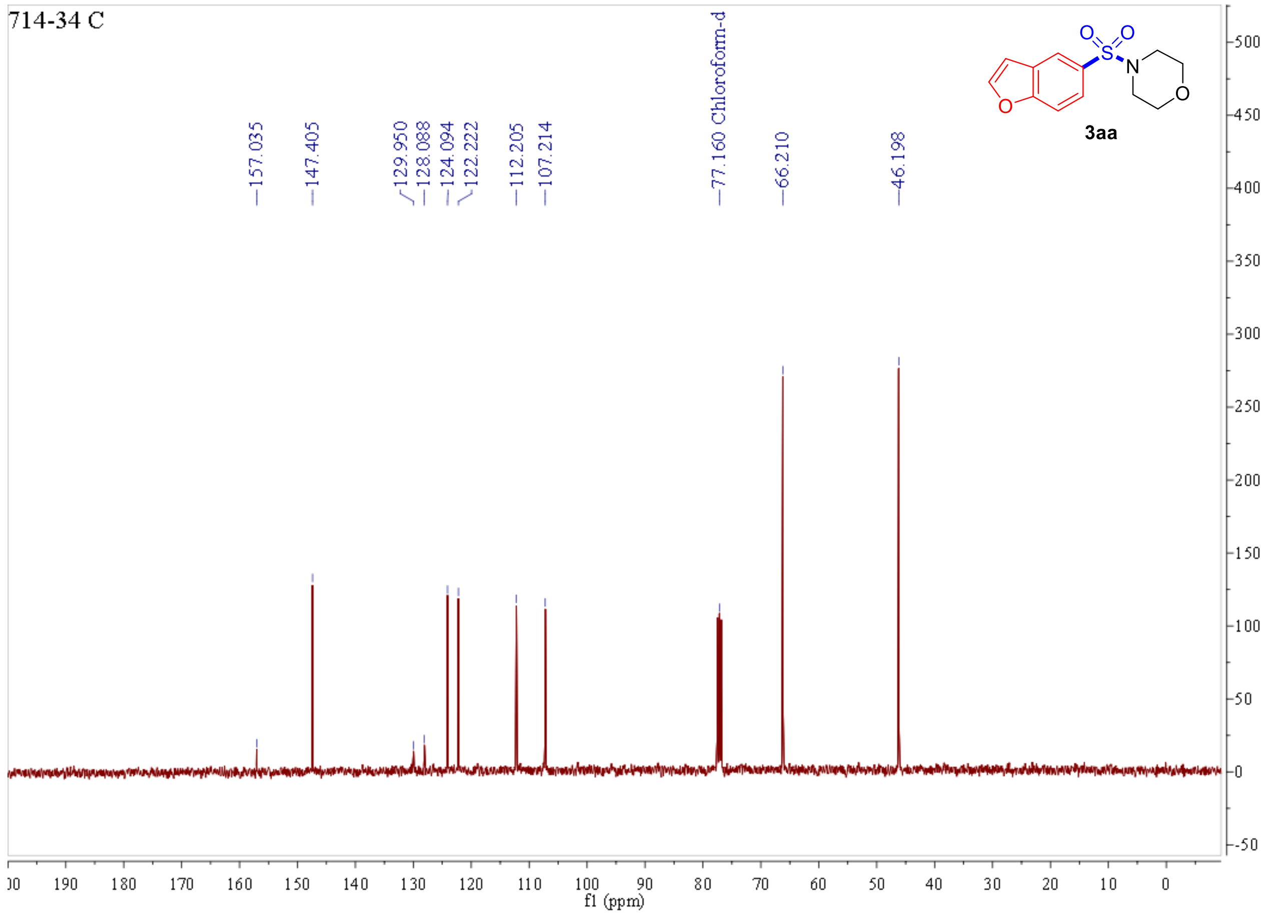
714-34 C
157.035
147.405
129.950
128.088
124.094
122.222
112.205
107.214
77.160 Chloroform-d
66.210
46.198
3aa
f1 (ppm)

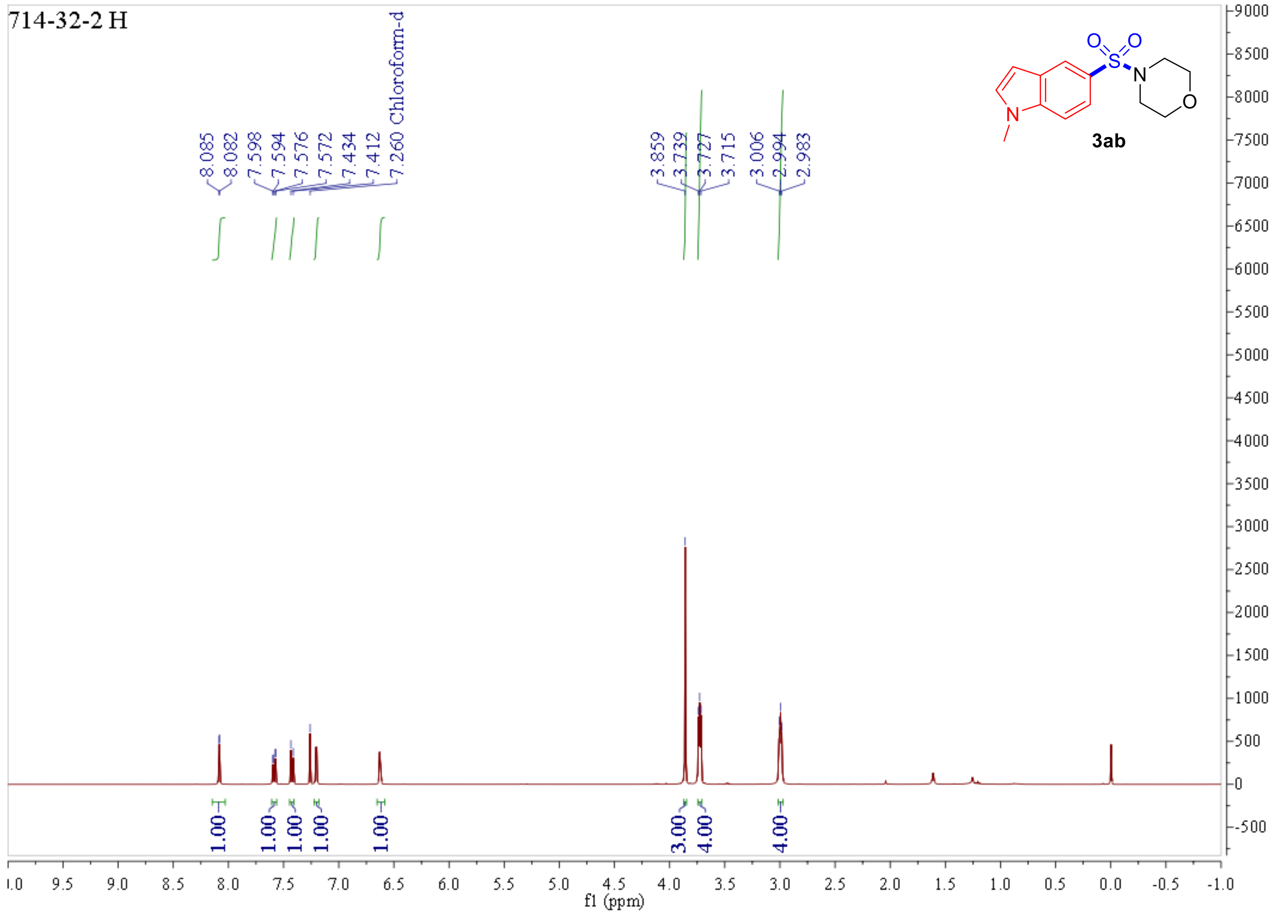
714-32-2 H
8.085
8.082
7.598
7.594
7.576
7.572
7.434
7.412
7.260 Chloroform-d
3.859
3.739
3.727
3.715
3.006
2.994
2.983
1.00
1.00
1.00
1.00
1.00
3.00
4.00
4.00
3ab
f1 (ppm)

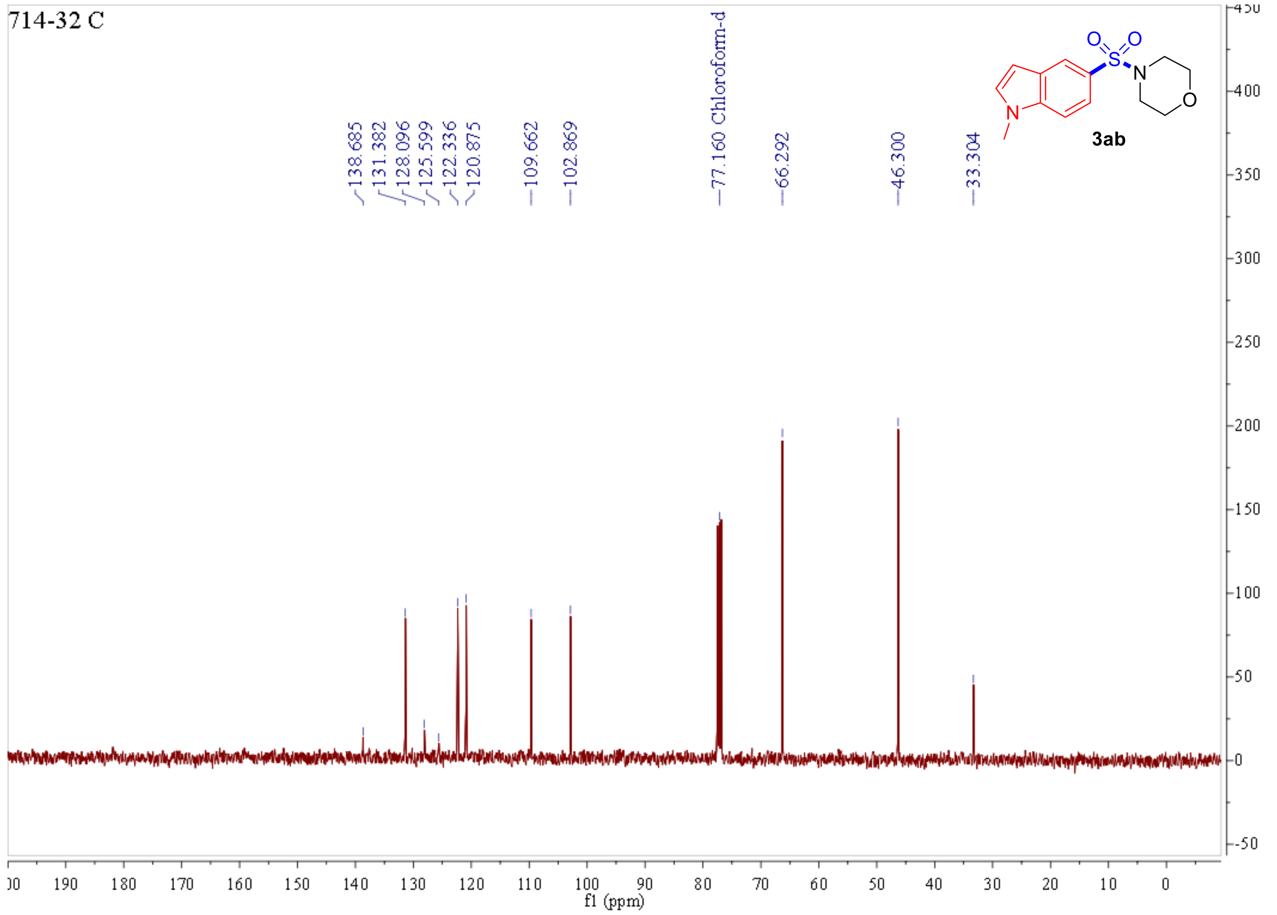
714-32 C
138.685
131.382
128.096
125.599
122.336
120.875
109.662
102.869
77.160 Chloroform-d
66.292
46.300
33.304
3ab
f1 (ppm)

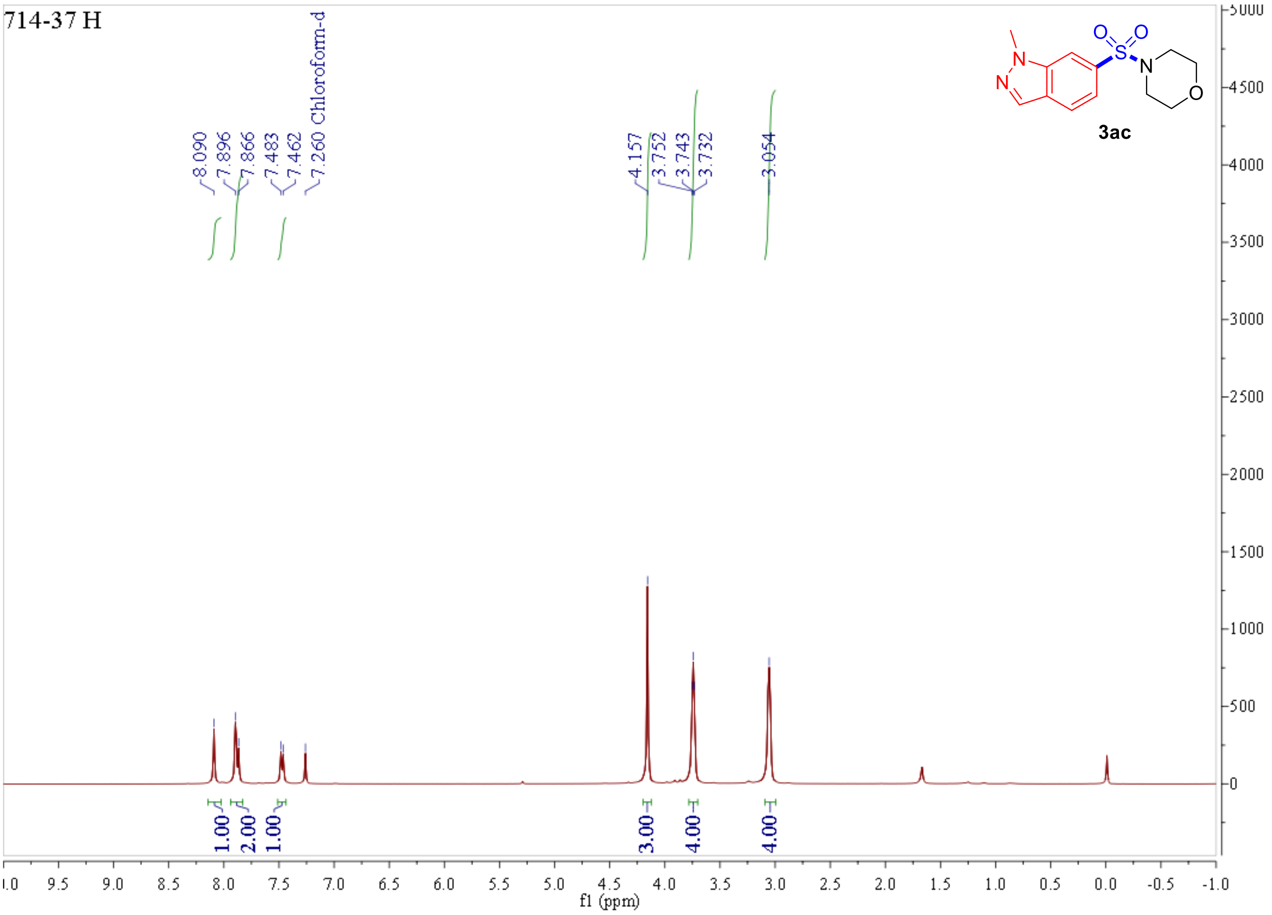
714-37 H
8.090
7.896
7.866
7.483
7.462
7.260 Chloroform-d
4.157
3.752
3.743
3.732
3.054
1.00
2.00
1.00
3.00
4.00
4.00
3ac
f1 (ppm)

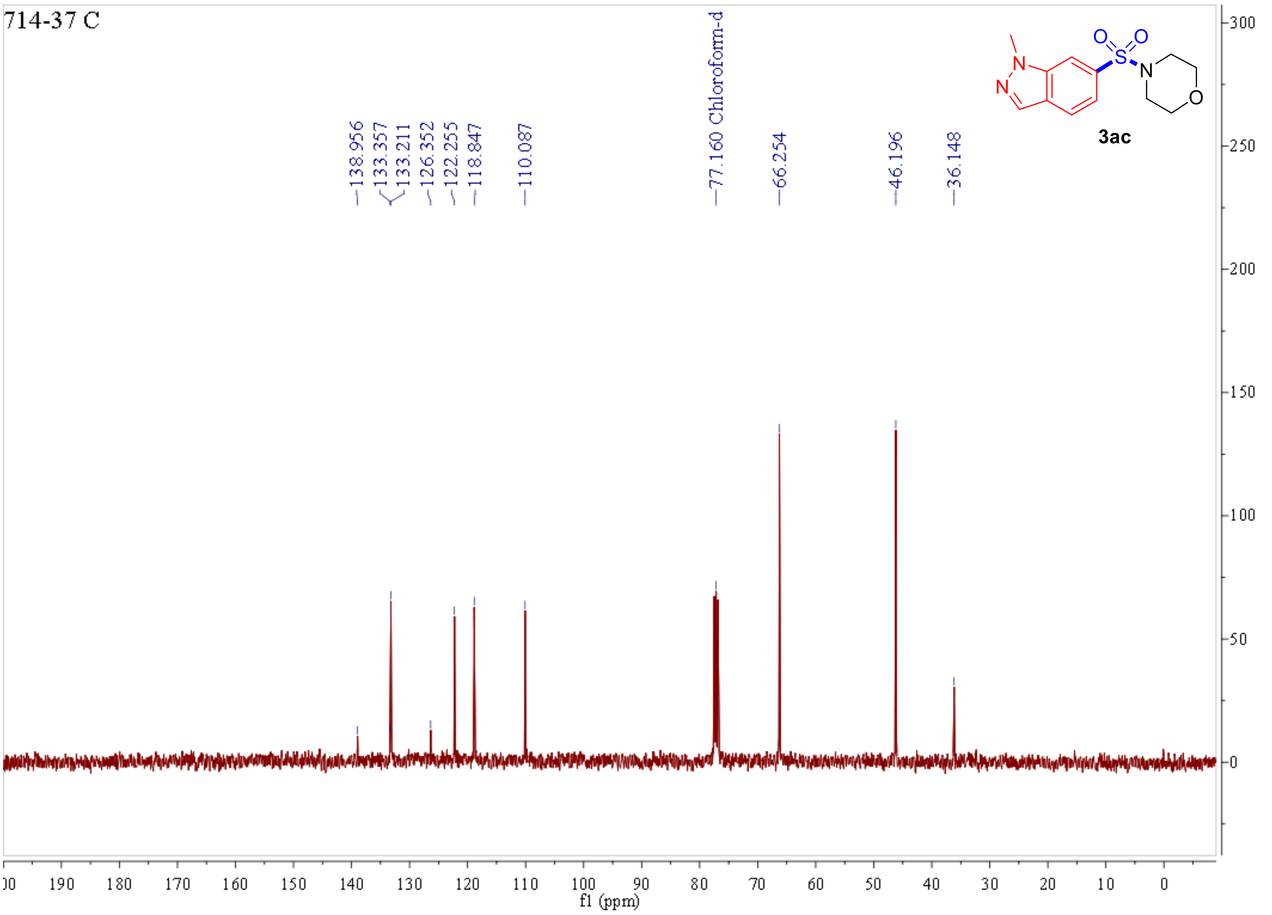
714-37 C
138.956
133.357
133.211
126.352
122.255
118.847
110.087
77.160 Chloroform-d
66.254
46.196
36.148
3ac
f1 (ppm)

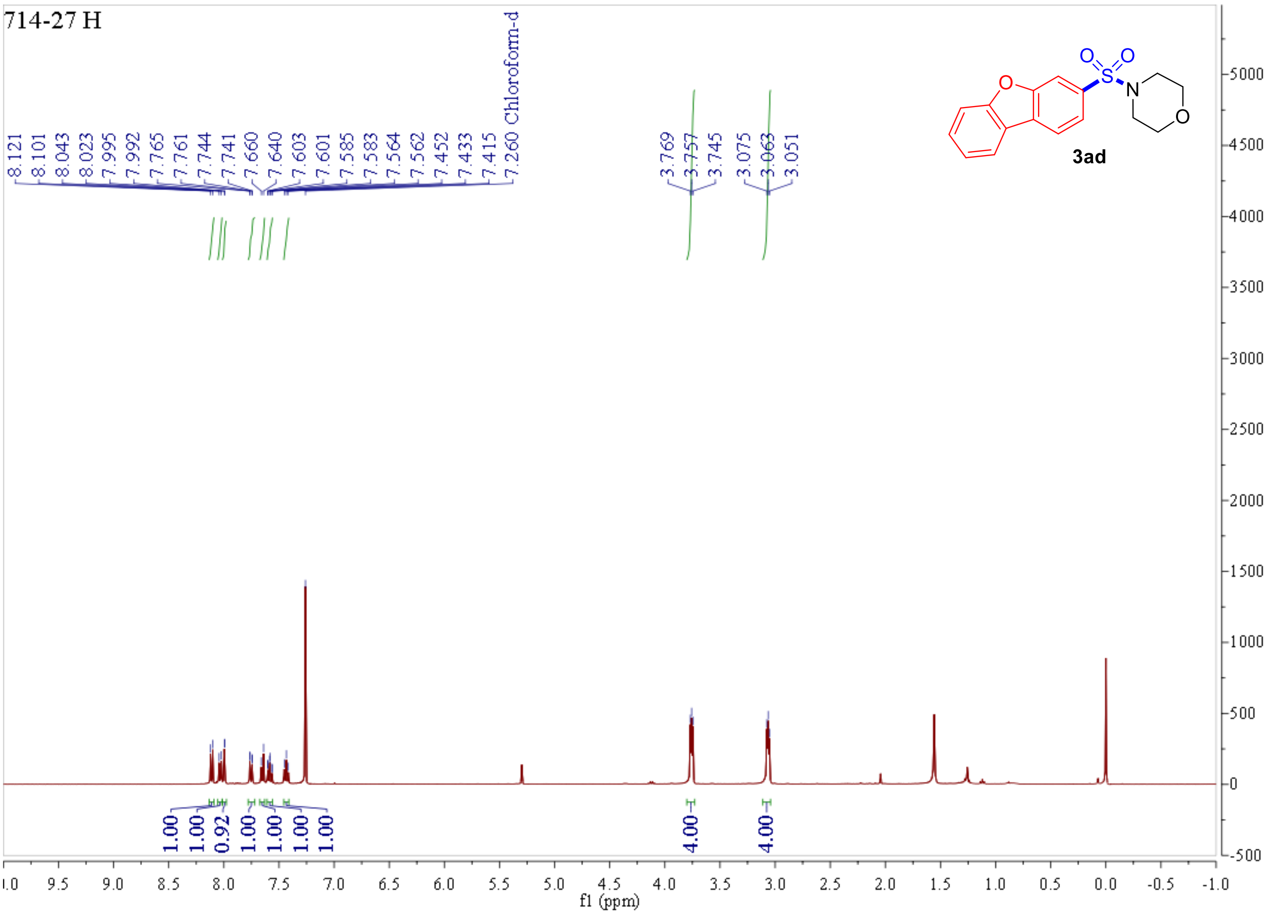
714-27 H
8.121
8.101
8.043
8.023
7.995
7.992
7.765
7.761
7.744
7.741
7.660
7.640
7.603
7.601
7.585
7.583
7.564
7.562
7.452
7.433
7.415
7.260 Chloroform-d
3.769
3.757
3.745
3.075
3.063
3.051
1.00
1.00
0.92
1.00
1.00
1.00
1.00
4.00
4.00
3ad
f1 (ppm)

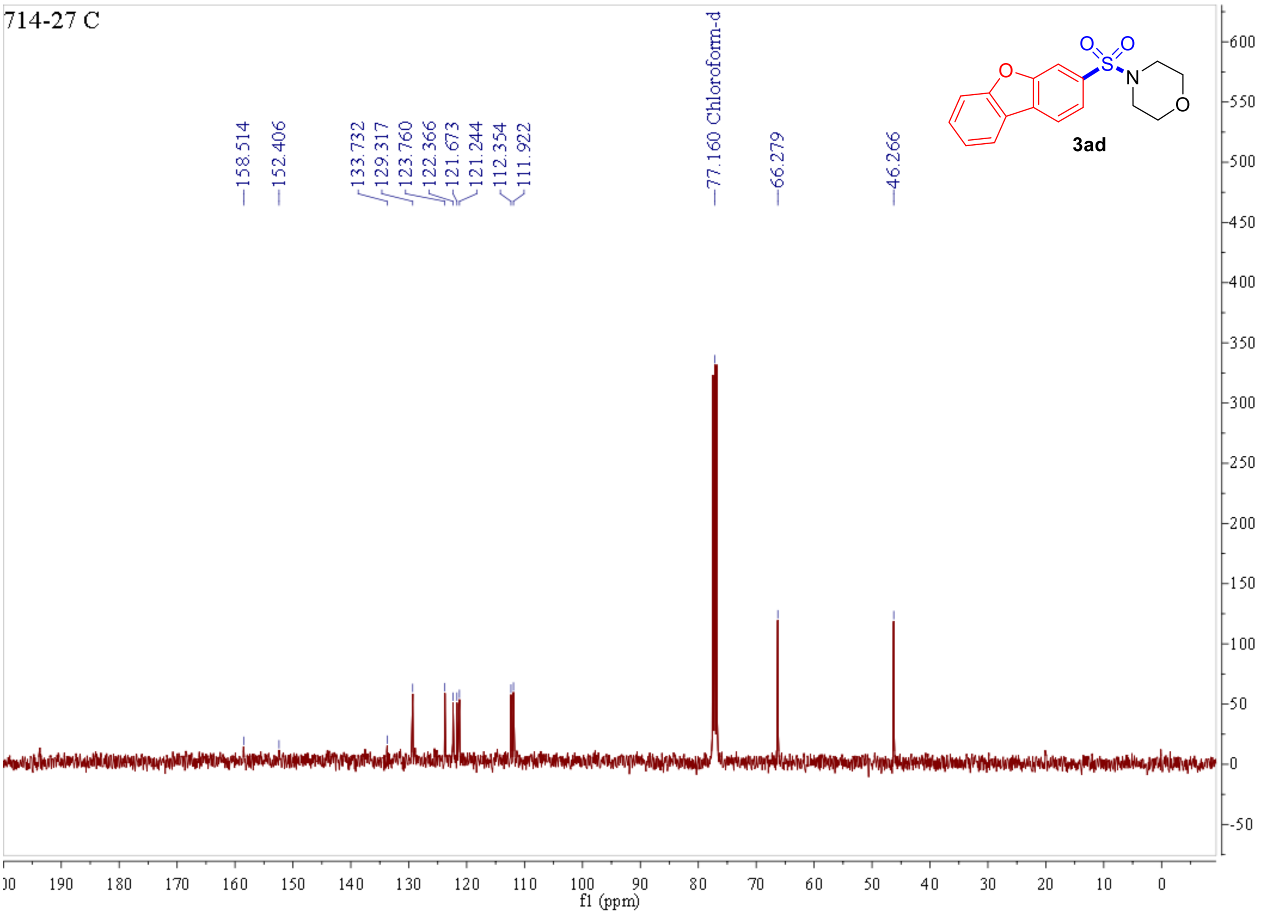
714-27 C
158.514
152.406
133.732
129.317
123.760
122.366
121.673
121.244
112.354
111.922
77.160 Chloroform-d
66.279
46.266
3ad
f1 (ppm)

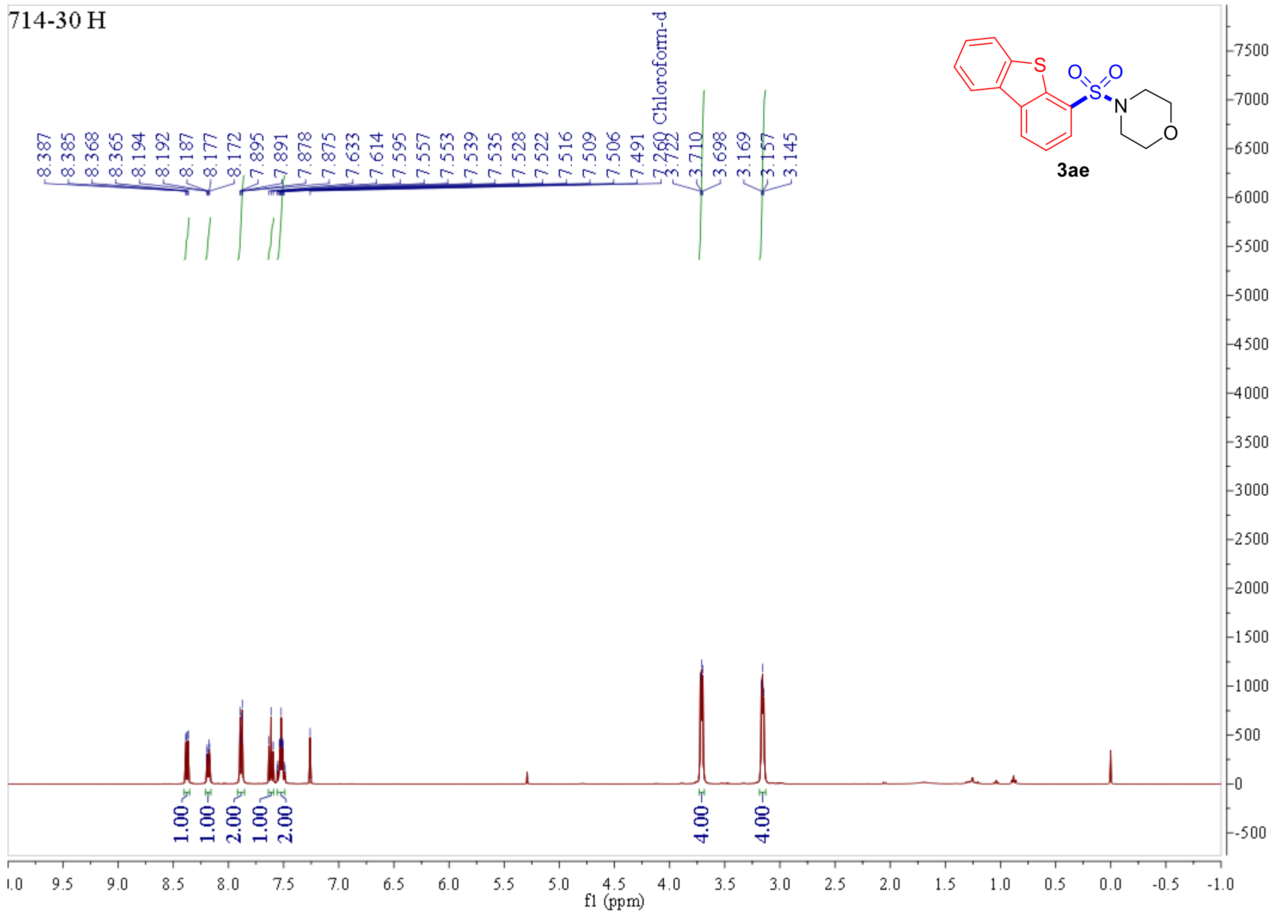
714-30 H
8.387
8.385
8.368
8.365
8.194
8.192
8.187
8.177
8.172
7.895
7.891
7.878
7.875
7.633
7.614
7.595
7.557
7.553
7.539
7.535
7.528
7.522
7.516
7.509
7.506
7.491
7.260 Chloroform-d
3.722
3.710
3.698
3.169
3.157
3.145
S
O
O
S
N
O
3ae
1.00
1.00
2.00
1.00
2.00
4.00
4.00
f1 (ppm)

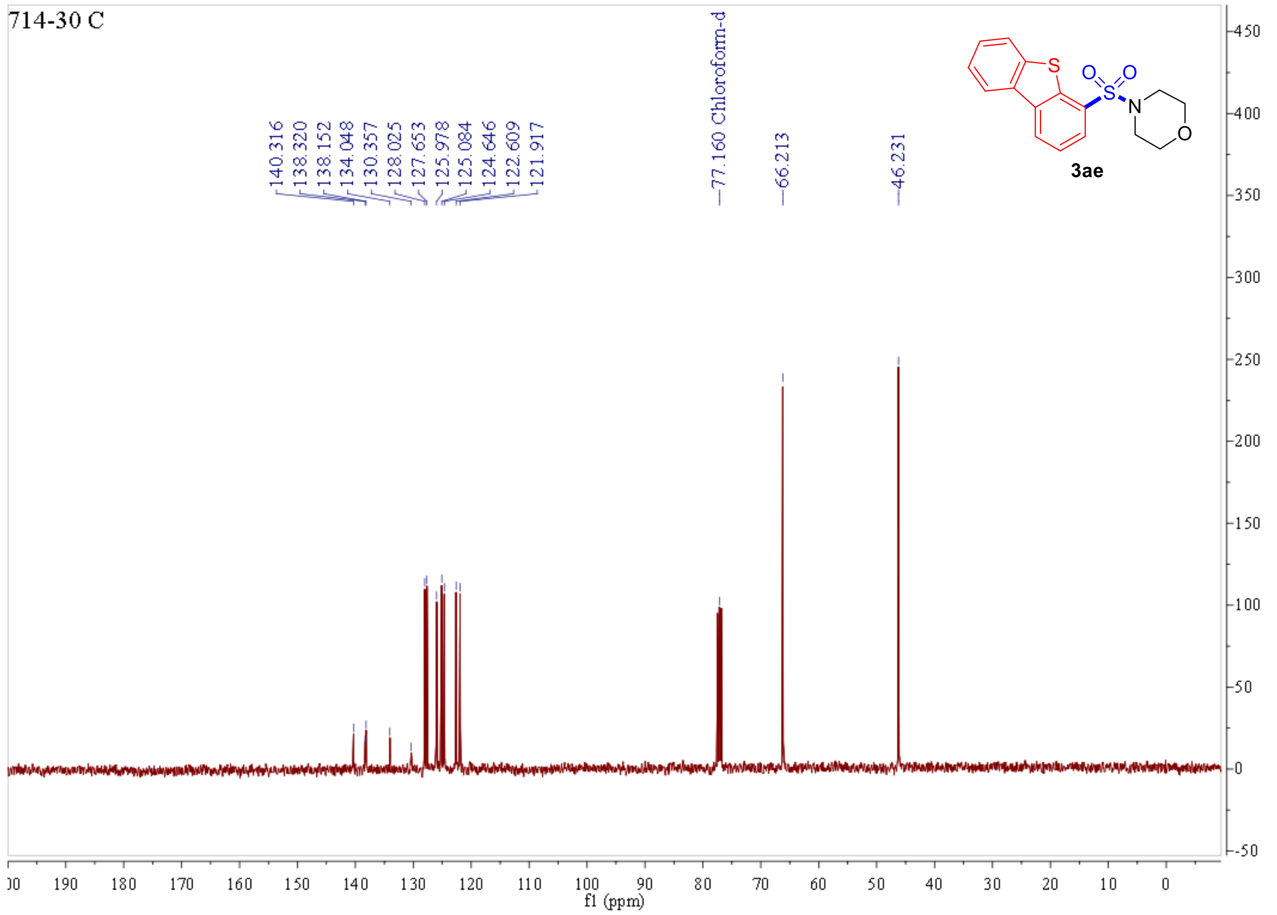
714-30 C
140.316
138.320
138.152
134.048
130.357
128.025
127.653
125.978
125.084
124.646
122.609
121.917
77.160 Chloroform-d
66.213
46.231
S
O
O
S
N
O
3ae
f1 (ppm)

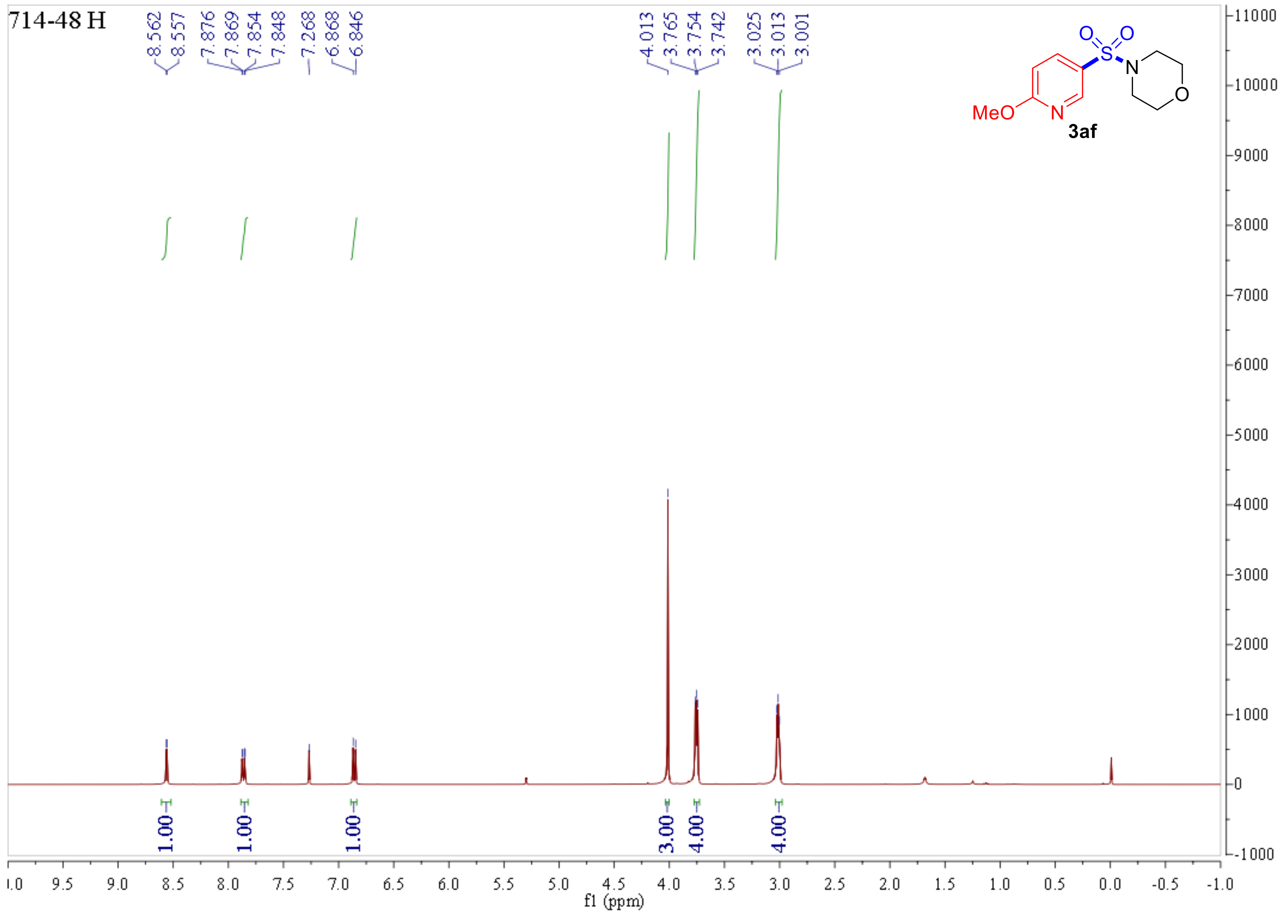
714-48 H
8.562
8.557
7.876
7.869
7.854
7.848
7.268
6.868
6.846
4.013
3.765
3.754
3.742
3.025
3.013
3.001
MeO
N
O
S
O
N
O
3af
1.00
1.00
1.00
3.00
4.00
4.00
f1 (ppm)

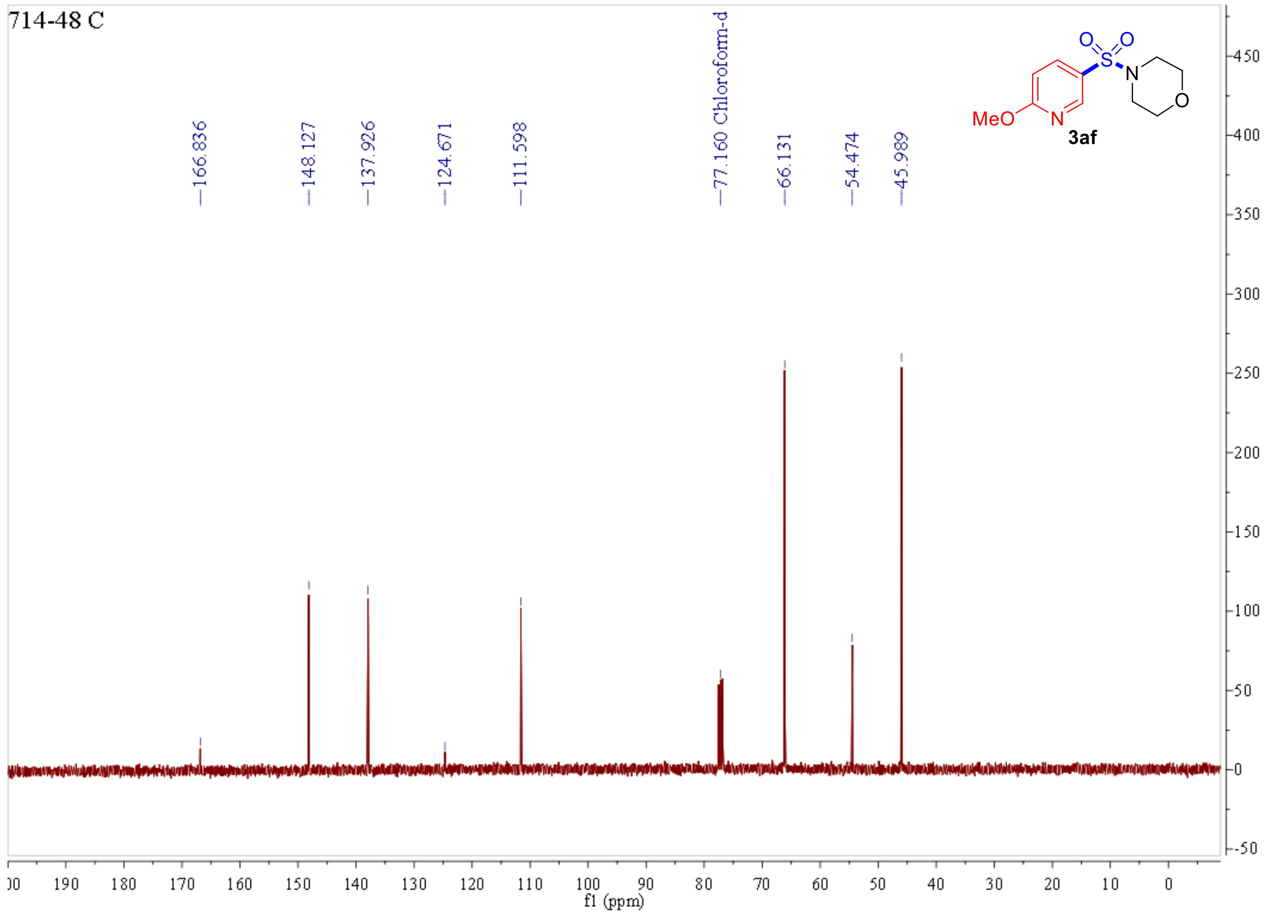
714-48 C
166.836
148.127
137.926
124.671
111.598
77.160 Chloroform-d
66.131
54.474
45.989
MeO
N
O
S
O
N
O
3af
f1 (ppm)

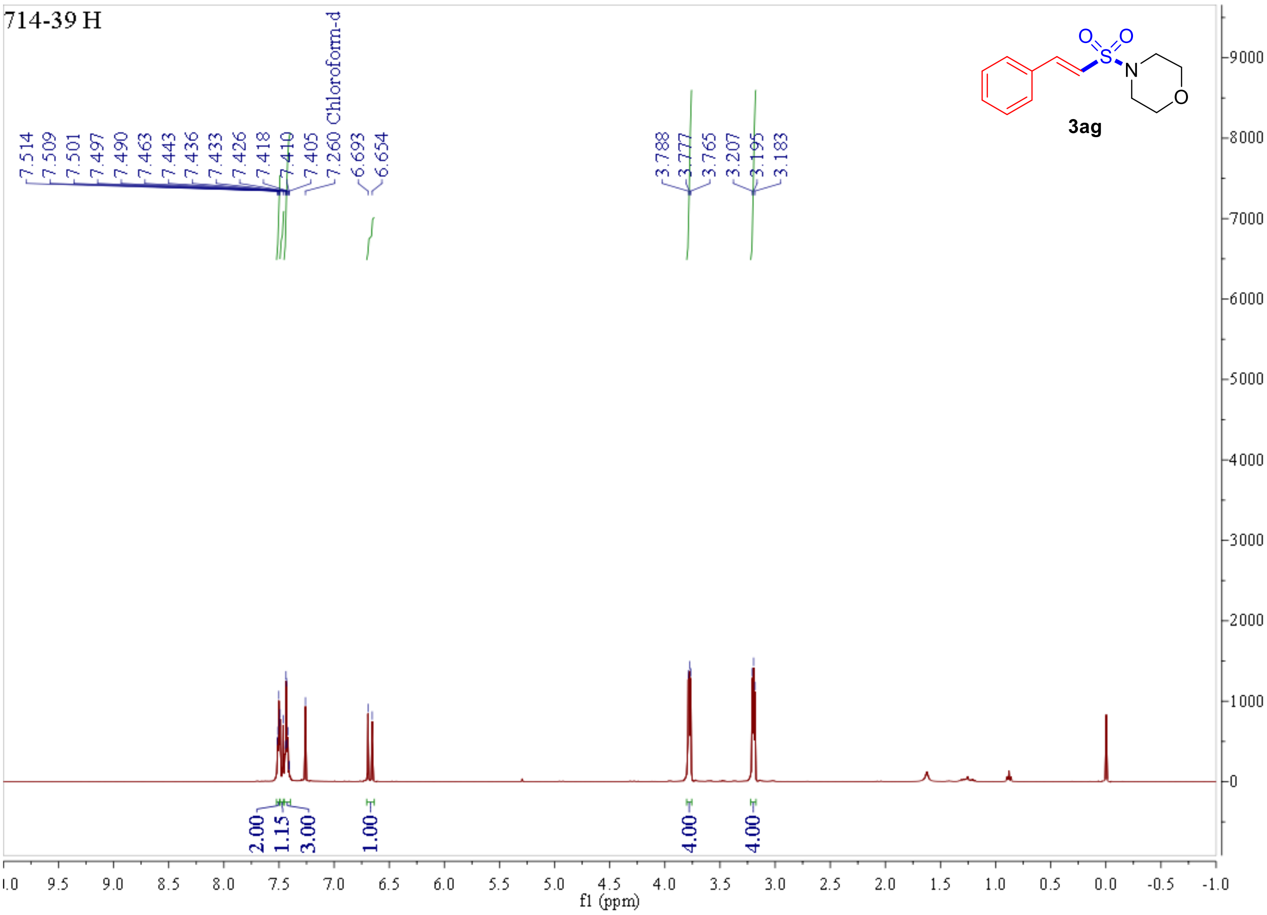
714-39 H
7.514
7.509
7.501
7.497
7.490
7.463
7.443
7.436
7.433
7.426
7.418
7.410
7.405
7.260 Chloroform-d
6.693
6.654
3.788
3.777
3.765
3.207
3.195
3.183
3ag
2.00
1.15
3.00
1.00
4.00
4.00
f1 (ppm)

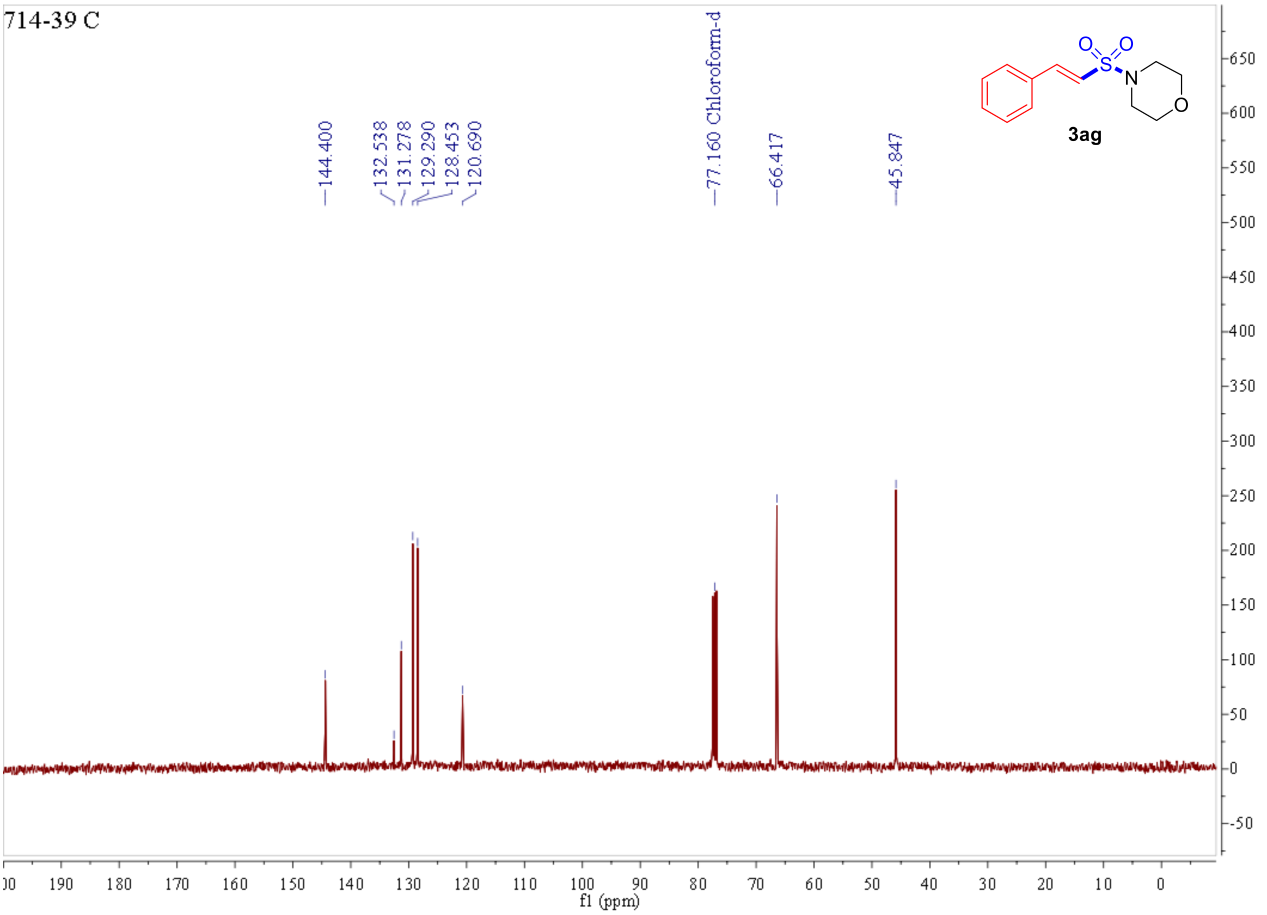
714-39 C
144.400
132.538
131.278
129.290
128.453
120.690
77.160 Chloroform-d
66.417
45.847
3ag
f1 (ppm)

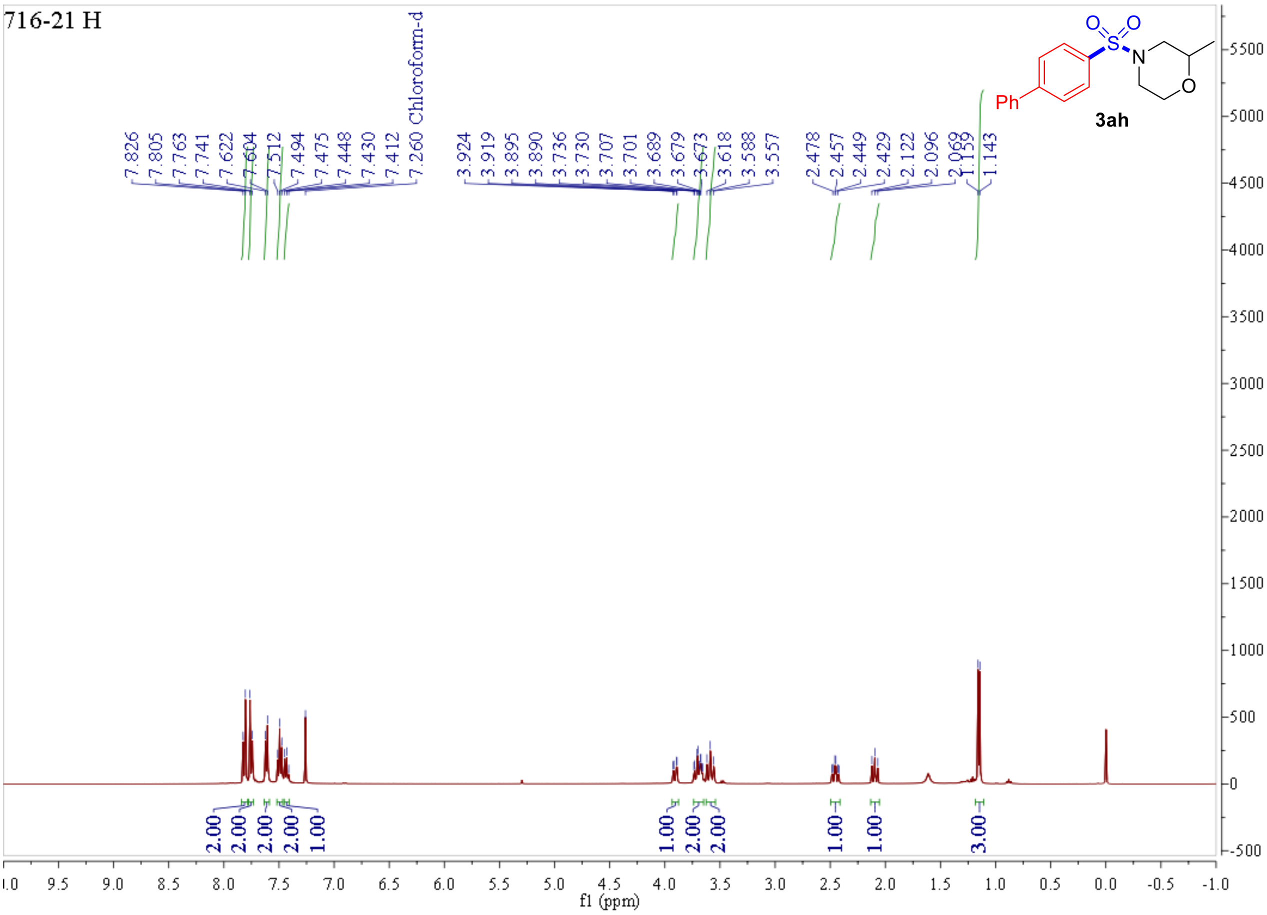
716-21 H
3ah
Ph
7.826
7.805
7.763
7.741
7.622
7.604
7.512
7.494
7.475
7.448
7.430
7.412
7.260 Chloroform-d
3.924
3.919
3.895
3.890
3.736
3.730
3.707
3.701
3.689
3.679
3.673
3.618
3.588
3.557
2.478
2.457
2.449
2.429
2.122
2.096
2.069
1.159
1.143
2.00
2.00
2.00
2.00
1.00
1.00
2.00
2.00
1.00
1.00
3.00
f1 (ppm)

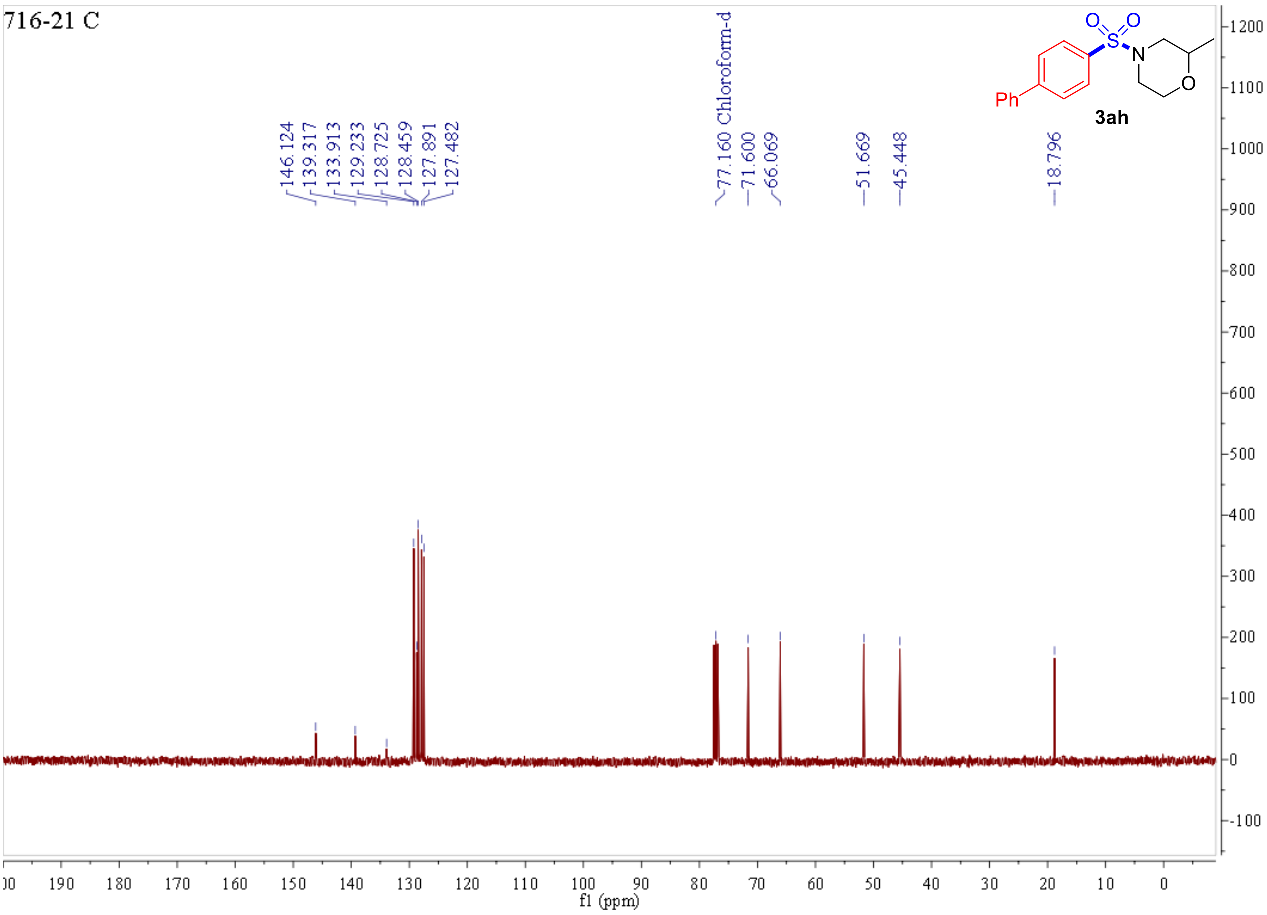
716-21 C
3ah
Ph
146.124
139.317
133.913
129.233
128.725
128.459
127.891
127.482
77.160 Chloroform-d
71.600
66.069
51.669
45.448
18.796
f1 (ppm)

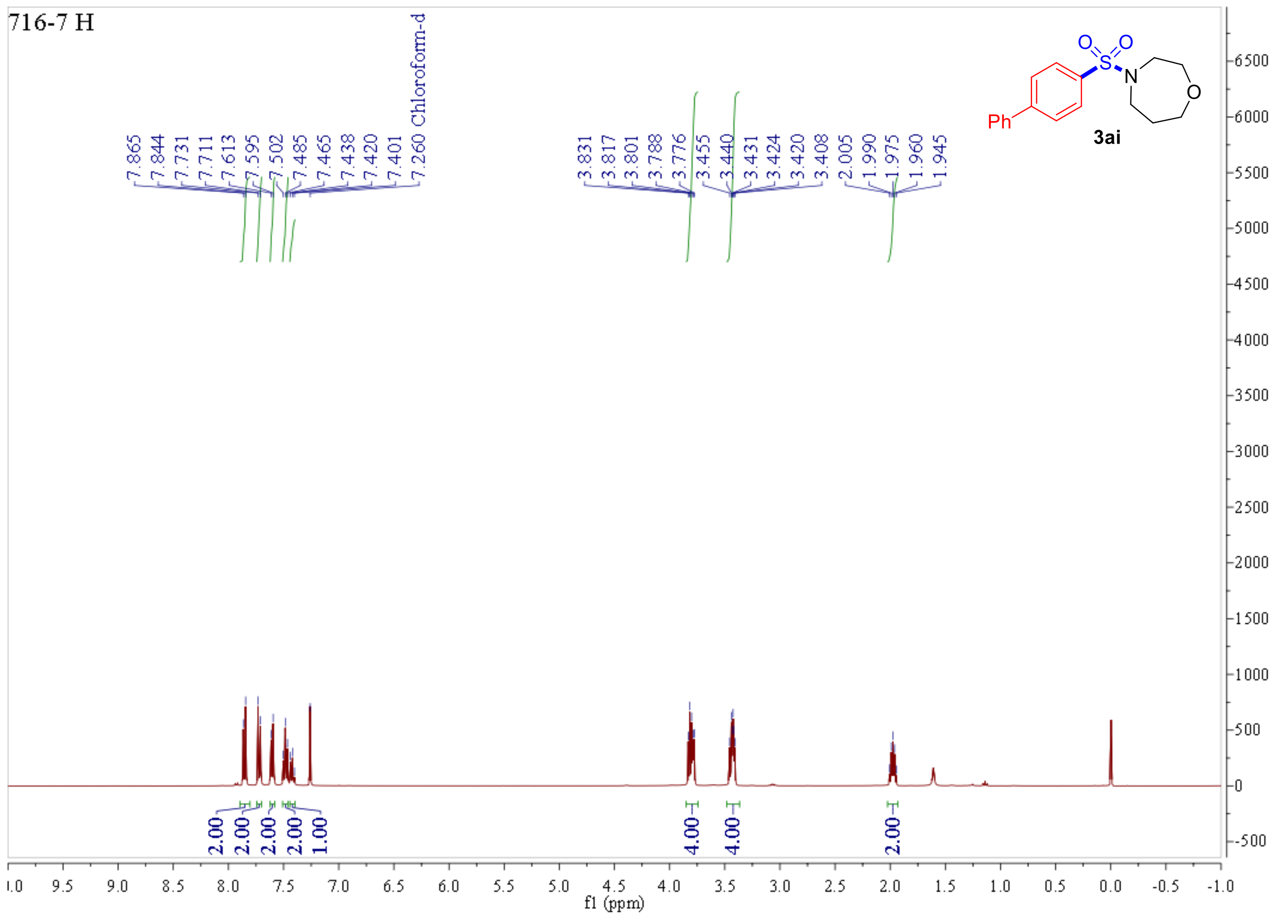
716-7 H
7.865
7.844
7.731
7.711
7.613
7.595
7.502
7.485
7.465
7.438
7.420
7.401
7.260 Chloroform-d
3.831
3.817
3.801
3.788
3.776
3.455
3.440
3.431
3.424
3.420
3.408
2.005
1.990
1.975
1.960
1.945
2.00
2.00
2.00
2.00
1.00
4.00
4.00
2.00
Ph
O
S
O
N
O
3ai
f1 (ppm)

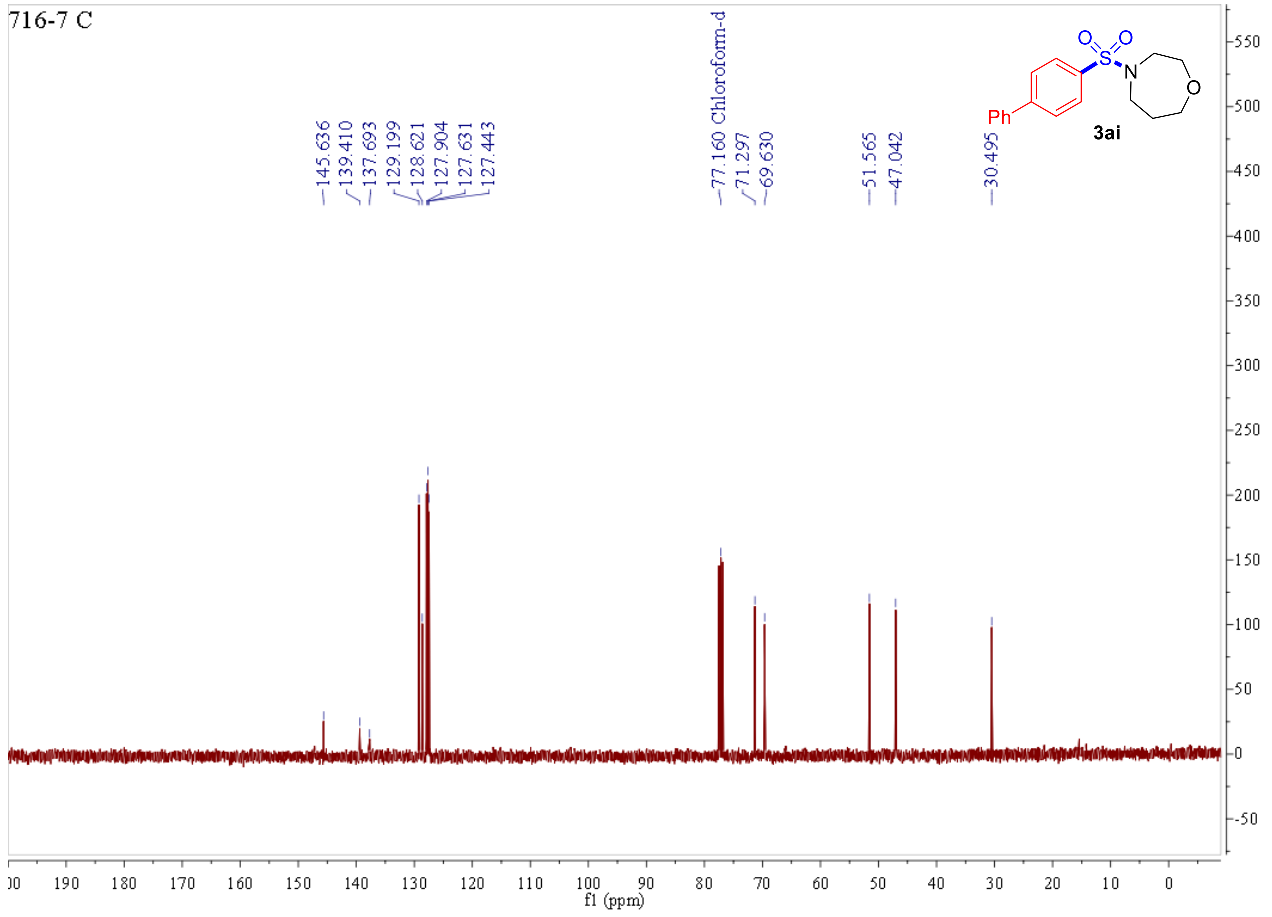
716-7 C
145.636
139.410
137.693
129.199
128.621
127.904
127.631
127.443
77.160 Chloroform-d
71.297
69.630
51.565
47.042
30.495
Ph
O
S
O
N
O
3ai
f1 (ppm)

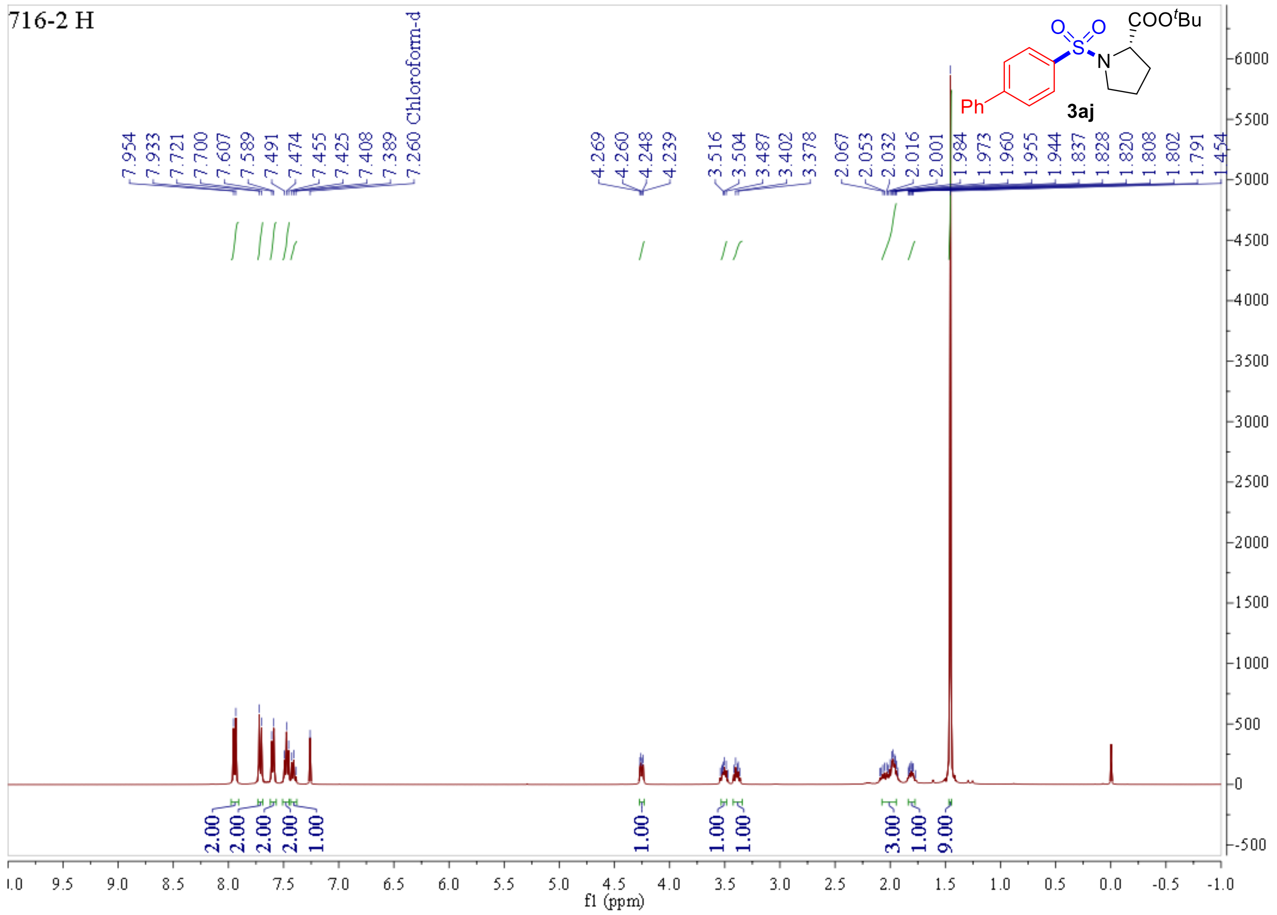
716-2 H
7.954
7.933
7.721
7.700
7.607
7.589
7.491
7.474
7.455
7.425
7.408
7.389
7.260 Chloroform-d
4.269
4.260
4.248
4.239
3.516
3.504
3.487
3.402
3.378
2.067
2.053
2.032
2.016
2.001
1.984
1.973
1.960
1.955
1.944
1.837
1.828
1.820
1.808
1.802
1.791
1.454
2.00
2.00
2.00
2.00
1.00
1.00
1.00
1.00
3.00
1.00
9.00
f1 (ppm)
COO^{t}Bu
Ph
3aj

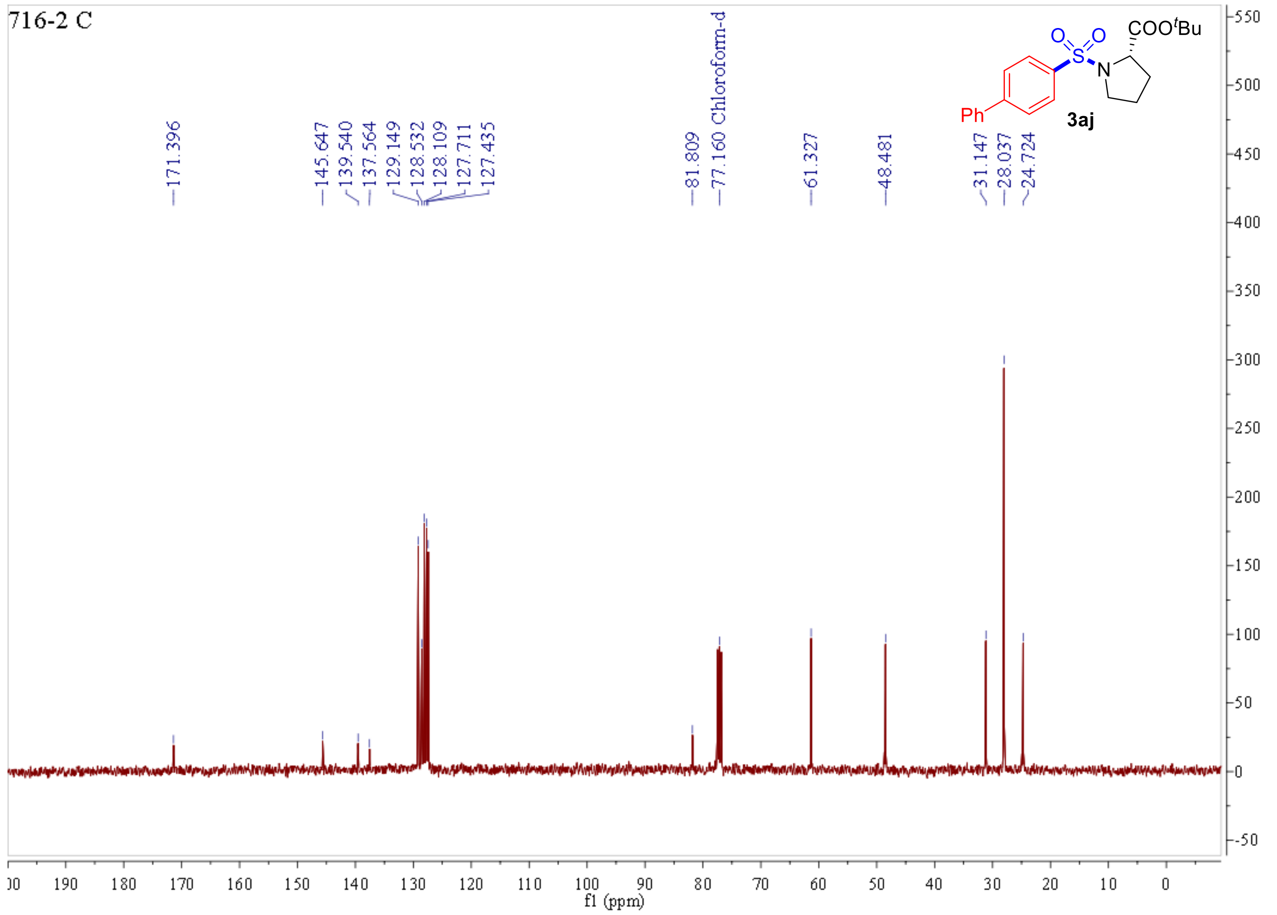
716-2 C
171.396
145.647
139.540
137.564
129.149
128.532
128.109
127.711
127.435
81.809
77.160 Chloroform-d
61.327
48.481
31.147
28.037
24.724
f1 (ppm)
COO^{t}Bu
Ph
3aj

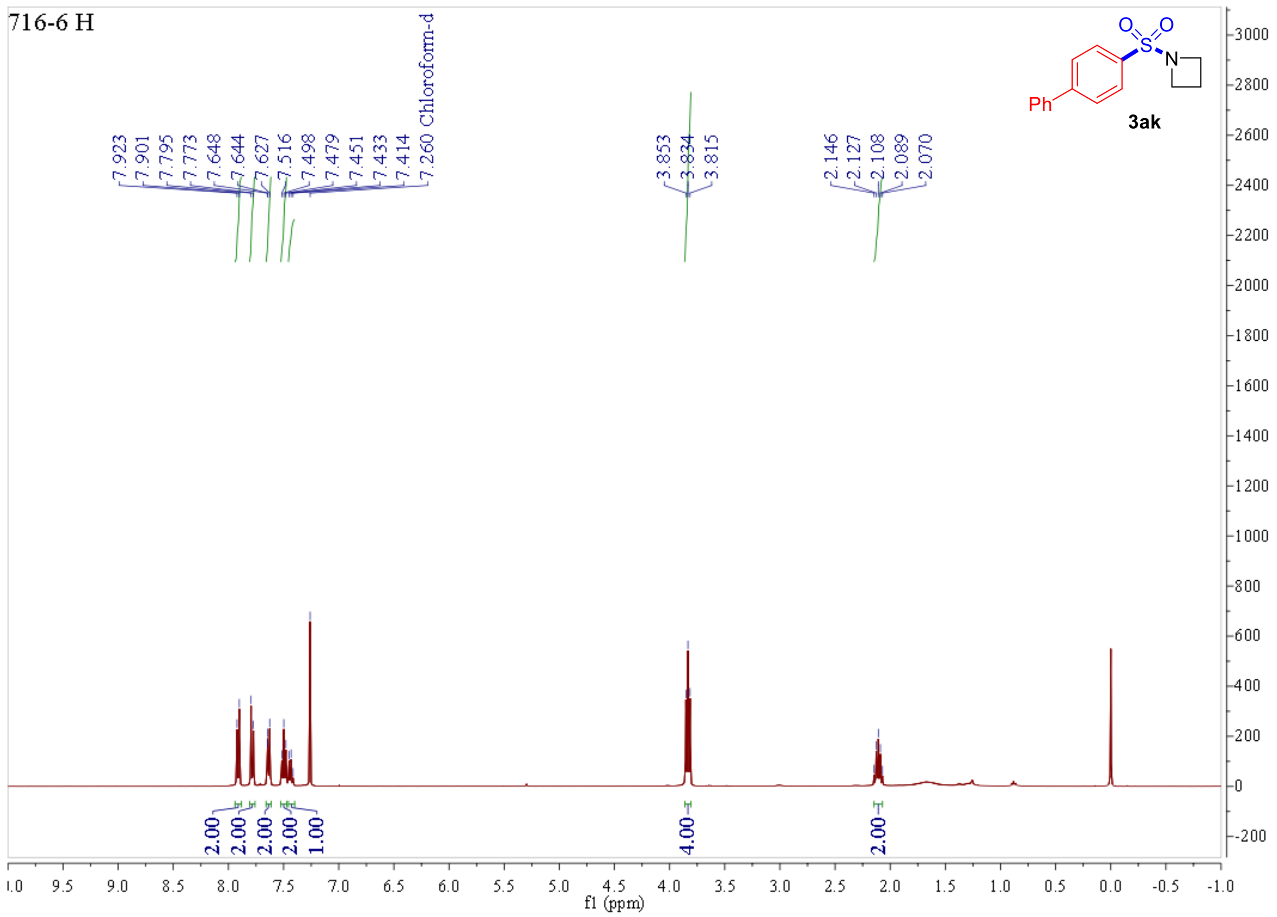
716-6 H
7.923
7.901
7.795
7.773
7.648
7.644
7.627
7.516
7.498
7.479
7.451
7.433
7.414
7.260 Chloroform-d
3.853
3.834
3.815
2.146
2.127
2.108
2.089
2.070
Ph
3ak
2.00
2.00
2.00
2.00
1.00
4.00
2.00
f1 (ppm)

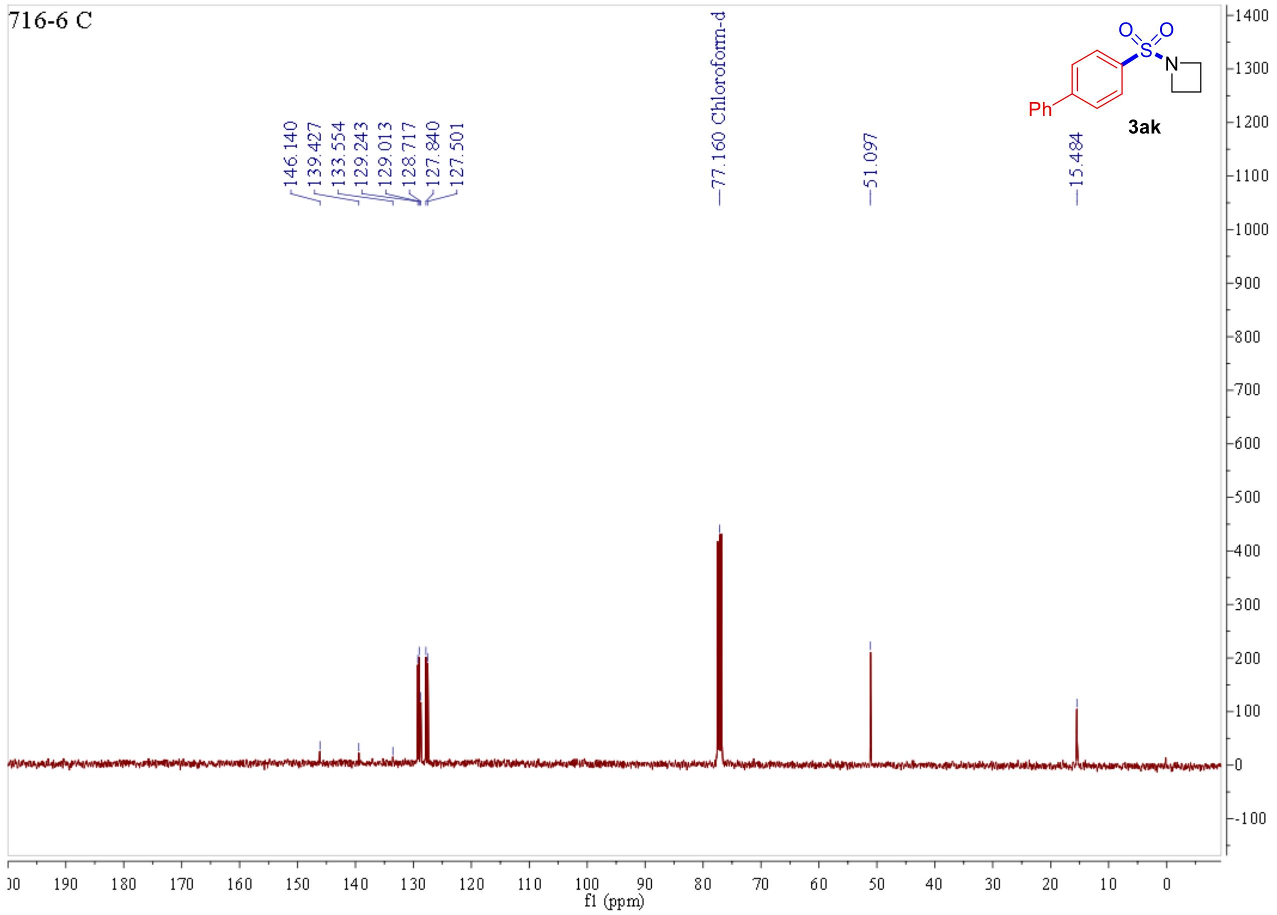
716-6 C
146.140
139.427
133.554
129.243
129.013
128.717
127.840
127.501
77.160 Chloroform-d
51.097
15.484
Ph
3ak
f1 (ppm)

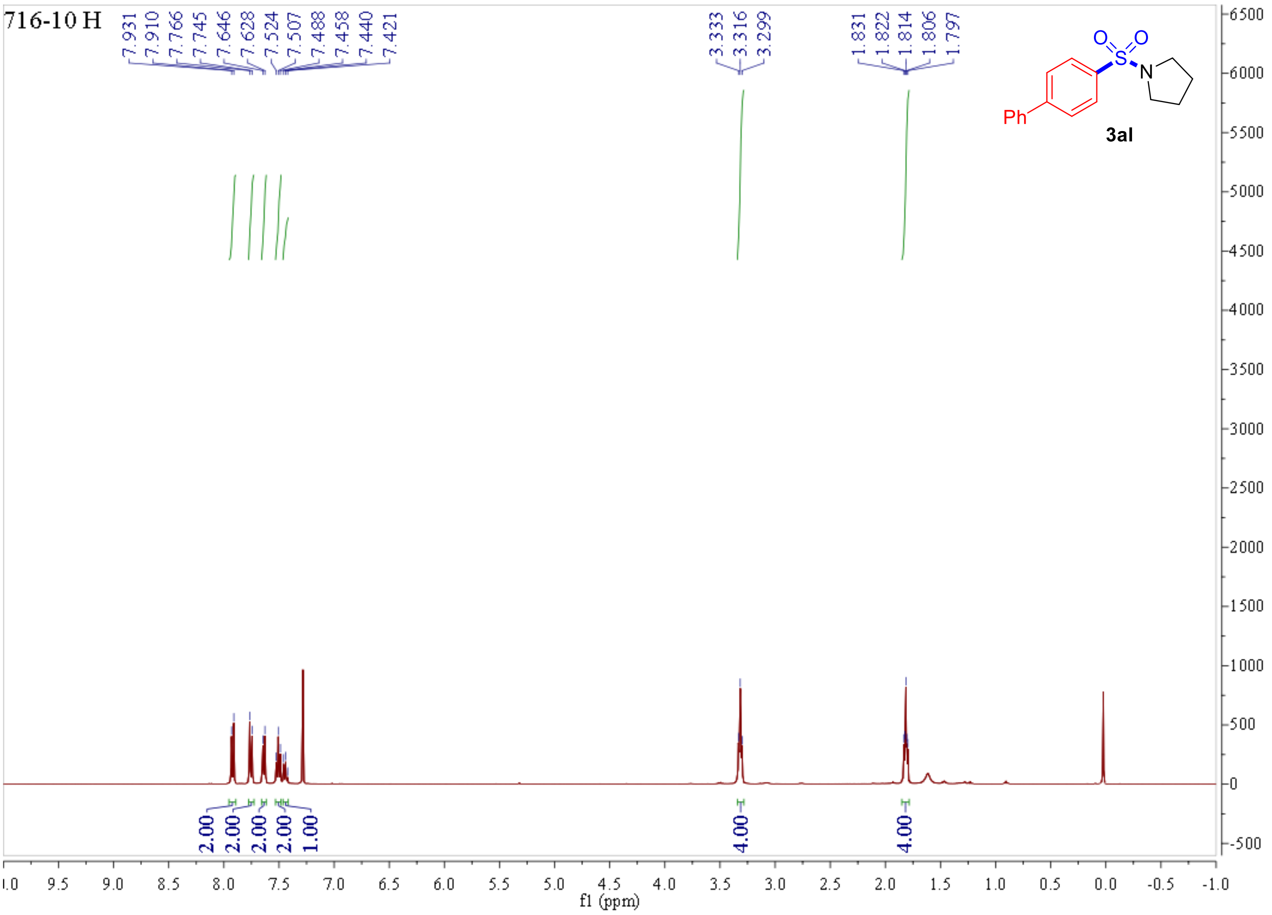
716-10 H
7.931
7.910
7.766
7.745
7.646
7.628
7.524
7.507
7.488
7.458
7.440
7.421
3.333
3.316
3.299
1.831
1.822
1.814
1.806
1.797
Ph
3al
2.00
2.00
2.00
2.00
1.00
4.00
4.00
f1 (ppm)

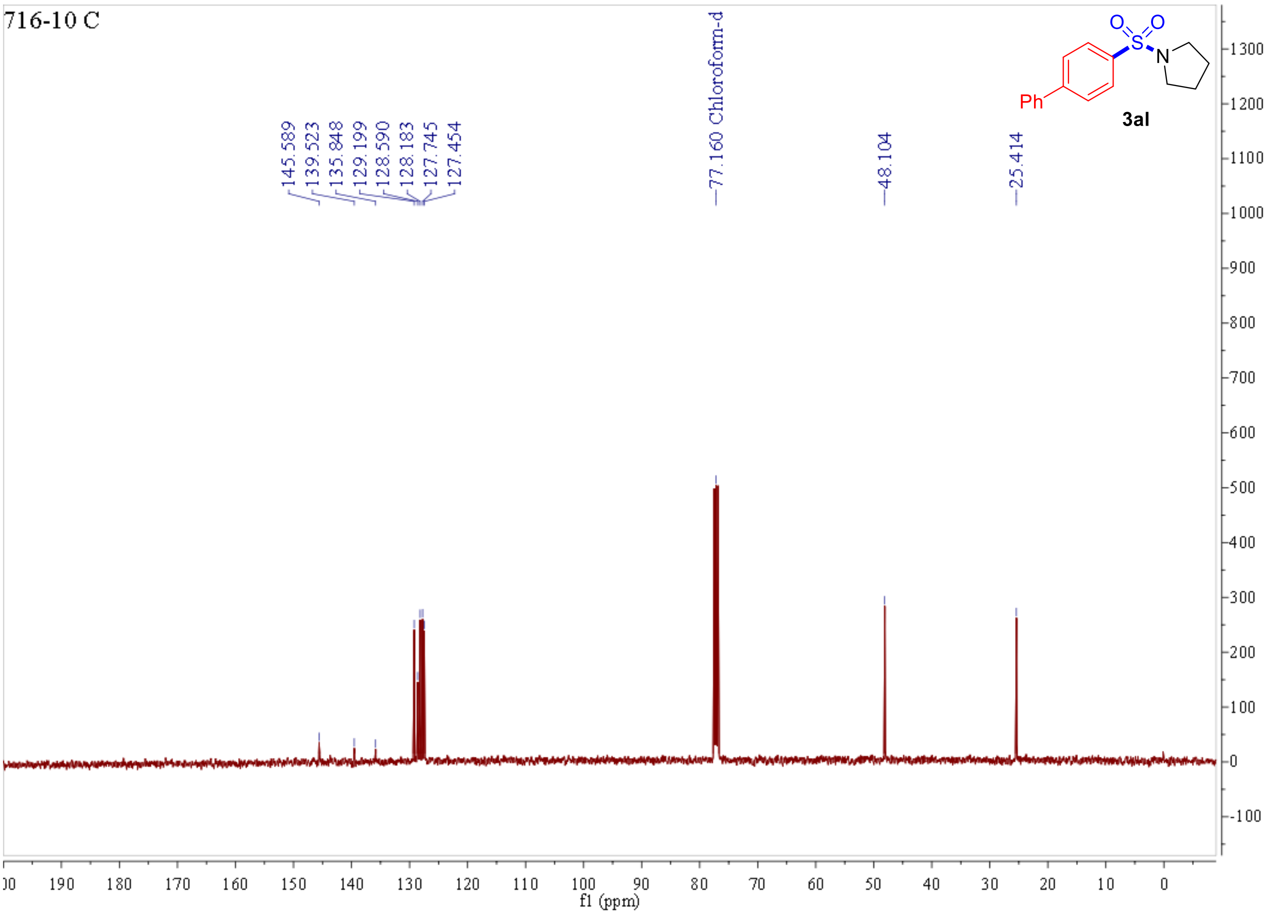
716-10 C
145.589
139.523
135.848
129.199
128.590
128.183
127.745
127.454
77.160 Chloroform-d
48.104
25.414
Ph
3al
f1 (ppm)

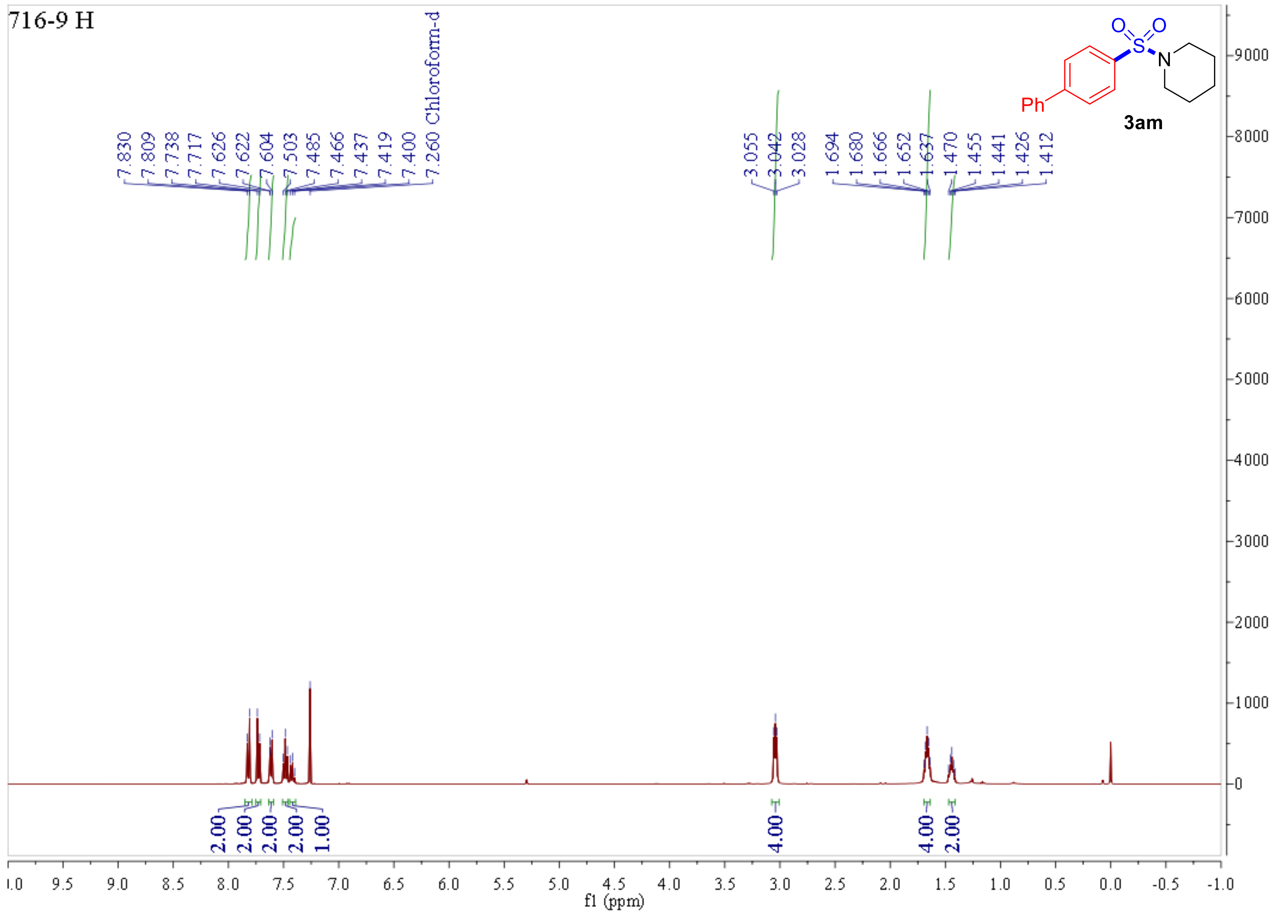
716-9 H
7.830
7.809
7.738
7.717
7.626
7.622
7.604
7.503
7.485
7.466
7.437
7.419
7.400
7.260 Chloroform-d
3.055
3.042
3.028
1.694
1.680
1.666
1.652
1.637
1.470
1.455
1.441
1.426
1.412
Ph
O
S
O
N
3am
2.00
2.00
2.00
2.00
1.00
4.00
4.00
2.00
f1 (ppm)

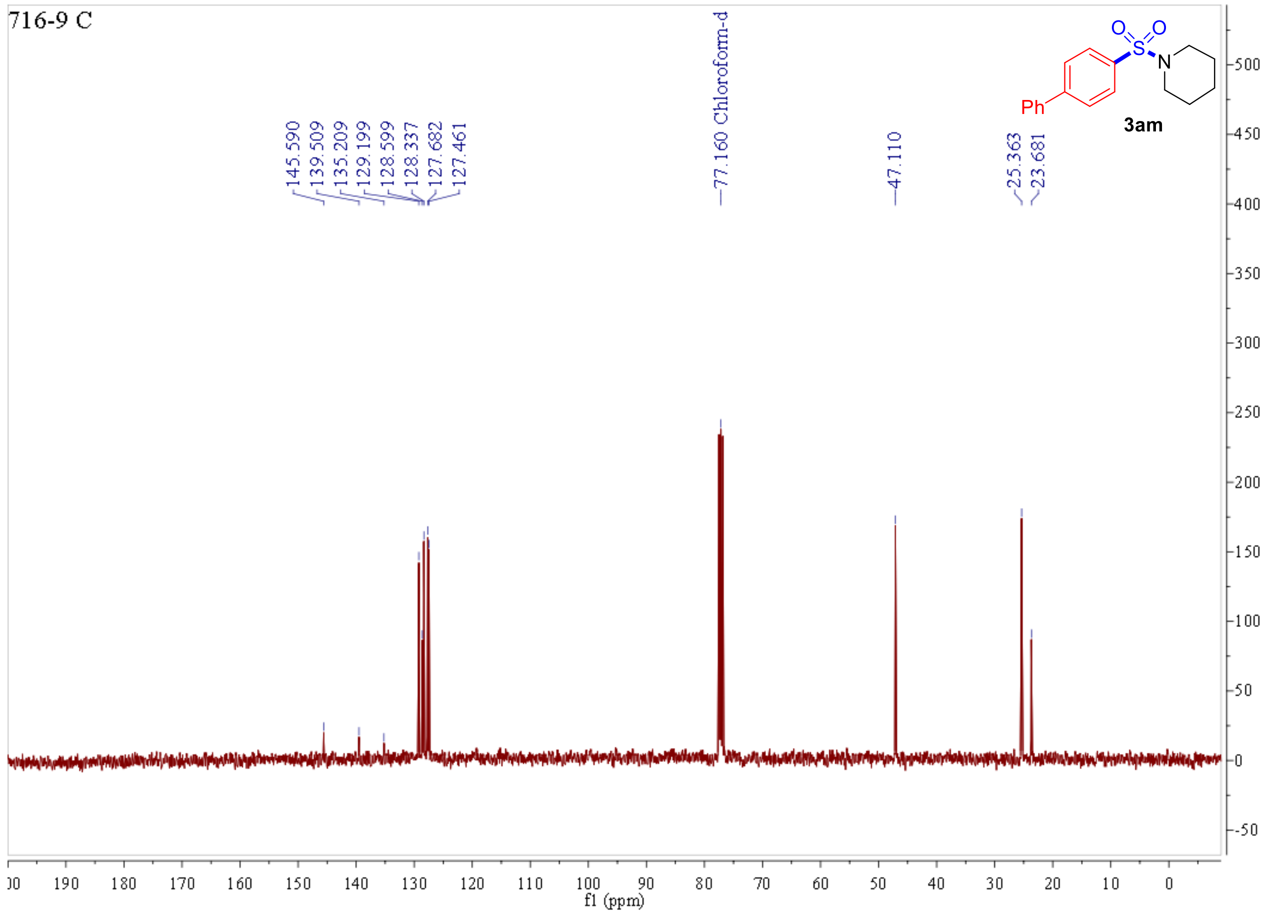
716-9 C
145.590
139.509
135.209
129.199
128.599
128.337
127.682
127.461
77.160 Chloroform-d
47.110
25.363
23.681
Ph
O
S
O
N
3am
f1 (ppm)

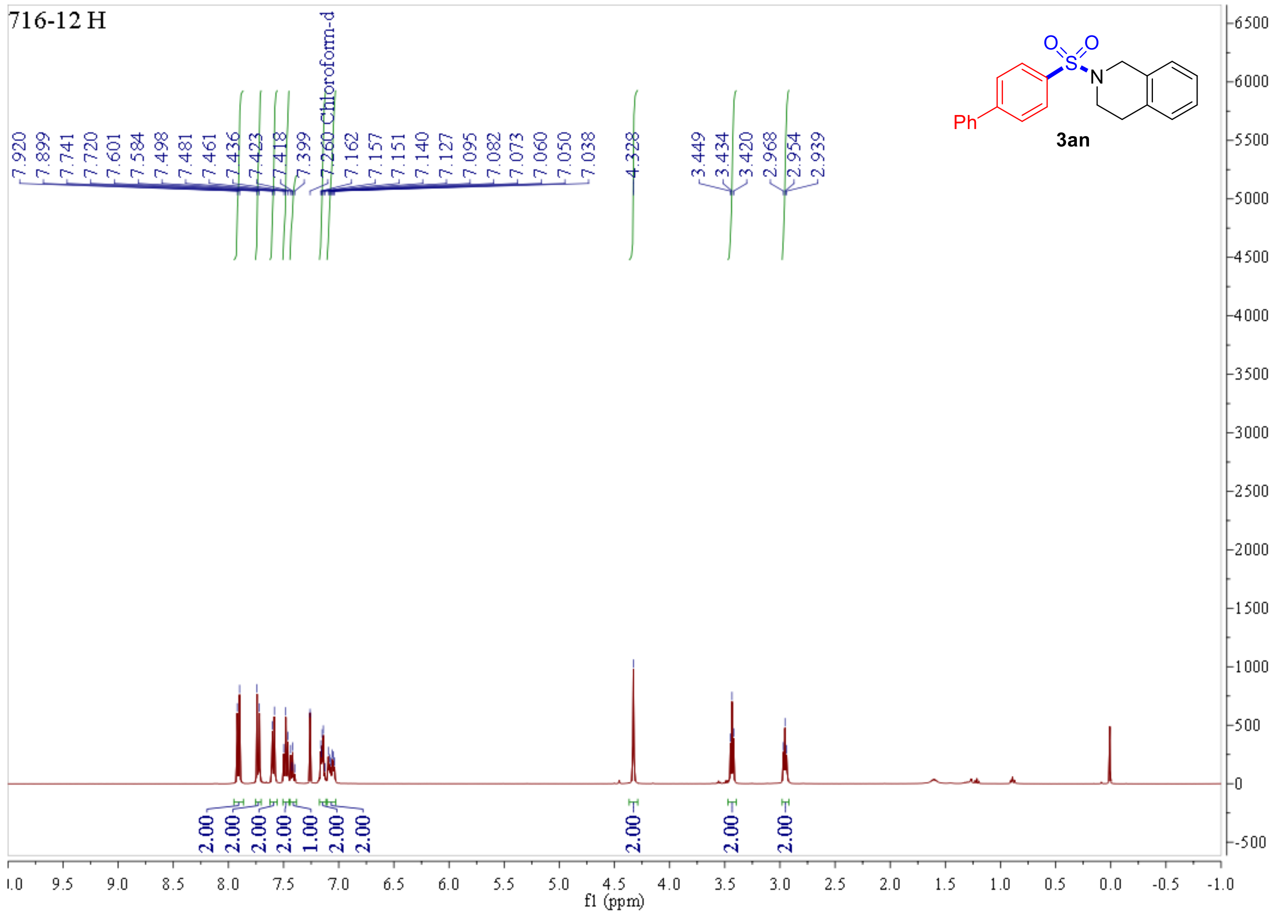
716-12 H
7.920
7.899
7.741
7.720
7.601
7.584
7.498
7.481
7.461
7.436
7.423
7.418
7.399
7.260 Chloroform-d
7.162
7.157
7.151
7.140
7.127
7.095
7.082
7.073
7.060
7.050
7.038
4.328
3.449
3.434
3.420
2.968
2.954
2.939
Ph
3an
2.00
2.00
2.00
2.00
1.00
2.00
2.00
2.00
2.00
2.00
f1 (ppm)

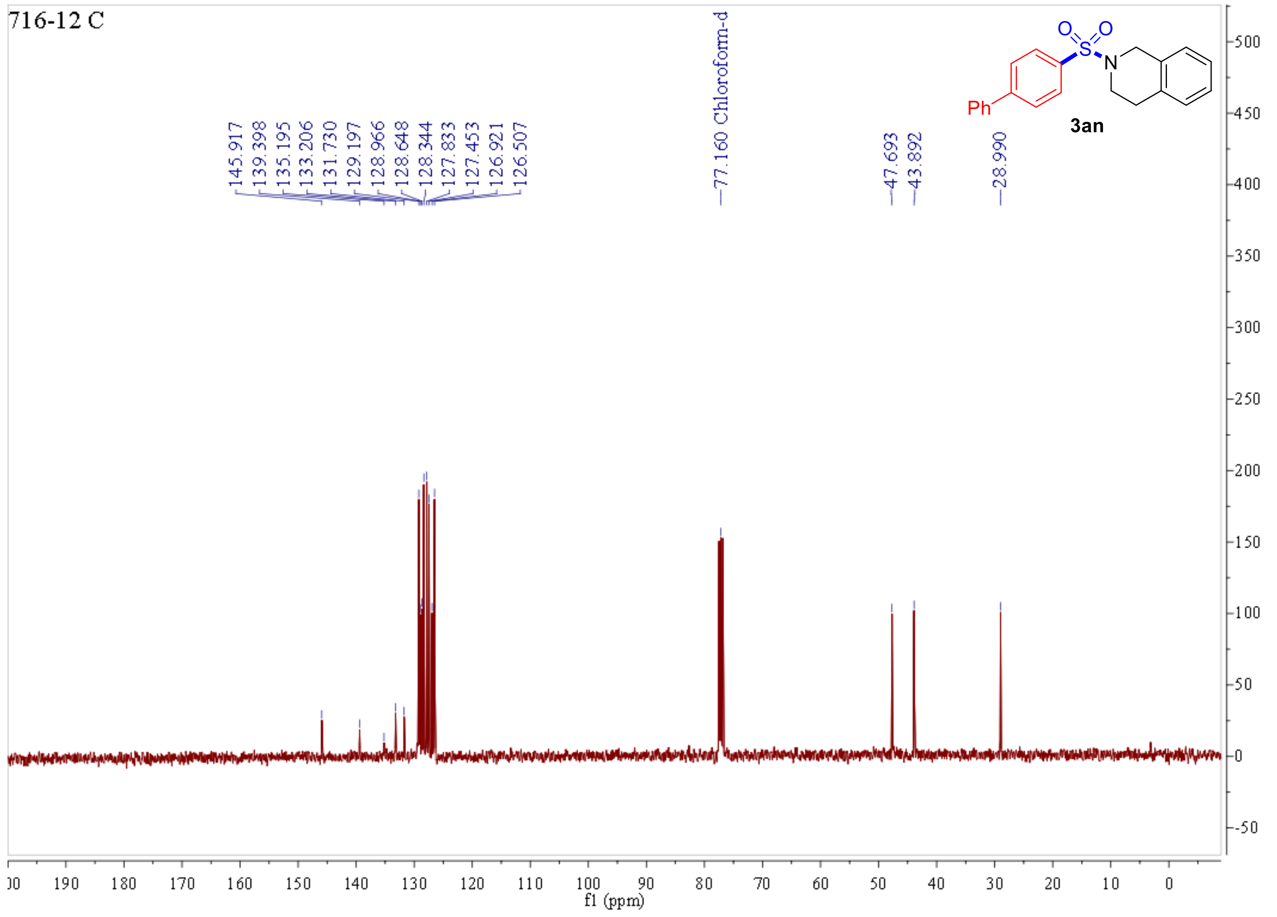
716-12 C
145.917
139.398
135.195
133.206
131.730
129.197
128.966
128.648
128.344
127.833
127.453
126.921
126.507
77.160 Chloroform-d
47.693
43.892
28.990
Ph
3an
f1 (ppm)

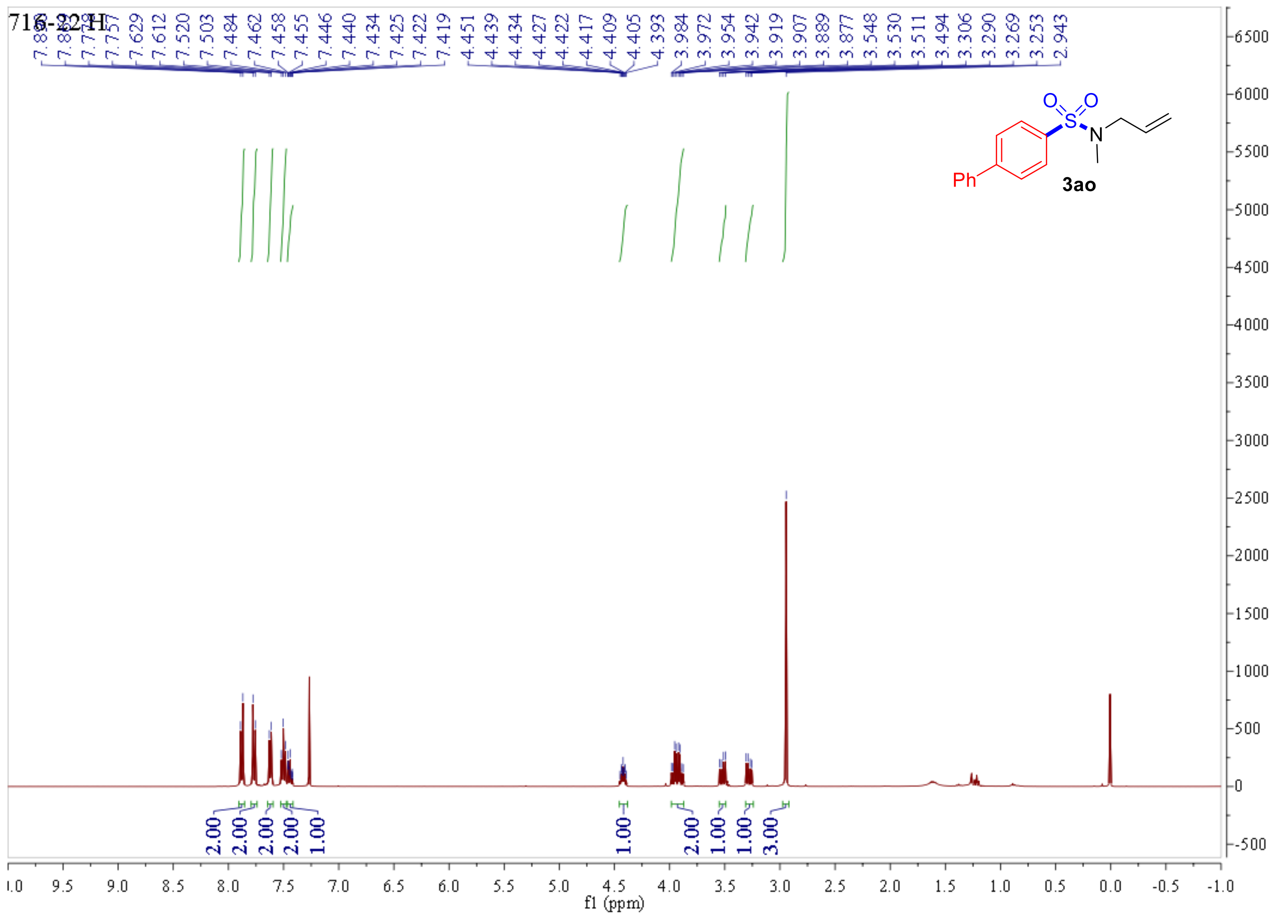
716-22 H
3ao
Ph
f1 (ppm)

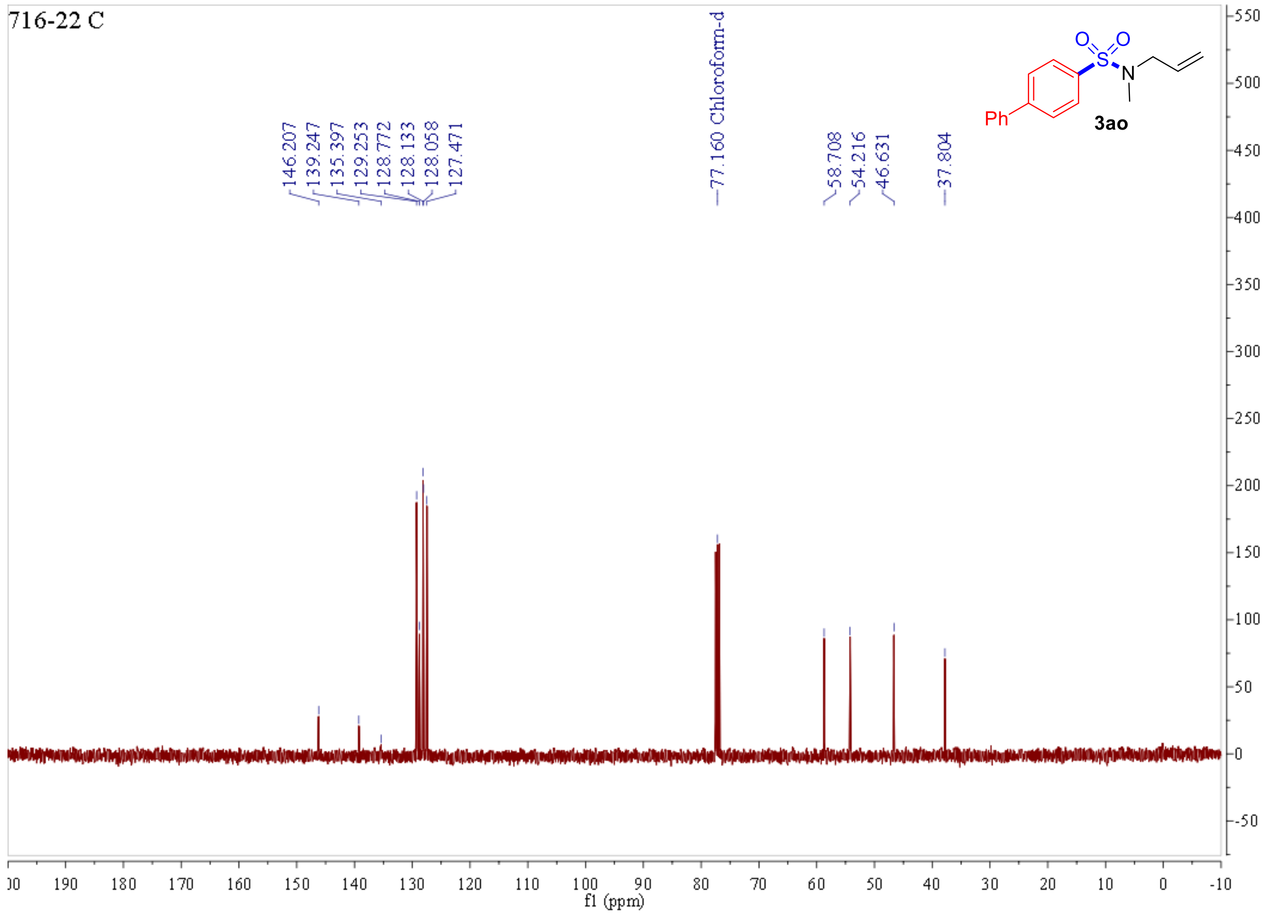
716-22 C
146.207
139.247
135.397
129.253
128.772
128.133
128.058
127.471
77.160 Chloroform-d
58.708
54.216
46.631
37.804
3ao
Ph
f1 (ppm)

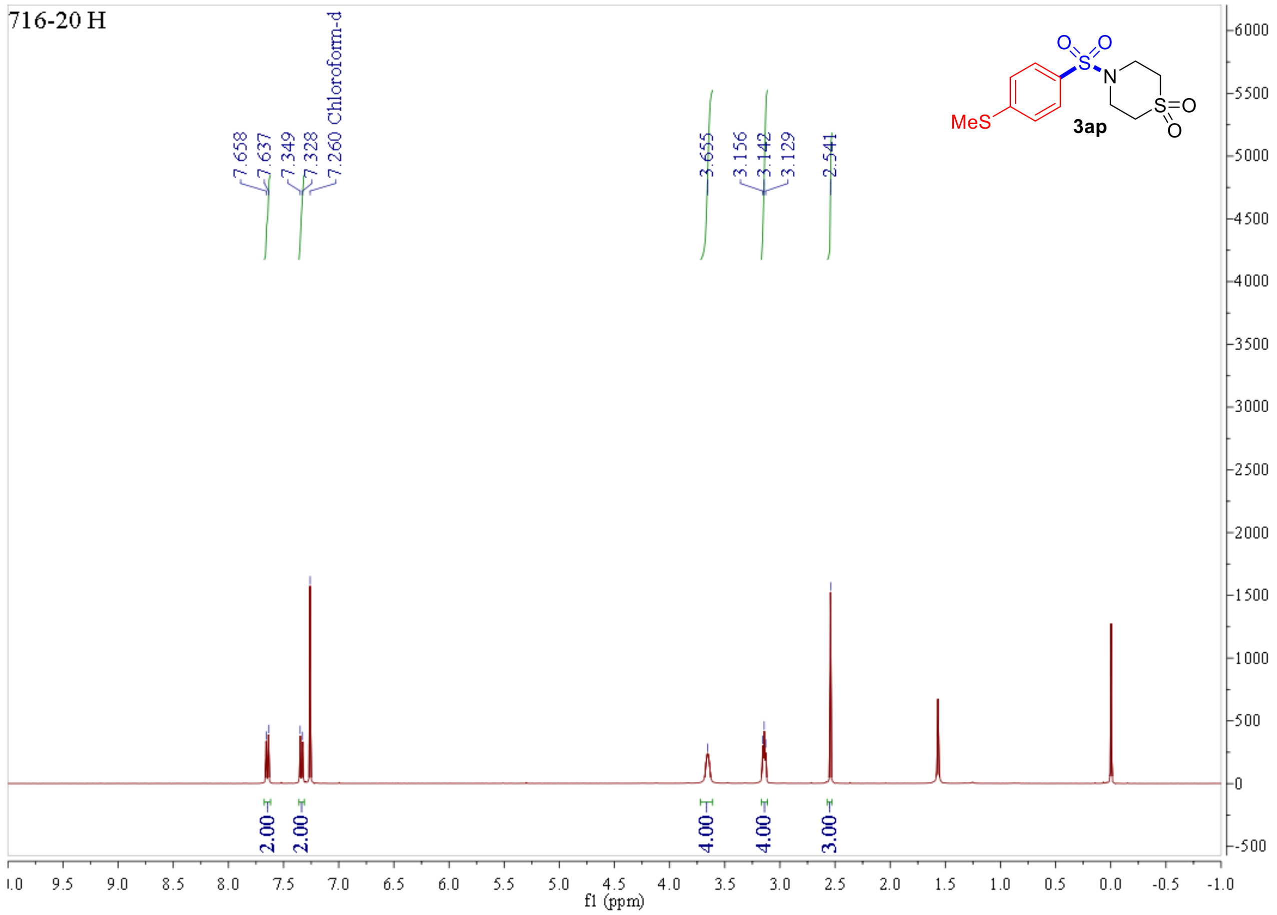
716-20 H
7.658
7.637
7.349
7.328
7.260 Chloroform-d
3.655
3.156
3.142
3.129
2.541
MeS
3ap
2.00
2.00
4.00
4.00
3.00
f1 (ppm)

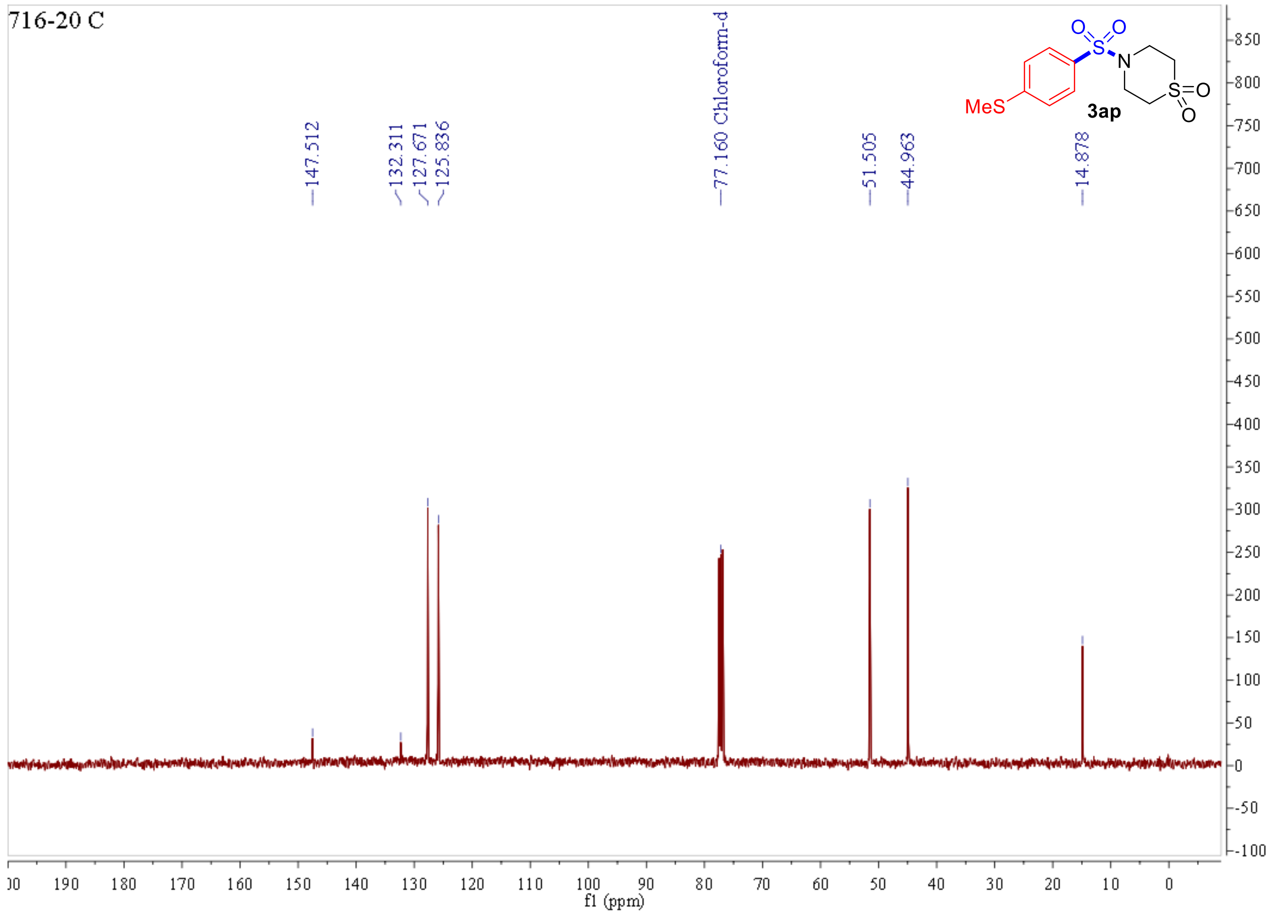
716-20 C
147.512
132.311
127.671
125.836
77.160 Chloroform-d
51.505
44.963
14.878
MeS
3ap
f1 (ppm)

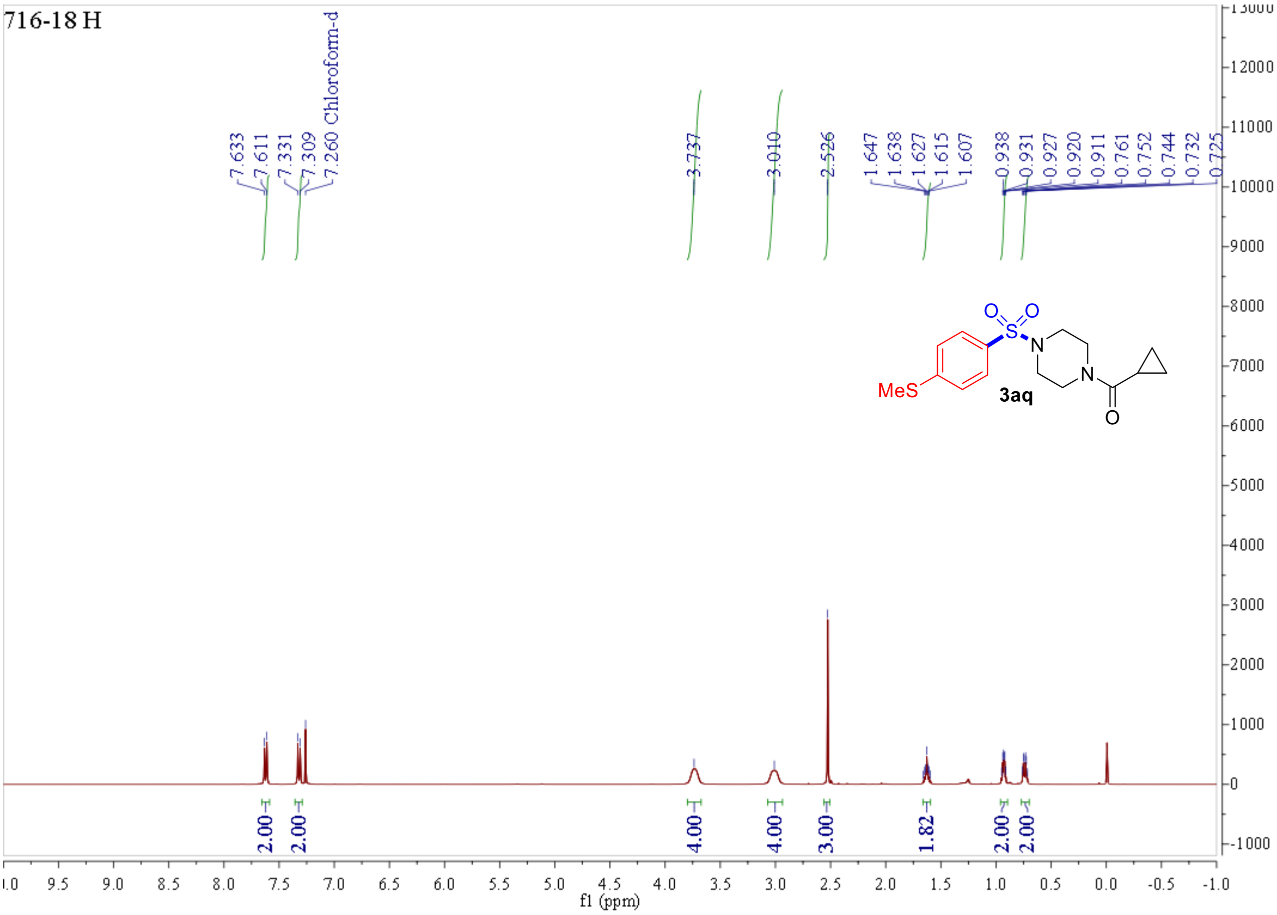
716-18 H
7.633
7.611
7.331
7.309
7.260 Chloroform-d
3.737
3.010
2.526
1.647
1.638
1.627
1.615
1.607
0.938
0.931
0.927
0.920
0.911
0.761
0.752
0.744
0.732
0.725
MeS
3aq
2.00
2.00
4.00
4.00
3.00
1.82
2.00
2.00
f1 (ppm)

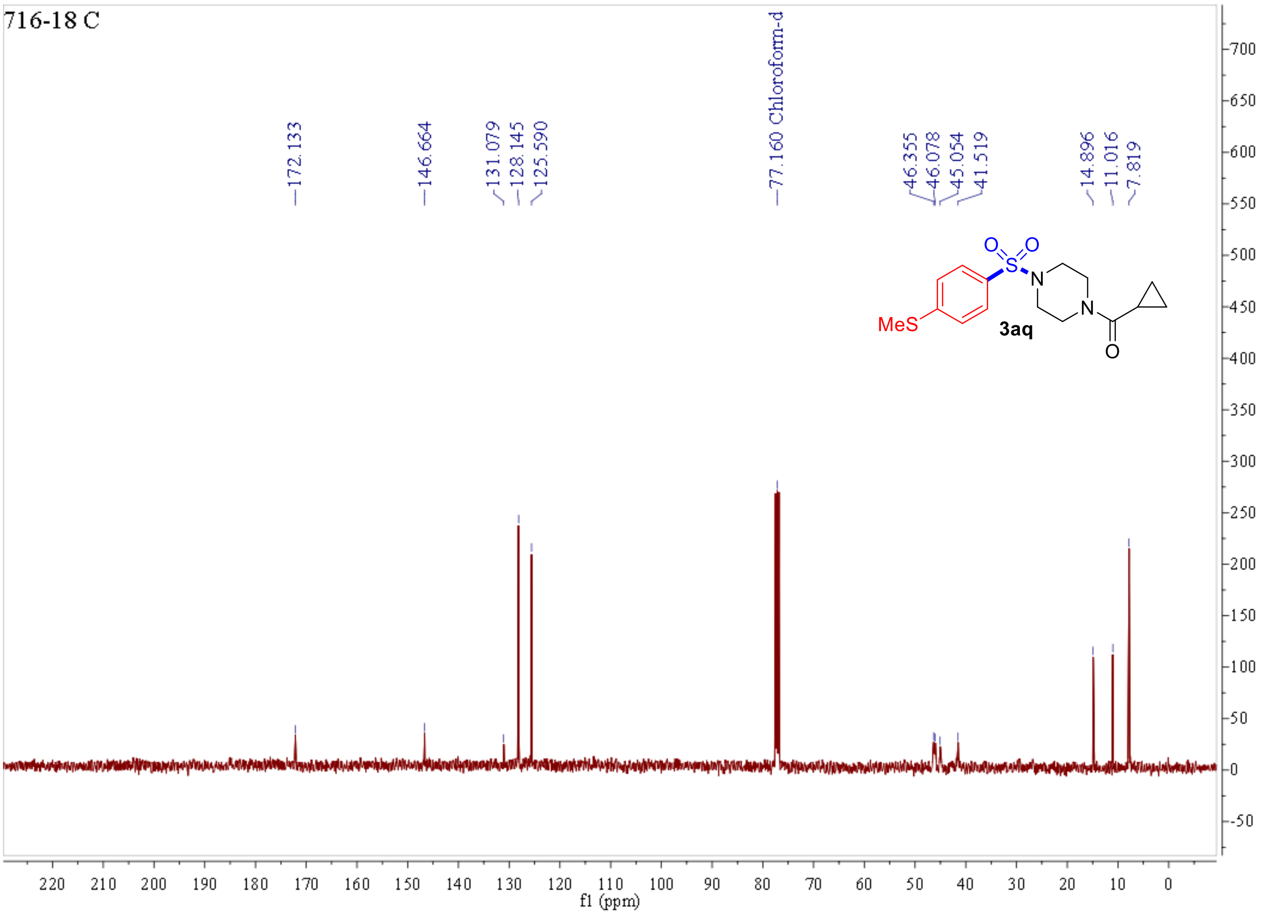
716-18 C
172.133
146.664
131.079
128.145
125.590
77.160 Chloroform-d
46.355
46.078
45.054
41.519
14.896
11.016
7.819
MeS
3aq
f1 (ppm)

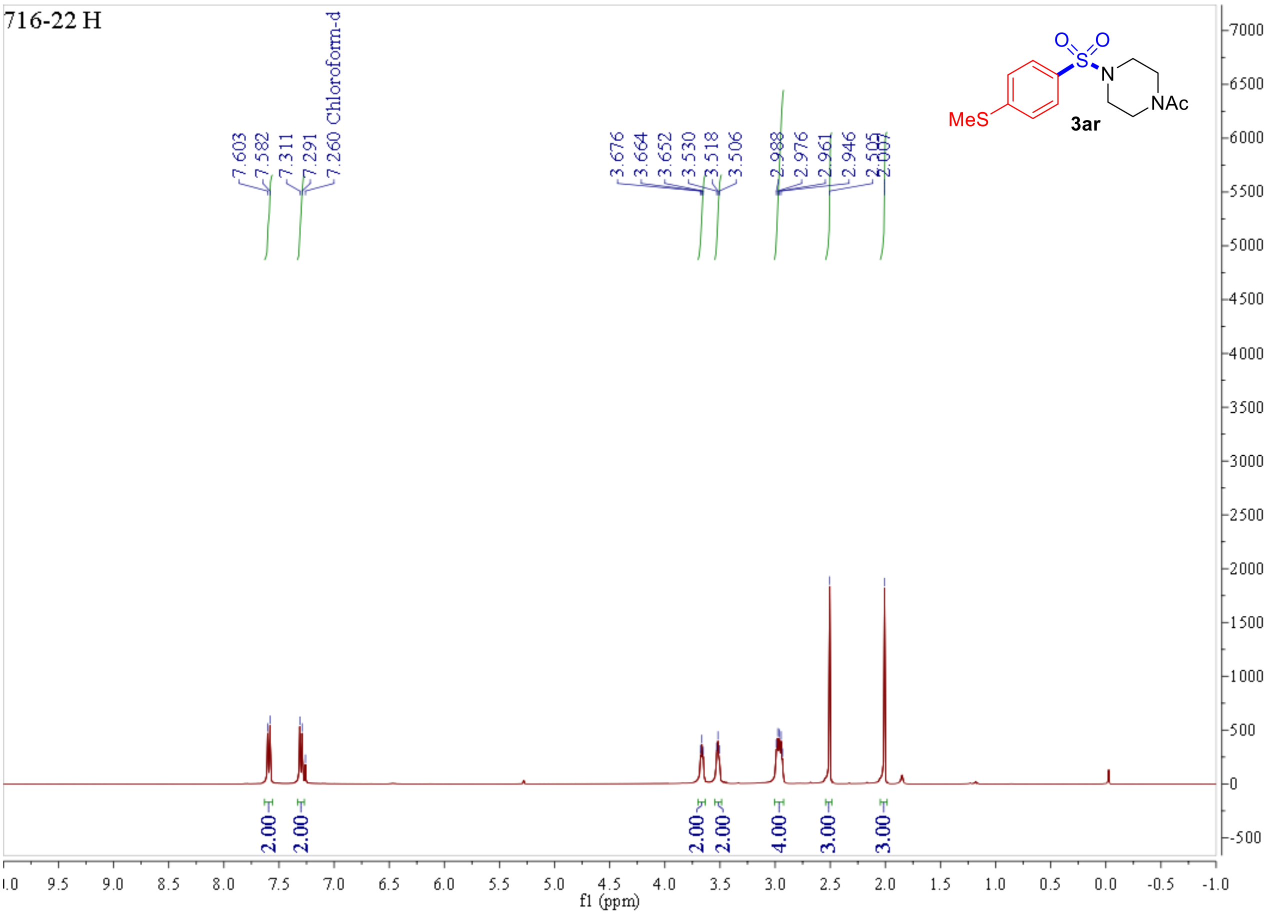
716-22 H
7.603
7.582
7.311
7.291
7.260 Chloroform-d
3.676
3.664
3.652
3.530
3.518
3.506
2.988
2.976
2.961
2.946
MeS
3ar
NAc
2.00
2.00
2.00
2.00
4.00
3.00
3.00
f1 (ppm)

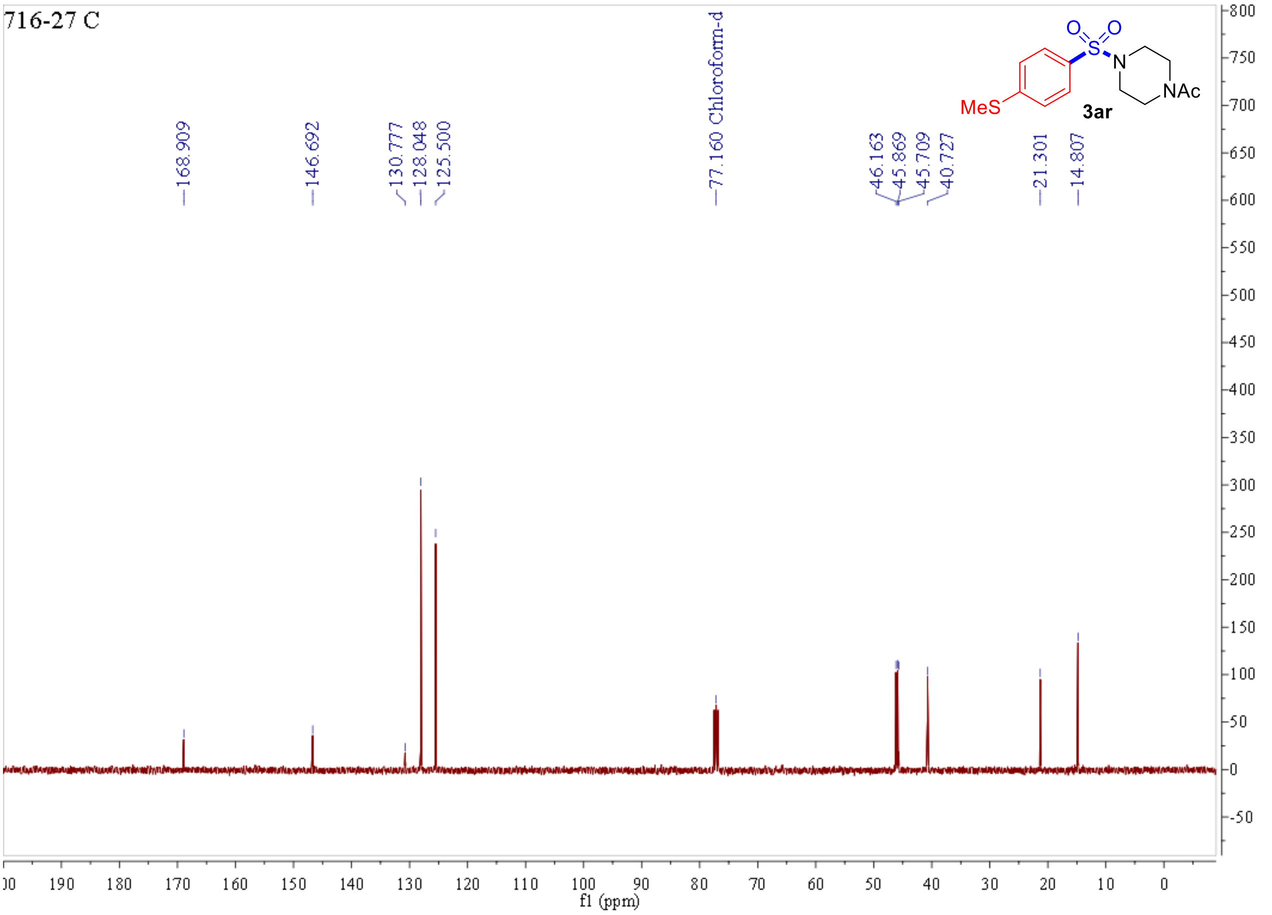
716-27 C
168.909
146.692
130.777
128.048
125.500
77.160 Chloroform-d
46.163
45.869
45.709
40.727
21.301
14.807
MeS
3ar
NAc
f1 (ppm)

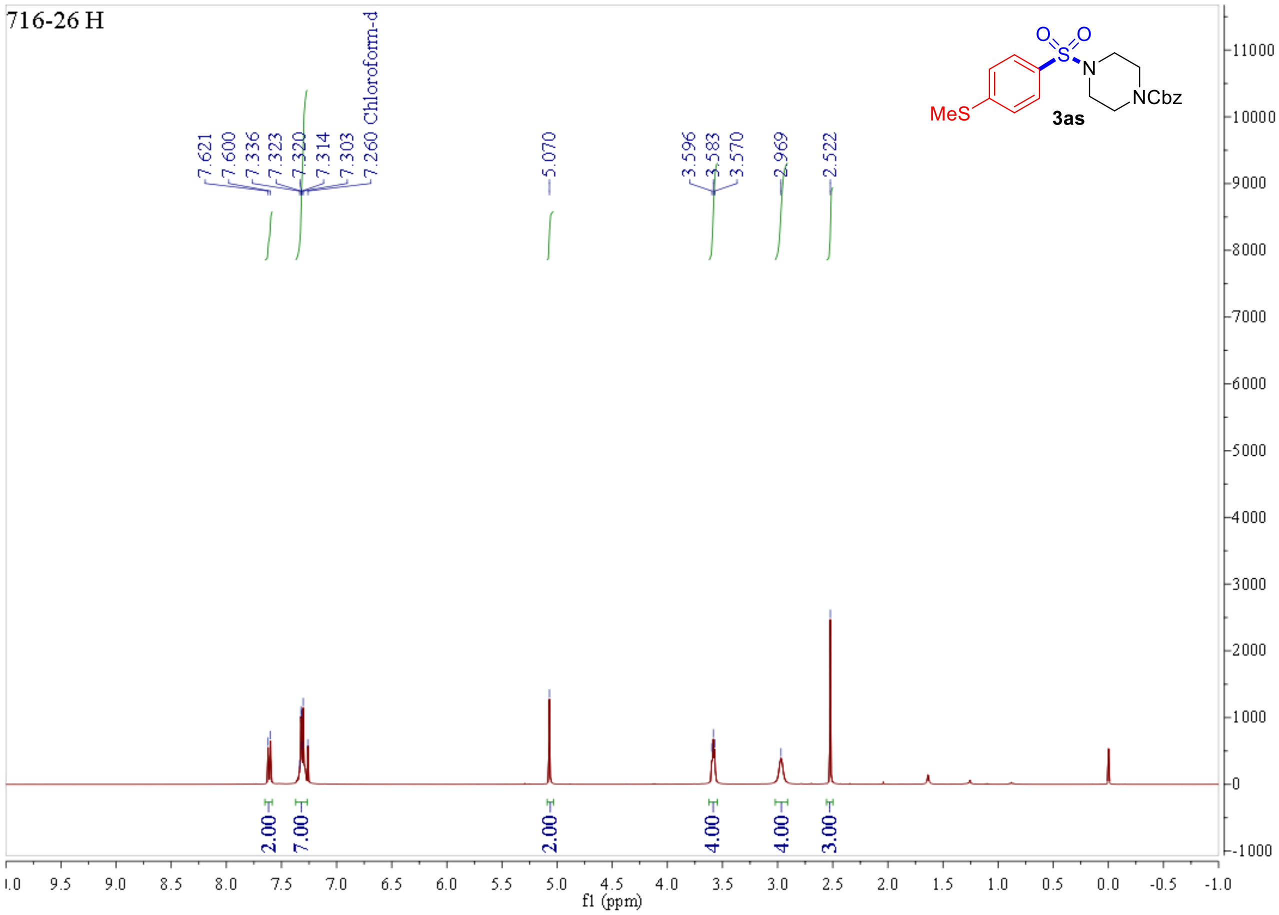
716-26 H
7.621
7.600
7.336
7.323
7.320
7.314
7.303
7.260 Chloroform-d
5.070
3.596
3.583
3.570
2.969
2.522
MeS
O
S
O
N
NCbz
3as
2.00
7.00
2.00
4.00
4.00
3.00
f1 (ppm)

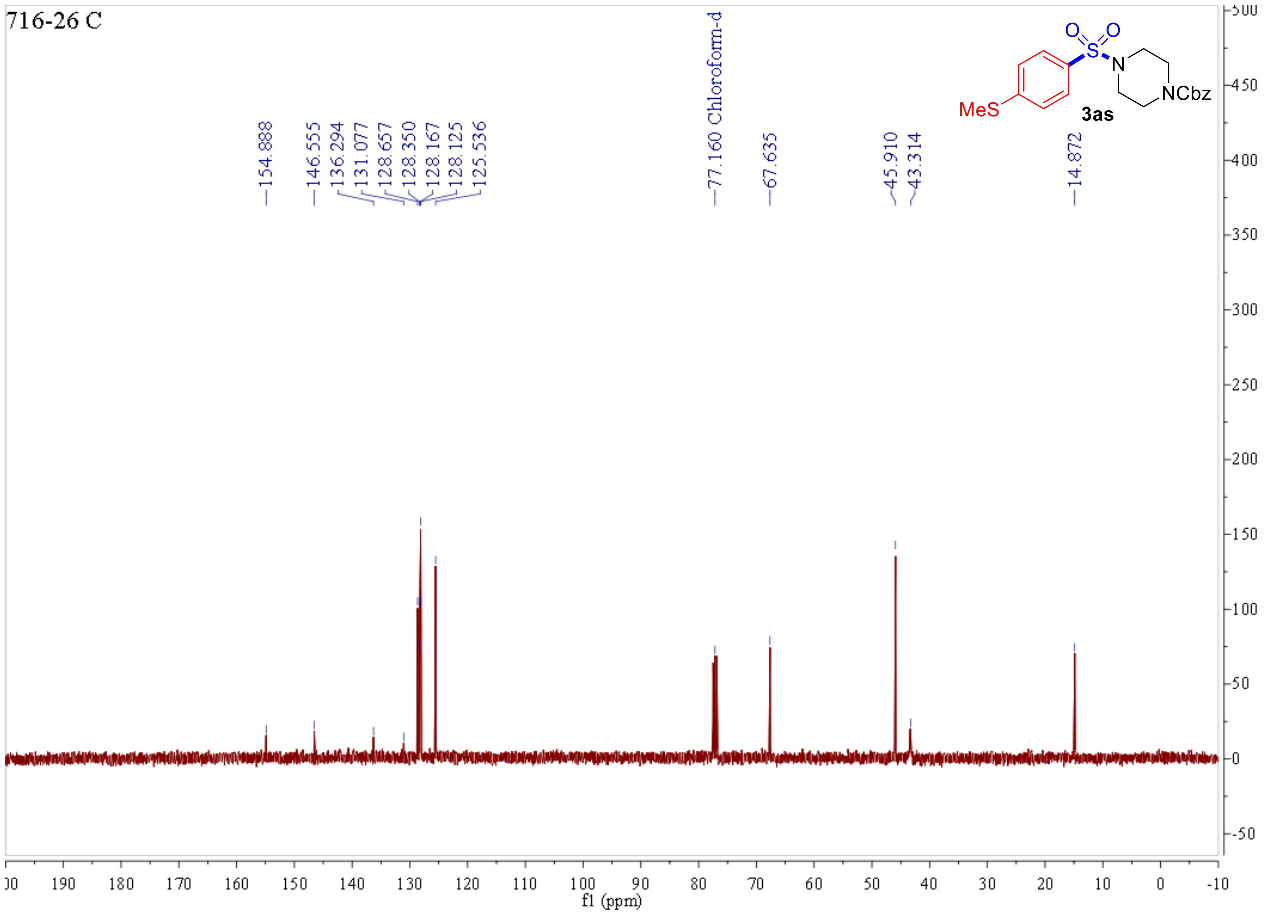
716-26 C
154.888
146.555
136.294
131.077
128.657
128.350
128.167
128.125
125.536
77.160 Chloroform-d
67.635
45.910
43.314
14.872
MeS
O
S
O
N
NCbz
3as
f1 (ppm)

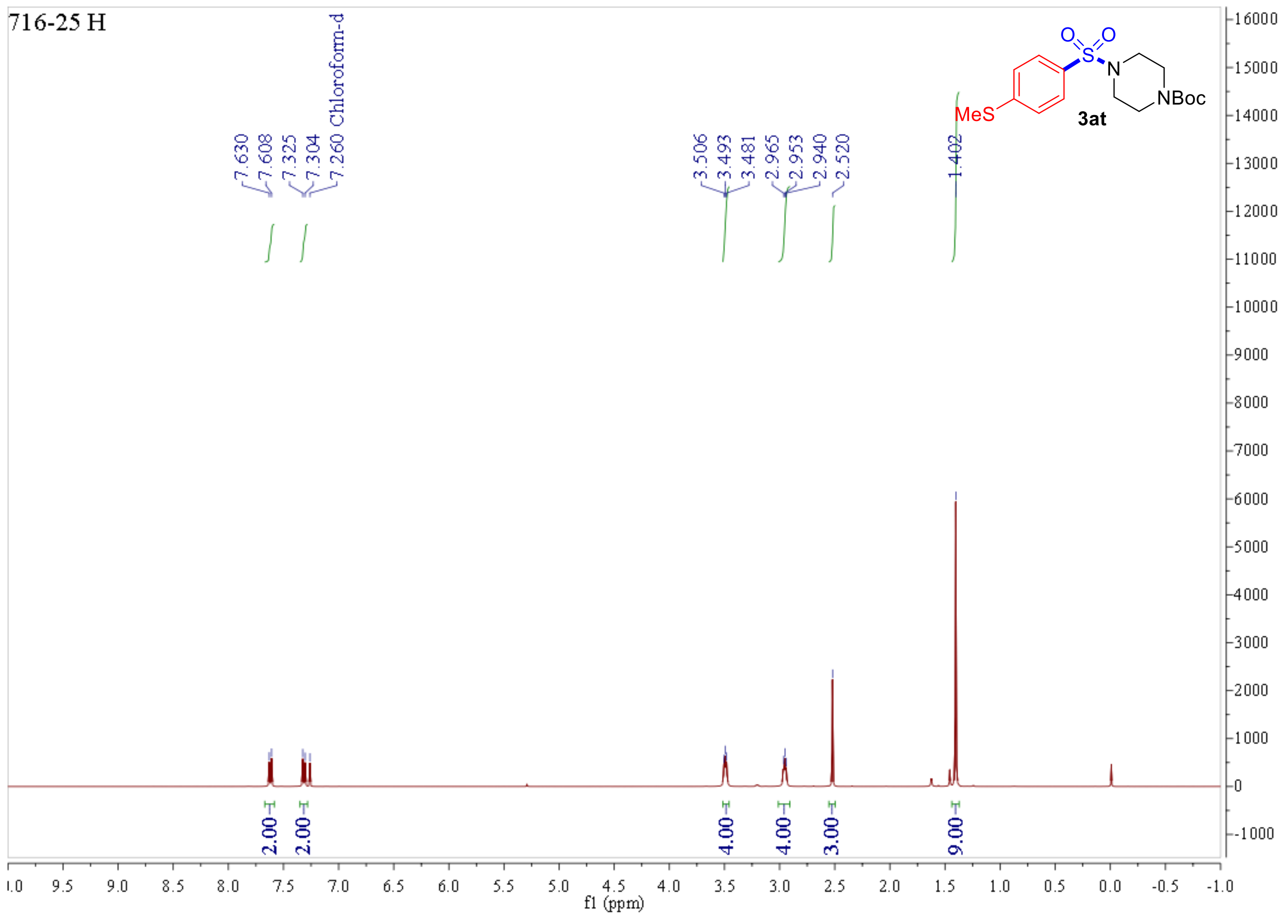

716-25 H
7.630
7.608
7.325
7.304
7.260 Chloroform-d
3.506
3.493
3.481
2.965
2.953
2.940
2.520
1.402
MeS
O
S
O
N
NBoc
3at
2.00
2.00
4.00
4.00
3.00
9.00
f1 (ppm)

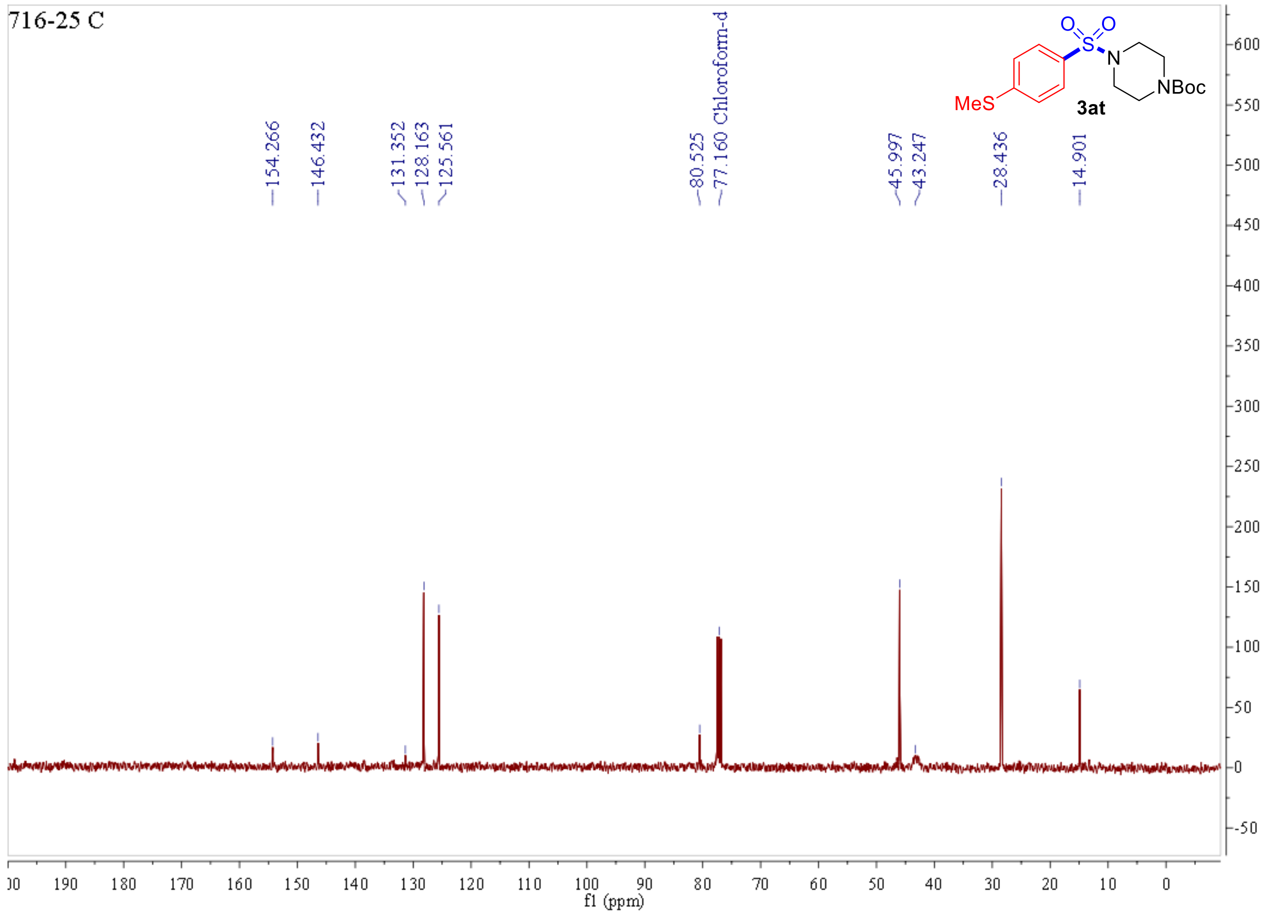

716-25 C
154.266
146.432
131.352
128.163
125.561
80.525
77.160 Chloroform-d
45.997
43.247
28.436
14.901
MeS
O
S
O
N
NBoc
3at
f1 (ppm)

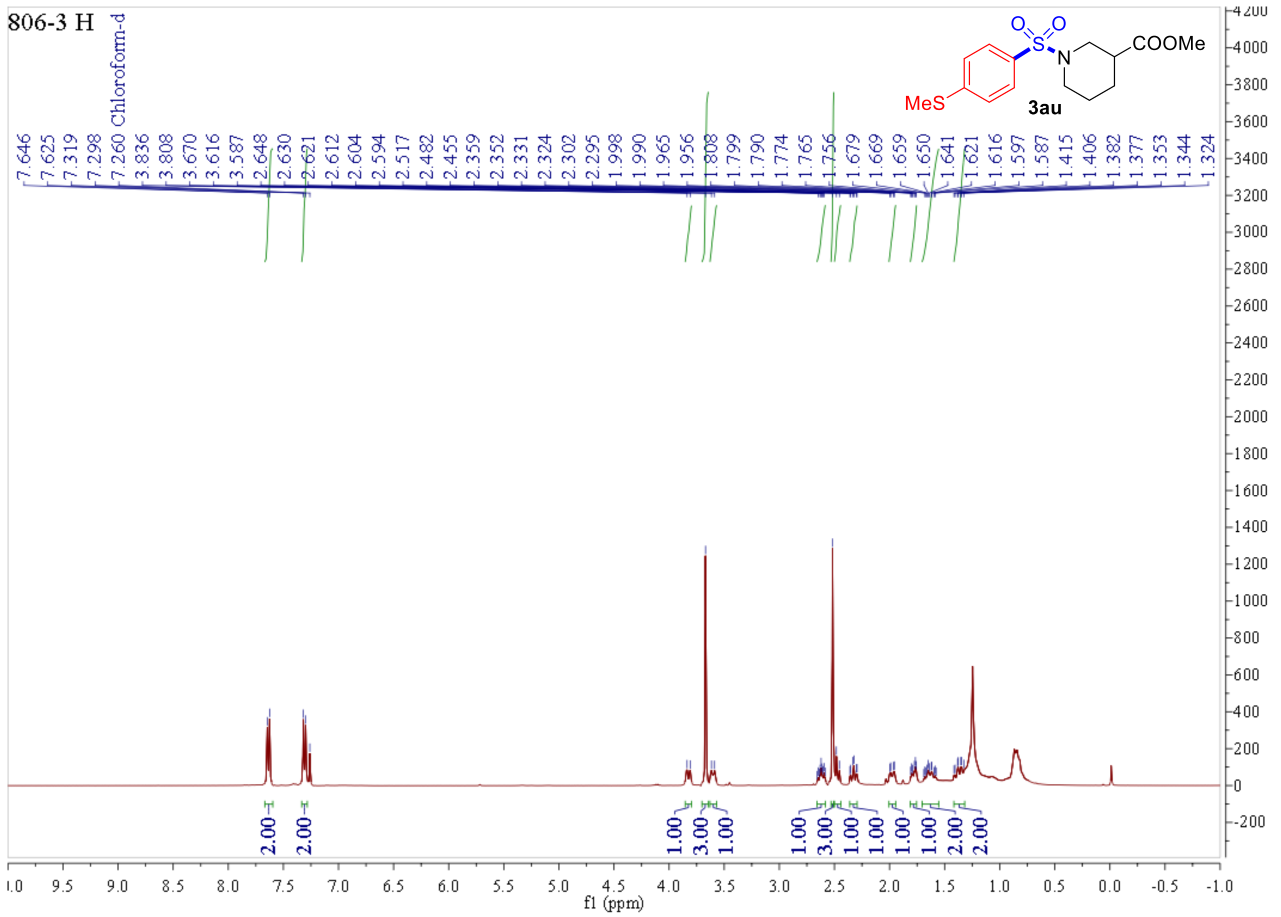
806-3 H
3au
MeS
COOMe
7.646 7.625 7.319 7.298 7.260 Chloroform-d 3.836 3.808 3.670 3.616 3.587 2.648 2.630 2.621 2.612 2.604 2.594 2.517 2.482 2.455 2.359 2.352 2.331 2.324 2.302 2.295 1.998 1.990 1.965 1.956 1.808 1.799 1.790 1.774 1.765 1.756 1.679 1.669 1.659 1.650 1.641 1.621 1.616 1.597 1.587 1.415 1.406 1.382 1.377 1.353 1.344 1.324
2.00 2.00 1.00 3.00 1.00 1.00 3.00 1.00 1.00 1.00 1.00 2.00 2.00
f1 (ppm)

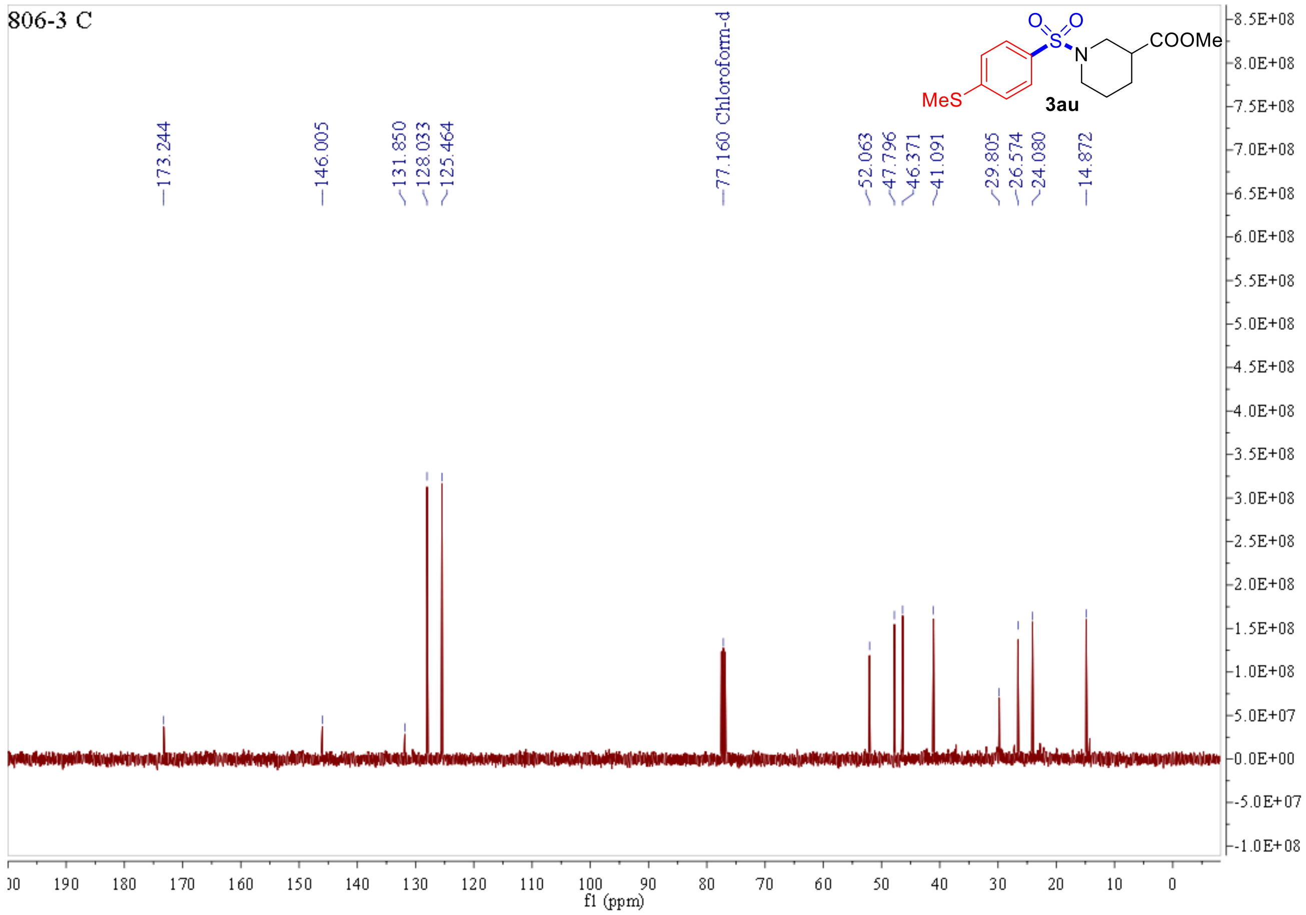
806-3 C
3au
MeS
COOMe
173.244 146.005 131.850 128.033 125.464 77.160 Chloroform-d 52.063 47.796 46.371 41.091 29.805 26.574 24.080 14.872
f1 (ppm)

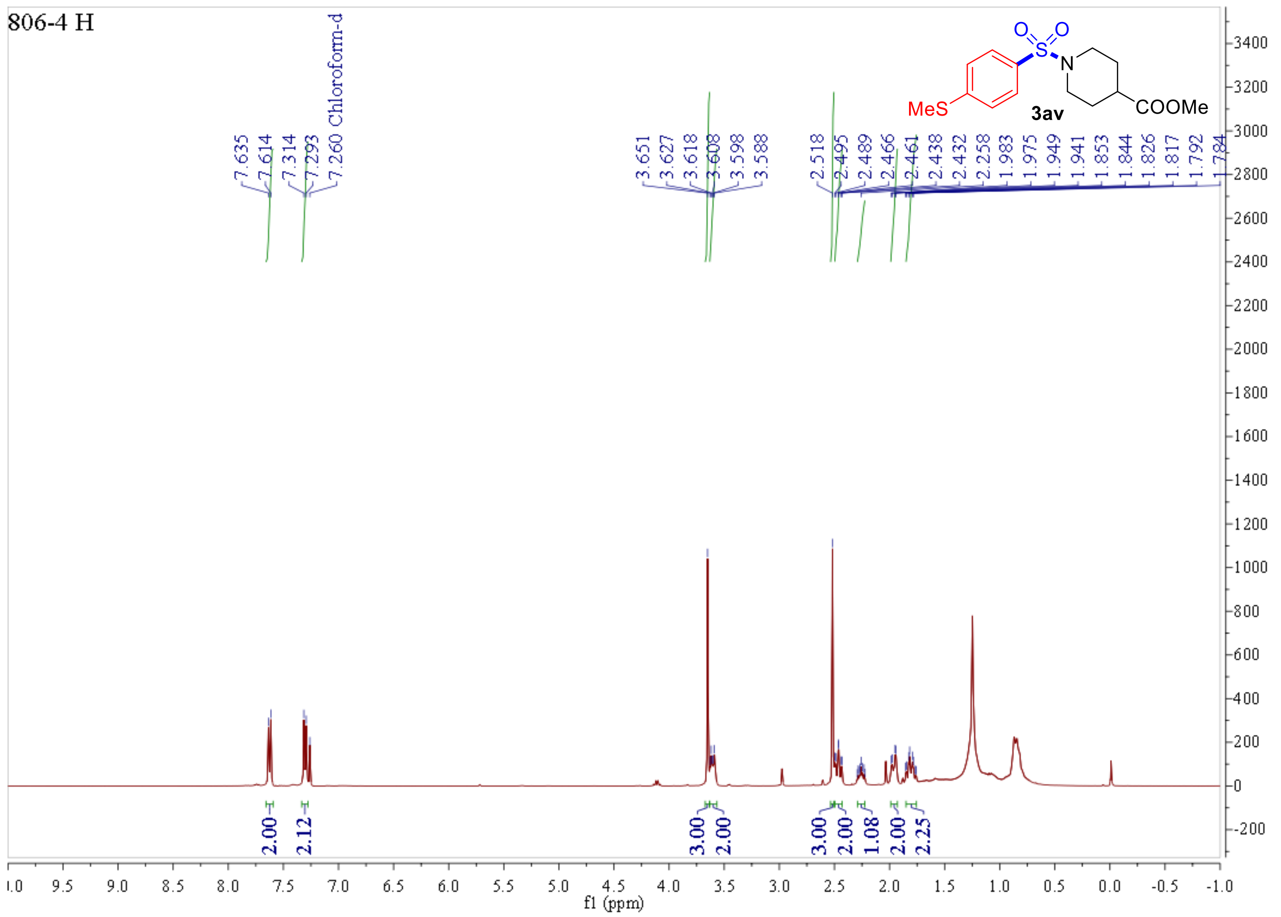
806-4 H
7.635
7.614
7.314
7.293
7.260 Chloroform-d
3.651
3.627
3.618
3.608
3.598
3.588
2.518
2.495
2.489
2.466
2.461
2.438
2.432
2.258
1.983
1.975
1.949
1.941
1.853
1.844
1.826
1.817
1.792
1.784
MeS
3av
COOMe
2.00
2.12
3.00
2.00
3.00
2.00
1.08
2.00
2.25
f1 (ppm)

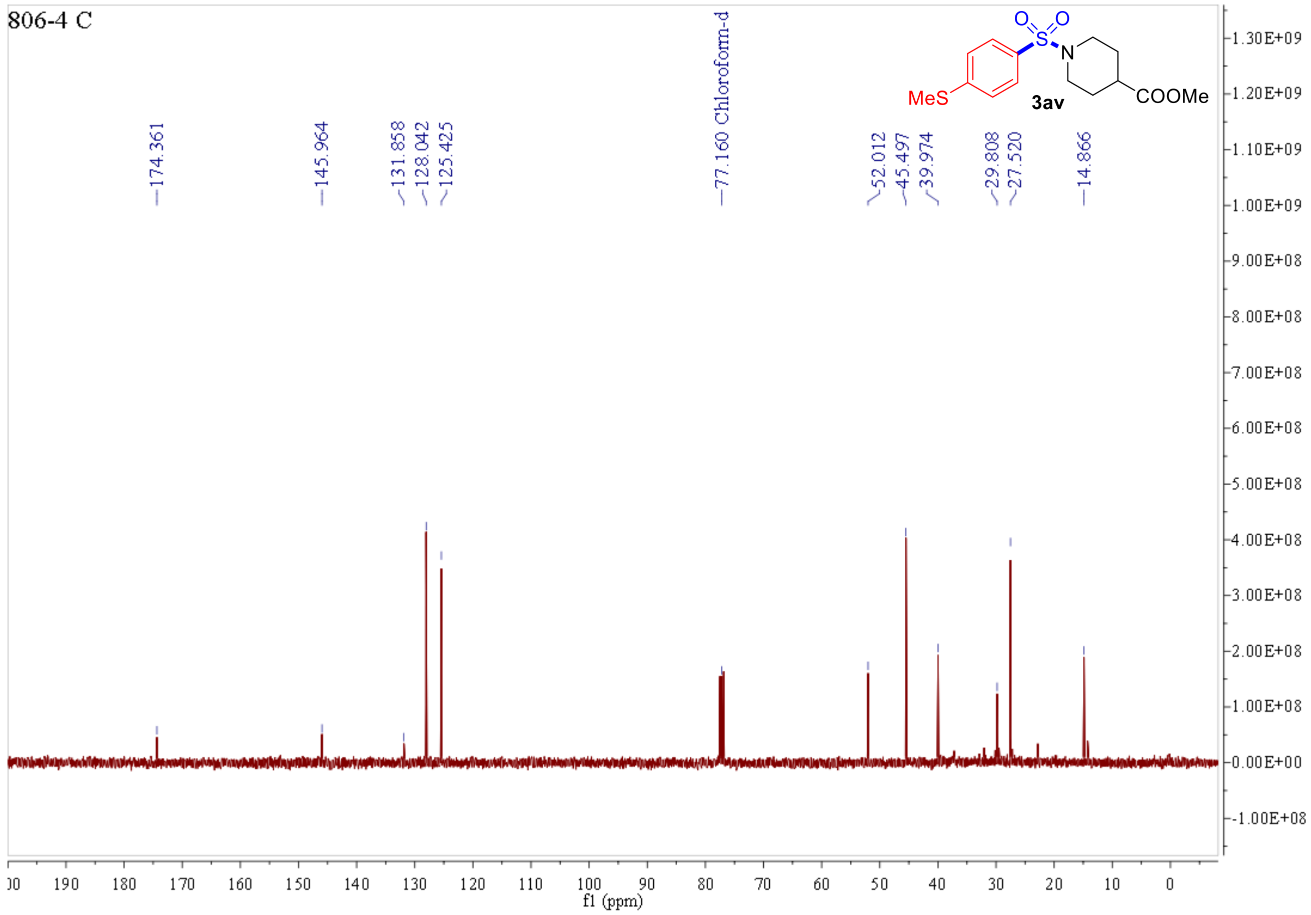
806-4 C
174.361
145.964
131.858
128.042
125.425
77.160 Chloroform-d
52.012
45.497
39.974
29.808
27.520
14.866
MeS
3av
COOMe
f1 (ppm)

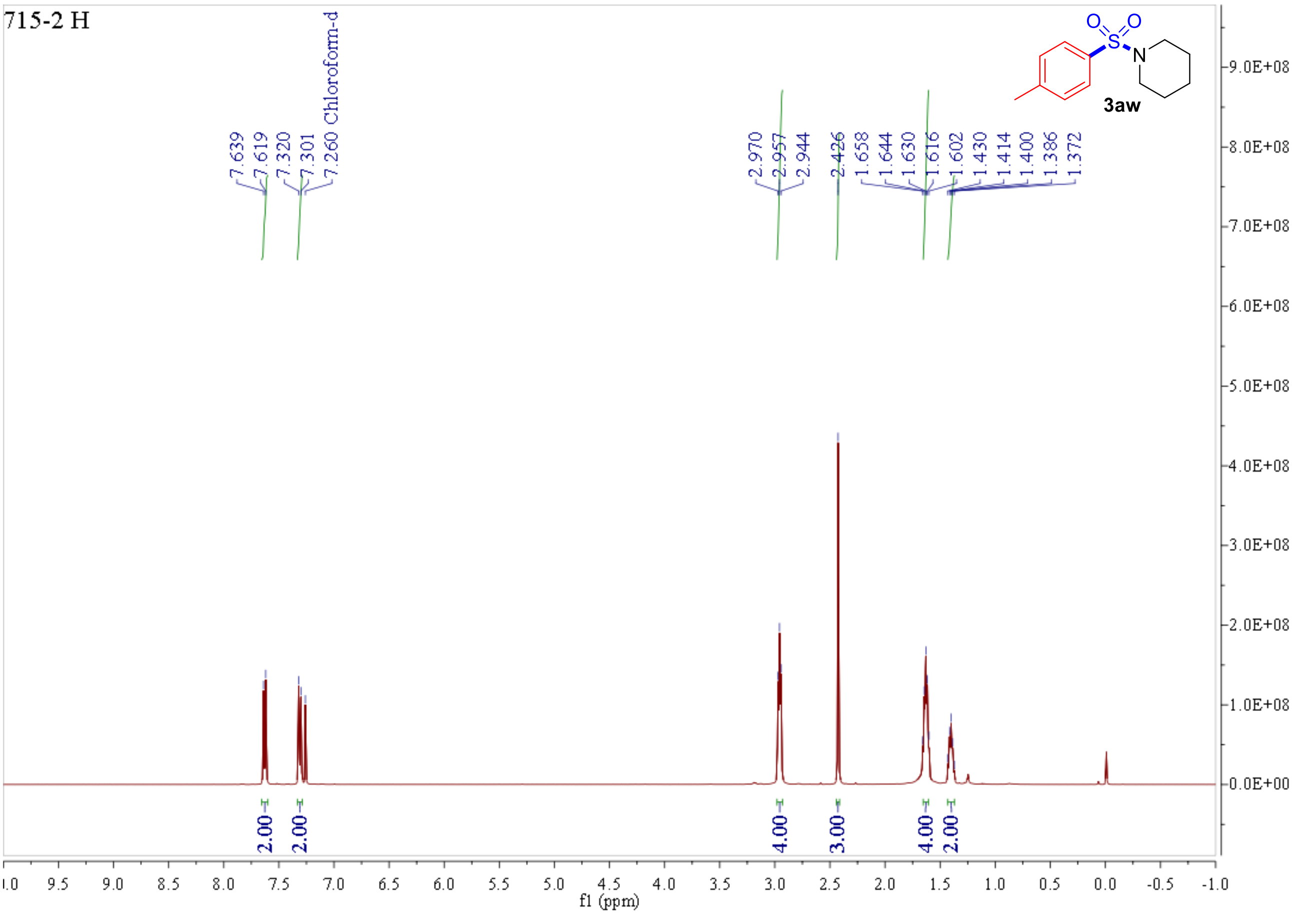
715-2 H
7.639
7.619
7.320
7.301
7.260 Chloroform-d
2.970
2.957
2.944
2.426
1.658
1.644
1.630
1.616
1.602
1.430
1.414
1.400
1.386
1.372
3aw
2.00
2.00
4.00
3.00
4.00
2.00
f1 (ppm)

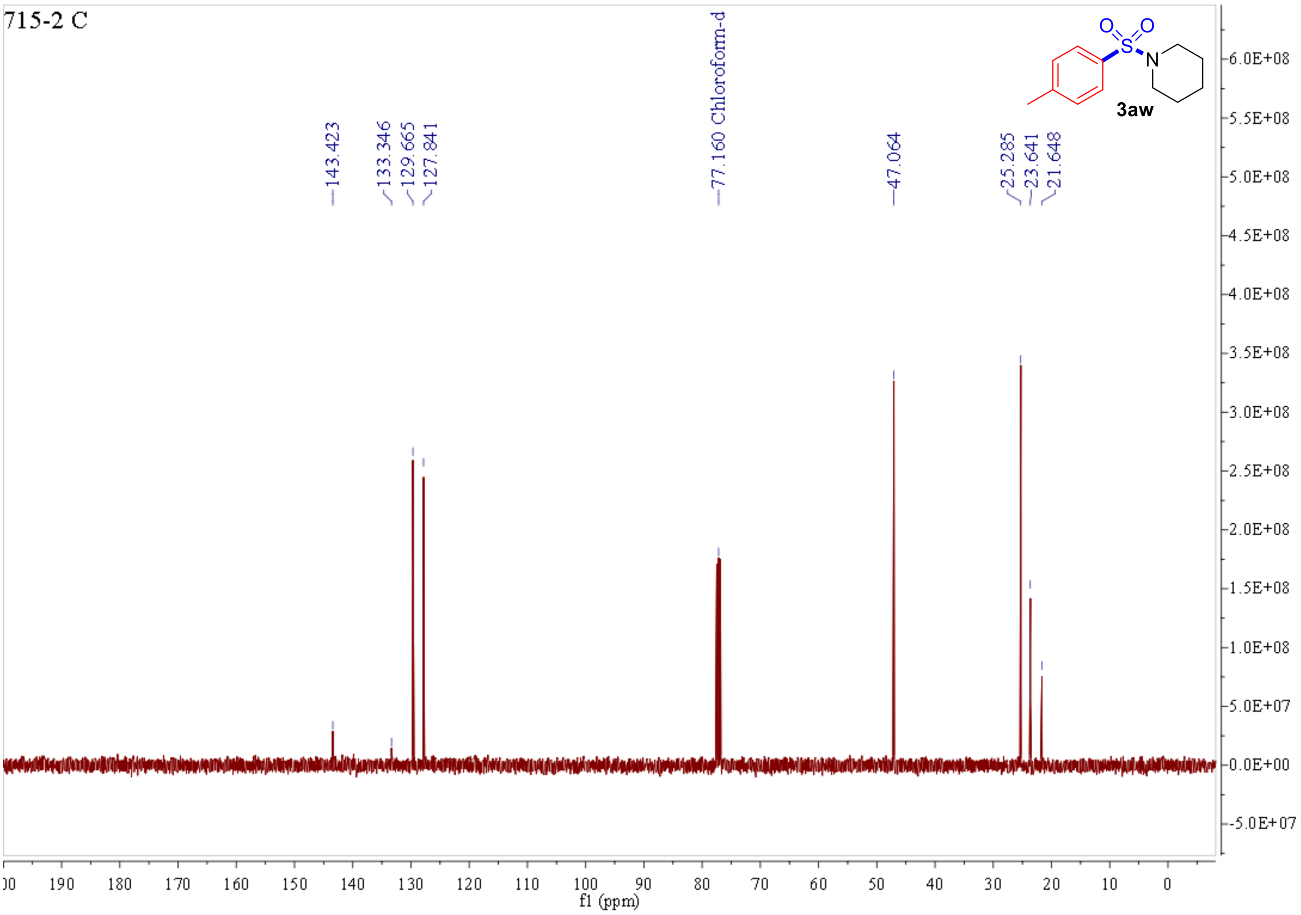
715-2 C
143.423
133.346
129.665
127.841
77.160 Chloroform-d
47.064
25.285
23.641
21.648
3aw
f1 (ppm)

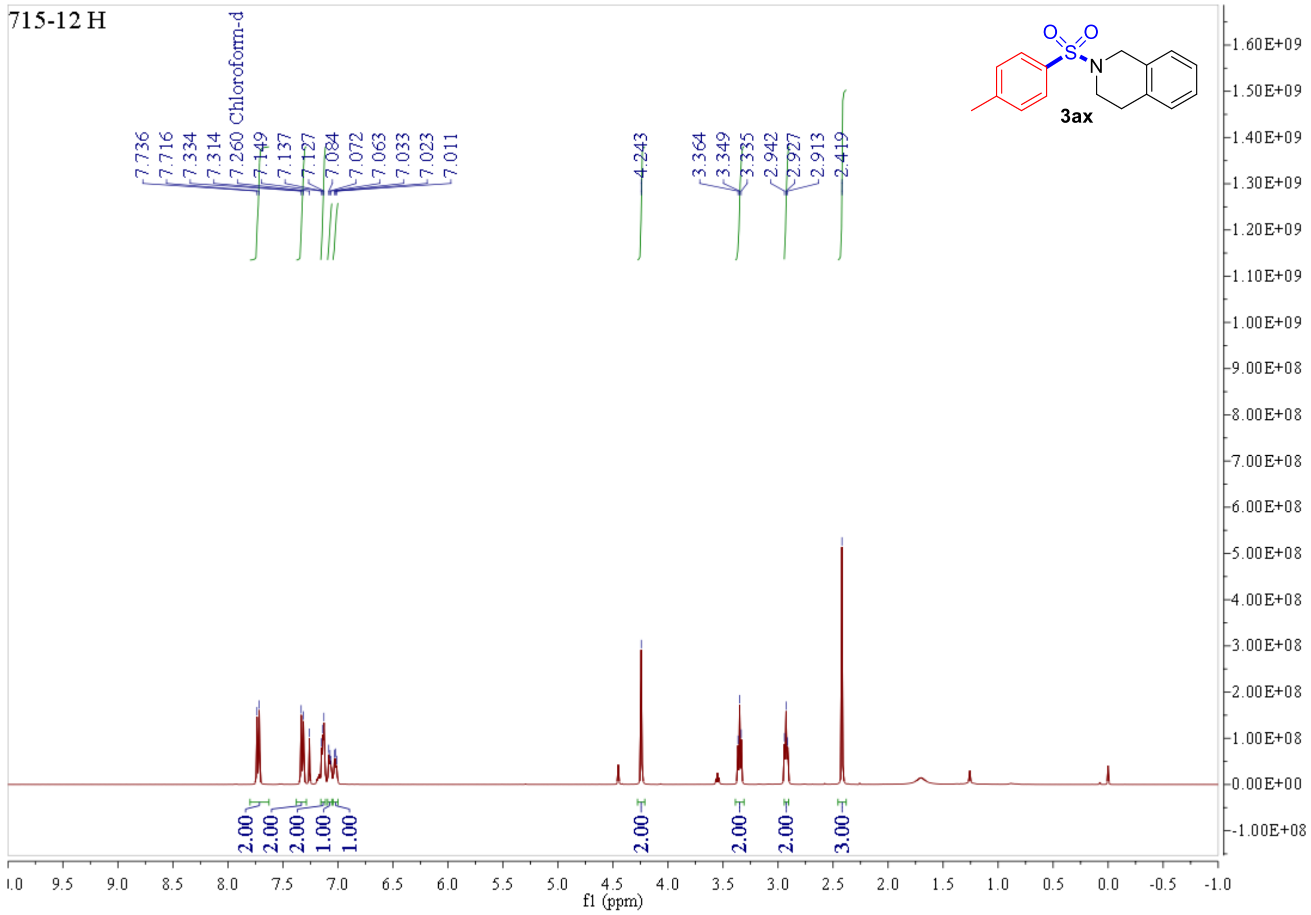
715-12 H
3ax
7.736
7.716
7.334
7.314
7.260 Chloroform-d
7.149
7.137
7.127
7.084
7.072
7.063
7.033
7.023
7.011
4.243
3.364
3.349
3.335
2.942
2.927
2.913
2.419
2.00
2.00
2.00
1.00
1.00
2.00
2.00
2.00
3.00
f1 (ppm)

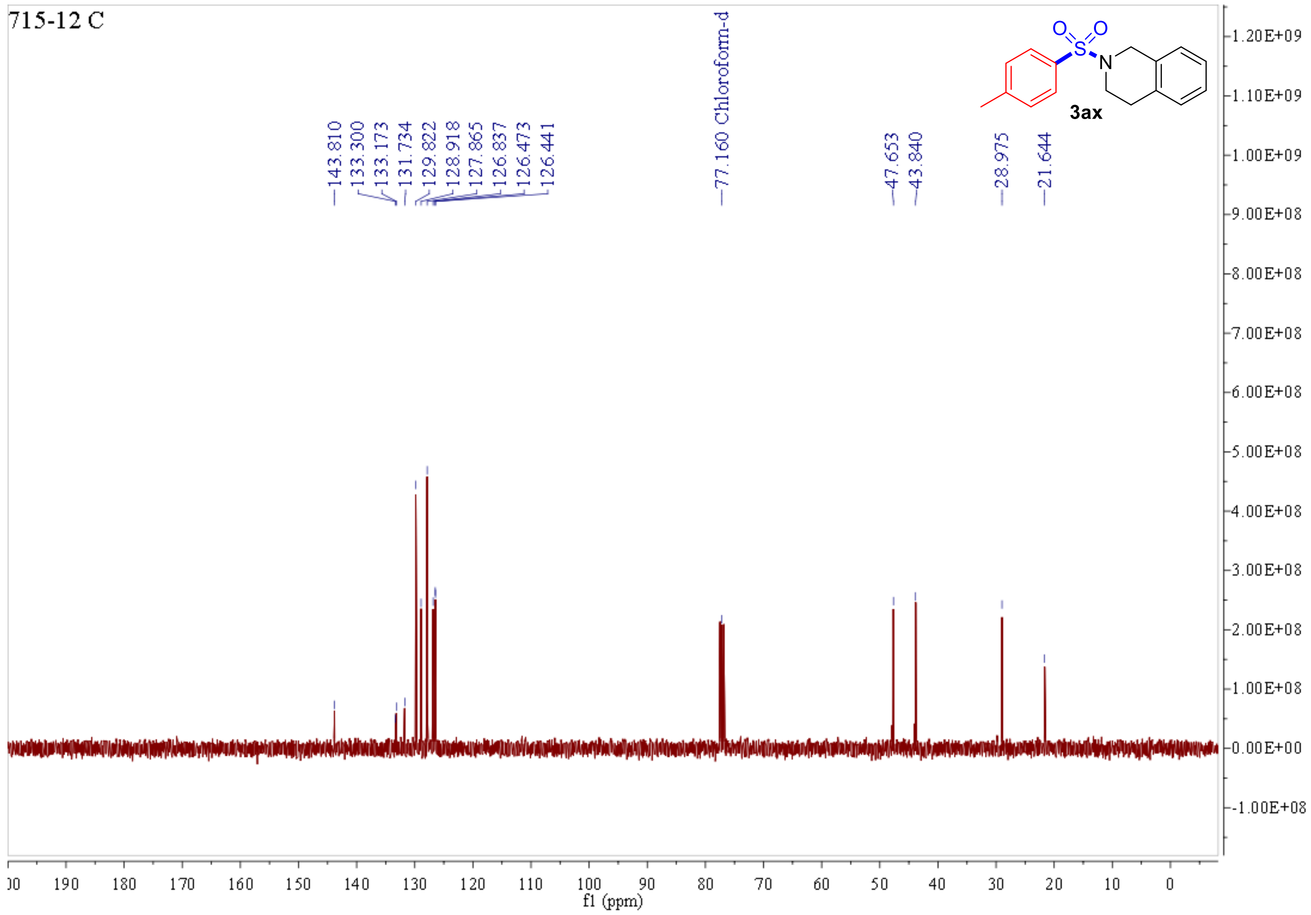
715-12 C
3ax
143.810
133.300
133.173
131.734
129.822
128.918
127.865
126.837
126.473
126.441
77.160 Chloroform-d
47.653
43.840
28.975
21.644
f1 (ppm)

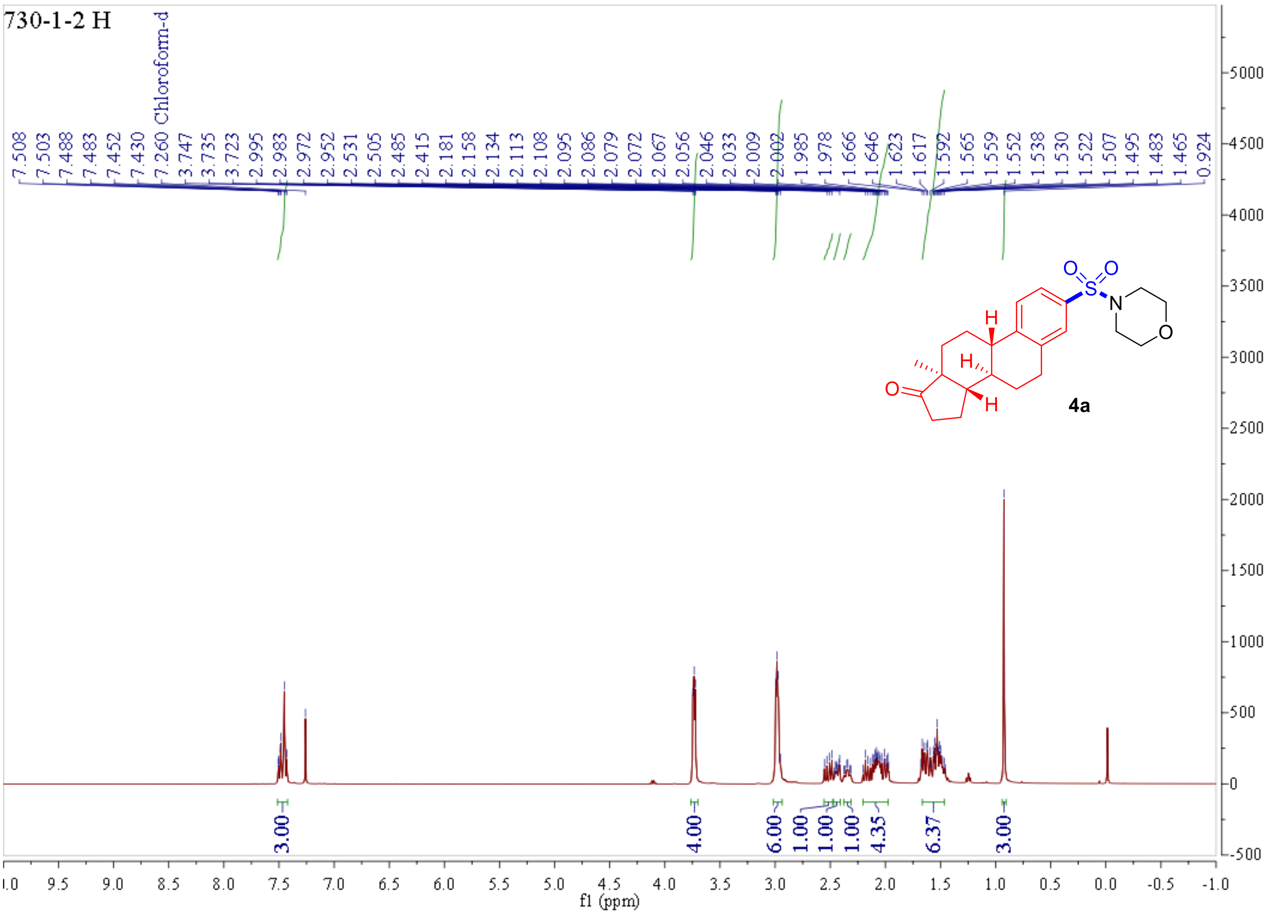
730-1-2 H
7.508
7.503
7.488
7.483
7.452
7.430
7.260 Chloroform-d
3.747
3.735
3.723
2.995
2.983
2.972
2.952
2.531
2.505
2.485
2.415
2.181
2.158
2.134
2.113
2.108
2.095
2.086
2.079
2.072
2.067
2.056
2.046
2.033
2.009
2.002
1.985
1.978
1.666
1.646
1.623
1.617
1.592
1.565
1.559
1.552
1.538
1.530
1.522
1.507
1.495
1.483
1.465
0.924
3.00
4.00
6.00
1.00
1.00
1.00
4.35
6.37
3.00
4a
f1 (ppm)

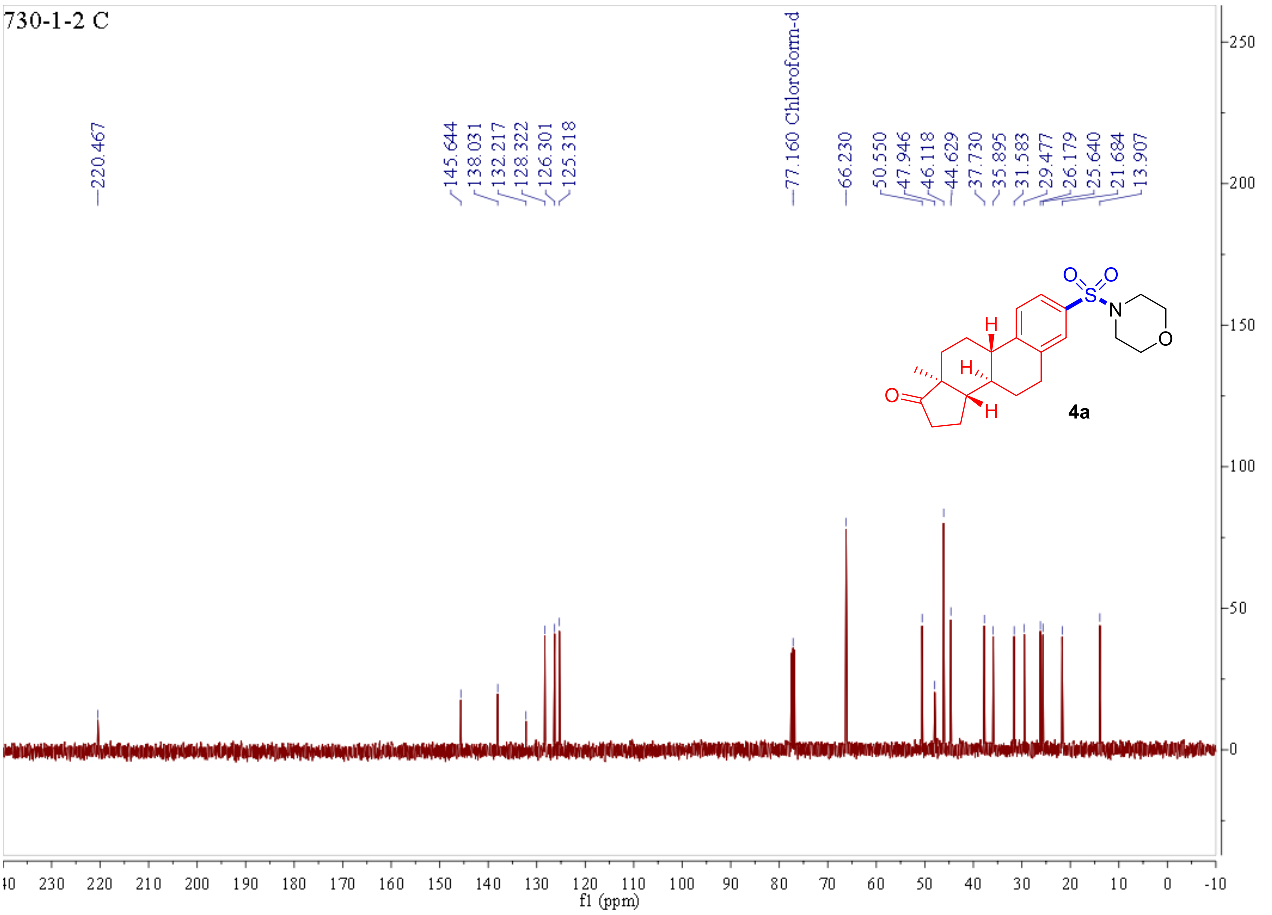
730-1-2 C
220.467
145.644
138.031
132.217
128.322
126.301
125.318
77.160 Chloroform-d
66.230
50.550
47.946
46.118
44.629
37.730
35.895
31.583
29.477
26.179
25.640
21.684
13.907
4a
f1 (ppm)

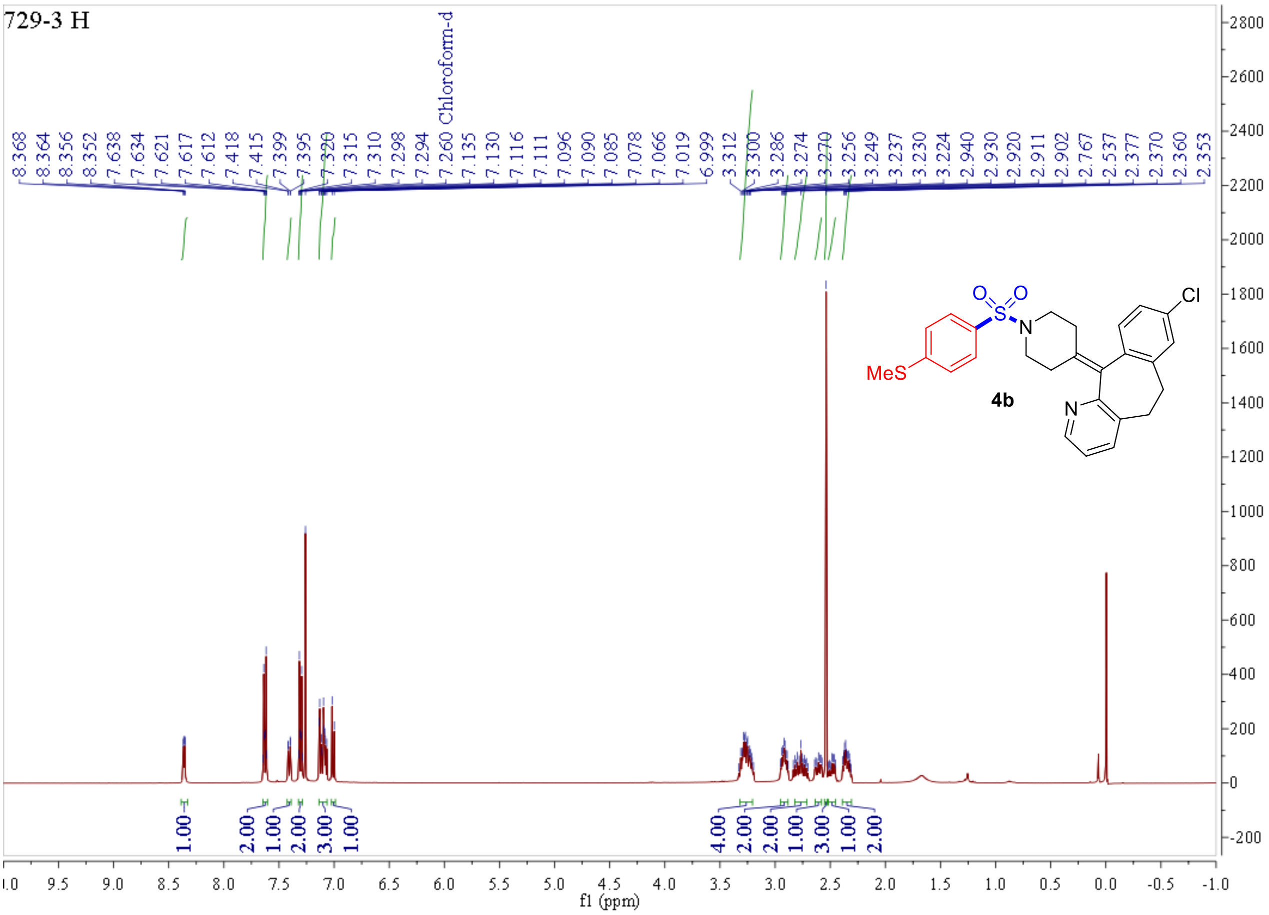

729-3 H
8.368
8.364
8.356
8.352
7.638
7.634
7.621
7.617
7.612
7.418
7.415
7.399
7.395
7.320
7.315
7.310
7.298
7.294
7.260 Chloroform-d
7.135
7.130
7.116
7.111
7.096
7.090
7.085
7.078
7.066
7.019
6.999
3.312
3.300
3.286
3.274
3.270
3.256
3.249
3.237
3.230
3.224
2.940
2.930
2.920
2.911
2.902
2.767
2.537
2.377
2.370
2.360
2.353
1.00
2.00
1.00
2.00
3.00
1.00
4.00
2.00
2.00
1.00
3.00
1.00
2.00
MeS
O
S
O
N
Cl
N
4b
f1 (ppm)

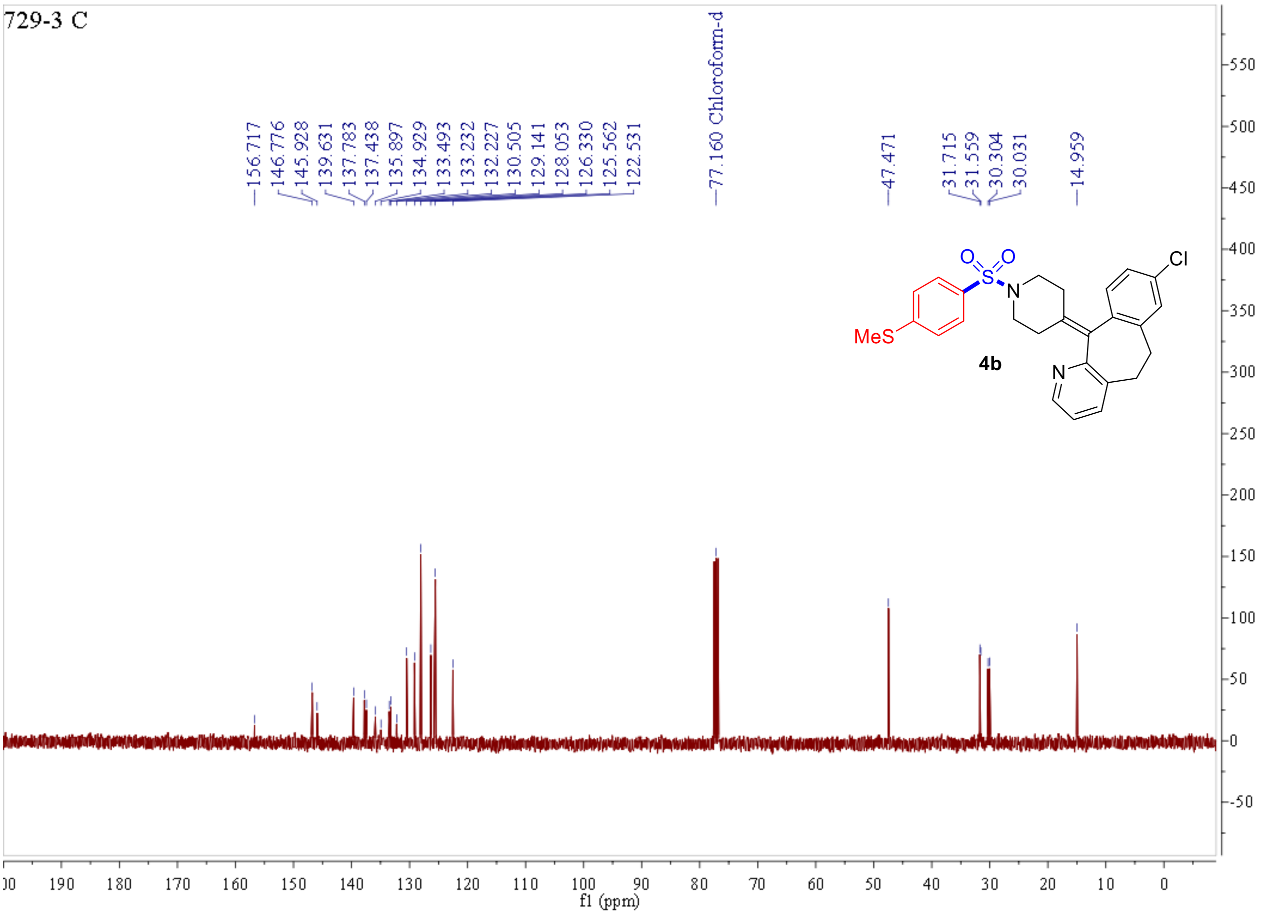

729-3 C
156.717
146.776
145.928
139.631
137.783
137.438
135.897
134.929
133.493
133.232
132.227
130.505
129.141
128.053
126.330
125.562
122.531
77.160 Chloroform-d
47.471
31.715
31.559
30.304
30.031
14.959
MeS
O
S
O
N
Cl
N
4b
f1 (ppm)
